# Predictive functional, statistical and structural analysis of *CSNK2A1* and *CSNK2B* variants linked to neurodevelopmental diseases

**DOI:** 10.3389/fmolb.2022.851547

**Published:** 2022-10-13

**Authors:** Prasida Unni, Jack Friend, Janice Weinberg, Volkan Okur, Jennifer Hochscherf, Isabel Dominguez

**Affiliations:** ^1^ Department of Medicine, Boston University School of Medicine and Boston Medical Center, Boston University, Boston, MA, United States; ^2^ Department of Biostatistics, Boston University School of Public Health, Boston University, Boston, MA, United States; ^3^ New York Genome Center, New York, NY, United States; ^4^ Department of Chemistry, Institute of Biochemistry, University of Cologne, Cologne, Germany

**Keywords:** CK2α, CK2β, protein kinase CK2, Okur-Chung neurodevelopmental syndrome (OCNDS), Poirier-Bienvenu neurodevelopmental syndrome (POBINDS), autism spectrum disorder (ASD), epilepsy, non-random mutation clustering (NMC)

## Abstract

Okur-Chung Neurodevelopmental Syndrome (OCNDS) and Poirier-Bienvenu Neurodevelopmental Syndrome (POBINDS) were recently identified as rare neurodevelopmental disorders. OCNDS and POBINDS are associated with heterozygous mutations in the *CSNK2A1* and *CSNK2B* genes which encode CK2α, a serine/threonine protein kinase, and CK2β, a regulatory protein, respectively, which together can form a tetrameric enzyme called protein kinase CK2. A challenge in OCNDS and POBINDS is to understand the genetic basis of these diseases and the effect of the various CK2⍺ and CK2β mutations. In this study we have collected all variants available to date in *CSNK2A1* and *CSNK2B*, and identified hotspots. We have investigated CK2⍺ and CK2β missense mutations through prediction programs which consider the evolutionary conservation, functionality and structure or these two proteins, compared these results with published experimental data on CK2α and CK2β mutants, and suggested prediction programs that could help predict changes in functionality of CK2α mutants. We also investigated the potential effect of CK2α and CK2β mutations on the 3D structure of the proteins and in their binding to each other. These results indicate that there are functional and structural consequences of mutation of CK2α and CK2β, and provide a rationale for further study of OCNDS and POBINDS-associated mutations. These data contribute to understanding the genetic and functional basis of these diseases, which is needed to identify their underlying mechanisms.

## Introduction

Okur-Chung Neurodevelopmental Syndrome (OCNDS; OMIM #617062) and Poirier-Bienvenu Neurodevelopmental Syndrome (POBINDS; OMIM # 618732) are rare, novel congenital autosomal-dominant neurodevelopmental conditions. Recently identified by whole exome sequencing, OCNDS and POBINDS are attributed mostly to *de novo* (germline non-inherited) variants in the genes *CSNK2A1* and *CSNK2B*, respectively (Iossifov et al., 2014; Okur et al., 2016; Poirier et al., 2017). Common clinical features of patients with OCNDS include intellectual disability, developmental delay, autism spectrum disorder (ASD), language impairment, ataxia, attention deficit hyperactivity disorder, microcephaly and craniofacial dysmorphisms (Iossifov et al., 2014; Okur et al., 2016; Trinh et al., 2017; Yuen et al., 2017; Akahira-Azuma et al., 2018; Chiu et al., 2018; Owen et al., 2018; Duan et al., 2019; Nakashima et al., 2019; Martinez-Monseny et al., 2020; van der Werf et al., 2020; Wang et al., 2020; Wu R et al., 2020; Xu et al., 2020; Dominguez et al., 2021; Wu et al., 2021). Typical clinical features of patients with POBINDS include early onset seizures, intellectual disability and developmental delay, although some patients are also characterized by ASD, attention deficit hyperactivity disorder, language impairment and facial dysmorphisms (Poirier et al., 2017; Sakaguchi et al., 2017; Fernández-Marmiesse et al., 2019; Li et al., 2019; Nakashima et al., 2019; Ernst et al., 2021; Rahman and Fatema 2021; Selvam et al., 2021; Valentino et al., 2021; Yang et al., 2021).

**FIGURE 1:**
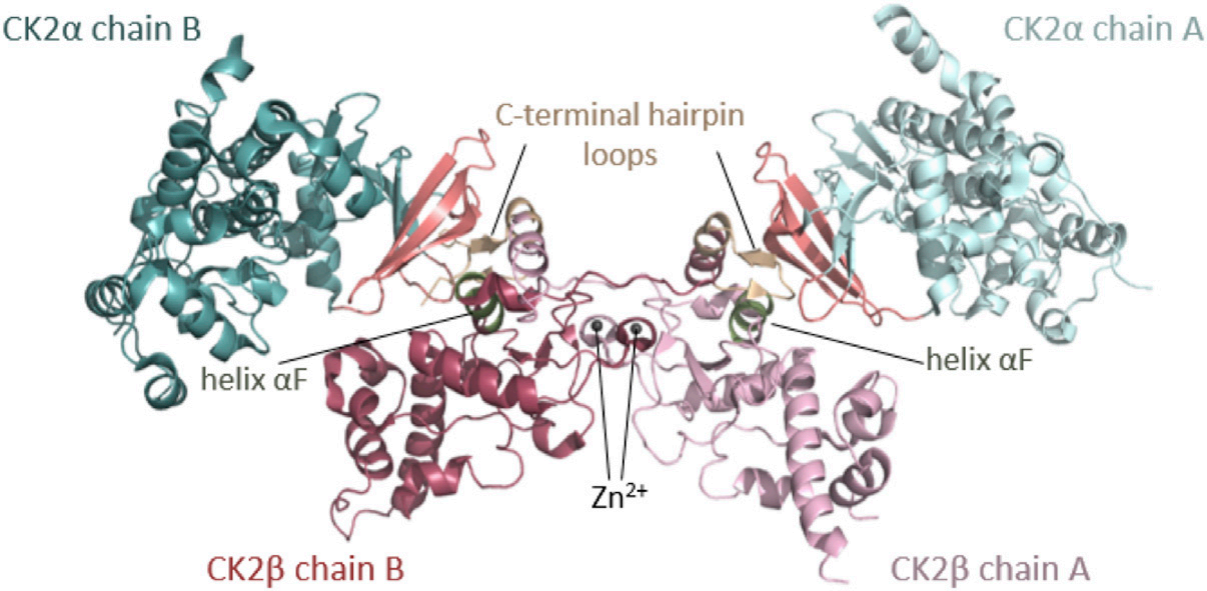
Architecture of the heterotetrameric CK2 holoenzyme. CK2α has two lobes: a N-terminal domain based on a central 5-stranded β-sheet and an α-helical C-terminal domain. CK2β has two domains: an α-helical domain and a zinc finger domain that serves as a dimerization interface. CK2β forms dimers via the zinc finger, hydrophobic core residues, and the crossover of the C-terminal tails over the other CK2β subunit. Due to the crossover of the C-terminal tails of the CK2β dimer, both CK2β chains are involved in the interaction with both CK2α chains. The contact of the CK2β dimer to the two spatially separated CK2α subunits is mediated *via* the C-terminal hairpin loop of one CK2β chain (α/β tail contact, coloured wheat) and the area around helix αF of the other CK2β chain (α/β body contact, coloured dark green). The subunit interaction interface of CK2α is located at the outer surface of the β-sheet of the N-terminal lobe (coloured salmon) (Niefind et al., 2001). The Figure was drawn with PyMOL (Schrödinger, 2013) using the CK2 holoenzyme structure (PDB_ID: 4DGL (Lolli et al., 2012)). Detailed figures of functional domains in the structural section.

*CSNK2A1* and *CSNK2B* genes encode CK2⍺, a highly conserved serine/threonine protein kinase, and CK2β, a regulatory protein, respectively. CK2α is present in cells in monomeric form and in a heterotetrameric form composed of two CK2α subunits bound to a dimer of two CK2β subunits (CK2β) (Figure 1). Monomeric CK2α kinase and CK2α holoenzyme phosphorylate common and distinct substrates, as docking to CK2β is required for some substrates to be phosphorylated (reviewed in (Roffey and Litchfield 2021)). The CK2α-paralog CK2α’ is encoded by the *CSNK2A2* gene and differs mainly in the length and sequence of the C-terminus (Pyerin and Ackermann 2003).

CK2α and CK2β are linked to physiological and cellular functions in unicellular and pluricellular eukaryotes and are indispensable for embryonic development of animal and plants; they are also linked to cancer and neurodegenerative diseases, among others (Litchfield 2003; Meggio and Pinna 2003; Ahmad et al., 2008; Duncan and Litchfield 2008; Guerra and Issinger 2008; Dominguez et al., 2009; Ruzzene and Pinna 2010; Trembley et al., 2010; Macias Alvarez et al., 2013; Chua et al., 2017; Gotz and Montenarh 2017; Borgo et al., 2021). CK2α and CK2α′ mouse knockouts had different phenotypes (reviewed in (Macias Alvarez et al., 2013)). CK2α (Lou et al., 2008) and CK2β (Buchou et al., 2003) knockouts are embryonic lethal while CK2α′ knockout mice are viable but have impaired spermatogenesis (Xu et al., 1999). *In vitro*, the isoenzymes display partial functional redundancy, however there are examples that indicate different functions: the C-terminal phosphorylation sites of CK2α which are phosphorylated in a cell cycle dependent manner are absent in CK2α′ (St-Denis et al., 2009). The affinity of CK2α′ to CK2β is lower than the affinity between CK2α and CK2β (Bischoff et al., 2011a), and isoform specific binding partners have been described (Hériché et al., 1997; Bosc et al., 2000; Litchfield et al., 2001).

CK2α has the kinase-typical bilobal architecture with a N-terminal domain with a central β-sheet and a α-helical C-terminal domain (Figure 1, detailed schemes of functional domains are found in subsequent figures), which harbor common structural and functional elements of eukaryotic protein kinases (EPKs), such as the Gly-rich loop, the hinge region, the catalytic loop, the activation loop and the P+1 loop (all analyzed in this study). CK2α belongs to the CMGC family of EPKs—with cyclin dependent kinases (CDKs), mitogen-activated kinases (MAPKs), glycogen synthase kinase-3 (GSK-3) and cell division control 2 (CDC2)-like kinases (CLKs)—which is characterized by a helical insert after helix αG in the C-terminal lobe—(Manning et al., 2002). Unlike its highly regulated relatives within the CMGC family and the majority of other EPKs, CK2α is constitutively active (Niefind et al., 1998; Niefind et al., 2007; Borgo et al., 2021; Roffey and Litchfield 2021). Typically, EPKs are activated or inactivated in response to an extracellular signal and undergo large conformational changes. For example, often upon activation, the activation loop opens to allow the substrate to bind and the helix αC is re-oriented leading to the formation of a salt bridge between a conserved lysine and glutamine, which is critical for the coordination of the α- and β-phospho-groups of the cosubstrate ATP (Huse and Kuriyan 2002). The structural plasticity of most EPKs that enables the rearrangements of these regulatory key elements is absent in CK2α. On the contrary, the active conformation is fixed by internal structural constraints. Firstly, the N-terminal segment stabilizes the activation loop and helix αC in the canonical active kinase conformation (Niefind et al., 1998). Secondly, the Phe residue in the DFG-motif at the beginning of the activation loop is replaced by a Trp residue in CK2α. This unique DWG motif in CK2α is internally stabilized by an additional hydrogen bond and therefore disfavors the inactive “DFG-out” conformation known from other EPKs (Pargellis et al., 2002). Compared to the other EPKs, CK2α shows different epicentres of plasticity: in particular, the hinge region and the helix αD (Niefind and Issinger 2010) and the glycine-rich loop display a high degree of flexibility (Raaf et al., 2009). A number of publications describe many unconventional mechanisms of regulation for CK2 which have been recently reviewed (Borgo et al., 2021; Roffey and Litchfield 2021).

Unlike most other protein kinases, CK2α can efficiently use both ATP or GTP as cosubstrates. The arrangement of water molecules in the active site is crucial to switch from an ATP- to a GTP-compatible state explaining the dual cosubstrate specificity (Niefind et al., 1999). CK2α has an acidophilic substrate profile with the minimal consensus sequence S/T-D/E-X-D/E (Marchiori et al., 1988). The preference to phosphorylate its substrate at acidic clusters is determined by two anion binding sites (P+3 site and P+1 site) at the activation segment and is strongly interconnected with the enzyme’s constitutive activity. The function of the equivalent anion binding sites in the CMGC kinase family ranges from regulation to substrate recognition (Niefind et al., 2007). For example, during activation in MAP kinases the positive charge of the P+1 pocket is neutralized by a phosphorylated residue accompanied by large conformational rearrangements (Bellon et al., 1999), and in GSK-3 the positive charge is neutralized by an auto-inhibitory phosphorylation in its N-terminus (Cross et al., 1995) or by a primarily phosphorylated substrate (Frame et al., 2001). However, in CK2α, the anion binding sites solely serve substrate recognition and are not involved in regulation (Niefind et al., 2007). An additional determinant of CK2α′s preference for acidic substrate is the polybasic stretch.

Rigidly fixed in its active form, CK2α can only be regulated by more unconventional or subtle mechanisms (Niefind et al., 2007). In this context, the regulatory subunit CK2β comes into play: although it does not serve as an on/off-switch, it modulates CK2α substrate specificity and its stability against denaturation forces (Issinger et al., 1992; Meggio et al., 1992). CK2β has an absolutely conserved zinc finger motif that serves as a dimerization interface (Figures 1, 7F). Due to the zinc finger, hydrophobic core residues, and the crossover of the C-terminal tails, the dimerization surface is highly effective and causes CK2β to form permanent dimers (Chantalat et al., 1999). Integrated in the CK2 holoenzyme, the contact of the CK2β dimer with the two spatially separated CK2α subunits is mediated via the C-terminal hairpin loop of one chain (α/β tail contact) and the area around helix αF of the other chain (α/β body contact). The subunit interaction interface of CK2α is located at the outer surface of the β-sheet of N-terminal lobe (Niefind et al., 2001).

We are beginning to gather information about the genetic basis of OCNDS and POBINDS. To date, thirty six *CSNK2A1* variants (mostly *de novo*) are published in both male and female individuals with OCNDS (Iossifov et al., 2014; Okur et al., 2016; Trinh et al., 2017; Yuen et al., 2017; Akahira-Azuma et al., 2018; Chiu et al., 2018; Owen et al., 2018; Duan et al., 2019; Nakashima et al., 2019; Martinez-Monseny et al., 2020; van der Werf et al., 2020; Wang et al., 2020; Wu W et al., 2020; Xu et al., 2020; Dominguez et al., 2021; Wu et al., 2021). Most published gene variants are missense, and the rest are nonsense, splice and frameshift variants. Forty *CSNK2B* variants (*de novo*) are published in both male and female individuals with POBINDS (Poirier et al., 2017; Sakaguchi et al., 2017; Fernández-Marmiesse et al., 2019; Li et al., 2019; Nakashima et al., 2019; Ernst et al., 2021; Rahman and Fatema 2021; Selvam et al., 2021; Valentino et al., 2021; Yang et al., 2021). A small subset of the identified *CSNK2B* variants are missense, and the rest are nonsense, splice and frameshift variants. In addition to the published data, *CSNK2A1* and *CSNK2B* variants have also been deposited in data repositories, although these data have not been analyzed so far.

While NGS-based methods have improved clinical diagnosis of rare genetic diseases and accelerated the discovery rate of causative genes, there is a growing gap between the identification of rare-disease-causing genes and the medical and scientific knowledge leading to the formulation of effective therapies (Boycott et al., 2013). For CK2α and CK2β, published studies and databases have used programs to predict whether mutants are pathogenic, likely pathogenic or variants of unknown significance (VUS); sometimes these predictions have led to conflicting results. We are starting to learn the biochemical effects of the mutations in CK2α and CK2β. There is experimental evidence that the CK2α and CK2β mutations associated with OCNDS and POBINDS can cause biochemical changes in the proteins. Eighteen CK2α missense mutants have decreased kinase activity *in vitro* kinase assays and some have low expression levels, when recombinant and cell line-expressed mutant CK2α are analyzed (Dominguez et al., 2021). In addition, some of the CK2α mutant proteins show changes in subcellular localization compared to the wild-type protein (Dominguez et al., 2021). There is also experimental evidence that one CK2β mutant has low expression levels and another does not interact with CK2α (Nakashima et al., 2019). Recently, altered substrate specificity has been reported for CK2α variant K198R (Caefer et al., 2022; Werner et al., 2022). However, we still have no knowledge of the signaling, cellular and biological mechanism of action of the mutations in CK2α and CK2β that are associated with OCNDS and POBINDS, respectively.

The aims of this study are threefold: 1) provide an integrated list of all identified *CSNK2A1* and *CSNK2B* variants to date by leveraging multiple resources–scientific articles and data repositories–to have a broader understanding of the mutation hotspots and the protein domains affected; 2) analyze all mutations collected using the same diverse *in silico* functional predictions tools and assess their concordance with experimental data, and 3) hypothesize on the potential effects of the mutations on protein structure and function.

## Materials and methods

### *CSNK2A1* and *CSNK2B* variants

*CSNK2A1* and *CSNK2B* variants were compiled from the OCNDS literature (Iossifov et al., 2014; Okur et al., 2016; Trinh et al., 2017; Yuen et al., 2017; Akahira-Azuma et al., 2018; Chiu et al., 2018; Owen et al., 2018; Duan et al., 2019; Nakashima et al., 2019; Martinez-Monseny et al., 2020; van der Werf et al., 2020; Wang et al., 2020; Wu R et al., 2020; Xu et al., 2020; Dominguez et al., 2021; Wu et al., 2021), and the POBINDS literature (Poirier et al., 2017; Sakaguchi et al., 2017; Fernández-Marmiesse et al., 2019; Li et al., 2019; Nakashima et al., 2019; Ernst et al., 2021; Rahman and Fatema 2021; Selvam et al., 2021; Valentino et al., 2021; Yang et al., 2021). We also compiled *CSNK2A1* and *CSNK2B* variants from the DECIPHER database v11.6 (https://www.deciphergenomics.org; last accessed 1/5/22) (Firth et al., 2009), Simons Searchlight Single Gene Dataset v8.0, AutDB Autism Informatics Portal (http://autism.mindspec.org/autDB/; last accessed 1/5/22) (Basu et al., 2009), and website/url to ClinVar, “(https://www.ncbi.nlm.nih.gov/clinvar/) (last accessed 1/5/22) (Landrum et al., 2018).

To review the literature, we used the queries: OCNDS, POBINDS, CSNK2A1, CSNK2B. These queries identified papers focused on the two disorders, large-scale sequencing efforts, and other articles that did not contain OCNDS and POBINDS variants. In addition, we used the AutDB Autism Informatics Portal to find additional research articles that contain variants in *CSNK2A1* and *CSNK2B*. For the gene variants described in the literature, we recorded each patients’ variant on *CSNK2A1* or *CSNK2B*. For the ClinVar database, we compiled the gene variants described as OCNDS, POBINDS and also variants described as “neurodevelopmental syndrome/inborn genetic disease,” provided that the phenotypic description closely-related to OCNDS or POBINDS. Some of the patients in the DECIPHER database were published in research articles. These patients were identified by comparing clinical manifestations and/or sequencing facilities, to ensure that each patient data was only counted once.

We applied for and obtained the Simons Searchlight Single Gene Dataset v8.0 which includes *CSNK2A1* gene variants of patients. The collection of gene variants was reviewed and deemed non-human subjects research by the Institutional Review Board (IRB) of Boston University Medical Campus and Boston Medical Center (IRB # H-41033). To avoid duplicates, we selected variants from the Simons database not already included in ClinVar, as it was highly possible that the variants were already submitted to ClinVar by the original sequencing teams. These variant data were combined and presented in Tables.

### CK2α and CK2β sequences, protein domains and alignment

CK2α and CK2β gene and protein data were obtained from UniProt (Accession P68400; Accession P67870). Protein domains were determined based on the most consequent domain definition (according to the CATH database) given in (Niefind and Battistutta 2013), the crystal structure of protein kinase CK2α from *Zea mays* at 2.1 A resolution (Niefind et al., 1998), and the crystal structure of CK2β (Chantalat et al., 1999). For schemes and histograms, we utilized similar color palettes to facilitate reading and understanding of the data.

We utilized R (v4.0.3) and the Ggplot package (v3.3.5) to plot the number of unique patients per residue in the primary protein sequences, including data for missense, nonsense, and frameshift mutations (excluding splice-site variants). To determine the average number of point mutations per residue in each domain we calculated the number of unique patients per domain (End Residue − Start Residue + 1), and divided the total number of unique patients in each domain by the domain length in Microsoft Excel (v16.16.27).

To determine the probability of finding the number of patients with a particular mutation(s) in each residue, we calculated an exact chi-square test statistic comparing the observed mutation probabilities to those expected by chance and present the corresponding *p*-value. For this, we assumed that each of the 9 variants per codon are equally likely to occur, thus the counts across the 9 mutations will follow a multinomial distribution with the probability of observing each variant = 1/9 (=0.11).

The nonrandom mutation clustering (NMC) algorithm was utilized to identify nonrandom clusters of mutated residues on each protein (Ye et al., 2010). This probability model assumes that mutations along a protein follow a uniform distribution, that mutations are independent of each other in and within samples, and that the number of samples exceeds the number of mutations. NMC utilizes the differences between pair-wise order statistics to derive probabilities. In the original study, NMC was performed on missense mutations to identify activating mutations in cancers that could be targeted for pharmacological intervention. We have expanded the statistical method to identify residues that are found mutated non-randomly among the point mutations that we collected. We utilized all mutations, except splice, as our study differs from cancer studies where missense mutations are key for disease development and progression. All the mutation analyzed in CK2α and CK2β in this study appear in the patient populations that we are studying and therefore, all may have a role in disease (note that it has not been yet demonstrated that any of these mutations causes disease *in vivo*). For each analysis we obtained clusters ranging from 1 residue to almost full-length protein. Out of these data, significant (*p* < 0.05) clusters of only one residue were depicted in a table. R code used to generate histograms and NMC analysis is provided in R markdown upon request.

To determine the conservation of the changed residues across eukaryotic species through evolutionary alignment, the primary sequences of CK2α and CK2β from diverse eukaryotic organisms were downloaded. We utilized the species selected in Homologene, and utilized their curated sequences, except for *Xenopus laevis* and *Danio rerio*, for which we found sequences with high similarity to the human sequence using BLASTP. Multiple sequence alignment of the sequences utilized was built with MUSCLE (https://www.ebi.ac.uk/) (Edgar 2004) and displayed in HTML format. In this format, conserved residues appeared in blue (conservation across most species), gray (conservation across some species) or white (no conservation).

### Prediction tools

*In silico* tools were used to obtain predictions of the impacts of CK2α and CK2β missense mutations based on their evolutionary conservation (PANTHER, MutationTaster2), functional impact (SIFT, PROVEAN, Polyphen-2, I-Mutant 3.0 Disease, MutationAssessor, SNAP2), and importance in protein stability (PremPS, Kinact, I-Mutant ΔΔG). Two consensus programs (PredictSNP, REVEL) were also used for functional predictions. Our classification of these programs into categories of evolutionary conservation, functional impact, and effect on protein stability are not strict classifications, as many of these programs generate their predictions based on many of these factors. Rather, these classifications are used to more easily compare programs that consider similar protein characteristics when evaluating mutations. The following steps (exemplified for *CSNK2A1*) were followed to obtain predictions from each of these prediction programs:

1) PANTHER is a protein classification system that predicts the functional consequence of a single-nucleotide polymorphism (SNP) based on the preservation time of the mutated amino acid (Tang and Thomas 2016). Click on the cSNP Scoring tab. Input the FASTA sequence for *CSNK2A1* and a list of all missense substitutions. Select “*Homo sapiens*” for the organism and submit. Prediction outputs are “Benign,” “Possibly Damaging,” or “Probably Damaging.”
2) MutationTaster2 uses a Bayes classifier model to predict whether a mutation is disease-causing and examines conservation of amino acids across vertebrate and invertebrate species (Schwarz et al., 2014). Enter the gene name “*CSNK2A1*.” Select transcript “ENST00000217244 (*protein_coding*, 4416 bases) NM_177559.” Enter Position/snippet, which refers to “coding sequence ORF” nucleotide position. Enter the mutated base. Prediction outputs are “Polymorphism” or “Disease Causing.”
3) SIFT predicts the effect of missense mutations on protein function by assessing the evolutionary conservation as well as the physical characteristics of the wild type and variant amino acids (Sim et al., 2012). Input the FASTA sequence for *CSNK2A1* and the list of all missense substitutions. Keep default parameters (search in UniProt-SwissProt 2010_09, Median conservation of sequences: 3.00, Remove sequences more than 90 percent identical to query) and submit. Use the SIFT Amino Acid Predictions Tables to obtain predictions and the Scales Probabilities for Entire Protein Table for prediction scores. Prediction outputs are “Tolerated” or “Not Tolerated,” and scores range from 0 to 1, with a damaging score <= 0.05 and a tolerated score >0.05.
4) Polyphen-2 predicts damaging missense mutations based on sequence and structure changes that could result from them (Adzhubei et al., 2010). Enter gene name or FASTA sequence. Enter the residue position of the missense mutation. Click the reference amino acid and mutant amino acid, and submit. Prediction outputs are “Benign” if score is <0.5, “Possibly Damaging” if score is 0.5–0.95, and “Probably Damaging” if score is >0.95, with scores ranging from 0 to 1.
5) PROVEAN assesses the effects of amino acid substitutions, insertions, and deletions on protein function by utilizing sequence homology and comparing with variants that have known functional consequences (Choi et al., 2012). Enter FASTA sequence. Enter the list of missense mutations and submit. Prediction outcomes are “Deleterious” if score is <−2.5 or “Neutral” if score is >−2.5.
6) I-Mutant 3.0 Disease predicts whether a single-site mutation is disease-causing based on the change in protein sequence (Capriotti et al., 2005). Select “Protein Sequence” under “Prediction of Disease associated single point mutation from.” Enter protein sequence. Enter the residue position of mutation and the new residue. Select “sequence-based” prediction and submit. Prediction outcomes are “Neutral” or “Disease Causing.”
7) MutationAssessor provides a functional impact score for missense mutations based on sequence homology (Reva et al., 2011). Input the missense mutations with “CSK21_HUMAN or CSK2B_HUMAN” before each mutation and submit. Prediction outcomes are “Neutral” if Functional Impact (FI) score is 0.8, “Low” if FI score is between 0.8 and 1.9, “Medium” if FI score is between 1.9 and 3.5, or “High” if FI score is >3.5.
8) SNAP2 predicts whether a missense mutation will have a functional effect based on sequence homology and possible alterations to protein structure (Hecht et al., 2015). Enter FASTA sequence for protein of interest and click “Run Prediction.” This will produce a chart with all of the possible variants for each amino acid of the protein. Search for residue position of interest and locate the prediction for each missense mutation in the chart. Prediction outputs are “Negative/Neutral” or “Positive/Effect”
9) PremPS computes the change in Gibbs free energy produced by a variant, using a 3D structure model of the protein, to predict its impact on protein stability (Chen et al., 2020). Enter PDB code and upload PDB file (we used 2PVR for CK2α and 3EED for CK2β) and click next. Select protein chains (Chain A) and click next. Select “Chain A” for “Chain to Mutate,” select residue to be mutated, and select the mutant residue. Click submit. Prediction outputs are “Negative/Stabilizing” or “Positive/Destabilizing.”
10) Kinact specifically assesses the impact of missense mutations on the activity of kinases by using structure and sequence characteristics to predict changes in protein stability (Rodrigues et al., 2018). Upload CK2α PDB file (2PVR). Enter the missense mutations and specify the chain (Chain A). Enter the FASTA sequence and submit. Prediction outputs are “Positive/Stabilizing” or “Negative/Destabilizing.”
11) I-Mutant ΔΔG predicts the effect of a single-site mutation on protein stability by calculating the difference between the unfolding Gibbs free energy of wild type and mutant protein structures (Capriotti et al., 2005). There are two options for generating an output: 1) Enter protein sequence, residue position of mutation, and the new residue. Enter temperature (37°C) and pH (7.4). Select “ΔΔG Value and Binary Classification” and submit. 2) Select “protein structure” under “Prediction of protein stability changes upon single point mutation from”. Upload the PDB file for the protein of interest (2PVR for CK2α and 3EED for CK2β). Enter the residue position of mutation and the new residue. Enter temperature (37°C) and pH (7.4). Select “ΔΔG Value and Binary Classification” and submit. Prediction outputs are “Positive/Stabilizing” or “Negative/Destabilizing.”
12) PredictSNP is a consensus program that compiles predictions from MAPP, PhD-SNP, Polyphen-1, Polyphen-2, SIFT, SNAP, nsSNPAnalyzer, and PANTHER to predict which missense mutations may be related to disease (Bendl et al., 2014). Load the FASTA sequence. Select positions of interest and the corresponding mutant residues. Select tools for evaluation (PredictSNP, MAPP, PhD-SNP, Polyphen-1, Polyphen-2, SIFT, SNAP), and click evaluate. Prediction outputs are “Neutral” or “Deleterious” along with a percentage indicating the confidence of the prediction.
13) REVEL is a consensus program that compiles predictions from MutPred, FATHMM v2.3, VEST 3.0, PolyPhen-2, SIFT, PROVEAN, MutationAssessor, MutationTaster, LRT, GERP++, SiPhy, phyloP, and phastCons to generate an overall prediction of pathogenicity for missense mutations (Ioannidis et al., 2016). Download the REVEL spreadsheet which corresponds to the genomic position of *CSNK2A1* (clicking on the link for gene segment of interest will automatically download the REVEL spreadsheet for that segment). Search for the GRCh38 position of each variant to find corresponding predictions. Prediction outputs are “Non-diseasing causing” if score is <0.5 or “Disease causing” if score is >0.5.

The McNemar’s test was used to compare the categorical outputs of the functional programs (SIFT, Polyhphen-2, PROVEAN, Mutation Assessor, SNAP2, I-Mutant3.0 Disease, REVEL, and PredictSNP) and the stability programs (PremPS, Kinact mCSM, Kinact SDM, Kinact DUET, and I-Mutant3.0 ΔΔG). The Kinact suite of tools is only available for kinases such as CK2α therefore for CK2β we only compared PremPS and I-Mutant3.0 ΔΔG. We calculated two values to compare each pair of tests that were performed on the mutations. First, the Kappa Coefficient (which calculates the degree of agreement beyond what would be expected by chance.) Second, the *p*-value from McNemar’s test, an assessment of whether there is a significant difference between tests in the ratings or “benign” vs. “effect”. Third, the *p*-value from McNemar’s test, an assessment of whether there is a significant difference between tests in the ratings or “benign” vs*.* “effect.”

For prediction of changes in protein-protein binding affinity on mutations we utilized BeAtMuSiC (http://babylone.ulb.ac.be/beatmusic). BeatMusic predicts changes in binding free energy (ΔΔG) induced by point mutations (Dehouck et al., 2013) based on known protein structures, the strength of interactions at the interface and the overall stability of the complex. As input for this prediction we used the CK2 holoenzyme structure (PDB ID: 4DGL; (Lolli et al., 2012)).

### Structural analysis

The CK2α^1-335^ structure (PDB ID: 2PVR (Niefind et al., 2007)) or CK2β^1-193^ structure (PDB ID: 3EED (Raaf et al., 2008)) were used as reference structures. The protein structures were downloaded from the RCSB Protein Data Bank (Berman et al., 2000). To visualize the conserved residues of CK2α and CK2β the ConSurf server (Glaser et al., 2003; Landau et al., 2005; Ashkenazy et al., 2010; Celniker et al., 2013; Ashkenazy et al., 2016) was used. Multiple Sequence Alignments were built using MAFFT (Katoh et al., 2018). The Homologues were collected from UNIREF90 using the homolog search algorithm HMMER (Eddy 2011) (E-value: 0.0001; No. of HMMER Iterations: 1). As maximal identity between sequences 95% and as minimal identity for homologs 40% were chosen. The calculation was performed on a sample of 150 sequences that represent the list of homologues to the query (Supplementary Table S3). Conservation scores were calculated using the Bayesian method (Mayrose et al., 2004). For structural analysis the programs COOT (Emsley et al., 2010) and PyMOL were used. Figures 1, 4A,C, 6, 7 were drawn using PyMOL (Schrödinger 2013).

## Results and discussion

### Update on *CSNK2A1* and *CSNK2B* variants

The first publications for OCNDS and POBINDS associated these syndromes with variants in *CSNK2A1* and *CSNK2B*, respectively, and provided clinical manifestations that defined the syndromes. Since then, other variants in these two genes have been published and classified as OCNDS and POBINDS. In addition, a number of unpublished *CSNK2A1* and *CSNK2B* variants have been placed in data repositories which include clinical manifestations similar to the published variants. In view of the spread of the data, we collected all of the *CSNK2A1* and *CSNK2B* variants from publications and data repositories. Chromosomal variants were not collected in this study. The results of this data collection are found in Tables 1, 2, where the data is divided into non-conservative amino acid mutations and splice mutations. Table 1 includes the 36 published *CSNK2A1* variants (some of which were also in data repositories), and 32 unique variants from data repositories (Simons, DECIPHER, ClinVar, AutDB). Table 2 includes 40 published *CSNK2B* variants (some of which were also in data repositories) and 27 unique variants from data repositories. By pooling these diverse variant data resources, we have increased the number of variants analyzed by 89% in CK2α and by 68% in CK2β. Interestingly, no genetic diseases are known so far to be associated with mutants of *CSNK2A2*, a *CSNK2A1* paralog, located on a different chromosome (Yang-Feng et al., 1991).

**TABLE 1:** CK2α mutants associated with OCNDS phenotypes. Tables include nucleotide mutation, amino acid residue change (when applicable), number of patients per mutation and source. (A) includes variants that affect amino acid residues and (B) includes splice variants. Total number of patients for CK2α was 129. For samples found duplicated in different sets, we included all sources separated with a “/”.

Position	Nucleotide change	Mutation	Ref	Residue	Mut	Number of patients	Source
1	c.1A>G	p.Met1?	M	1	?	1	Chiu et al., 2018/Wang et al., 2020/ClinVar (1)
21	c.62G>A	p.Arg21Gln	R	21	Q	1	ClinVar (1)
27	c.79G>A	p.Glu27Lys	E	27	K	2	Lelieveld et al., 2017/Chiu et al., 2018 (1);
							(1)
32	c.96delA	p.Glu32Aspfs*14	E	32	Dfs*14	1	ClinVar (1)
36	c.106C>T	p.Gln36*	Q	36	*	1	DECIPHER (1)
39	c.116A>G	p.Tyr39Cys	Y	39	C	1	Wang et al., 2020 (1)
39	c.116A>C	p.Tyr39Ser	Y	39	S	1	DECIPHER (1)
39	c.117C>A	p.Tyr39Ter	Y	39	*	1	ClinVar (1)
46	c.137G>T	p.Gly46Val	G	46	V	1	ClinVar (1)
47	c.139C>G	p.Arg47Gly	R	47	G	2	ClinVar (1); SFARI (1)
47	c.140G>A	p.Arg47Gln	R	47	Q	8	Okur et al., 2016 (1); Chiu et al., 2018 (1)
							Owen et al., 2018/DECIPHER (1); DECIPHER (1); ClinVar (4)
50	c.149A>G	p.Tyr50Cys	Y	50	C	9	Martinez-Monseny et al., 2020 (1); Wu R et al., 2020 (1); ClinVar (5); DECIPHER (2)
50	c.149A>T	p.Tyr50Phe	Y	50	F	1	Dominguez et al., 2021 (1)
50	c.149A>C	p.Tyr50Ser	Y	50	S	1	Okur et al., 2016 (1)
51	c.152G>A	p.Ser51Asn	S	51	N	1	Owen et al., 2018/DECIPHER (1)
51	c.151A>C	p.Ser51Arg	S	51	R	2	Chiu et al., 2018 (1); ClinVar (1)
	c.153T>A						
51	c.152G>T	p.Ser51Ile	S	51	I	1	Wang et al., 2020 (1)
52	c.154G>A	p.Glu52Lys	E	52	K	1	ClinVar (1)
53	157G>C	p.Val53Leu	V	53	L	1	SFARI (1)
73	c.218T>A	p.Val73Glu	V	73	E	1	Chiu et al., 2018 (1)
80	c.238C>T	p.Arg80Cys	R	80	C	1	Wang et al., 2020/Wu et al., 2021 (1)
80	c.239G>A	p.Arg80His	R	80	H	5	Owen et al., 2018/DECIPHER (1); ClinVar (4)
107	c.319C>T	p.Arg107*	R	107	*	2	Wang et al., 2020 (1); ClinVar (1)
126	c.376delC	p.Gln126Argfs*2	Q	126	Rfs*2	1	ClinVar (1)
127	c.380C>T	p.Thr127Met	T	127	M	1	Wang et al., 2020 (1)
128	c.383T>A	p.Leu128*	L	128	*	1	ClinVar (1)
147	c.440G>A	p.Cys147Tyr	C	147	Y	1	Wang et al., 2020 (1)
153	c.458T>G	p.Met153Arg	M	153	R	1	ClinVar (1)
156	c.466G>C	p.Asp156His	D	156	H	1	Trinh et al., 2017/ClinVar (1)
156	c.468T>A	p.Asp156Glu	D	156	E	2	ClinVar (2)
156	c.466G>T	p.Asp156Tyr	D	156	Y	1	ClinVar/SFARI (1)
158	c.472A>G	p.Lys158Glu	K	158	E	1	ClinVar (1)
158	473A>G	p.Lys158Arg	K	158	R	1	ClinVar (1)
160	c.479A>G	p.His160Arg	H	160	R	4	van der Werf et al., 2020 (1); Wu et al., 2021 (1); ClinVar (2)
161	c.482A>G	p.Asn161Ser	N	161	S	1	DECIPHER (1)
161	c.481A>G	p.Asn161Asp	N	161	D	1	ClinVar (1)
174	c.522A>G	p.Ile174Met	I	174	M	1	Owen et al., 2018/DECIPHER (1)
175	c.524A>G	p.Asp175Gly	D	175	G	2	Okur et al., 2016/ClinVar (1); Duan et al., 2019 (1)
175	c.596G>A	p.Asp175Glu	D	175	E	1	ClinVar (1)
177	529G>A	p.Gly177Ser	G	177	S	1	ClinVar (1)
178	c.533T>G	p.Leu178Trp	L	178	W	1	DECIPHER (1)
191	c.572G>A	p.Arg191Gln	R	191	Q	2	Owen et al., 2018/DECIPHER (1); DECIPHER (1)
191	c.571C>T	p.Arg191*	R	191	*	3	Nakashima et al., 2019 (1); Wang et al., 2020 (1); ClinVar (1)
194	581C>T	p.Ser194Phe	S	194	F	1	SFARI (1)
195	c.583C>T	p.Arg195*	R	195	*	3	Wang et al., 2020 (1); ClinVar (2)
197	c.589T>A	p.Phe197Ile	F	197	I	1	Owen et al., 2018/DECIPHER (1)
198	c.593A>G	p.Lys198Arg	K	198	R	24	Iossifov et al., 2014 (1); Okur et al., 2016 (1)
							Akahira-Azuma et al., 2018 (1); Chiu et al., 2018 (1)
							Owen et al., 2018/DECIPHER (4); Nakashima et al., 2019 (1); van der Werf et al., 2020 (1); Xu et al., 2020 (1); ClinVar (9); TGen (3)
199	c.596G>A	p.Gly199Asp	*G*	*199*	*D*	1	ClinVar (1)
210	c.628G>A	p.Asp210Asn	D	210	N	1	Wang et al., 2020 (1)
231	c.692C>G	p.Pro231Arg	P	231	R	1	Lelieveld et al., 2017/Chiu et al., 2018 (1)
261	c.783C>A	p.Tyr261Ter	Y	261	*	1	ClinVar (1)
306	c.916C>T	p.Arg306*	R	306	*	1	Wang et al., 2020 (1)
312	c.935G>A	p.Arg312Gln	R	312	Q	2	Chiu et al., 2018 (1); ClinVar (1)
312	c.934C>T	p.Arg312Trp	R	312	W	3	Owen et al., 2018/DECIPHER (1); ClinVar (2)
325	c.972_973dupCAG	p.Tyr325Serfs*5	Y	325	Sfs*5	1	Wang et al., 2020 (1)
326	c.976_977insG	p.T326Serfs*13	T	326	Sfs*13	2	DECIPHER (2)
333	c.997C>T	p.Arg333*	R	333	*	1	ClinVar (1)
356	c.1066T>A	p.Ser356Thr	S	356	T	1	ClinVar (1)
363	c.1088C>A	p.Pro363His	P	363	H	1	ClinVar (1)
382	c.1145C>T	p.Pro382Leu	P	382	L	2	ClinVar (2)

Position	Nucleotide change	Variant	Number of patients	Source
55	c.165G>A	Splice variant	2	ClinVar (2)
122	c.366_366+3del	Splice variant	1	DECIPHER (1)
122	c.367-1G>A	Splice variant	1	ClinVar (1)
142	c.426 + 1G>T (IVS6+1G>T)	Splice variant	1	ClinVar/SFARI (1)
274	c.824 + 2T>C	Splice variant	1	Okur et al., 2016/ClinVar (1)
324	c.973 + 1G>A	Splice variant	1	Wang et al., 2020 (1)
324	c.973 + 1G>C	Splice variant	1	Wang et al., 2020 (1)

**TABLE 2:** CK2β mutants associated with POBINDS phenotypes. Table includes nucleotide and amino acid residue changes, number of patients per mutation and reference/source. (A) includes variants that affect amino acid residues and (B) includes splice variants. Total number of patients for CK2α was 90. For samples found in different sets, we included all sources separated with a “/”.

Position	Nucleotide change	Mutation	Ref	Residue	Mut	Number of patients	Source
1	c.1A>G	p.Met1?	M	1	?	3	Ernst et al., 2021 (1); ClinVar (2)
1	c.2T>A	p.Met1?	M	1	?	1	Ernst et al., 2021 (1)
1	c.3G>A	p.Met1?	M	1	?	1	Yang et al., 2021 (1)
1	c.2T>G	p.Met1?	M	1	?	1	ClinVar (1)
5	c.13G>T	p.Glu5Ter	E	5	*	1	Li et al., 2019 (1)
9	c.27del	p.Trp9Ter	W	9	*	2	Ernst et al., 2021 (1); ClinVar (1)
20	c.58G>T	p.Glu20Ter	E	20	*	1	Ernst et al., 2021 (1)
21	c.63C>A	p.Phe21Leu	F	21	L	1	DECIPHER (1)
27	c.78_83dup	p.Glu27_Asp28dup	E	27	_D28dup	1	Ernst et al., 2021 (1)
31	c.91C>T	p.Gln31Ter	Q	31	*	1	ClinVar (1)
32	c.94G>A	p.Asp32Asn	D	32	N	6	Ernst et al., 2021 (3); ClinVar (2); DECIPHER (1)
32	c.95A>C	p.Asp32Ala	D	32	A	1	DECIPHER
34	c.101T>C	p.Phe34Ser	F	34	S	1	Ernst et al., 2021 (1)
34	c.101T>G	p.Phe34Cys	F	34	C	1	ClinVar (1)
35	c.105T>A	p.Asn35Lys	N	35	K	1	Ernst et al., 2021 (1)
37	c.108dupT	p.Thr37TyrfsTer5	T	37	Yfs*5	2	Sakaguchi et al., 2017 (1); ClinVar (1)
42	c.124C>T	p.Gln42Ter	Q	42	*	2	Fernández-Marmiesse et al., 2019 (1); Ernst et al., 2021 (1)
47	c.139C>T	p.Arg47Ter	R	47	*	6	Selvam et al., 2021 (1); Ernst et al., 2021 (1); ClinVar (4)
57	c.170del	p.Glu57Glyfs*15	E	57	Gfs*15	1	Valentino et al., 2021 (1)
61	c.181G>T	p.Glu61Ter	E	61	*	1	Ernst et al., 2021/ClinVar (1)
77	c.229G>A	p.Glu77Lys	E	77	K	2	Ernst et al., 2021 (1); ClinVar (1)
77	c.229G>T	p.Glu77Ter	E	77	*	1	ClinVar (1)
80	c.238T>A	p. Tyr80Asn	Y	80	N	1	ClinVar (1)
82	c. 245T>A	P.Leu82Ter	L	82	*	1	ClinVar (1)
86	c.256C>T	p.Arg86Cys	R	86	C	4	Li et al., 2019 (1); Ernst et al., 2021 (1); ClinVar (2)
88	c.264delC	p.Ile88Ilefs*46	I	88	Ifs*46	1	Li et al., 2019 (1)
90	c.268dupA	p.Thr90Asnfs*24	T	90	Nfs*24	1	ClinVar (1)
97	c.291G>A	p.Met97Ile	M	97	I	1	Ernst et al., 2021 (1)
101	c.303C>A c.303C>G	p.Tyr101Ter	Y	101	*	3	Ernst et al., 2021 (2); ClinVar (1)
106	c.316T>G	p.Phe106Val	F	106	V	1	Ernst et al., 2021 (1)
111	c.332G>C	p.Arg111Pro	R	111	P	3	Li et al., 2019 (2); ClinVar (1)
117	c.349C>T	p.Gln117Ter	Q	117	*	1	DECIPHER (1)
132	c.394_404del	p.Met132LeufsTer110	M	132	Lfs*110	1	Ernst et al., 2021 (1)
137	c.409T>C	p.Cys137Arg	C	137	R	1	Ernst et al., 2021 (1)
137	c.409T>G	p.Cys137Gly	C	137	G	1	Li et al., 2019 (1)
137	c.410G>T	p.Cys137Phe	C	137	F	2	Li et al., 2019 (1); Yang et al., 2021 (1)
158	c.472del	p.Tyr158fs	Y	158	fs	1	ClinVar (1)
164	c.491C>G	p.Pro164Arg	P	164	R	1	ClinVar (1)
165	c.494A>G	p.His165Arg	H	165	R	3	Nakashima et al., 2019 (1), Yang et al., 2021 (1), ClinVar (1)
167	c.499delC	Leu167Serfs*60	L	167	Sfs*60	1	Yang et al., 2021 (1)
179	c.533_534insGT	p.Pro179TyrfsTer49	P	179	Yfs*49	1	Nakashima et al., 2019/ClinVar (1)
181	c.542del	p.Asn181ThrfsTer46	N	181	Tfs*46	1	Ernst et al., 2021 (1)
186	c.554_555dupCC	p.Arg186fs	R	186	fs	1	ClinVar (1)
187	c.560T>C	p.Leu187Pro	L	187	P	1	ClinVar (1)
187	c.560T>G	p.Leu187Arg	L	187	R	1	Li et al., 2019 (1)
188	c.564del	p.Tyr188Ter	Y	188	*	1	Rahman and Fatema 2021 (1)
189	c.566G>T	p.Gly189Val	G	189	V	1	ClinVar (1)
207	c.620_621insC	p.Phe207Phefs*39	F	207	Ffs*39	1	Li et al., 2019 (1)

Position	Nucleotide change	Variant	Number of patients	Source
24	c.72 + 1G>A	Splice variant	1	ClinVar (1)
24	c.73-2A>G	Splice variant	1	Ernst et al., 2021/ClinVar (1)
58	c.175 + 2T>G	Splice variant	1	Poirier et al., 2017/ClinVar (1)
97	c.292-2A>C	Splice variant	2	Yang et al., 2021 (1); ClinVar (1)
97	c.292-2A>G	Splice variant	1	ClinVar (1)
122	c.367 + 1G>A	Splice variant	1	ClinVar (1)
122	c.367 + 2T>C	Splice variant	1	Poirier et al., 2017/ClinVar (1)
122	c.367+5del	Splice variant	2	ClinVar (2)
122	c.367 + 6T>C	Splice variant	1	ClinVar (1)
122	c.368-2A>G	Splice variant	1	Li et al., 2019/ClinVar (1)
185	c.557 + 2T>C	Splice variant	1	DECIPHER (1)
185	c.557 + 1G>A	Splice variant	1	Ernst et al., 2021 (1)
186	c.558-2A>G	Splice variant	1	Ernst et al., 2021 (1)
186	c.558-3T>G	Splice variant	1	Yang et al., 2021 (1)

**FIGURE 2:**
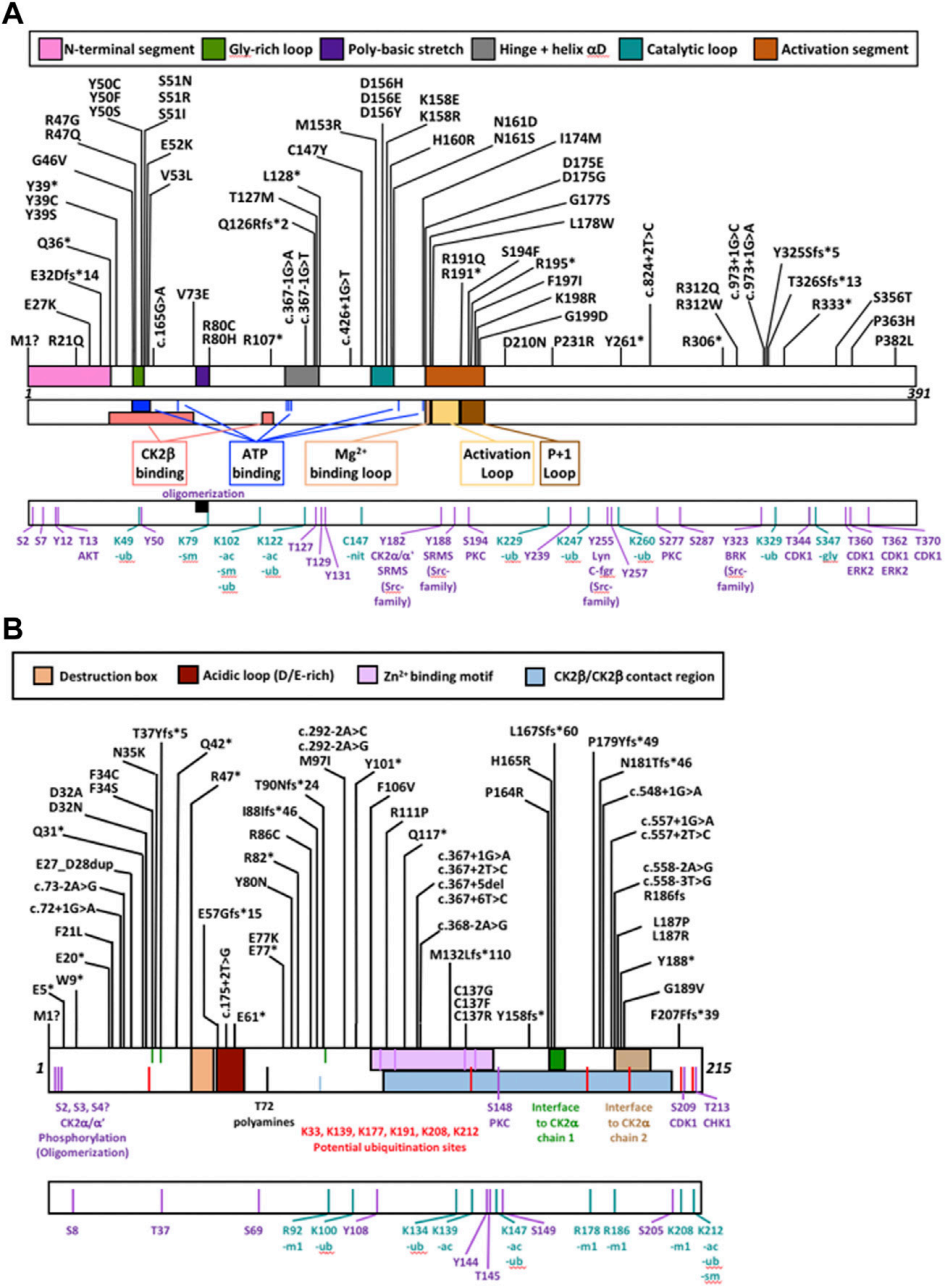
Protein diagram of CK2α and CK2β including the location of mutations to date. CK2α **(A)** and CK2β **(B)** mutants (nonsense, missense, start site variants, frameshift and splice) are represented on a diagram of the primary structure of the proteins. The diagrams include distinct structural and functional domains, and putative functional residues for each protein. For CK2β, the acidic loop domain has also function as pseudosubstrate, and for oligomerization, polyamine binding and polybasic peptide binding. The four Cys (residues 109, 114, 137, 140) key for zinc binding are highlighted in the Zn^2+^ binding motif. Ac, acetylation; gly, glycosylation; m1, monomethylation; nit, nitrosylation; sm, sumorylation.

For *CSNK2A1*, we collected 7 splice variants and 61 single nucleotide variants (SNVs) (Table 1). The SNVs were: 1 start site, 47 missense variants (S51R was coded by 2 different variants), 9 nonsense, and 4 frameshift variants. For *CSNK2B*, we collected 14 splice variants and 49 SNVs: 4 different start site variants, 20 missense, 13 nonsense (Y101* was coded by 2 different variants), 11 frameshift, and 1 codon duplication variant (Table 2). Most of the SNVs in *CSNK2A1* were substitutions leading to missense mutations while in *CSNK2B* there was a high number of duplications and deletions, which resulted in a larger number of nonsense and frameshift mutants compared to *CSNK2A1*. Figure 2 depicts the location of the CK2α and CK2β mutants along their primary protein structures. Indicated in the Figure are distinct structural domains, highly conserved functional domains and putative functional residues from the literature and from PhosphositePlus (Hornbeck et al., 2012) that we will analyze and discuss below.

### CK2α and CK2β mutation frequency and hotspots

**FIGURE 3:**
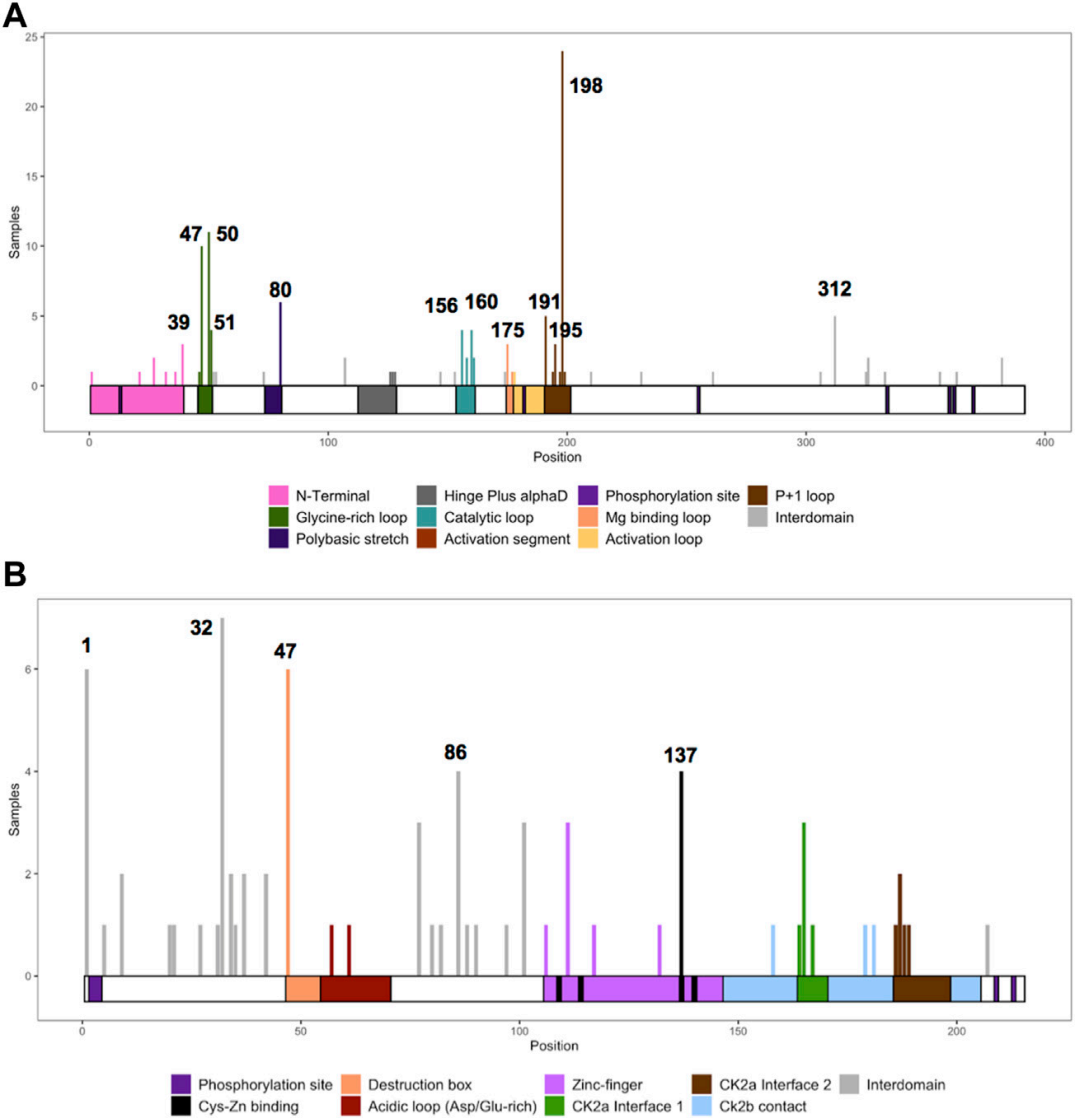
Histogram representing the number of mutations along the primary sequence of CK2α and CK2β. The X-axis represents the primary sequence and the functional domains of CK2α **(A)** and CK2β **(B)**. The Y axis represents the number of patients with missense, nonsense, and frameshift mutations along the primary structure of CK2α and CK2β. Numbers in the histogram represent single residues found significant in the non-random mutation cluster (NMC) analysis.

We collected the number of patients with each variant to identify hotspots (excluding splice sites). First, we represented the total number of unique patients per mutation in each residue (Figure 3). Then, we utilized a non-random mutation clustering (NMC) approach to determine clusters of mutated residues that cannot be explained by random mutation in the nucleotide sequence. We found a number of significant clusters of residues of varying lengths (Supplementary Table S1), of which we selected single-residue clusters (Table 3). For CK2α, these were (in order of significance): K198, Y50, R47, R80, R191, R312, H160, D156, S51, Y39, R195 and D175. For CK2β, these were D32, R47, M1, C137 and R86. Since mutations in these residues were determined to be non-random in the NMC analysis, these residues may be potential mutational hotspots.

**TABLE 3:** Nonrandom mutation clustering (NMC) for CK2α and CK2β mutations. Analysis was performed on the total number of patients with mutations in each amino acid residue (excluding splice site mutations). Table only includes significant clusters with a cluster size of one amino acid residue.

CK2α residue	Number samples	*p*-value	Functional domain or description
39	3	3.12 × 10^−2^	N-terminal segment; CK2b binding
47	10	4.16 × 10^−11^	Gly-rich loop; ATP binding; CK2b binding
50	11	1.28 × 10^−12^	Gly-rich loop; ATP binding; CK2b binding
51	4	3.03 × 10^−3^	Gly-rich loop; ATP binding; CK2b binding
80	6	1.64 × 10^−5^	Polybasic stretch
156	4	3.03 × 10^−3^	Catalytic loop
160	4	3.03 × 10^−3^	Catalytic loop
175	3	3.12 × 10^−2^	Activation segment; Mg^2+^ binding loop
191	5	2.60 × 10^−4^	Activation segment (P+1 loop)
195	3	3.12 × 10^−2^	Activation segment (P+1 loop)
198	24	2.73 × 10^−34^	Activation segment (P+1 loop)
312	5	2.60 × 10^−4^	Interdomain
**CK2β residue**	**Number samples**	***p*-value**	**Functional domain or description**
1	6	3.12 × 10^−4^	N-terminal segment (Truncated protein)
32	7	1.04 × 10^−4^	N-terminal segment (Chantalat’s Conserved surface cluster 1)
47	6	3.02 × 10^−4^	N-terminal Segment (Destruction box; Truncated protein)
86	4	2.08 × 10^−2^	Chantalat’s Conserved surface cluster 1
137	4	2.08 × 10^−2^	Zinc finger (Cys bound to Zinc), CK2α contact region

For some residues, all the patient mutations were the same (e.g., K198R in CK2α, and R47* in CK2β) while for others, there were multiple different mutations in the same residue (Supplementary Table S2). For CK2α, 24% of residues had 2 or more distinct mutations (11 of 45 residues), and 18% for CK2β (7 of 39 residues) (Supplementary Table S2). Therefore, we calculated the probability of finding the number of patients with mutation(s) in each residue. If we assume that each of the 9 variants per codon are equally likely, then the counts across the 9 mutations will follow a multinomial distribution with the probability of observing each variant once in nine (1/9 = 0.11). We calculated an exact chi-square test statistic comparing the observed mutation probabilities to those expected by chance and present the corresponding *p*-value (Supplementary Table S2). For CK2α, significant results were found for R47G/Q, Y50C/F/S, R80C/H, H160R, R191Q/*, R195*, K198R and R312Q/W; and for CK2β for D32A/N, R47*, R86C, R111P, and H165R.

Understanding the genetic mechanisms (population, gene characteristics, etc.) underlying the higher mutability of these sites may lead to important insights that could help us understand the etiology of these diseases.

### Mutation clusters

Studies on neurodevelopmental diseases (NDD) find that missense mutations cluster in or near the functional domains of NDD-associated proteins, in contrast with rare missense mutations found in the 1000 Genomes project (Geisheker et al., 2017). CK2α and CK2β missense mutants were found in both functional domains and interdomain sequences (Figures 2, 3).

We compiled the number of patients in the different functional domains (excluding splice sites) and calculated the ratio of mutations to residues (number of mutations in a protein domain divided by the number of residues in the protein domain) (Table 4; Supplementary Table S1). All functional domains had mutations, albeit at different ratios. None of the Ser and Thr phosphorylated sites in CK2α or CK2β were mutated, but as we will discuss in the structural section, phosphorylation may be affected. For CK2α, the highest ratio was found in the Gly-rich-loop (4.3) followed by the P+1 loop (3.2) while the lowest ratio was found in the activation loop (0.08, with a similar ratio to interdomain sequences). For CK2β, the highest ratios were in the Cys bound to Zinc (1), putative destruction box (0.75) and interface one to CK2α (0.71) while the lowest was in the acidic loop (0.13). Intriguingly, the ratio of the N-terminal segment was among the four highest, suggesting that this region contains functional domains. The interdomain sequences in CK2β showed a higher ratio (0.17) than one known functional domain, suggesting that there may be unidentified functional domains in CK2β located in these sequences. Both these hypotheses are supported by the fact that most of the clusters of one residue in the NMC analysis for CK2β fall in known functional domains, except for D32 and R47 in the N-terminal segment, and R86 in interdomain sequences.

**TABLE 4:** Number of mutations per CK2α and CK2β functional and structural domain. Table summarizes the numbers of CK2α **(A)** and CK2β **(B)** mutants in each functional domain and the # mutations/# number of residues (excluding splice sites).

Domain	Residues	Total residues	Number of patients	#Patients/#residues
N-terminal segment	1–39	39	9	0.23
Gly-rich loop	46–51	6	26	4.33
Polybasic stretch	74–80	7	6	0.86
Hinge plus αD	113–128	16	3	0.19
Catalytic loop	154–161	8	12	1.5
Activation segment	175–201	27	40	1.48
CK2β binding	36–73, 103–108	44	35	0.8
ATP binding	46–51, 66, 114, 115, 116, 163, 174	12	27	2.25
Mg^2+^ binding loop	175–177	3	4	1.33
Activation loop	178–190	13	1	0.08
P+1 loop	191–201	11	35	3.18
Phosphorylation sites	13, 182, 255, T344, T360, S362, S370	7	0	0.00
Interdomain	—	247	19	0.08
N-terminal segment	1–54	54	33	0.61
Phosphorylation sites	2,3,4, S209, T213	5	0	0.00
Destruction box	47–54	8	6	0.75
Acidic loop (Asp/Glu-rich)	55–70	16	2	0.13
Zinc-finger	106–146	41	10	0.24
Cys bound to Zinc	109, 114, 137, 140	4	4	1.00
CK2β/CK2β contact region	110–205	96	22	0.23
Interface to CK2α 1	164–170	7	5	0.71
Interface to CK2α 2	186–198	13	5	0.38
C-terminal segment	179–215	37	8	0.22
Interdomain	39–45 & 71–105	86	15	0.17

Therefore, as the regulation of CK2α and CK2β is still not fully understood, we examined the NMC data to identify short clusters of mutations outside the known functional domains that could be novel functional domains (Supplementary Table S1). For CK2α, we found clusters including the known functional domains (or shorter versions), except for the hinge region that appears in clusters including the activation segment. Residues in the N-terminal segment were found at minimum in clusters that included at least the Gly-rich loop (e.g.: cluster 21-53), suggesting a functional interaction between the N-terminal and the Gly-rich loop. We also found clusters from residue 107 until the second CK2β binding domain, and from residue 147 to the catalytic loop, the activation segment and residues 312/326. These residues (107, 147 and 312/326) are outside of the known functional domains. This suggests that these residues have functional interactions with known functional domains and/or form part of novel functional domains, particularly the Ct residues that are essential for CK2α’s tertiary structure. This is supported by the fact that most of the clusters of one residue in the NMC analysis fall in known functional domains, except for R312 in CK2α (discussed in our structural analysis).

For CK2β, the significance found in the NMC analysis was lower than in CK2α, most likely due to the limited sample number. We did not find short clusters that included the residues of the known functional domains. A number of clusters started at residues 1, 20, 21 and 27 of different lengths (highest significance ending in 32–47). We found significant short clusters around residue 32 (e.g., 31–35), and clusters including residues 47 (34/42-47) and 86 (86–88). These data suggest that these may be one to three novel functional domains, which will be further discussed in our structural analysis.

Overall, these data suggest that protein function will be affected at least by some of these mutations. We will need experimental data to show that the identified potential domains in CK2α and CK2β are *bona fide* functional domains. If so, NMC analysis could be used to identify novel potential functional domains for NDD-associated proteins. Our next analyses will assess, via diverse methods, the potential impact of these mutations in protein structure and function, and will indicate potential biochemical mechanisms.

### Evolutionary conservation

**FIGURE 4:**
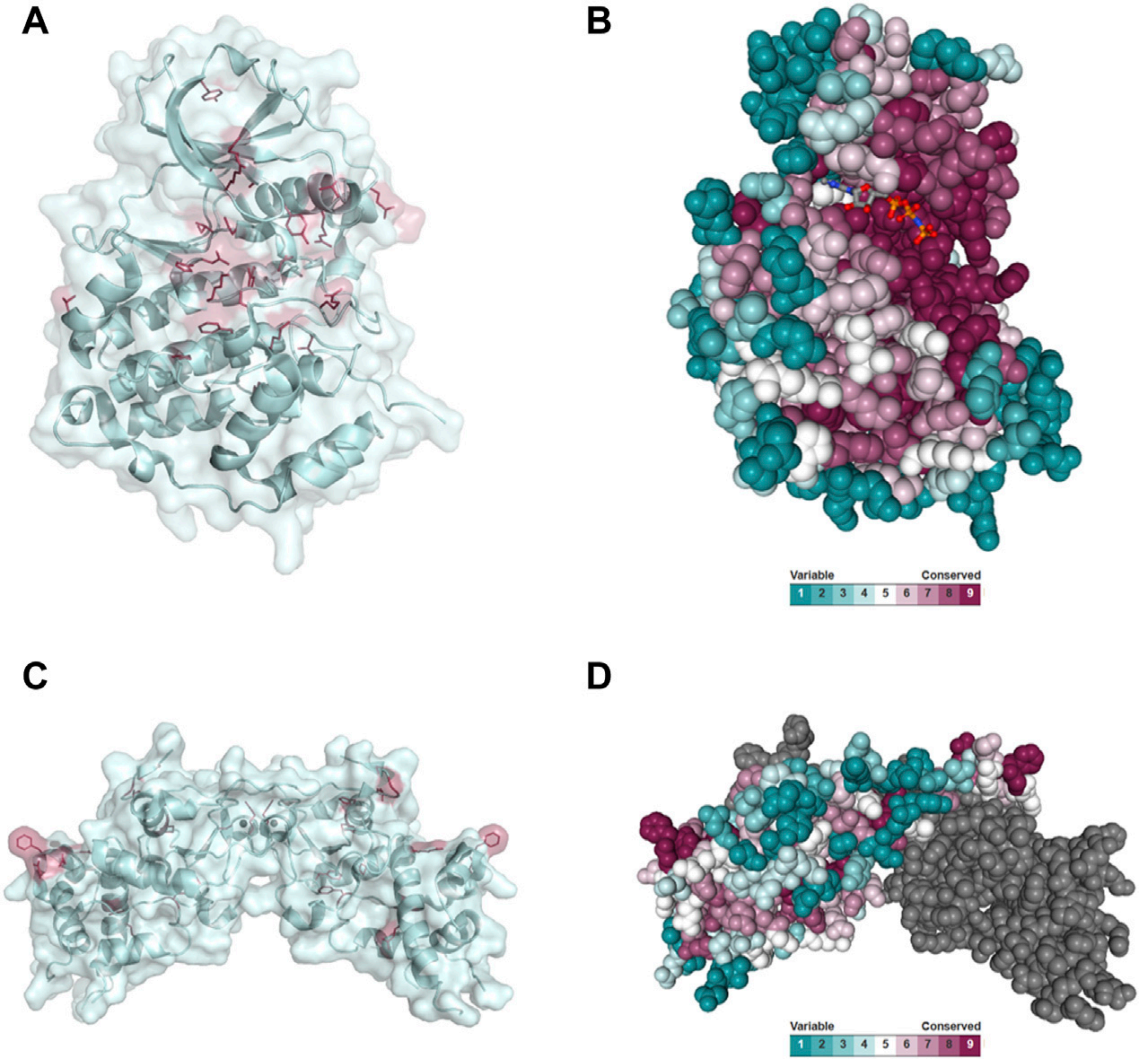
Conserved residues in CK2α and CK2β across 150 species. **(A)** General clustering of the mutation sites in CK2α including surface representation. The structure of the CK2α^1-335^ monomer is shown as a pale cyan cartoon representation, the OCNDS related mutation sites are shown as sticks and are coloured raspberry red. The CK2α^1-335^ structure (PDB_ID: 2PVR (Niefind et al., 2007)) was used for the figure. The figure was drawn using PyMOL (Schrödinger 2013). **(B)** Conserved residues of CK2α. Conserved residues of CK2α in spacefill representation. The figure was created using the ConSurf server (Glaser et al., 2003; Landau et al., 2005; Ashkenazy et al., 2010; Celniker et al., 2013; Ashkenazy et al., 2016) with the CK2α^1-335^ structure (PDB_ID: 2PVR (Niefind et al., 2007)). Multiple Sequence Alignment was built using MAFFT (Katoh et al., 2018). The Homologues were collected from UNIREF90 using the homolog search algorithm HMMER (Eddy 2011) (E-value: 0.0001; No. of HMMER Iterations: 1). As maximal identity between sequences 95% and as minimal identity for homologs 40% were chosen. The calculation was performed on a sample of 150 sequences (see Supplementary Data Sheet S3) that represent the list of homologues to the query. Conservation scores were calculated using the Bayesian method (Mayrose et al., 2004). **(C)** General clustering of the mutation sites in CK2β including surface representation. The structure of the CK2β^1-193^ dimer is shown as a pale cyan cartoon representation, the mutation sites related to neurodevelopmental disability and epilepsy are shown as sticks and are coloured raspberry red. The CK2β^1-193^ structure (PDB_ID: 3EED (Raaf et al., 2008)) was used for the figure. The figure was drawn using PyMOL (Schrödinger 2013). **(D)** Conserved residues in chain A of the CK2β dimer in spacefill representation. The figure was created analogously to Figure 3B with the ConSurf server (Glaser et al., 2003; Landau et al., 2005; Ashkenazy et al., 2010; Celniker et al., 2013; Ashkenazy et al., 2016). The CK2β^1-193^ structure (PDB_ID: 3EED (Raaf et al., 2008)) was used as input file. Multiple Sequence Alignment was built using MAFFT (Katoh et al., 2018). The Homologues were collected from UNIREF90 using the homolog search algorithm HMMER (Eddy 2011) (E-value: 0.0001; No. of HMMER Iterations: 1). As maximal identity between sequences 95% ID and as minimal identity for homologs 40% ID were chosen. The calculation was performed on a sample of 150 sequences (see Supplementary Data Sheet S4) that represent the list of homologues to the query. Conservation scores were calculated using the Bayesian method (Mayrose et al., 2004).

Disease-causing mutations are more likely to occur in evolutionary conserved positions in proteins (Michaelson et al., 2012; Yuen et al., 2016; Geisheker et al., 2017; Lelieveld et al., 2017; Wang et al., 2020); therefore, we assessed whether the residues mutated in CK2α and CK2β in OCNDS and POBINDS were conserved across species including vertebrates, invertebrates, plants, yeast and fungi. Supplementary Figures S1, S2 show an alignment of a subset of species using MUSCLE (highest conserved residues highlighted in blue), with the residues mutated in OCNDS and POBINDS highlighted in red. Supplementary Figures S3, S4 show an alignment of 150 species (highest conserved residues highlighted in raspberry red) using MUSCLE. The accession numbers of the 150 sequences can be found in Supplementary Table S3. Figure 4 shows a 3D representation of alignment of 150 species using a 3D surface representation of the conserved residues calculated using the ConSurf server (Glaser et al., 2003; Landau et al., 2005; Ashkenazy et al., 2010; Celniker et al., 2013; Ashkenazy et al., 2016), and Supplementary Figure S5 shows the 3D surface representation using the subset of sequences in Supplementary Figures S1, S2. The alignments showed that the highest conservation across species was found in the first 324 CK2α residues. The CK2β sequence is less conserved than the CK2α sequence and there was a higher degree of conservation between vertebrate and invertebrate sequences and between plant and unicellular organisms (Supplementary Figure S5). Chantalat et al. (Chantalat et al., 1999) found 40 identical residues in the seven species they investigated, which are highlighted in Supplementary Figure S2. Among these conserved residues, 32, 34, 35 and 86 have missense mutations in POBINDS (defined by Chantalat as surface cluster 1, and discussed in the structural section). As discussed above, based on the NMC analysis, these residues could form part of novel functional domain(s).

Prediction programs based on evolutionary conservation are frequently used to determine the possible consequences associated with mutations. We used two well-known prediction programs, MutationTaster2 and PANTHER, to analyze CK2α and CK2β mutations (Supplementary Table S4). In MutationTaster2, all CK2α mutants were classified as disease causing and in PANTHER, all mutants were classified as probably damaging except for S356T (possible damaging). For CK2β mutants, both MutationTaster2 and PANTHER classified the mutants as disease causing/probably damaging. There was a concordance between the two prediction programs even for residues not fully conserved across species.

### Functional predictions

**FIGURE 5:**
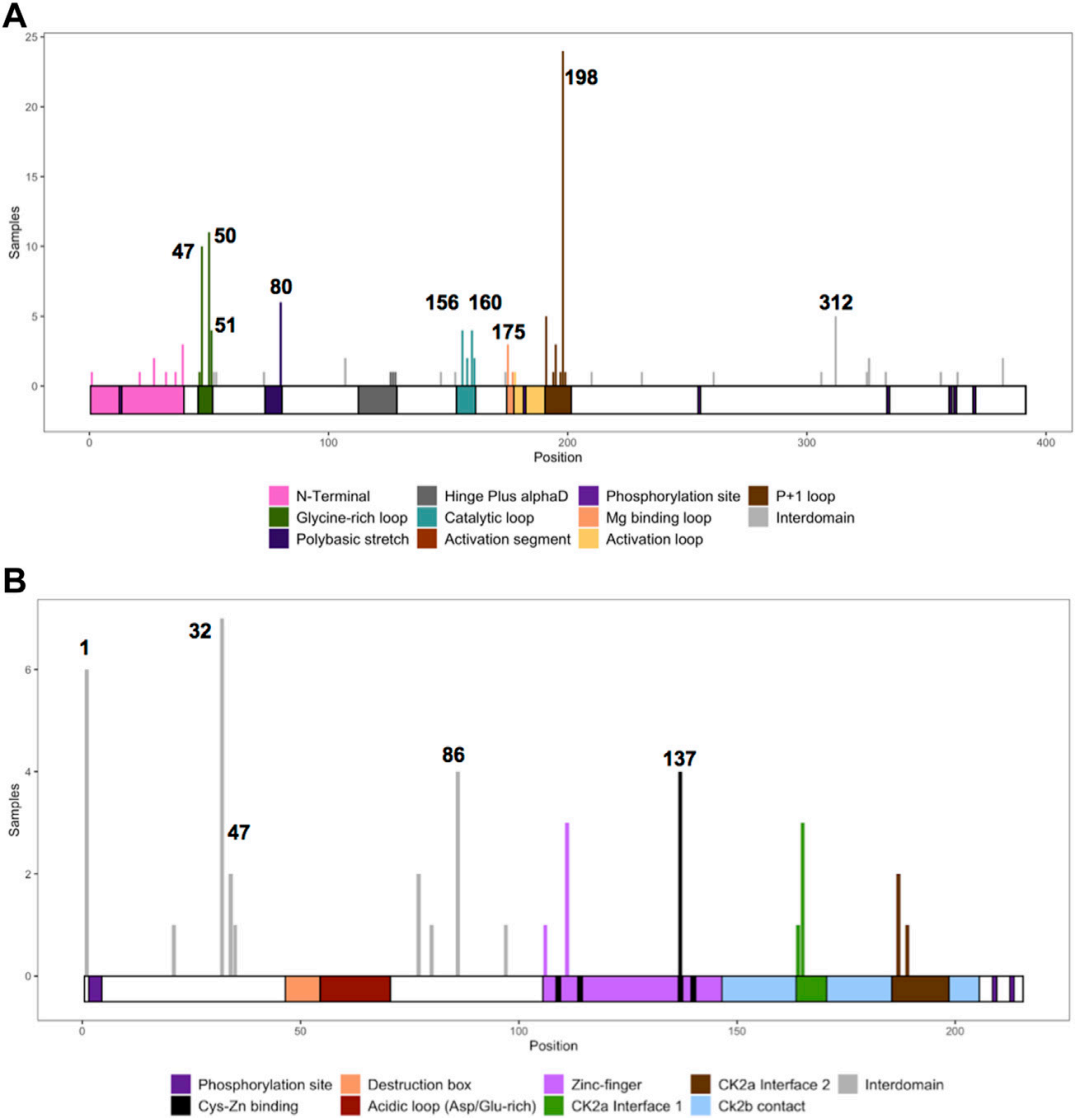
Histogram representing the number of missense mutations along the primary sequence of CK2α and CK2β. The Y axis represents the number of patients with missense mutations in each amino acid residue along the primary structure of CK2α **(A)** and CK2β **(B)**. The X-axis represents the primary sequence and the functional domains of CK2α **(A)** and CK2β **(B)**. Numbers mark single residues found significant in the non-random mutation cluster (NMC) analysis.

We assessed the functional effects of CK2α and CK2β mutations with six functional prediction programs (SIFT, PROVEAN, I-Mutant 3.0 Disease, MutationAssessor, Polyphen-2, and SNAP2) and two consensus prediction programs (REVEL and PredictSNP). The majority of the programs only analyze missense mutations; therefore, the computational analyses below are restricted to these mutations. Figure 5 represents the residues and number of patients with missense mutations.

For CK2α, the mutations predicted to be functionally impacted across all programs were D156H, D156E, D156Y, K158E, N161S, N161D, P231R, R312Q, and R312W (Table 5A). Mutations Y50C, Y50S, S51I, S51R, V53L, C147Y, K158R, G177S, S194F, and F197I may also be highly affected as none of the programs predicted these mutations to be neutral, tolerated, or benign. In addition to these mutations, M1?, G46V, M153R, D175G, D175E, and L178W were predicted to be damaging by both consensus programs. Mutations S356T, P363H and P382L were predicted neutral/benign/non-disease causing in most of the programs. For the rest of the mutations there were discrepancies between the programs. For CK2β, the mutations predicted to be functionally impacted across all programs were D32A, N35K, R86C, R111P, C137R, C137G, C137F, and H165R (Table 5B). The mutations D32N, F34S, F106V, L187P, and L187R may also be highly affected because none of the programs predicted these mutations to be neutral/tolerated/benign. Six prediction programs also provided numerical values associated with the predictions (Supplementary Table S5).

**TABLE 5:** Analysis of CK2α and CK2β protein mutations using functional prediction programs. Predictions for CK2α **(A)** and **(B)** CK2β. Colors in the cells reflect the effect from neutral (white) to high effect (red). The numbers obtained in the analysis can be found in Supplementary Table S4. **(C, D)** Comparison between CK2α predictions and experimental data on CK2α from Dominguez et al., 2021. (C) kinase activity of the recombinant CK2α protein mutants purified from bacteria; from 0% to 25% (top rows) to 25%–50% (bottom rows) compared to wild-type purified protein. (D) Degree of similarity between nuclear/cytoplasmic localization CK2α protein mutants to wild-type proteins expressed in cell lines; from low to high. N/A (= not applicable) indicates residues that could not generate a prediction. [Note: MutationAssessor has been down precluding us from analyzing 7 mutants, indicated with (—)].

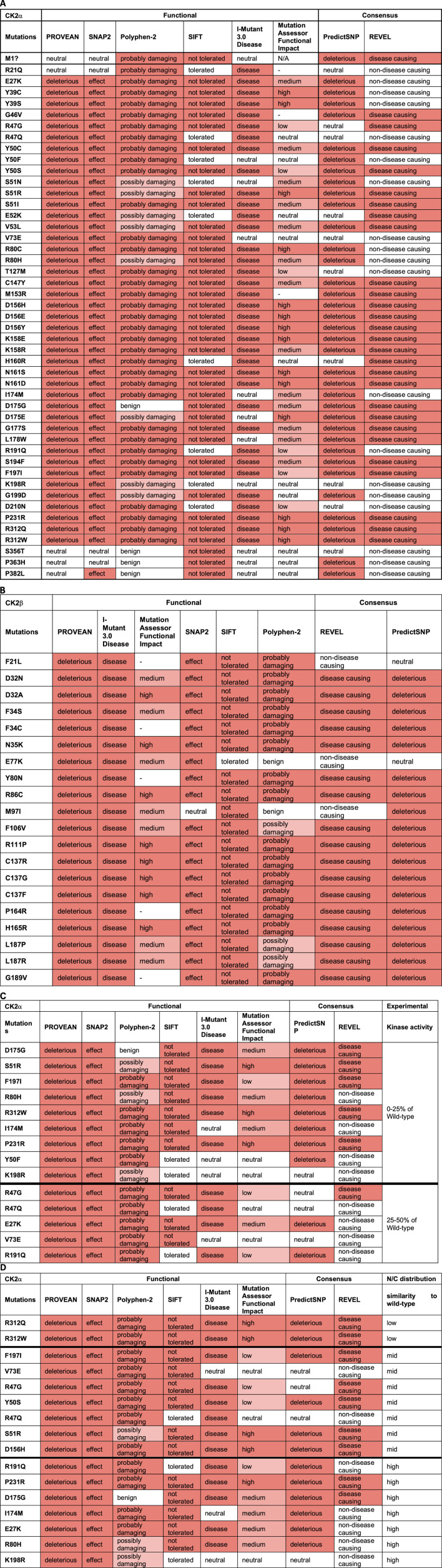

We performed McNemar’s tests to determine whether there were statistically significant differences in the categorial values between the programs. For this, each categorical value was coded as either as “benign” (neutral, benign, tolerated, non-disease causing) or “effect” (deleterious, effect, probably damaging, possibly damaging, not tolerated, disease, low/medium/high impact, disease causing). We then calculated percent agreement, the Kappa coefficient (to address agreement beyond what would be expected by chance) and the *p*-value in McNemar’s test (Table 6A,B). For CK2α functional predictions, REVEL had the least agreement with most other programs followed by Mutation Assessor Functional Impact, while SIFT and PredictSNP had agreement with the most number of programs. For CK2β there were fewer significant agreements between programs, probably due to limited sample size.

**TABLE 6:** McNemar’s test of categorical values. Each categorical value was coded as either a benign or effect. We calculated percent agreement, the Kappa coefficient and performed McNemar’s test to determine statistically significant differences in categorical values.

	PROVEAN	SNAP2	Polyphen-2	SIFT	I-Mutant 3.0 disease	MAFI	PredictSNP
SNAP2	κ = 0.8773 (*p* = 0.5637)						
Polyphen-2	κ = 0.6319 (*p* = 0.5637)	κ = 0.4535 (*p* = 0.999)					
SIFT	κ = 0.0070 (*p* = 0.2482)	κ = 0.0408 (*p* = 0.1317)	κ = -0.1336 (*p* = 0.1655)				
I-Mutant 3.0 Disease	κ = 0.4382 (*p* = **0.0027**)	κ = 0.3597 (*p* = 0**.0016**)	κ = 0.2316 (*p* = 0**.0039**)	κ = 0.1496 (*p* = 0.1967)			
MAFI	κ = 0.3968 (*p* = 0**.0082**)	κ = 0.2773 (*p* = 0**.0047**)	κ = 0.3410 (*p* = 0**.0339**)	κ = 0.4397 (*p* = 0.4795)	κ = 0.4788 (*p* = >0.999)		
PredictSNP	κ = 0.1455 (*p* = 0.1317)	κ = 0.1869 (*p* = 0.0578)	κ = 0.0242 (*p* = 0.0833)	κ = 0.5386 (*p* = 0.7055)	κ = 0.1132 (*p* = 0.3173)	κ = 0.5275 (*p* = 0.7055)	
REVEL	κ = 0.1983 (*p* = 0**.0005**)	κ = 0.1399 (*p* = 0**.0003**)	κ = 0.8773 (*p* = 0.5637)	κ = 0.3244 (*p* = 0**.0075**)	κ = 0.3081 (*p* = 0.1967)	κ = 0.3886 (*p* = 0**.0209**)	κ = 0.2828 (*p* = 0**.0201**)
SNAP2	κ = * (*p* = 0.3173)						
Polyphen-2	κ = * (*p* = 0.1573)	κ = 0.6429 (*p* = 0.3173)					
SIFT	κ = * (*p* = 0.3173)	κ = −0.0526 (*p* = >0.999)	κ = 0.6429 (*p* = 0.3173)				
I-Mutant 3.0 Disease	**	κ = * (*p* = 0.3173)	κ = * (*p* = 0.1573)	κ = * (*p* = 0.3173)			
MAFI	**	κ = * (*p* = 0.3173)	κ = * (*p* = 0.1573)	κ = * (*p* = 0.3173)	**		
PredictSNP	κ = * (*p* = 0.1573)	κ = −0.0714 (*p* = 0.5637)	κ = 0.4444 (*p* = >0.999)	κ = 0.6429 (*p* = 0.3173)	κ = * (*p* = 0.1573)	κ = * (*p* = 0.3173)	
REVEL	κ = * (*p* = 0.0833)	κ = 0.4595 (*p* = 0.1573)	κ = 0.7727 (*p* = 0.3173)	κ = 0.4595 (*p* = 0.1573)	κ = * (*p* = 0.0833)	κ = * (*p* = 0.1573)	κ = 0.7727 (*p* = 0.3173)

	PremPS	Kinact mCSM ΔΔG	Kinact SDM ΔΔG	Kinact DUET ΔΔG
Kinact mCSM ΔΔG	κ = −0.0320 (*p* = 0.5637)			
Kinact SDM ΔΔG	κ = −0.0854 (*p* = **0.0126**)	κ = 0.1296 (*p* = **0.0016**)		
Kinact DUET ΔΔG	κ = 0.1357 (*p* = **0.0339**)	κ = 0.1887 (*p* = **0.0082**)	κ = 0.2621 (*p* = 0.3657)	
I-Mutant 3.0 ΔΔG	κ = * (*p* = 0.1573)	κ = * (*p* = 0.3173)	κ = * (*p* = 0**.0009**)	κ = * (*p* = **0.0047**)

	PremPS
I-Mutant 3.0 ΔΔG	**

κ = Kappa coefficient, MAFI = Mutation Assessor Functional Impact**,** p = *p*-value from McNemar’s test. * Could not be computed, ** Could not compute κ or *p*-value.

*p*-values in bold are significant (*p* μ 0.05).

Since disease-causing mutations are more likely to have common structural features, these computational analyses could identify the best mutant candidates for experimental testing of the functional impact. A complexity in using these programs is that they each have their own thresholds for determining what is damaging and the extent to which a mutation is damaging. For example, some programs predict nearly all mutations to be damaging, while other programs categorize mutations based on predicted severity. In addition, not all programs consider other factors, such as position of the mutation, which have a significant impact on the consequence of the mutation. For instance, in the case of M1?, some of the programs predicted this mutation to be neutral/benign/tolerated and did not take into consideration that this is a mutation at the start codon, and therefore cannot not be neutral. Therefore,

Our next aim was to compare the results from these computation analyses with experimental data to determine which prediction program(s) can best help guide us in understanding the potential consequences of CK2α and CK2β mutations. We acknowledge that we have limited experimental data to date, particularly for CK2β, nonetheless, we correlated experimental data, in particular, kinase activity and subcellular localization with the functional predictions. The *in vitro* kinase activity of 18 CK2α recombinant mutant proteins purified from bacteria ranges from 0% to 50% of wild-type protein (Dominguez et al., 2021). Table 5C displays the CK2α mutants sorted from low (0% for D175G, S51R and F197I) to medium activity (50% for V73E and R191Q) compared with the wild type protein. Given that all of these mutants had less than 50% activity, PROVEAN and SNAP2 could be used to find mutants that have impacted activity since these two programs properly categorized all of these mutants as having an effect (except M1?). For the rest of the programs, we assessed whether their categorical values may have discrimination power between mutants with the less activity (0%–25%) compared to those with higher activity (25%–50%) (bold line in the table). Based on this cut-off, PredictSNP appears to most accurately predict most of the 0%–25% range to be deleterious (eight out of nine mutations) and it predicts less of the 25%–50% to be deleterious (only 2 out of five mutations). This suggests that predictSNP may be able to pinpoint the mutants most affected and may be utilized to predict large activity changes. We also investigated the numerical predictions to determine whether we can set a range of values for mutants more or less affected (Supplementary Table S5). For CK2α, SNAP2 and REVEL showed somewhat overlapping ranges. For SNAP2: mutants with 0%–25% activity were in the range 96–6 and those with 25%–50% activity in the range: 79–31, suggesting that the most affected mutants could be found in the 96–79 range. For REVEL: mutants with 0%–25% activity were in the range 0.902–0.361 and mutants with 25%–50% activity were in the range: 0.552–0.357, suggesting that the most affected mutants could be found in the 0.902–0.552 range.

We also compared these prediction results with experimental data on nuclear/cytoplasmic distribution of CK2α mutant proteins (Dominguez et al., 2021) as the subcellular distribution could be an indicator of the similarity to the wild-type protein structure (e.g., folding). CK2α wild-type is predominantly nuclear in 80% of cells. To assess the predictions, we categorized the mutants into three groups: high, medium and low similarity to wild-type depending on the percentage of cells with predominantly nuclear distribution. High similarity mutants to wild-type had approximately 80% nuclear distribution (E27K, R80H, I174M, D175G, R191Q, K198R, P231R), medium similarity mutants had 50%–70% nuclear distribution (R47G, R47Q, Y50S, S51R, V73E, D156H and F197I), and low similarity mutants had 20%–30% nuclear distribution (R321Q/W). Table 5D displays the CK2α mutants from low to high similarity to wild-type nuclear distribution. There does not seem to be a clear correlation between the categorial predictions and CK2α subcellular distribution for any of the programs. We also investigated the numerical predictions to determine whether we can find a range of values to distinguish mutants more or less affected (Supplementary Table S5). SNAP2 and MutationAssessor showed somehow overlapping ranges, as follows. For SNAP2, these were the range 96–90 (20%–30% nuclear distribution); range: 91–31 (50%–70% nuclear distribution), and range 89–6 (80% nuclear distribution) suggesting that the most affected mutants could be found in the 96–91 range. For MutationAssessor, these were range >4.61 (20%–30% nuclear distribution), range 4.21–0.525 (50%–70% nuclear distribution), and range 3.865–0.095 (80% nuclear distribution), suggesting that the most affected mutants would be ranked >4.61 and the less affected mutants ranked <0.525.

More data is needed to determine the predictive power of these programs as it relates to CK2α and CK2β, including computing of more complex characteristics that can be affected by changes in the primary structure of these proteins, such as interaction and oligomerization.

### Protein stability predictions

We utilized PremPS, Kinact, and I-Mutant 3.0 ΔΔG to predict protein stability changes of missense mutations. All three of these programs were utilized for CK2α mutations. Only PremPS and I-Mutant 3.0 ΔΔG were used for CK2β mutations since Kinact is specifically for kinase proteins. The prediction programs provide the Gibbs Free energy (kcal/mol) for change in protein stability. As in the above analyses only missense mutations were analyzed (Table 7).

**TABLE 7:** Analysis of the CK2 protein mutations using stability prediction tools. Predictions for each mutant in CK2α **(A)** and CK2β **(B)** according to each program. Highlight colors in the cells reflect the effect from stabilizing (white) to high destabilizing (red). The numbers obtained in the analysis can be found in Supplementary Table S5. **(C)** Comparison between predictions and experimental data from Dominguez et al., 2021. Table includes CK2α mutants whose expression in cell lines is significantly altered.

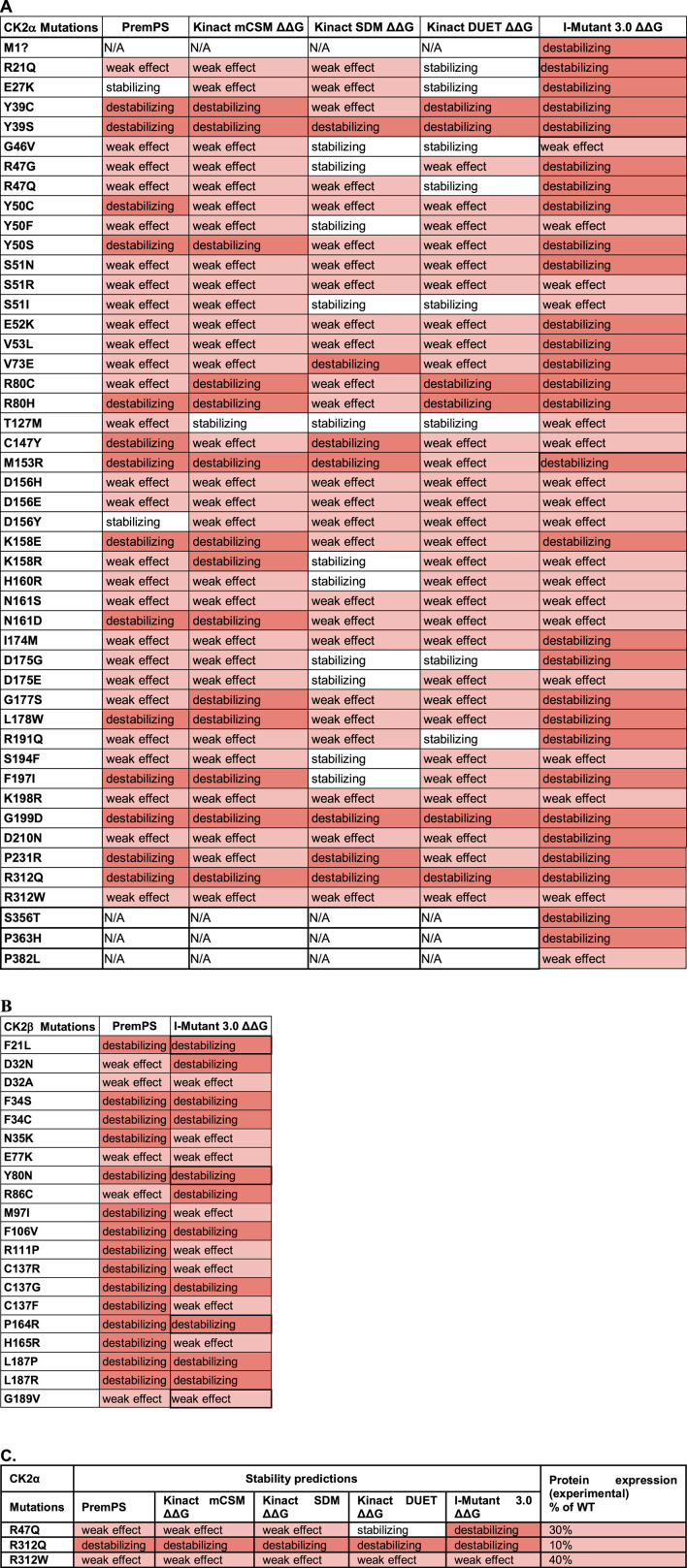

For CK2α, mutations Y39S, G199D, and R312Q were predicted to be highly destabilizing across all programs (Table 7A; Supplementary Table S6). Mutations Y39C, Y50S, R80C, R80H, K158E, L178W, and P231R may also be destabilizing as several programs predicted them to be highly destabilizing and none of the programs gave them a stabilizing prediction. Predictions were somehow different between the programs. Kinact mCSM and I-Mutant 3.0 ΔΔG predicted all mutants to be destabilizing at some level, and PremPS predicted all mutants to be destabilizing at some level except for E27K and D156Y. Both Kinact SDM and Kinact DUET predicted all mutations to be destabilizing except for a few, including G46V, S51I, T127M and D175G that both programs predicted stabilizing. For CK2β, both PremPS and I-Mutant 3.0 ΔΔG predicted all CK2β mutations to be destabilizing at some level (Table 7B; Supplementary Table S6). Mutations F34S, F34C, F106V, C137G, L187P, and L187R were predicted to be highly destabilizing by both programs while D32A and E77K were predicted to have a lower destabilizing effect. We performed McNemar’s tests for the differences in the categorial values between the programs (Tables 6C,D). For CK2α stability, Kinact SDM ΔΔG and Kinact DUET ΔΔG had the least agreement with other programs (significant *p*-value).

A complexity of the use of these programs is that these prediction programs required a structure input, except for I-Mutant 3.0 ΔΔG that used protein sequence. Overall, there are 287 CK2α structures in the PDB; 228 of them are from the two human paralogs CK2α and CK2α. Selecting the structure to use may be difficult for the newcomer to the field. Indeed, we obtained varied results when using three different structures for human CK2α to run Kinact (PDB ID’s 2PVR, 5OMY, 2ZJW) (not shown). For the data in the table, we used PDB ID 2PVR as it was the structure with the highest resolution.

There is no experimental study of the stability of CK2α mutants to be compared with the results from these program analyses. However, there is data on expression levels from a subset of CK2α mutant proteins that were expressed in cell lines (Dominguez et al., 2021). Three mutants appear less expressed than others in cell lines: R312Q, R312W and R47Q. As we will discuss below, R312 is a key residue to maintain the 3D structure of CK2α as it forms an ion pair with Q201 in α-EF helix, coupling the a-GHI-subdomain with the α-EF-helix; therefore, it is predicted to be less stable. The mutant with the lowest expression, R312Q, was predicted to be destabilizing by all programs (Table 7C). We also investigated the numerical predictions to determine whether we can find a range of values to distinguish mutants more or less affected (Supplementary Table S6). For CK2α, the three less expressed mutants had I-Mutant 3.0ΔΔG scores from −1.27 to 0.73. For CK2β, T37Yfs*5 had low expression levels (Nakashima et al., 2019). However, we could not assess this mutant, as the prediction programs only analyze missense mutations. More experimental data is needed to determine specific programs that are better at predicting stability properties of the CK2α and CK2β mutants.

#### Prediction of changes in binding free energy induced by point mutations

The binding free energy change between the wild type and mutant complex (ΔΔG) was predicted using BeAtMuSiC (http://babylone.ulb.ac.be/beatmusic) (Dehouck et al., 2013). For this, the binding of one CK2α chain to a CK2β dimer was evaluated, using the CK2 holoenzyme structure (PDB ID: 4DGL; (Lolli et al., 2012)) as input file. Individual side chains whose contributions strongly dominate the binding affinity of protein-protein interactions—so-called “hotspots”—(Clackson and Wells 1995) are usually defined as positions which cause an increase of binding free energy of more than 2.0 kcal/mol upon mutation. BeAtMuSiC identifies a residue as part of the protein–protein interface if its solvent accessibility in the complex is at least 5% lower than in the individual partner (Dehouck et al., 2013). First, ΔΔG values for experimentally characterized CK2α and CK2β mutants were predicted and compared to the experimental data (Table 8A,B). Indeed BeAtMuSiC predicted the highest ΔΔG values for the exchanges to Ala for the described hotspots of the CK2α/CK2β interaction, namely Leu41 and Phe54 of CK2α (Raaf et al., 2011) as well as Tyr188 and Phe190 of CK2β (Laudet et al., 2007) (Table 8A,B).

**TABLE 8:** Binding free energy changes between the wild type and mutant complexes. ΔΔG values were predicted using BeAtMuSiC (Dehouck et al., 2013). For this structure based approach, the binding of one CK2α chain (chain C) to the CK2β dimer (chains A/B) was evaluated, using the CK2 holoenzyme structure (PDB_ID 4DGL; (Lolli et al., 2012)) as input file. Mutations which cause an increase of binding free energy of more than 2.0 kcal/mol upon mutation are highlighted in red in the first column. Positive ΔΔG values indicate that the mutation decreases binding affinity (in red), and negative ΔΔG values indicate that the mutation increases binding affinity (in green). BeAtMuSiC (Dehouck et al., 2013) identifies a residue as interface residue if its solvent accessibility in the complex is at least 5% lower than in the unbound form. (A) ΔΔG values for previously experimentally characterized CK2α variants (Raaf et al., 2011). (B) ΔΔG values for previously experimentally characterized CK2 variants or corresponding exchanges in a CK2β-derived cyclic peptide including CK2β residues Arg186 to His193 (Laudet et al., 2007). (C) ΔΔG values for OCNDS-related CK2α variants. (D) ΔΔG values for POBINDS-related CK2β variants.

CK2α Mutations	ΔΔGBind (kcal/mol)	Solvent accessibility (in partner(s))	Solvent accessibility (in complex)	Interface
L41A	**3.52**	53.34%	0%	Yes
F54A	**2.92**	21.10%	0%	Yes
I69A	**2.35**	27.07%	2.76%	Yes

CK2β Mutations	ΔΔGBind (kcal/mol)	Solvent accessibility (in partner(s))	Solvent accessibility (in complex)	Interface
M166A	**1.53**	22.62%	11.55%	yes
R186A	**1.35**	43.95%	36.13%	yes
L187A	**1.94**	12.69%	6.99%	yes
Y188A	**2.45**	71.58%	46.03%	yes
G189A	**1.74**	46.54%	23.84%	yes
F190A	**3.63**	68.45%	33.21%	yes
K191A	**0.87**	61.78%	46.50%	yes
I192A	**1.49**	17.13%	14.36%	
H193A	**1.34**	60.99%	37.78%	yes

CK2α Mutations	ΔΔGBind (kcal/mol)	Solvent accessibility (in partner(s))	Solvent accessibility (in complex)	Interface
R21Q	**−0.01**	58.20%	58.20%	
E27K	**0.24**	40.82%	40.82%	
Y39C	**1.12**	11.82%	2.96%	Yes
Y39S	**1.76**	11.82%	2.96%	Yes
G46V	**−0.1**	34.05%	34.05%	
R47G	**1.69**	69.92%	29.30%	Yes
R47Q	**0.79**	69.92%	29.30%	Yes
Y50C	**0.7**	50.25%	43.07%	Yes
Y50F	**0.21**	50.25%	43.07%	Yes
Y50S	**0.51**	50.25%	43.07%	Yes
S51N	**0.31**	24.65%	24.65%	
S51R	**0.27**	24.65%	24.65%	
S51I	**0.01**	24.65%	24.65%	
E52K	**0.45**	31.15%	11.28%	Yes
V53L	**0.24**	14.59%	14.59%	
V73E	**0.75**	32.83%	25.53%	Yes
R80C	**0.7**	10.94%	10.94%	
R80H	**0.36**	10.94%	10.94%	
T127M	**0.21**	54.43%	54.43%	
C147Y	**0.27**	0%	0%	
M153R	**0.41**	0.49%	0.49%	
D156H	**0.01**	11.34%	11.34%	
D156E	**0.38**	11.34%	11.34%	
D156Y	**0.13**	11.34%	11.34%	
K158E	**0.38**	20.81%	20.81%	
K158R	**0.06**	20.81%	20.81%	
H160R	**−0.03**	74.07%	74.07%	
N161S	**0.49**	2.42%	2.42%	
N161D	**0.49**	2.42%	2.42%	
I174M	**0.42**	13.26%	13.26%	
D175G	**0.16**	39.07%	39.07%	
D175E	**0.5**	39.07%	39.07%	
G177S	**0.16**	13.62%	13.62%	
L178W	**−0.08**	21.75%	21.75%	
R191Q	**0.01**	68.75%	68.75%	
S194F	**0.18**	20.80%	20.80%	
F197I	**0.76**	6.28%	6.28%	
K198R	**0.2**	9.30%	9.30%	
G199D	**0.21**	0%	0%	
D210N	**0.25**	5.04%	5.04%	
P231R	**0.86**	12.26%	12.26%	
R312Q	**0.41**	1.17%	1.17%	
R312W	0.21	1.17%	1.17%
S356T	n.d.	n.d.	n.d.	n.d.
P363H	n.d.	n.d.	n.d.	n.d.
P382L	n.d.	n.d.	n.d.	n.d.
**D**				
**CK2α Mutations**	**ΔΔGBind (kcal/mol)**	**Solvent accessibility (in partner(s))**	**Solvent accessibility (in complex)**	**Interface**
F21L	**1.23**	1.80%	1.80%	
D32N	**0.12**	50.72%	48.83%	
D32A	**0.22**	50.72%	48.83%	
F34S	**0.39**	75.85%	51.17%	Yes
F34C	**0.88**	75.85%	51.17%	yes
N35K	**0.66**	24.17%	22.36%	
E77K	**0.03**	49.95%	49.95%	
Y80N	**2.4**	0%	0%	
R86C	**0.96**	28.91%	28.12%	
M97I	**0.37**	0%	0%	
F106V	**1.42**	0.45%	0.45%	
R111P	**0.56**	8.79%	8.79%	
C137R	**2.25**	0%	0%	
C137G	**2.04**	0%	0%	
C137F	**0.96**	0%	0%	
P164R	**−0.43**	0%	0%	
H165R	**0.29**	1.23%	1.23%	
L187P	**2.25**	12.69%	6.99%	yes
L187R	**1.04**	12.69%	6.99%	yes
G189V	**1.59**	46.54%	23.84%	yes

Among the OCNDS-linked CK2α mutants, Tyr39Ser, Tyr39Cys and Arg47Gly have the highest ΔΔG values (1.76 kcal/mol, 1.12 kcal/mol and 1.69 kcal/mol) but are not considered binding hotspots as they are close but not directly involved in the interaction site (although the BeAtMuSiC algorithm recognizes them as interface residues) (Table 8C). Residues not in the interface were not predicted to change the affinity for CK2β. This hypothesis is supported by the literature, at least in the case of Lys198Arg (Werner et al., 2022).

For CK2β, the interface mutation Leu187Pro has a predicted ΔΔG value of 2.25 kcal/mol (Table 8D). Laudet et al. included Leu187 in a CK2β-derived cyclic peptide mimicking the C-terminal CK2β hairpin loop (Arg186 to His193) essential for binding of CK2α (Laudet et al., 2007). In this study, each amino acid of the cyclic peptide was individually replaced by Ala and the derivatives of the original peptide were explored regarding their antagonistic activity in a CK2α/CK2β-binding assay. The exchange to Ala of the Leu187 equivalent position in the peptide caused a marginal reduction of CK2α/CK2β-binding and the role of this residue was interpreted as passive. Indeed, the Leu187Ala mutation is predicted to induce a smaller change in binding free energy (1.94 kcal/mol; Table 8B) than the POBINDS-related mutation Leu187Pro (Table 8D). Compared to the Leu187Arg variant with a predicted ΔΔG value of 1.04 kcal/mol, the Leu187Pro variant is predicted to have a stronger impact on the CK2 subunit interaction. It has to be noted that other variants of CK2β with a predicted ΔΔG value higher than 2 kcal/mol are not located in the interface, namely Tyr80Asn, Cys137Arg and Cys137Gly. As described later, Tyr80 and Cys137 are important for the global architecture of CK2β and hence, the high ΔΔG values for the exchange of these residues may be due to changes in folding free energy.

Therefore, this analysis identified Leu187Pro in CK2β as a potential mutation with consequences for CK2 holoenzyme formation. Further analyses could test the influence of mutations on the oligomerization of the holoenzyme, pseudosubstrate region or the binding to other partners.

#### A structural perspective on the OCNDS-related CSNK2A1 mutations

**FIGURE 6:**
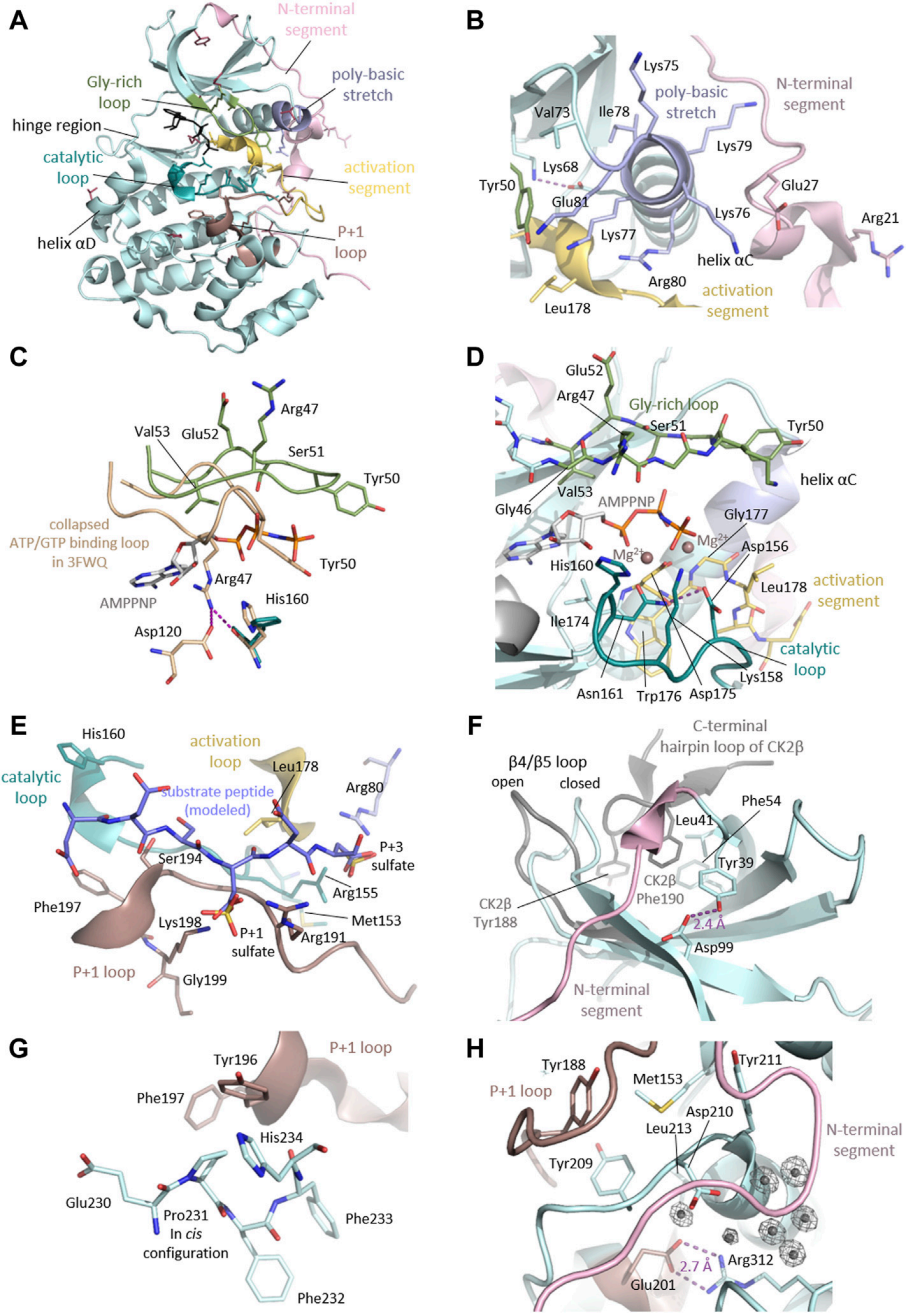
Detailed CK2α functional and structural domains. **(A)** Clustering of mutation sites in the catalytic and structural key elements of CK2α. The side chains of the mutated residues are shown as sticks and are coloured according to the different elements as described in the figure. Mutation sites not residing in these elements are highlighted in raspberry red. The CK2α^1-335^ structure in complex with AMPPNP (PDB_ID: 2PVR (Niefind et al., 2007)) was used for the figure. **(B)** Mutation sites in the N-terminal segment and the helix αC. The N-terminal region of CK2α with the mutation sites Arg21 and Glu27 drawn in light pink. The helix αC, which is located adjacent to mutation site Val73, harbors the basic cluster (light blue) including the mutation site Arg80. The critical salt bridge between Lys68 and Glu81 is shown as well as the mutation sites residing in proximity such as Tyr50 from the Gly-rich loop (pale green) and Leu178 of the activation loop (yellow). The CK2α^1-335^ structure PDB_ID: 2PVR (Niefind et al., 2007) was used for the figure. **(C)** Mutation sites in the Gly-rich loop. The Gly-rich loop is shown in its open conformation (pale green, PDB_ID: 2PVR (Niefind et al., 2007)) and collapsed conformation (wheat, PDB_ID: 3FWQ (Raaf et al., 2009). The mutation sites of the Gly-rich loop Arg47, Tyr50, Ser51, Glu52 and Val53 are shown as sticks, as well as AMPPNP. In the collapsed conformation, Arg47 forms hydrogen bonds to Asp120 and His160, which are depicted as purple dashed lines. **(D)** Mutation sites in the active site of CK2α. The mutation sites in the catalytic loop (Asp156, Lys158, His160 and Asn161) are drawn as dark cyan sticks. The activation loop is shown as stick representation, the respective mutation sites are Gly177 and Leu178. The Gly-rich loop is drawn as sticks and the mutation sites Arg47, Tyr50, Ser51, Glu52 and Val53 are labelled. Magnesium ions are shown as dark salmon spheres. The maize CK2α structure in complex with AMPPNP and magnesium ions (PDB ID: 1LP4 (Yde et al., 2005)) served as a reference for the binding mode of AMPPNP and magnesium ions in this figure. **(E)** Mutation sites involved in substrate binding. To illustrate the substrate binding mode, a peptide substrate (coloured blue) was modelled into the substrate binding site as described (Niefind et al., 2007). Sulfate ions present in the structure mark the anion binding sites for the substrate. Mutation sites which are relevant for substrate binding from the P+1 loop (Arg191 and Ser194, Phe197, Lys198 and Gly199, dark salmon), catalytic loop (Met153 and His160, dark cyan), activation loop (Leu178, yellow) and the basic cluster (Arg80, light blue) are shown as stick representations. In addition to the mutated residues, Arg155 is shown as stick representation, as it creates a positively charged anion binding site together with Arg80 which is required for substrate recognition at the P+3 position. The CK2α^1-335^ structure (PDB_ID: 2PVR (Niefind et al., 2007)) was used for the figure. **(F)** Mutation sites in the subunit interaction interface. Critical residues for binding on the CK2α (Leu41 and Phe54, pale cyan) and CK2β (Tyr188 and Phe190, grey) surface are shown as sticks. The mutation site Tyr39 is not directly involved in the interaction but stabilizes the protein fold through a hydrogen bond to Asp99. To illustrate the movement of the β4β5 loop upon assembly of the CK2 holoenzyme, the closed conformation of the CK2α monomer (pale cyan) and the open conformation of CK2α with bound CK2β (grey) are shown. The CK2α^1-335^ structure monomer (PDB_ID: 2PVR (Niefind et al., 2007)) and the CK2 holoenzyme structure (PDB_ID: 4DGL (Lolli et al., 2012)) were used for the figure. **(G)** *Cis*-configuration of the Pro231. The mutation site Pro231 is located in the C-terminal segment of CK2α. Pro231 was found as a *cis*-peptide in all CK2α structures published so far. Pro231 is located in proximity to the P+1 loop and its mutation to a non-proline residue lacking *cis*-peptide propensity may disturb the local or even global fold of the protein. The CK2α^1-335^ structure (PDB_ID: 2PVR (Niefind et al., 2007)) was used for the figure. **(H)** Mutation sites in the hydrophobic cluster around Met153 and the C-terminal region. The mutation site Met153 is part of a hydrophobic cluster and resides in close proximity to Leu213 and the aromatic rings of Tyr188, Tyr209, Tyr211. The mutation site Arg312 forms a critical salt bridge with Glu201 of the P+1 loop/activation segment. Position Arg312 is in close proximity to the mutation site Asp210 and to a critical water cluster, which mediates the contact of the N-terminal segment (light pink), the activation loop, and the αC helix, which keeps them in the active conformation. The CK2α^1-335^ structure (PDB_ID: 2PVR (Niefind et al., 2007)) was used for the figure. Electron densities around water molecules have a cutoff level of 2σ.

As we discussed above, the majority (57%) of the CK2α variants linked to OCNDS lead to missense mutations which cluster in highly conserved functional domains and key structural regions, such as the Gly-rich loop, the basic cluster, the activation loop and the P+1 loop (Figures 2A, 4, 6). Accordingly, the catalytic and structural key elements share a high score of conservation as represented in Supplementary Figures S1, Supplementary Data Sheet S3. Apart from residues involved in catalysis, certain mutations are located in the N- and C-terminal segments, which are important for the global fold of the kinase and are critical at stabilizing the constitutively active conformation (Figures 6B,H). Other mutations, such as Tyr39Cys, are located in the CK2β subunit interaction site (Figure 6F). Two of the reported mutations, Thr127Met and Cys147Tyr, are not located in functional domains. Thr127 is located in the helix αD and is exposed to the solvent, so its exchange with the hydrophobic Met might disturb solubility of CK2α.

The side chain of Cys147 is directed to the inside of the kinase and its replacement with the bulkier Tyr is likely sterically incompatible with the protein fold. These sites could be functional as Thr127 has been found phosphorylated (Phosphosite Plus) and Cys147 to be nitrosylated (Wu W et al., 2020; Borgo et al., 2021). For the nitrosylation at Cys147, however, the side chain must get exposed.

##### Missense mutations in the N-terminal segment

The N-terminal segment (residues 1-39) stabilizes the helix αC and the activation loop in their active conformations, and it is therefore a decisive element for the constitutive activity of CK2α (Niefind et al., 1998). Indeed, the deletion of the first 30 residues leads to loss of kinase activity (Sarno et al., 2002). The Met1? Mutation will affect the start codon, and is predicted to lead to an N-terminal truncation of the protein until amino acid 137, the position of the next in-frame start codon (Chiu et al., 2018). The mutation site Arg21 is directed towards the surface however, from a structural point of view, the consequences of the exchange with Gln cannot be anticipated. The likewise critical position Glu27 (Chiu et al., 2018) is in close contact to the basic cluster of helix αC and its exchange to Lys may have an repellent electrostatic effect (Figure 6B).

##### Missense mutations in the Gly-rich loop

The mutations Gly46Val, Arg47Gln/Gly, Tyr50Cys/Ser/Phe, Ser51Arg/Asn/Ile, Glu52Lys and Val53Leu reside in the highly flexible, Gly-rich loop (Figure 6C). Gly-rich loops are highly conserved among kinases and many other nucleotide-binding proteins. Gly residues are highly conserved in nucleotide positioning loops (NPLs) because of their minimal steric repulsion and their contribution to high backbone flexibility. In the protein kinase family the Gly-rich loop connects the β strands 1 and 2, and its sequence is **Gly**-X-**Gly**-X-X-**Gly**-X-Val (Grant et al., 1998). In CK2α however, the third Gly is replaced by a Ser residue: **Gly**_**46**_-Arg-**Gly**_**48**_-Lys-Tyr-**Ser**_**51**_-Glu-Val. The spatially undemanding Gly residues allow a close proximity of the loop backbone and the β- and γ-phosphates of ATP (and GTP in the case of CK2) (Niefind and Battistutta 2013). A systematic literature review revealed that mutations altering the Gly-rich loop are more likely to cause the widest range of phenotypes (Wu et al., 2021). Gly46 interacts with the ribose moiety of ATP/GTP (as shown in maize CK2α structures in complex with AMPPNP (Niefind et al., 1998) and GMPPNP (Niefind et al., 1999). Mutation of the equivalent position in PKA (Gly50) showed a 10-fold decrease of affinity for ATP (Grant et al., 1998), therefore Gly46Val may lead to a decreased affinity to ATP/GTP. Structural studies revealed that this particular loop can collapse so that Arg47 blocks the active site (Raaf et al., 2009). Tyr50 is of topological interest because cyclin-dependent kinases (CDKs) also carry a Tyr residue at the equivalent position. This tyrosine is an important phosphorylation site in CDKs because its dephosphorylation in a CDK/cyclin complex is necessary for full catalytic activity (Dorée and Galas 1994). In CK2α, there has been speculation about the regulatory significance of Tyr50 (Allende and Allende 1995). Mass spectrometry studies found Tyr50 to be phosphorylated (Gu et al., 2010; Schreiber et al., 2010) however, if putative phosphorylation of Tyr50 plays a role in the regulation of CK2 remains unresolved.

Val53 is a highly conserved residue in the catalytic spine (C-spine; residues: Leu41, Val53, Phe54, Val66, Val162, Met163, Ile164, Phe121, Met221, Met225) (Kornev et al., 2008; Taylor and Kornev 2011), and is located in the β2 strand at the end of the Gly-rich loop. The C-spine and its counterpart, the regulatory spine (R-spine; residues: Leu85, Leu97, His154, Trp176), are two stacks of hydrophobic side chains within the catalytic core of eukaryotic protein kinases (EPKs) extending from the C-lobe to the N-lobe of the kinase (Kornev et al., 2008). Unlike the R-spine, the C-lobe requires an external supplementation: it needs to be completed by the purine ring of ATP/GTP. Val53 is one of the residues sandwiching the purine moiety; hence, the introduction of Leu, another hydrophobic amino acid with a bulkier side chain, may subtly affect ATP/GTP cosubstrate binding.

The Gly-rich loop is in close proximity to the CK2α/CK2β subunit interface. Therefore, mutations in this loop might alter the flexibility of the Gly-rich loop or affect the assembly of the CK2 holoenzyme. GST-tagged CK2α Ser51Arg was inactive in an *in vitro* activity assay, while the Arg47Gln/Gly mutants showed 40%–50% activity compared to WT (Dominguez et al., 2021). The activity of these three GST-CK2α mutants remained unchanged in the presence of GST-CK2β. In contrast GST CK2α Tyr50Phe was partially rescued from 10% to 40% of *in vitro* kinase activity by addition of GST-CK2β, suggesting a potential conformation change due to the binding of CK2β. Noteworthy, the C-spine of CK2α has a remarkable extension that interacts with the interaction ‘‘hot spots’’ in CK2β: Leu41 and Phe54 (Bischoff et al., 2011b). Considering this, it makes even sense to test whether the mutation Val53Leu has an indirect effect on CK2β binding.

##### Missense mutations in the basic cluster

Val73 is located N-terminal of the CK2α typical KKKKIKRE-sequence which is important for substrate recognition and binding (Figure 6B). The exchange from Val to the negatively charged Glu in OCNDS may interfere with binding of the typically negatively charged substrates. Indeed, GST-CK2α Val73Glu had half the activity of the wildtype protein towards a synthetic negative charged peptide substrate, and could not be rescued by adding GST-CK2β. Arg80 is part of the RE-motif (Arg80-Glu81) which is characteristic for EPKs in the CMGC family. Arg80 resides in helix αC and is followed by Glu81. Glu81 forms a characteristic salt bridge with a conserved Lys68 (Figure 6B). Through this salt bridge, Lys68 is positioned in the correct conformation to bind the α and β phospho-groups of the ATP/GTP cosubstrate (Huse and Kuriyan 2002). Spatially, Arg80 is in close proximity to Arg155 to create a positively charged anion binding site required for substrate recognition at the P+3 position (Niefind et al., 2007) (Figure 6E). Therefore, loss of the positive charge upon exchange from Arg80 to His (Owen et al., 2018) or Cys (Wu et al., 2021) likely interferes with substrate binding, and might also disturb the conformation of the critical residue Glu81. Experimentally, GST-tagged CK2α Arg80His was only minimally active and the GST-Val73Glu mutant showed 40–50% activity compared to WT *in vitro* activity assays. Neither mutant is rescued by addition of GST-CK2β (Dominguez et al., 2021). This region of the protein is also involved in holoenzyme oligomerization however a mutation in the KKKKIKRE sequence would not have a big impact on oligomerization as the KKKKIKRE sequence is close to CK2β but the P+1 residues of the substrate binding site are significantly much closer.

##### Missense mutations in the active site

Met153 is a buried residue. Although its function is structural, it has been assigned to the active site in this study as it is located just before the beginning of the catalytic loop. Met153 is part of a hydrophobic cluster, and resides in close proximity to Leu213 and the aromatic rings of Tyr188, Tyr209, Tyr211 (Figure 6H). Met153 is located close to Arg155, which is involved in substrate binding at the P+3 site (Figure 6E). The exchange of Met153 to an Arg would significantly disrupt this hydrophobic arrangement and therefore the global folding of the protein.

Asp156 is located in the active site (Figure 6D). It is the catalytic base of the kinase, therefore the Asp156His or Asp156Tyr mutations likely abolish activity, as it was shown for the kinase dead mutant CK2α Asp156Ala (Korn et al., 1999). Even though the mutation Asp156Glu is electrostatically conservative, the exchange at this critical catalytic position may greatly affect kinase activity. The catalytic key residue Lys158 is highly conserved among EPKs and stabilizes the transition state of the phosphorylation reaction. An exchange of Lys158 to Glu/Arg may interfere with this stabilizing effect. In addition to their catalytic functions, it was shown that Asp156 and Lys158 contribute to heparin binding in a structure of CK2α complexed with the substrate competitive inhibitor heparin (Schnitzler and Niefind 2021). Analogously, both residues could be involved in substrate binding. His160 was shown to interact with the acidic residue of the substrate at the P-3 site (Schnitzler and Niefind 2021) (Figure 6E), therefore the substitution of His160 with Arg may sterically interfere with substrate binding. Asn161 coordinates Mg^2+^, which is essential for balancing the negative charges of ATP (or GTP). In addition to this, it forms a hydrogen bond to the catalytic base Asp156 (see Figure 6D). Exchange from the highly conserved Asn to Ser or Asp may interfere with these functions. Ile174 is a critical residue of the active site as well (Figure 6D). Together with the residues Met163, Val53 and Ile66 it forms a hydrophobic area to fix the adenine/guanine group of ATP/GTP (Niefind et al., 1998). Exchange with Met might interfere with ATP/GTP binding (Owen et al., 2018).

The Mg^2+^ binding Asp175 (Figure 6D) is a highly conserved residue among protein kinases and is involved in the coordination of both Mg^2+^ ions. GST-tagged CK2α Ile174Met showed 10% activity compared to WT, while GST-CK2α Asp175Gly was no longer catalytically active at all; Ile174Met showed a slight rescue by GST-CK2β (Dominguez et al., 2021). Gly177 is in close proximity to the Lys68-Glu81 salt bridge. A replacement with Ser might disturb the salt bridge and thereby alter catalytic activity. The intact Lys68-Glu81 salt bridge is critical for catalytic activity, and the formation of the corresponding salt bridges is involved in regulation of many EPKs. In the inactive state of many regulated EPKs, this critical salt bridge is broken and the helix αC is rotated. As part of coupled conformational rearrangements upon inactivation, the so-called DFG-motif at the beginning of the activation loop can rotate into the active site and block nucleotide and substrate binding (Huse and Kuriyan 2002). In CK2α, however, Gly177 is part of the equivalent DWG-motif (see Figure 6D) which—compared to the canonical DFG-motif—is internally stabilized by an additional hydrogen bond. Consistent with CK2α’s constitutive activity, the aforementioned rearrangements for inactivation have never been observed in CK2α. Leu178 is located close to the P+3 site of the substrate (Figure 6E), and replacement with the bulkier Trp residue might interfere with substrate binding.

##### Missense mutations in the P + 1 loop

The OCNDS-sensitive positions Arg191, Ser194, Phe197, Lys198 and Gly199 belong to the P+1 loop of the activation segment and are important for substrate recognition (Figure 6E). Mutational studies in which the combination of Arg191, Arg195 and Lys198 were replaced by alanines showed defective substrate recognition at the P+1 position and decreased inhibition by the substrate competitive inhibitor heparin (Sarno et al., 1996; Vaglio et al., 1996). In a complex structure of CK2α with heparin, it was shown that Arg191, Ser194, Phe197 and Lys198 are involved in heparin binding (Schnitzler and Niefind 2021). Changes in side chain charges in this region could alter the formation of regulatory holoenzyme oligomers.

Protein kinase C (PKC)-mediated phosphorylation at CK2α residues Ser194 and Ser277, and at CK2β residue Ser148 stimulate CK2 activity (Lee et al., 2016). Accordingly, the Ser194Phe mutation should interfere with the phosphorylation by PKC. Further phosphorylation sites in the proximity of the mutated residues are Tyr182 and Tyr188. Phosphorylation of Tyr182 and Tyr188 by Src-related kinase SRMS was shown to increase CK2α activity (Goel et al., 2018). Mutations near the phosphorylation sites may disturb the recognition by SRMS (Roffey and Litchfield 2021).

The Lys198Arg mutation is the most frequently observed variant in OCNDS patients and is considered as a mutation hotspot (Okur et al., 2016; Akahira-Azuma et al., 2018; Chiu et al., 2018; Owen et al., 2018; Nakashima et al., 2019; Xu et al., 2020). Lys198 was described as the key residue determining the anion binding potential of the P+1 loop (Sarno et al., 1995; Sarno et al. 1996; Sarno et al. 1997). The crystal structure of the CK2α Lys198Arg variant discloses a significant shift of a sulfate ion marking the anion binding site in the P+1 position (Werner et al., 2022). This observation supports the notion by Caefer et al. that the Lys198Arg mutation causes an alteration of substrate specificity (Caefer et al., 2022). In this recent publication (Caefer et al., 2022), a Proteomic Peptide Library Approach (ProPel) using CK2α Lys198Arg expressed in *E. coli* revealed that the mutation shifts substrate specificity by decreasing the preference for acidic residues in the P+1 position. Furthermore, the Lys198Arg mutation alters the phosphoacceptor preference: in contrast to the WT, CK2α Lys198Arg strongly disfavoured Thr phosphorylation and showed an increased preference for Tyr phosphorylation. The Lys198 equivalent position in protein kinase A (PKA-C) is Leu205 and, interestingly, the mutation Leu205Arg is linked to cortisol-secreting adrenocortical adenomas responsible for Cushing’s Syndrome. It was reported that the Leu205Arg mutation disrupts the binding of R (regulatory)-subunits; thereby, it renders the enzyme constitutively active (Calebiro et al., 2014) and it drastically changed the phosphorylation profile in a phosphoproteomic mapping (Lubner et al., 2018). Using NMR spectroscopy, thermodynamics, kinetic assays, and molecular dynamics simulations, Walker et al. (Walker et al., 2019) found that the Leu205Arg mutation causes global changes in PKA-C: it rewires the intra- and intermolecular interactions, and causes losses in nucleotide/pseudo-substrate binding cooperativity. By rewiring its internal allosteric network, PKA-C Leu205Arg is able to bind and phosphorylate non-canonical substrates. An additional interesting structural observation concerning Lys198 is that it stabilizes Arg193 in an unfavourable backbone conformation via hydrogen bonds. Similar tensions in the P+1 loop can be found in other CMGC Kinases, where tension and release are factors in the control mechanism (Huse and Kuriyan 2002). For CK2α, the tension at Ala193 is without any evident function (Niefind et al., 2007). GST-tagged CK2α Lys198Arg showed 20%–30% activity and CK2α Arg191Gln showed 40%–50% activity compared to wild type using a standard peptide (Dominguez et al., 2021).

The mutation Gly199Asp most likely changes the main characteristics of the P+1 loop and, therefore, the ability to bind substrates due to steric clashes of the larger residue may result in rearrangements of the P+1 loop. The substitution for an acidic residue will have a repulsive effect on the binding of acidic substrates.

##### Missense mutations in the C-terminal segment

This study defines the C-terminal segment as the sequence after the P+1 loop (residues 202–391). The OCNDS-associated mutations Asp210Asn, Pro231Arg, Arg312Gln/Trp, Ser356Thr, Pro363His, Pro382Leu and the C-terminal truncation Arg333* occur in the C-terminal segment. The GST-tagged variants of CK2α Pro231Arg and CK2α Arg312Gln lost 90% of their catalytic activity compared to WT, and Pro231Arg was rescued by CK2β (Arg312Gln was not tested) (Dominguez et al., 2021). A *cis*-peptide was found at Pro231, a in all CK2α structures published so far. The role of the Pro231 *cis*-peptide bond in CK2α (Figure 6G) is not known yet, but possibly the mutation to a non-proline residue lacking *cis*-peptide propensity destabilizes the local or even global fold of the protein. Position Arg312 forms a critical salt bridge with Glu201 of the activation segment. Arg312 is in close proximity to Asp210 which was substituted for Asn in OCNDS (Figure 6H). Both residues are located near a critical cluster of water molecules -observed in high-resolution CK2α structures- that mediates the close contact of the N-terminal segment, the activation loop, and the αC helix, keeping them in the active conformation. The C-terminus of CK2α is an important site of posttranslational modifications and mediates the interaction with peptidyl-prolyl isomerase Pin1 (Messenger et al., 2002). However, structural information about this region is not available except for a CK2α-derived peptide in complex with O-GlcNAc transferase (Lazarus et al., 2011). CK2α is phosphorylated in a cell cycle dependent manner by Cdk1/cyclin B at the positions Thr344, Thr360, Ser362 and Ser370 (Bosc et al., 1995) and can be O-GlcNAc-modified on Ser347 by O-GlcNAc transferase (Tarrant et al., 2012). Mutations in proximity to critical residues can potentially interfere with recognition motifs or binding site; for example, P363H may affect Thr360/Ser362 phosphorylation by CDK1 and MAPK1 (Ji et al., 2009).

##### Truncating and frameshift mutations

The majority of the truncating mutations (nonsense and frameshifts) likely result in the complete loss of the normal protein folding. Interestingly, the C-terminal truncated variant Arg333 resembles the CK2α^1-335^ construct commonly used for crystallization and other *in vitro* experiments. This variant is less prone to C-terminal degradation under *in vitro* conditions (Ermakova et al., 2003). Some frameshift mutants may also be an exception to miss-folding/destabilization, as we describe below for CK2β. This indicates that each mutant must be individually studied to address stability and function.

#### A structural perspective of the POBINDS-related CSNK2B mutations

In contrast to the *CSNK2A1* mutations in OCNDS, most mutations of the *CSNK2B* gene linked to POBINDS or related syndromes are frameshift, truncating, and splice site mutations. Missense mutations cluster in highly conserved regions (Figures 2B, 4D; Supplementary Figure S2), including regions important for CK2β dimerization or CK2α subunit interaction and therefore for the assembly of the CK2 holoenzyme (Figure 1). Many of the missense mutations are located at buried residues important for the global architecture of the protein: Phe21Leu, Tyr80Asn, Met97Ile, Arg111Pro, Phe106Val, Cys137Arg/Gly/Phe, Pro164Arg and His165Arg. These missense mutations likely destabilize the protein. Still, there are surface exposed residues in the N-terminal segment (Asp32Asn/Ala/His, Phe34Ser/Cys, Asn35Lys, Arg86Cys), in the acidic loop (Glu77Lys), and in the CK2α interaction site, Leu187Arg/Pro and Gly189Val.

##### Missense mutations in Chantalat’s clusters and N-terminal segment (residues 1-54)

**FIGURE 7:**
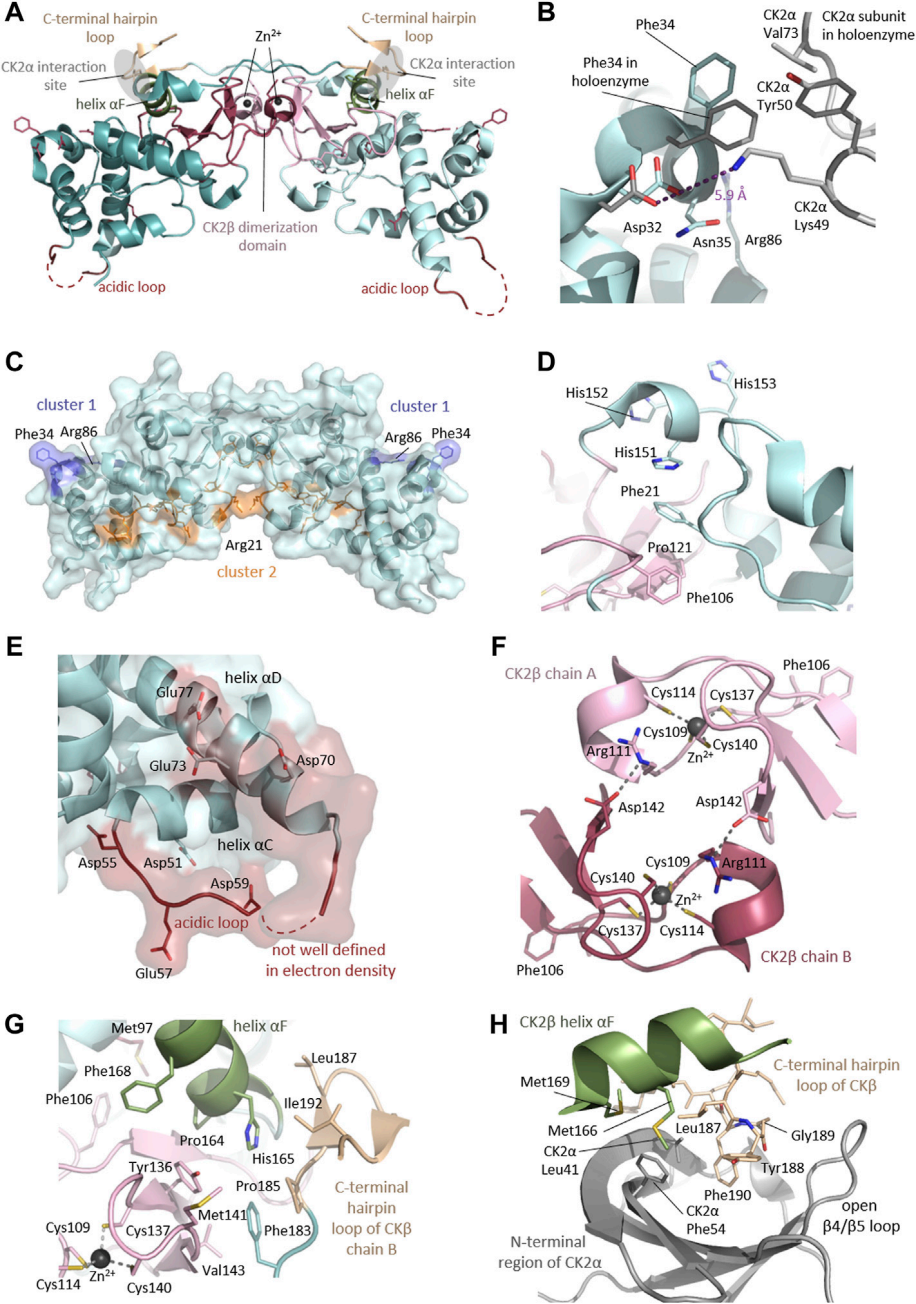
Detailed CK2β functional and structural domains. **(A)** Clustering of mutation sites in structural and functional key elements of the CK2β dimer. The mutated residue side chains are shown as sticks and are coloured according to the different elements as described in the figure. Mutation sites residing outside of the highlighted elements are shown in raspberry red. The CK2β^1-193^ structure (PDB_ID: 3EED (Raaf et al., 2008)) was used for the figure. Parts of chain A of the CK2β^1-193^ dimer, which are not belonging to the highlighted elements, are drawn in light cyan and the dimerization domain is drawn in light pink. Analogously, parts of chain B are drawn in dark cyan and the dimerization domain is drawn in raspberry red. The acidic loops were not defined by electron density and are therefore indicated as dashed lines. **(B)** Mutation sites in the N-terminal region forming a conserved surface exposed cluster. The structure of the CK2β^1-193^ dimer (PDB_ID: 3EED (Raaf et al., 2008)) is drawn in light cyan and the structures of the CK2β and CK2α subunit assembled in the CK2 holoenzyme (PDB_ID: 4DGL (Lolli et al., 2012)) are drawn in grey. The CK2β mutation sites Asp32, Phe34, Asn35 and Arg86 are drawn as sticks, as well as CK2α residues, which are located near the CK2β mutation sites in the assembled CK2 holoenzyme. The distance between CK2β Asp32 and CK2α Lys49 is shown as a purple dashed line to illustrate that the distance is too far to establish a close contact via a salt bridge. **(C)** Conserved surface clusters as described by Chantalat (Chantalat et al., 1999). The first conserved surface exposed cluster is composed of Asp32, Phe34, Asn35 and Arg86 (drawn in blue). The second surface cluster is composed of: Ile10, Glu20, Phe21, Phe22, Cys23, Glu24, Lys100, Asp105, Gly107, Pro110, Tyr144, Pro146, Lys147 and Ser148 (drawn in orange). The side chains of residues Ile10, Cys23, Phe21, Phe22, Pro146 and Tyr144 point into the inside of the protein. The mutation site Phe21 is highlighted. **(D)** Environment of the Chantalat’s cluster 2 mutation site Phe21. Phe21 and His151 are interacting through π-π stacking. The other His-residues of the of the karyophilic cluster (residues 147–153) as well as Pro121 and the mutation site Phe106 are highlighted. **(E)** Mutation site Glu77 residing in the helix αD of the e acidic groove. Further acidic residues are shown as sticks. The acidic loop is indicated as a red dashed line. The CK2β^1-193^ structure (PDB_ID: 3EED (Raaf et al., 2008)) was used for the figure. **(D)** Mutation sites in the dimerization domain of the CK2β^1-193^ dimer (PDB_ID: 3EED (Raaf et al., 2008)). The dimerization domain of CK2β^1-193^ chain A is drawn in light pink and the dimerization domain of CK2β^1-193^ chain B is drawn in raspberry red respectively. The mutation sites Phe106, Arg111 and Cys137, as well as the remaining Cys residues of the zinc binding motif and Asp142, which is involved in an inter-subunit salt bridge with Arg111 of the other CK2β chain are shown as sticks. Zinc ions are drawn as spheres and the interactions between Zn^2+^ and the coordinating Cys residues are indicated as purple dashed lines. The CK2β^1-193^ structure (PDB_ID: 3EED (Raaf et al., 2008)) was used for the figure. **(E)** Mutation sites located inside of CK2β and the contact area to the CK2β chain A body and the CK2β chain B C-terminal tail/hairpin loop. The mutation sites Met97, Phe106, Cys137 and His165 are buried inside of CK2β chain A and are shown as sticks as well as the mutation site Leu187 of the C-terminal hairpin loop of chain B. Further residues of the hydrophobic cluster such as Met141, Val143, Tyr136 and Phe168 of CK2β chain A and Phe183, Pro185 and Ile192 residing in CK2β chain A are shown as sticks, The Cys residues coordinating the Zn^2+^ ion in chain A are shown as sticks as well. The CK2β^1-193^ structure (PDB_ID: 3EED (Raaf et al., 2008)) was used for the figure. **(F)** Mutation sites Leu187 and Gly189 are residing in the subunit interaction interface of CK2β. Critical residues for the subunit interaction of the C-terminal hairpin loop (coloured wheat) of CK2β chain A (Tyr188 and Phe190) and the helix αF (coloured green) of CK2β chain B (Met166 and Met169) surface are shown as sticks the CK2α (Leu41 and Phe54, pale cyan). The N-terminal region of CK2α subunit in the assembled CK2 holoenzyme is shown in grey and residues important for the subunit interaction (Leu41 and Phe54) are shown as sticks. The CK2 holoenzyme structure (PDB_ID: 4DGL (Lolli et al., 2012)) was used for the figure.

In the first published structure of CK2β (CK2β^1-182^), Chantalat et al. described two clusters of exposed, conserved residues (Chantalat et al., 1999). Since CK2β functions as a docking platform, it has been suggested that these conserved surface regions play a role in ligand binding (Chantalat et al., 1999). The first conserved surface cluster is composed of Asp32, Phe34, Asn35 and Arg86 and is directed away from the rest of the protein (Chantalat et al., 1999) (Figures 4C, 7B). Interestingly, the missense mutations of each of the residues (Asp32Asn/Ala/His, Phe34Ser/Cys, Asn35Lys and Arg86Cys) have been linked to neurodevelopmental disability and/or early onset epilepsy in POBINDS and in a new CK2β-linked intellectual disability-craniodigital syndrome (Asif et al., 2022). As discussed above, this could be a novel functional domain of CK2β. One possibility is that this region controls protein stability as Lys33 is found ubiquitinated; therefore, mutations in this region may affect protein half-life (Borgo et al., 2021). It has to be noted that in the CK2 holoenzyme, mutations of this surface cluster will be located in the neighbourhood of the Gly-rich loop and the basic stretch of CK2α—in particular, next to Tyr50, Lys49 and Val73 (Figure 7B). This proximity to the holoenzyme catalytic machinery could mean that mutations in the conserved surface cluster of CK2β may impact the catalytic properties and/or binding of substrate proteins of the CK2 holoenzyme.

The second cluster of conserved surface residues is a helical groove wrapping around the dimer, containing identical residues in 6 species: residues Ile10, Glu20, Phe21, Phe22, Cys23, Glu24, Lys100, Asp105, Gly107, and Pro110 in chain A, and Tyr144, Pro146, Lys147, and Ser148 in chain B of the CK2β dimer (Chantalat et al., 1999) (Figure 7C; Supplementary Figure S2). However, the side chains of residues Ile10, Phe21, Phe22, Cys23, Pro146 and Tyr144 point to the inside of the protein according to their hydrophobic nature. Two of the residues of this groove are reportedly linked to POBINDS: residues 20 (Glu20Ter) and 21 (Phe21Leu). Although the hydrophobic character is retained by the exchange of Phe21 with Leu, the aromatic stacking with His151 can no longer be established Interestingly, His151 is one of the three His residues of the karyophilic cluster (^147^KSSRHHH^153^) proposed to serve as a nuclear localization signal (NLS) (Filhol et al., 2003). Residues adjacent to the ones in this second cluster (Met97Ile, Phe106Val, and Arg111Pro) were found mutated in POBINDS, which may affect the cluster’s structure.

##### Missense mutations in the acidic stretch

CK2β is characterized by an acidic stretch consisting of an acidic loop (Asp55 - Asp70) and an extended acidic groove with residues from helices αA, αC and the N-terminus of helix αD (Chantalat et al., 1999). It is noteworthy that the acidic loop plays a role in the modulation of CK2 activity. It was shown that mutation of residues 55-57 to alanine results in the neutralization of the acidic loop, and hyperactivates CK2 *in vitro* (Boldyreff et al., 1993). Furthermore, the crystal structure of the CK2 holoenzyme showed that crystal contacts between neighbouring CK2 heterotetramers are mediated by the CK2β acidic loop that binds to the positively charged substrate binding region of CK2α (Niefind and Issinger 2005). Among the residues located in helix αD is Glu77 (Figure 7E), which is mutated to Lys in CK2β-related neurodevelopmental disability (Ernst et al., 2021). The replacement with positively charged Lys changes the electrostatics of the acidic stretch and could alter the interactions with other proteins as well as it might affect the formation of oligomeric forms of the CK2 holoenzyme.

##### Missense mutations in the zinc-binding and CK2β dimerization domain

Many mutations in POBINDS patients occur in CK2β’s dimerization region (Figure 7F). Cys137 is one of the four cysteines in CK2β’s zinc finger/dimerization motif (Cys109, Cys114, Cys137 and Cys140). Three different POBINDS-associated mutations at position 137 have been reported (Cys137Arg/Gly/Phe). Lys139 has been proposed as a ubiquitination site as it is an exposed residue (Zhang et al., 2002; French et al., 2007), and we hypothesize that the ubiquitination may serve as a signal to degrade non-dimerized CK2β proteins. Mutation of Cys109 and Cys114 to Ser disrupted CK2β dimerization and CK2 holoenzyme formation *in vitro* and *in vivo* in previous studies (Canton et al., 2001). Arg111 is involved in an inter-subunit salt bridge with Asp142 of the other CK2β chain (Niefind et al., 2001). In addition to the loss of the salt bridge, the Arg111Pro (Li et al., 2019) mutation may lead to a structural rearrangement incompatible with zinc complexation. His165 is a part of the hydrophobic cluster in the dimerization interface (Niefind et al., 2001) (see Figure 7G). Exchange to Arg may induce steric clashes. The neighbouring residue Pro164 resides at the beginning of helix αF and has the function of a helix breaker. The exchange of Pro164 with Arg interferes with function and leads to steric clashes (see Figure 7G).

##### Missense and frameshift mutations in the C-terminal segment (residues: 179-215) and CK2α interaction site

POBINDS-related mutations were found also in the C-terminal region of CK2β harbouring the CK2α interaction site (see Figure 7H). Leu187 and Gly189 are important for CK2α binding and were included in a CK2β-derived cyclic peptide mimicking the highly conserved CK2β hairpin loop essential for binding of CK2α (Laudet et al., 2007). A Gly residue at this position is essential for the close contacts of the hairpin structure, therefore, Leu187/Arg or Pro and Gly189 Val are very likely to disturb the CK2α/CK2β interaction. In this segment, Arg186 is found methylated and Lys191 ubiquitinated, suggesting that mutations in neighbouring residues may affect these two posttranslational modifications (Phosphosite Plus) (Hornbeck et al., 2012).

The Pro179Tyrfs*49 mutation leads to an altered CK2β mutant with a prolonged C-terminus which in co-IP experiments lacks the ability to bind CK2α (Nakashima et al., 2019). Similarly, other frameshift variants with altered sequence of the C-terminal hairpin loop are lacking the main determinant for the subunit interaction and, consequently, may not be able to interact with CK2α.

##### Truncating mutations

The majority of the reported truncating mutations should affect the global fold of CK2β and its dimerization. Exceptions to this are truncating mutations in the C-terminal region. The crystal structure of the C-terminal deletion mutant CK2β^1-182^ showed that the global fold of the protein remains intact (Chantalat et al., 1999). All C-terminal deletion variants with complete loss of C-terminal hairpin loop (main determinant for CK2α interaction) may not interact with CK2α.

## Concluding remarks

We collected these data on *CSNK2A1* and *CSNK2B* variants with the aim to provide a consolidated resource on *CSNK2A1* and *CSNK2B* variants, and analyzed all the mutants using diverse methods to provide integrated information on their potential functional effects that can be used to inform researchers of OCNDS and POBINDS syndromes. We analysed both syndromes together, as the proteins encoded, CK2α and CK2β, can form a functional unit in the cell (protein kinase CK2). We acknowledge that there may be other variants yet to be identified that could modify some of the discussion and conclusions in this study.

Our study has opened additional research questions, including addressing the genetic mechanisms leading to hotspots in *CSNK2A1* SNVs. The ratio of #mutations/#residues could serve to prioritize domains/mutations to study experimentally (e.g., Gly-rich loop in CK2α). Our study indicates that NMC analysis can be used to identify potential novel functional domains in the primary sequence of NDD-associated genes (e.g., N-terminal sequences in CK2α). Our approach in this study, combining NMC analyses with structural analysis (i.e., residues exposed or forming clusters) and evolutionary analyses (highly conserved residues), results in a more solid identification of novel domains that should be further studied experimentally.

A number of key residues involved in substrate recognition are found mutated majorly to a specific amino acid (e.g., Lys198Arg, Arg80His, His160Arg, Arg47Gln and Tyr50Cys). It is possible that mutations in these codons that lead to other amino acids result in embryonic lethality, and thus are not identified in patients. For example, for Lys198 and Arg80 the other potential mutations in the codon are less conservative. It is conceivable that the exchange of these residues involved in substrate recognition to a less conserved residue may result in a defect other than reduced CK2α activity. This hypothesis is supported by the fact that several mutations that strongly interfere with CK2 kinase activity (Dominguez et al., 2021) are present in patients.

For the mutations identified, potential mechanisms of action include: 1) change of substrate specificity but still a strong overlap with canonical CK2 substrates leading to phosphorylation of non-canonical substrates, as is likely the case for Lys198Arg as supported by the literature (Caefer et al., 2022; Werner et al., 2022); 2) change in substrate specificity with no overlap to CK2 canonical substrates (e.g., Lys198 exchange to Thr, Gln, Glu Asn, Ile) which we hypothesize is deleterious as proteins will be phosphorylated in an unregulated way; 3) interference with substrate phosphorylation; 4) the global fold of CK2α is disrupted whenever the amino acid is involved in stabilizing part of the architecture (e.g., Arg312 mutations). We do not expect a loss on the ability to bind substrates, as substrate recognition is not determined by one single residue.

Regarding the correlation of experimental to prediction data for CK2α, we found ranges in the numerical scores from some prediction programs that relate to the mutants with the lowest activity in kinase assays, with the most altered N/C distribution, and with the lowest expression levels. Future work should experimentally address the stability of the mutant proteins. Mutant stability will have implications for treatment; mutants that are destabilizing and degrade rapidly may lead to haploinsufficient phenotypes.

This study utilized prediction programs that analyze missense mutants. Nonsense, frameshift and splice variants that are also found in CK2α and CK2β are typically deemed as *de facto* loss-of-function. Determining the impact of these types of mutants is complex as the prediction programs are still under development (Abrahams et al., 2021; Lord and Baralle 2021). For CK2α and CK2β, it will be key to develop predictions programs that include computing of more complex characteristics that can be affected by changes in the primary structure (e.g., binding to each other or other proteins, post-translational modifications, subcellular localization or the formation of oligomeric forms that inhibit activity (Glover 1986; Valero et al., 1995; Borgo et al., 2021; Roffey and Litchfield 2021). The NMC analysis assumes that each residue has an independent and equal likelihood of mutation (i.e., homogeneous mutation probability across all base positions), and calculates the probability that mutations (or clusters of mutations) are non-random. Both population and gene characteristics can affect site mutability. Published studies estimate the genome-wide average mutation rate at ∼1–1.5 × 10^−8^ mutations per base pair per generation (Carlson et al., 2018), and that *de novo* missense mutations in patients with neurodevelopmental diseases are not randomly distributed, and cluster in fewer genes compared to controls (The Deciphering Developmental Disorders Study 2015). A potential explanation is that for mutations associated with neurodevelopmental disease, embryonic lethality will impact on the variants that are found in patients. This will result in a non-homogeneous distribution of patient mutations.

Genotype data for many of the patients included in this study is absent or partial. However, Wu et al. indicate that mutations in protein-coding CK2α regions appear to influence the phenotypic spectrum of OCNDS. In particular mutations residing in the Gly-rich loop were more likely to cause the widest range of phenotypes (Wu et al., 2021). For POBINDS, Bonanni et al. very recently reviewed the available literature, where they correlate the neurodevelopmental abnormalities to epilepsy severity and highlight the heterogeneity of the clinical phenotypes that have been associated with the CSNK2B gene (Bonanni et al., 2021). A recent article has reviewed both diseases (Ballardin et al., 2022). Broader phenotype-genotype correlation data will help us understand the severity of the diseases and may lead to a specific differential diagnosis to clearly identify OCNDS and POBINDS patients. These types of correlations will help us understand other diseases were these genes are mutated or functionally affected (Castello et al., 2017; Colavito et al., 2018).

Further experimental data is necessary to determine the impact (if any) of each identified mutation in protein structure and biochemistry, subcellular localization and molecular mechanism of disease in embryonic development and in adulthood, which will lead to insights to the severity and nature of reported symptoms. *CSNK2A1* and *CSNK2B* variants could lead to mutants with reduced or increased activity, or dominant negative or gain of function effects. Defining these effects will help indicate therapeutic approach strategies that may be needed.

## Data Availability

The original contributions presented in the study are included in the article/Supplementary Material, further inquiries can be directed to the corresponding author.

## Supplementary material

Supplementary Figure 1 Alignment showing conservation in CK2a mutated residues across eukaryotic species.

Supplementary Figure 2 General statistics about orthologous gene groups.

Supplementary Table 2 General statistics about orthologous gene groups.

Supplementary Table 2 General statistics about orthologous gene groups.
