# Supplementary material for: Predictive functional, statistical and structural analysis of CSNK2A1 and CSNK2B variants linked to neurodevelopmental diseases
Source: Front Mol Biosci. 2022 Oct 13;9:851547. doi: 10.3389/fmolb.2022.851547 (PMC9608649; doi:10.3389/fmolb.2022.851547)
Supplement: Supplementary file 1 [file DataSheet2.PDF]

Alignment showing conservation in CK2β mutated residues across eukaryotic species. Alignment was performed using MUSCLE (HTML format). Residues in Blue are mostly conserved across species, somehow conserved in gray and not conserved in white. The human sequence is bolded and the residues mutated are marked in red. Chatalat et al. 1999 conserved residues are highlighted in the *C. elegans*'s sequence (pink): pink bold= 40 conserved residues across species and black bold= conserved residues in some species. In the *S. Cerevisiae* sequence, additional residues conserved across species identified in his study (pink bold) = additional conserved residues identified here. In the *G. gallus* sequence (orange): bold = cluster 1, and bold orange=cluster 2. A revision of the 3D structure of the groove shows that residues Ser11, Arg17, Glu20, Glu24, Asp26, Glu27, Asp28, Gln31, Arg92, Gln96, Glu99, Lys100, Gln103, Asp105, Gly107, Tyr108, Val112, Lys147, Ser148, Ser149, Arg150 and His152 are solvent-exposed.

```
MUSCLE alignment. HTML format

gi|6324613|ref|NP_014682.1|          -----------------------------------------------------------m
gi|50309855|ref|XP_454941.1|         ---------------------------------------------------------Msi
gi|45185773|ref|NP_983489.1|         ------------------------------------------------------------
gi|19115518|ref|NP_594606.1|         ------------------------------------------------------------
gi|389625781|ref|XP_003710544.1|     ------------------------------------------------------------
gi|164427743|ref|XP_001728406.1|     ------------------------------------------------------------
gi|15224934|ref|NP_181996.1|         mykdrsgggimggggssrseilggaidrkrindaldkhlkksspstsrvftskdkdsVps
gi|17508229|ref|NP_492254.1|         ------------------------------------------------------------
gi|45554910|ref|NP_996415.1|         ------------------------------------------------------------
gi|18858421|ref|NP_571262.1|         ------------------------------------------------------------
gi|109070470|ref|XP_001112540.1|     ------------------------------------------------------------
NP_001084126.1                       ------------------------------------------------------------
gi|545518516|ref|XP_003431700.2|     ------------------------------------------------------------
gi|23503295|ref|NP_001311.3|         ------------------------------------------------------------
gi|410040528|ref|XP_003950832.1|     ------------------------------------------------------------
gi|114326228|ref|NP_001039919.1|     ------------------------------------------------------------
gi|7106277|ref|NP_034105.1|          ------------------------------------------------------------
gi|78214347|ref|NP_001030315.1|      ------------------------------------------------------------
NP_001244133.1                       ------------------------------------------------------------

gi|6324613|ref|NP_014682.1|          gSrSenvgtvtregsrveqddvlmDddsdSSEyVdmWIdlFlGrkGhEyFCdVDpeYItD
gi|50309855|ref|XP_454941.1|         peESvetteqpdvvmadalvvndqDdgsdSgEyVeYWIdlFlGkkGhEyFCdiDteYItD
gi|45185773|ref|NP_983489.1|         mSErrleehgtaetadvemadavetnStgSSdyVelWIdlFlGkkGhEyFCdVDteYItD
gi|19115518|ref|NP_594606.1|         ---------------------mqlysSeSeSdDsqYWVdWFlGLkGNEFFCEVDEDfIQD
gi|389625781|ref|XP_003710544.1|     ---------------------medfgSeSdSdytSYWrdWFiGskGNEyFCEiDEDYltD
gi|164427743|ref|XP_001728406.1|     ---------------------mddfvSeSeSdyaSYWrdWFissRGNEyFCEiDEDYItD
gi|15224934|ref|NP_181996.1|         tStAksqlhsrspdvesdtdsegsDvSgSegdDtS-WISWFCnLRGNEFFCEVDEDYIQD
gi|17508229|ref|NP_492254.1|         ---------------------------MSSSEEVS-WItWFCGLRGNEFFCEVDEeYIQD
gi|45554910|ref|NP_996415.1|         ---------------------------MSSSEEVS-WVtWFCGLRGNEFFCEVDEDYIQD
gi|18858421|ref|NP_571262.1|         ---------------------------MSSSEEVS-WISWFCGLRGNEFFCEVDEDYIQD
gi|109070470|ref|XP_001112540.1|     --------mekckgtsrmagttsaDvkMSSSEEVS-WISWFCGLRGNEFFCEVDEDYIQD
NP_001084126.1                       ---------------------------MSSSEEVS-WISWFCGLRGNEFFCEVDEDYIQD
gi|545518516|ref|XP_003431700.2|     -------mekckgtssrmagatsaDvkMSSSEEVS-WISWFCGLRGNEFFCEVDEDYIQD
gi|23503295|ref|NP_001311.3|         ---------------------------MSSSEEVS-WISWFCGLRGNEFFCEVDEDYIQD
gi|410040528|ref|XP_003950832.1|     ---------------------------MSSSEEVS-WISWFCGLRGNEFFCEVDEDYIQD
gi|114326228|ref|NP_001039919.1|     ---------------------------MSSSEEVS-WISWFCGLRGNEFFCEVDEDYIQD
gi|7106277|ref|NP_034105.1|          ---------------------------MSSSEEVS-WISWFCGLRGNEFFCEVDEDYIQD
gi|78214347|ref|NP_001030315.1|      ---------------------------MSSSEEVS-WISWFCGLRGNEFFCEVDEDYIQD
NP_001244133.1                       ---------------------------MSSSEEVS-WISWFCGLRGNEFFCEVDEDYIQD
                                                                M1  E5    W9        E20-F21  E27 Q31-D32

gi|6324613|ref|NP_014682.1|          rFNLmnLqktVskfsyvvqyIvD-dldD--siLEnMtharleqlEsdsrkLYGLIHARYI
gi|50309855|ref|XP_454941.1|         rFNLinLqktVskfttvvqymvD-dlee--giiEsMsParleqlEtdtrkLYGLIHARYI
gi|45185773|ref|NP_983489.1|         rFNLinLqktVskftQviqymvD-eldD--ttLEaMsrtkltqlEadArkLYGLIHARYI
gi|19115518|ref|NP_594606.1|         rFNLTGLsheVPHYsQsLDlILDvLDPDlpEEvqD-------eVEasArhLYGLIHARYI
gi|389625781|ref|XP_003710544.1|     rFNLTGLNteVsyYqyALDlItD-vf-----DLdc-dddmretIEksArhLYGLvHARYI
gi|164427743|ref|XP_001728406.1|     rFNLTGLNteVqyYqyALDlItD-vf-----DLdc-dddmretIEksArhLYGLvHARYI
gi|15224934|ref|NP_181996.1|         dFNLcGLsgQVPyYdyALDlILD-vEss--ngdmf-teeQheMVEsAAEMLYGLIHvRYI
gi|17508229|ref|NP_492254.1|         rFNLTGLNEQVPkYRQALDMILD-LEPe--dDiED-NatnTDLVEQAAEMLYGLIHARYI
gi|45554910|ref|NP_996415.1|         KFNLTGLNEQVPnYRQALDMILD-LEPe--dELED-NPlQSDMtEQAAEMLYGLIHARYI
gi|18858421|ref|NP_571262.1|         KFNLTGLNEQVPHYRQALDMILD-LEPD--EELED-NPNQSDLIEQAAEMLYGLIHARYI
gi|109070470|ref|XP_001112540.1|     KFNLTGLNEQVPHYRQALDMILD-LEPD--EELED-NPNQSDLIEQAAEMLYGLIHARYI
NP_001084126.1                       KFNLTGLNEQVPHYRQALDMILD-LEPD--EELED-NPNQSDLIEQAAEMLYGLIHARYI
gi|545518516|ref|XP_003431700.2|     KFNLTGLNEQVPHYRQALDMILD-LEPD--EELED-NPNQSDLIEQAAEMLYGLIHARYI
gi|23503295|ref|NP_001311.3|         KFNLTGLNEQVPHYRQALDMILD-LEPD--EELED-NPNQSDLIEQAAEMLYGLIHARYI
gi|410040528|ref|XP_003950832.1|     KFNLTGLNEQVPHYRQALDMILD-LEPD--EELED-NPNQSDLIEQAAEMLYGLIHARYI
gi|114326228|ref|NP_001039919.1|     KFNLTGLNEQVPHYRQALDMILD-LEPD--EELED-NPNQSDLIEQAAEMLYGLIHARYI
gi|7106277|ref|NP_034105.1|          KFNLTGLNEQVPHYRQALDMILD-LEPD--EELED-NPNQSDLIEQAAEMLYGLIHARYI
gi|78214347|ref|NP_001030315.1|      KFNLTGLNEQVPHYRQALDMILD-LEPD--EELED-NPNQSDLIEQAAEMLYGLIHARYI
NP_001244133.1                       KFNLTGLNEQVPHYRQALDMILD-LEPD--EELED-NPNQSDLIEQAAEMLYGLIHARYI
                                  F34-N35 T37  Q42  R47       E57   E61           E77 Y80 L82 R86 I88

gi|6324613|ref|NP_014682.1|          iTikGlqkMyaKYkEaDFGrCPRVYCnlQqlLPVGLhDIPGidcVKLYCPsCeDlYiPKS
gi|50309855|ref|XP_454941.1|         iTakGlqkMLqKYkaaDFGrCPRyhCnhQPlLPVGLhDIPGidcVKLYCPsCeDlYnPKS
gi|45185773|ref|NP_983489.1|         iTikGlqkMLqKYrdaDFcrCPRVhCnfQPlLPVGLhDvPGidcVKLYCPsCeDlYiPKS
```

```
gi|19115518|ref|NP_594606.1|          LTaqGlykMLEKYkkcDFGHCPRVlCngQPMLPVGLSDIahtksVKLYCPrCeDVYTPKS
gi|389625781|ref|XP_003710544.1|      vTtRGlskMLEKYkkGDFGkCPRVhCgshPlLPmGmcDIPsskpVKLYCarCeDIYnPKS
gi|164427743|ref|XP_001728406.1|      vTtRGlqkMfEKYkkaDFGkCPRVmCsshPlLPmGLSDvPnskpVKLYCarCeDIYnPKS
gi|15224934|ref|NP_181996.1|          LTtkGmAaMmEKYknyDFGrCPRVfCcgQscLPVGqSDIPrsstVKiYCPKCeDIYyPrS
gi|17508229|ref|NP_492254.1|          LTNRGIsQMvEKwrdhDFGvCPRVYCENQPMLPIGLSDvPGEAMVKLYCPrCnDVfvPrS
gi|45554910|ref|NP_996415.1|          LTNRGIAQMiEKYQtGDFGHCPRVYCEsQPMLPlGLSDIPGEAMVKtYCPKCIDVYTPKS
gi|18858421|ref|NP_571262.1|          LTNRGIAQMLEKYQQGDFGYCPRVYCENQPMLPIGLSDIPGEAMVKLYCPKCMDVYTPKS
gi|109070470|ref|XP_001112540.1|      LTNRGIAQMLEKYQQGDFGYCPRVYCENQPMLPIGLSDIPGEAMVKLYCPKCMDVYTPKS
NP_001084126.1                        LTNRGIAQMLEKYQQGDFGYCPRVYCENQPMLPIGLSDIPGEAMVKLYCPKCMDVYTPKS
gi|545518516|ref|XP_003431700.2|      LTNRGIAQMLEKYQQGDFGYCPRVYCENQPMLPIGLSDIPGEAMVKLYCPKCMDVYTPKS
gi|23503295|ref|NP_001311.3|          LTNRGIAQMLEKYQQGDFGYCPRVYCENQPMLPIGLSDIPGEAMVKLYCPKCMDVYTPKS
gi|410040528|ref|XP_003950832.1|      LTNRGIAQMLEKYQQGDFGYCPRVYCENQPMLPIGLSDIPGEAMVKLYCPKCMDVYTPKS
gi|114326228|ref|NP_001039919.1|      LTNRGIAQMLEKYQQGDFGYCPRVYCENQPMLPIGLSDIPGEAMVKLYCPKCMDVYTPKS
gi|7106277|ref|NP_034105.1|           LTNRGIAQMLEKYQQGDFGYCPRVYCENQPMLPIGLSDIPGEAMVKLYCPKCMDVYTPKS
gi|78214347|ref|NP_001030315.1|       LTNRGIAQMLEKYQQGDFGYCPRVYCENQPMLPIGLSDIPGEAMVKLYCPKCMDVYTPKS
NP_001244133.1                        LTNRGIAQMLEKYQQGDFGYCPRVYCENQPMLPIGLSDIPGEAMVKLYCPKCMDVYTPKS
                                       T90    M97 Y101 F106 R111  Q117          M132 C137

gi|6324613|ref|NP_014682.1|           SRHssiDGAYFGTsFPgMflqafPdmvPKhPtkryVPkifGFelHkqAqltr--------
gi|50309855|ref|XP_454941.1|          SRHsaiDGAYFGTsFPgMflqgfPEmcPKhPtkryVPkvfGFdlHkqAqltr--------
gi|45185773|ref|NP_983489.1|          SRHssiDGAYFGTsFPgMflqafPEvvPKhPtkryVPkvfGFdlHkqAqlar--------
gi|19115518|ref|NP_594606.1|          qRHasiDGAYFGTsFPHMLFqVyPElavpksqeryiPRifGFKvHsysatfk--------
gi|389625781|ref|XP_003710544.1|      SRHagiDGAYFGTsFhniLFqVyPalmPvksAeryVPRcfGFrvHaaAslVr--------
gi|164427743|ref|XP_001728406.1|      SRHaaiDGAYFGTsFhnifFqVyPtlvPaksveryiPRcYGFKvHaaAalVr--------
gi|15224934|ref|NP_181996.1|          kyqgniDGAYFGTtFPHlflMaygnmKPqkPAqnyVPkifGFKvHnkq------------
gi|17508229|ref|NP_492254.1|          SRHqHTDGsYFGTGFPHMLFfVHPdlRPrRPvtQFVPkLYGFKIHPvAYggQegnsggnt
gi|45554910|ref|NP_996415.1|          SRHHHTDGAYFGTGFPHMLFMVHPEYRPKRPtNQFVPRLYGFKIHsLAYQIQ--------
gi|18858421|ref|NP_571262.1|          SRHHHTDGAYFGTGFPHMLFMVHPEYRPKRPANQFVPRLYGFKIHPMAYQLQ--------
gi|109070470|ref|XP_001112540.1|      SRHHHTDGAYFGTGFPHMLFMVHPEYRPKRPANQFVPRLYGFKIHPMAYQLQ--------
NP_001084126.1                        SRHHHTDGAYFGTGFPHMLFMVHPEYRPKRPANQFVPRLYGFKIHPMAYQLQ--------
gi|545518516|ref|XP_003431700.2|      SRHHHTDGAYFGTGFPHMLFMVHPEYRPKRPANQFVPRLYGFKIHPMAYQLQ--------
gi|23503295|ref|NP_001311.3|          SRHHHTDGAYFGTGFPHMLFMVHPEYRPKRPANQFVPRLYGFKIHPMAYQLQ--------
gi|410040528|ref|XP_003950832.1|      SRHHHTDGAYFGTGFPHMLFMVHPEYRPKRPANQFVPRLYGFKIHPMAYQLQ--------
gi|114326228|ref|NP_001039919.1|      SRHHHTDGAYFGTGFPHMLFMVHPEYRPKRPANQFVPRLYGFKIHPMAYQLQ--------
gi|7106277|ref|NP_034105.1|           SRHHHTDGAYFGTGFPHMLFMVHPEYRPKRPANQFVPRLYGFKIHPMAYQLQ--------
gi|78214347|ref|NP_001030315.1|       SRHHHTDGAYFGTGFPHMLFMVHPEYRPKRPANQFVPRLYGFKIHPMAYQLQ--------
NP_001244133.1                        SRHHHTDGAYFGTGFPHMLFMVHPEYRPKRPANQFVPRLYGFKIHPMAYQLQ--------
                                             Y158 P164-H165 L167  P179 N181  R186-L187-Y188-G189

gi|6324613|ref|NP_014682.1|           ---wQel----------------qrlKlveKleskDvDltksgGfkt--------------
gi|50309855|ref|XP_454941.1|          ---wQel----------------qrlKlekllItkgiNlNEsgGyQf--------------
gi|45185773|ref|NP_983489.1|          ---wQel----------------qrlKlerelIKkgvDltkngGykk--------------
gi|19115518|ref|NP_594606.1|          ---kQdv----------------ykeKqkkRlqgaEaEsknK-----------------lai
gi|389625781|ref|XP_003710544.1|      ---wQsgRREDMRRRLRKMEVESgFKdgadeIedDeEeDEEeddEdgmkqegmavglvds
gi|164427743|ref|XP_001728406.1|      ---wQnsQRDEMRRRLRKLEVESgFKdaedeaelDdDdEEEeeeEeeeeelaamdeaega
gi|15224934|ref|NP_181996.1|          ------------------------------------------------------------
gi|17508229|ref|NP_492254.1|          annvaAA----------------qNntTPagqqsggqfnNygl------------------
gi|45554910|ref|NP_996415.1|          ---LQAA----------------aNFKmPLRaqRgqppkDEEpennadtvpkrl-------
gi|18858421|ref|NP_571262.1|          ---LQAA----------------SsFKSPVKaIR---------------------------
gi|109070470|ref|XP_001112540.1|      ---LQAA----------------SNFKSPVKTIR---------------------------
NP_001084126.1                        ---LQAA----------------SNFKSPVKTMR---------------------------
gi|545518516|ref|XP_003431700.2|      ---LQAA----------------SNFKSPVKTIR---------------------------
gi|23503295|ref|NP_001311.3|          ---LQAA----------------SNFKSPVKTIR---------------------------
gi|410040528|ref|XP_003950832.1|      ---LQAA----------------SNFKSPVKTIR---------------------------
gi|114326228|ref|NP_001039919.1|      ---LQAA----------------SNFKSPVKTIR---------------------------
gi|7106277|ref|NP_034105.1|           ---LQAA----------------SNFKSPVKTIR---------------------------
gi|78214347|ref|NP_001030315.1|       ---LQAA----------------SNFKSPVKTIR---------------------------
NP_001244133.1                        ---LQAA----------------SNFKSPVKTIR---------------------------
                                                              F207

gi|6324613|ref|NP_014682.1|           ------------------------
gi|50309855|ref|XP_454941.1|          ------------------------
gi|45185773|ref|NP_983489.1|          ------------------------
gi|19115518|ref|NP_594606.1|          t-----------------------
gi|389625781|ref|XP_003710544.1|      atvpgdSmmqgv------------
gi|164427743|ref|XP_001728406.1|      qqqhaaAaagtatggvaaggegvh
gi|15224934|ref|NP_181996.1|          ------------------------
gi|17508229|ref|NP_492254.1|          ------------------------
gi|45554910|ref|NP_996415.1|          ------------------------
gi|18858421|ref|NP_571262.1|          ------------------------
gi|109070470|ref|XP_001112540.1|      ------------------------
NP_001084126.1                        ------------------------
gi|545518516|ref|XP_003431700.2|      ------------------------
gi|23503295|ref|NP_001311.3|          ------------------------
gi|410040528|ref|XP_003950832.1|      ------------------------
gi|114326228|ref|NP_001039919.1|      ------------------------
gi|7106277|ref|NP_034105.1|           ------------------------
gi|78214347|ref|NP_001030315.1|       ------------------------
NP_001244133.1                        ------------------------
```

**Accession**	**Organism**	**Common name or disease**	**Classification**
gi|6324613|ref|NP_014682.1|	Saccharomyces cerevisiae	Baker's yeast	Yeast and Fungi
gi|50309855|ref|XP_454941.1|	Kluyveromyces lactis	Milk yeast	
gi|45185773|ref|NP_983489.1|	Eremothecium gossypii	Filamentous fungus	
gi|19115518|ref|NP_594606.1|	Schizosaccharomyces pombe	Fission yeast	
gi|389625781|ref|XP_003710544.1|	Pyricularia oryzae	Rice blast disease fungus	
gi|164427743|ref|XP_001728406.1|	Neurospora crassa	Red bread mold	
gi|15224934|ref|NP_181996.1|	Arabidopsis thaliana	Thale cress	Plant
gi|17508229|ref|NP_492254.1|	Caenorhabditis elegans	Nematode roundworm	Invertebrates
gi|45554910|ref|NP_996415.1|	Drosophila melanogaster	Fruit fly	
gi|18858421|ref|NP_571262.1|	Danio rerio	Zebrafish	Vertebrates
gi|109070470|ref|XP_001112540.1|	Macaca mulatta	Rhesus monkey	
NP_001084126.1	Xenopus laevis	African claw-toed frog	
gi|545518516|ref|XP_003431700.2|	Canis lupus familiaris	Domestic dog	
**gi|23503295|ref|NP_001311.3|**	**Homo sapiens**	Human	
gi|410040528|ref|XP_003950832.1|	Pan troglodytes	Chimpanzee	
gi|114326228|ref|NP_001039919.1|	Bos taurus	Cow	
gi|7106277|ref|NP_034105.1|	Mus musculus	Mice	
gi|78214347|ref|NP_001030315.1|	Rattus norvegicus	Rat	
NP_001244133.1	Gallus gallus	Chicken	
