# Supplementary material for: Predictive functional, statistical and structural analysis of CSNK2A1 and CSNK2B variants linked to neurodevelopmental diseases
Source: Front Mol Biosci. 2022 Oct 13;9:851547. doi: 10.3389/fmolb.2022.851547 (PMC9608649; doi:10.3389/fmolb.2022.851547)
Supplement: Supplementary file 3 [file DataSheet4.PDF]

ConSurf Color-Coded MSA

001 Input_pdb_SEQRES_A	MSSSEEVSWISWFCGLRGNEFF------CEVDEDYIQDKFNLTGLNEQV-
002 UniRef90_D3PJV2_1_193	MSSSEEVSWISWFCGLRGNEFF------CEVDEDYIQDKFNLTGLNEQV-
003 UniRef90_A0A443SMC7_9_201	MSSSEEVSWISWFCGLRGNEFF------CEVDEDYIQDKFNLTGLSESV-
004 UniRef90_A0A7R8WGP2_1_193	MSSSEEVSWITWFCGLRGNEYF------CEVDEDYIQDKFNLTGLNEQV-
005 UniRef90_A0A7R9KER7_1_193	MSSSEEVSWISWFCGLRGNEYF------CEVDEDYIHDKFNLTGLSESV-
006 UniRef90_K7FHH3_1_194	MSSSEEVSWISWFCGLRGNEFARLP--PPQVDEDYIQDKFNLTGLNEQV-
007 UniRef90_A0A1I8ISK0_1_192	MSSSEEVSWISWFCGLRGNEFF------CEVDEDYIQDKFNLTGLNEQV-
008 UniRef90_A0A6P9F8P8_1_185	MSNSEEVSWISWFCGLRGNEFF------CEVAEDYIQDKFNLTGLNEQV-
009 UniRef90_A0A315WGG2_1_182	MSSSEEVSWISWFCGLRGNELF------CEA--NYIQDKFNLTGLNEQV-
010 UniRef90_A0A0B2UX36_1_192	MSSSEEISWISWFCGLRGNEFF------CEVDEDYITDKFNLTGLNEQV-
011 UniRef90_A0A210QLV3_15_188	-------------------QLV------MHVDEDYIQDKFNLTGLNEQV-
012 UniRef90_A0A6A4WWH4_1_175	MSSSEEVSWISWFCGLRGNEFF------CEVDEDYIQDKFNLTGLNEQV-
013 UniRef90_A0A673UYT7_1_167	MSSSEEVSWISWFCGLRGNEFF------CEVDEDYIQDKFNLTGLNEQV-
014 UniRef90_A0A090MVV2_1_191	MSSSEEVSWVTWFCGLRGNDFF------CEVDEDYIQDRFNLTGLSEQV-
015 UniRef90_A0A0N5AZ67_43_214	---------------------F------LKVEEEYIQDKFNLTGLNEQV-
016 UniRef90_A0A0D2U6T3_2_190	--SSDEISWITWFCNLRGNEFF------CEVDEEWIQDKFNLTGLAEMV-
017 UniRef90_A0A0N4UWY4_7_187	-----------------------LGSVACAVDEDYINDRFNLTGLSEQV-
018 UniRef90_A0A1W4V542_1_191	MTDSDETAWIHWFCKQRGNEFF------CEVDEDYIQDKFNLNFLDSNV-
019 UniRef90_A0A6P4JVR0_1_191	MTDSDDTAWIHWFCKQRGNEFF------CEVEEDYIHDKFNLNFLDSNV-
020 UniRef90_A0A5F8HIB5_36_187	-----------------------------EVDEDYIQDKFNLPGLNEQV-
021 UniRef90_A0A7S4IQT0_22_213	-ASDEDGSWISWFCSLRGNEFF------CEVDEDYIQDDFNLTGLNGLV-
022 UniRef90_A0A6J0MJ32_91_282	-SEGEDTSWISWFCNLRGNEFF------CEVDDDYIQDDFNLCGLNHQV-
023 UniRef90_A0A388JRV3_116_307	-SDEEDTSWISWFCGLKGNEFF------CEVDDEYIQDDFNLSGLSSQV-
024 UniRef90_A0A0D6QXG8_112_303	-SDADDTSWISWFCNLRGNEFF------CEVDDEYIQDDFNLCGLSSQV-
025 UniRef90_A0A151ZRX5_21_208	---EDEMAWIPWYCSLKGNEFF------ATIDEDYIQDDFNLTGLSSLV-
026 UniRef90_UPI0019298C3E_89_278	---VDDTSWISWFCNLRGNEFF------CEVDDEYIQDDFNLCGLSSQV-
027 UniRef90_A0A7S3PWQ0_98_288	-SASDDGSWISWFCSLRGNEFF------CEVDEDYIQDDFNLTGLHLLV-
028 UniRef90_A0A6I8V063_1_191	MTDSDETGWIQWFCKQRGNEFF------TFVDEEYIRDKFNLMFLDTEL-
029 UniRef90_A0A250WT55_67_256	---EEEPSWIQWFCSLRGNEFF------CEVDEDYIEDDFNLSGLSSQV-
030 UniRef90_A0A6P3Z3J9_56_245	--DGDDTSWISWFCNLRGNEFF------CEVDDDYIQDDFNLCGLSSQV-
031 UniRef90_M4CKX0_70_259	---DEDTSWISWFCNLRGNEFF------CEVDEDYVQDDFNLCGLSGLV-
032 UniRef90_A0A1U8N6I0_93_282	--DGDDTSWISWFCNLRGNEFF------CEVDDDYIQDDFNLCGLSSQV-
033 UniRef90_A0A1U8N824_86_276	--DGDDTSWISWFCNLRGNEFF------CEVDDAYIQDDFNLCGLTSQV-
034 UniRef90_A0A0D2QNY2_22_212	--DGDDTSWISWFCNLRGNEFF------CEVDDDYIQDDFNLCGLSSQV-
035 UniRef90_UPI0007EFC624_83_271	----VDSSWISWFCNLRGNEFF------CEVDEDYIQDDFNLCGLSSQV-
036 UniRef90_A0A6J0MMZ8_88_277	---DDDTSWISWFCNLRGNEFF------CQVDEDYVEDDFNLCGLSAQV-
037 UniRef90_A0A2I0X454_94_284	--DGDDTTWISWFCNIRGNELF------CEIDDEYIQDDFNLCGLSSQV-
038 UniRef90_UPI00092FBD2C_80_270	--GEDDTSWISWFCNLRGNEFF------CEVDDDYIRDDFNLSGLSTQV-
039 UniRef90_F0ZXH6_18_205	---EDEMAWIPWYCNLKGNEFF------ATIDEDYINDDFNLTGLSSLV-
040 UniRef90_A0A1U8IV83_84_274	--EGDDTSWISWFCGLRGNEFF------CEVDDEYIQDDFNLCGLSSQV-
041 UniRef90_A0A261XW74_26_208	-------YWIDWFLNTKGNEYF------CEVDEEYILDRFNLTGLNTEV-
042 UniRef90_A0A162PQW6_12_196	-------YWIDWFLGTKGNEYF------CEVDEEYILDRFNLTGLNVEV-
043 UniRef90_A0A7S3CG89_21_212	-SASEETSWVNWFCRLQGNEFF------VEVDEDYIQDEFNLCGLNQQV-
044 UniRef90_A0A162UUP8_10_199	--SEYVKYWIDWFLGIKGNEYF------CEIDEEYILDRFNLTGLNAEV-
045 UniRef90_A0A7R9Y293_54_246	-SEEEDTSWISWFCSLKGNEFF------CEVDSEYIEDDFNLSGLSSQV-
046 UniRef90_UPI00142E405F_77_267	---GSDTLWVSWFCSLRGNEFF------CEVDDDYIQYDFNLCGLSSLV-
047 UniRef90_A0A0K9Q617_79_270	-SDEEETSWISWFCNLRGNEFF------CEVDDEYIQDDFNLCGLSRQV-
048 UniRef90_A0A6P5GZT7_89_278	--EGDDSSWISWFCGLRGNEFL------CEVDDDYIQDDFNLCGLQGQV-
049 UniRef90_A0A1R1PTJ3_11_196	-------YWVEWFLNTKGNEFF------CEIDDEYILDRFNLTGLHAEV-
050 UniRef90_A0A7J7KUM0_133_320	----YDSSWISWYCGLRGNELL------CEVDEDYIQDDFNLCGLQGQV-
051 UniRef90_F0W6R1_98_288	--SGEETSWITWFCSLRENDFL------CEVDEEYIEDDFNLTGLSGIV-
052 UniRef90_A0A4P9XR61_13_199	-----QKYWVDWFLSLRGNEYF------CEVDEEYILDRFNLTGLSAEV-
053 UniRef90_A0A7S0ES88_51_226	---NEGLSWIQWFCGLQGNEFF------TEVAEEFVQDDFNLTGLGSQV-
054 UniRef90_A0A420Y4K1_15_197	-------YWRDWFISSRGNEYF------CEIDEDYLTDRFNLTGLNAEV-
055 UniRef90_A4RSN9_26_212	----TEQSWIAWFCGLKGNELF------CEVDEEYIQDDFNLSGLSSSV-
056 UniRef90_A0A642UK79_17_202	----SEDMWVDWFLGTKGNEYF------CDIDTEFITDRFNLTGLNTEV-
057 UniRef90_A0A1R3S306_15_197	-------YWRDWFISSRGNEYF------CEIDEDYLTDRFNLTGLNTEV-
058 UniRef90_A0A183REZ2_109_233	--
059 UniRef90_A0A067N327_22_192	-----SNSWISWFLSSKGNEYF------CEVEEDYILDRFNLTGLNTEV-
060 UniRef90_A0A507CIU8_18_202	-----GKYWSEWFSQQKGNEFF------CEVDEEYILDRFNLTGLNAEV-

```
061 UniRef90_A0A7D8Z2K1_14_184      ------NTWIAWFLSTKGNEYF------AEVDEDYILDRFNLTGLNGEVV
062 UniRef90_A0A0F4ZAU1_16_198      -------YWRDWFISMRGHEYF------CEIDEDYLTDRFNLTGLNTEV-
063 UniRef90_A0A7S1VF37_12_200      ---SSDSPWIQWYCSLVGNEFF------CEVDEDFIRDNFNLTGLSAQV-
064 UniRef90_A0A0L0H4P4_15_200      ----STAYWIEWFQSLKGNEFY------CDVDEDYILDRFNLTGLNTEV-
065 UniRef90_A0A6H0Y5P8_15_197      -------YWRDWFIAARGNEYY------CEIDEDYLTDRFNLTGLNTEV-
066 UniRef90_A0A7R9BIZ0_390_519     --------------------------------------------------
067 UniRef90_A0A3E2HRT2_15_197      -------YWRDWFISSRGNEYF------CEIDEEYLTDRFNLTGLNTEV-
068 UniRef90_A0A6A6V4N0_15_197      -------YWRDWFISSRGNEYF------CEIDEEYLTDRFNLTGLNTEV-
069 UniRef90_A0A6A6YKN2_15_197      -------YWRDWFISSRGNEYF------CEIDEEYLTDRFNLTGLNTEV-
070 UniRef90_A0A2J5HQ92_16_198      -------YWRDWFISSRGNEYF------CEIDEEYLTDRFNLTGLNTEV-
071 UniRef90_A0A0J9XL38_11_196      ----SGEFWIDWFLTTKGNEYF------CDIDPEFITDRFNLTGLNTDV-
072 UniRef90_A0A2D3V5L0_22_206      -----WLSFSDMFISSRGNEYF------CEIDEDYLTDRFNLTGLNTEV-
073 UniRef90_M5BRL5_5_172           -------SWVSWFLSSKGNEYF------CEVEEDYILDRFNLTGLNSEV-
074 UniRef90_A0A3N4L0N9_15_197      -------YWRDWFISARGHEYF------CEIDEEYLTDRFNLTGLQQEV-
075 UniRef90_A0A397J4Z1_55_250      ---TESLTWISWFCSLSGHEYF------AEVAEEFIEDDFNLTGLNSMV-
076 UniRef90_A0A6J3MH45_20_208      -SSSLSLTCMPQFVSSRGNEYF------CEIDEDYLTDRFNLTGLNLEV-
077 UniRef90_A0A2C5YX06_1078_1260   -------YWRDWFISSRGNEYF------CEIDEDYLTDRFNLTGLNTEV-
078 UniRef90_UPI0018755BD5_76_247   ---ADELQWISWFLSSKGNEYF------CEVDEDFILDRFNLTGLNNEV-
079 UniRef90_A0A4R0R889_49_217      ------AKWIFWFLSSKGNEYF------CEVEEDFILDRFNLTGLNNEV-
080 UniRef90_A0A0P1BJJ4_107_281     -SATDSLTWISWFCSLPGHEYF------AEVAEEFIEDDFNLTGLNALV-
081 UniRef90_A0A137PBH0_17_198      -------YWVDWYLSLKGSEYF------CEIDEDYLNDRFNLTGLPNEV-
082 UniRef90_A0A4U0WMK8_5_182       ------------FISSRGNEYF------CEIDEEYLTDRFNLTGLNTEV-
083 UniRef90_A0A1Y2F9D6_30_220      --GSENLSWIAWYCSLPGHDYF------CEVAEDYIEDDFNMTGLSSLV-
084 UniRef90_A0A0P8Y6J6_18_200      -----DGSWIGWFLNLQGNEFL------CRVPFDYLEDKFNLTGLENNV-
085 UniRef90_A0A2T9Z2A7_16_201      -------YWIRWFLNAKGNEYF------CEIDEEYILDRFNLTGLQSQV-
086 UniRef90_A0A1Y1YBP8_15_197      -------YWRDWFISSRGNEYF------CEIDEEYLTDRFNLTGLNTEV-
087 UniRef90_A0A0G2ESH3_15_197      -------YWRDWFISSRGNEYF------CEIDEEYLTDRFNLTGLQTEV-
088 UniRef90_A0A4V1J5H5_5_195       ---TDTMSWISWFCSYMGHEYF------TEVQEEFIEDEFNLTGLRQMV-
089 UniRef90_A0A4S8LIP9_20_193      -------QWISWFLSSKGNEYF------CEVEEDFILDRFNLTGLNNEV-
090 UniRef90_A0A1D8PNI6_16_200      ------EYWIDLFLGIKGNEYF------CDIDDEYIRDRFNLTGLNSEV-
091 UniRef90_A0A448YSS2_19_203      -------YWVDWFLGSRGNEYF------CDVDLEYITDRFNLTGLNQYV-
092 UniRef90_A0A2N1JDB9_48_222      -SGTEGLTWISWFCSLPSHQYF------AEVAEEFIEDDFNLTGLNFLV-
093 UniRef90_A0A0W4ZMB2_44_237      --DSETMTWISWWCSHVGHDYF------AEVSEEFIEDNFNLTGLSTLI-
094 UniRef90_A0A1D2VL05_15_200      -------YWVDSFLETKGNEYF------CDIDIEYITDRFNLTGLNNEV-
095 UniRef90_K0KFC4_18_201          -------FWIDWFLGTKGNEYF------VDIDVEYITDKFNLTGLYQEV-
096 UniRef90_A0A4P9ZSB4_16_199      -------YWAEWFLFLKGNEYF------VEIDEEFIGDRFNLTGLCQDV-
097 UniRef90_A0A7S2RTJ5_91_283      --SEESDTWVSWYCQLRGNEFF------CEVDQDYIDDPFNLYGLSSM--
098 UniRef90_UPI00053A2101_52_240   -SDKEDSSWISMFCNLRGNKFL------CEVDDDYILDDYNLCGLSHQV-
099 UniRef90_A0A1E3PJQ7_14_200      ----SEEYWVDWFLGAKGNEYF------CDVEQDFILDRFNLTGLQSEV-
100 UniRef90_A0A0W0FK04_40_210      ----STLTWISWFCSLPGHEYF------CEVTEDFIEDDFNLTGLNTMV-
101 UniRef90_K2LYQ1_23_212          ---EETIPWTVSFCQRKGHEFF------CIVDRNFIDDEFNLTGLSTMV-
102 UniRef90_A0A120K193_34_221      ------ELWIDLFLGMKGHEYF------CDIDTEYITDRFNLINLQKTV-
103 UniRef90_A0A1G4JQF6_19_211      -SSEYVEFWIDLFLGRKGHEYF------CDIDMDYITDRFNLINLQKSV-
104 UniRef90_A0A7G2C1K6_19_220      ---EEFISWINWFCELKGNEFF------CVVDREFIADEFNLTGLSSMV-
105 UniRef90_A0A4R5XEN6_51_221      ----SSLTWISWFCSLPGHEYF------CEVTEDFIEDEFNLTGLNSMV-
106 UniRef90_R4X9E9_30_221          -SSEEHLSWISWWCAHQGHEFF------AEVPEEFIEDDFNMTGLSSMC-
107 UniRef90_A0A1Y2FJU8_15_197      -------GFVDWILSSRGGEFF------CDIDAEYMTDRFNLTGLSAEV-
108 UniRef90_A0A1W4WCC7_11_192      ----ADGSWIGWFVGLQGNEFL------CRVPADYVEDKFNLLGLEGLV-
109 UniRef90_A0A6P4GHA8_18_195      ------GSWIGWFLGIKGNDFL------CRVPTDYIEDKFNLTGLE----
110 UniRef90_A0A0C7N2A5_19_211      -SSEYVEFWIDLFLGRKGHEYF------CDVDTDYIVDRFNLINLQKTV-
111 UniRef90_A7TQ13_22_208          -------LWIDLFLSRKGNEYF------CEVDVDYITDRFNLINLQKAV-
112 UniRef90_A0A0M4E7Q4_7_195       -SASHDGSWISWFLSLPMNEFL------CRVPVDYIQDRFNLTGLDAQLG
113 UniRef90_A0A1X2GUL9_10_193      -------RWVDWLLNQSGNDYL------CKIDLSYIEDRFNLTRLNEDI-
114 UniRef90_UPI000510F195_87_251   ---DEDASWISWFCNQKGNEFF------CEVDDDYTLDDFNRSGLRNQV-
115 UniRef90_A0A1B9HCU7_34_203      ----STITWINWYTSLPGHDYF------CEVHEDFIEDDFNLTGLQAMV-
116 UniRef90_A0A1A6A1S9_31_200      ---TSTLTWINWYTSLPGHDYF------CDVHEDFIEDDFNLTGLQAMV-
117 UniRef90_A0A4Q1BBE6_39_208      ----STITWINWFTSYPGHEYF------CEVHEEFIEDDFNLTGLQSMV-
118 UniRef90_A0A642VBA1_50_236      ------ETWISSFCSMVGHEYF------AEVLDDFVDDDFNLTGLGQMV-
119 UniRef90_A0A1E5RB29_103_291     ------EPWIDGFLGKKGNEYF------CEIDVDYITDRFNLMGLNRHI-
120 UniRef90_A0A060TJ45_17_202      -------TWVSAYCSMVGHEYF------CEVLDEFIDDDFNLTGLGQMV-
121 UniRef90_A0A376B670_70_257      -------QWIDTFLGKKSHEYF------CDIDTDYITDRFNLINLHKFV-
122 UniRef90_A0A507DW98_129_303     ---SSVISWVSWYCSLPGHEYF------LEVPEDFIEDDFNLTGLSYGI-
123 UniRef90_A0A0K3AQN1_24_214      -----ETTWIEWYCSLNGNQFY------VQVDEDYIRDDFNLIDIPNQV-
124 UniRef90_G0QMJ3_13_196          -------GWISWFCALEDHQFL------CEVDEDYIKDNFNLYGIKQKF-
```

```
125 UniRef90_A0A2E5QG28_55_190      ----TEQSWISWFCGLRGNELF------CEVDEDYIQDDFNLSGLSSSV-
126 UniRef90_W1Q7C8_11_202          -SSEEMDSWISQYCSIFGHDYF------VEVAPEFIEDDFNLTGLSSVV-
127 UniRef90_A0A1G4JSI2_22_213      -------EWIPSFCARFGHEYF------CQVPSEFIEDDFNMTGLAQEV-
128 UniRef90_R1E1U7_15_210          -SSLDEPTWVSWFCMLRNHEFF------CEVDPSFVQDRFNLYGLKELVG
129 UniRef90_C5M199_253_444         -SCSDDCGWIAWYCSLKGHEMF------AEVDEDYIRDAFNLYGLRAKV-
130 UniRef90_A0A0A1U1B9_13_198      --NSSDESWIEWFCGLDGNQYL------CEVDEDFIDDEFNIYGLSSYF-
131 UniRef90_L1LFG9_30_230          --SQSEVSWIEWYCNLKGNQYY------IQVDEAFIRDEFNLVGLQYHV-
132 UniRef90_A0A1X1BNS6_15_204      --SCDSMKWIQWFTSLEGHEFL------LEVDESYILDQFNLCGLKSM--
133 UniRef90_A7ART0_26_226          -----EIPWIEWYCSLKGHEFY------VQVDADYIRDEFNLVGLQQQV-
134 UniRef90_J9IFP4_26_217          ---ASQVGWIRWFCSLEGHEFL------TEVDEDYIKDQFNLYGLQATFP
135 UniRef90_A0A433P780_55_189      --------------------------------------------------
136 UniRef90_A0A7S1T6P4_10_137      --------------------------------------------------
137 UniRef90_F4PAV0_23_209          --SSSEVSWISWYCSLPEHEFF------LEIPEEYLEDEFNLAGLSSSV-
138 UniRef90_Q3SEC1_7_197           --ISEGGGWVQWFCAMEDHQFL------CEVDDEFIRDNFNLYGLKSKF-
139 UniRef90_A0A2A9M617_91_280      ----EGETWISWFCALEGHECF------GEVDEEYIKDTFNLFGLKPII-
140 UniRef90_A0A3M7MUJ5_976_1133    --SQSPDSWIAQFCSLVGHEYF------AEVSEDFIEDDFNLTGLQSQV-
141 UniRef90_A0A0K3C7J8_5_146       ----------------------------------------------ADI-
142 UniRef90_A0A507C5M5_148_333     --SSSLVSWITWYCSLPGHELF------VQVPEEFIEDDFNLTNLQNAI-
143 UniRef90_A0A1R1PPR8_25_196      ---DEPVSWIAWFCSLVGHEFF------CEVDESFIEDEFNLTDAEME--
144 UniRef90_A0A084G399_1151_1315   ----------SQFLSSRGNEYF------CEIDEEYILDRFNLTGLNTEV-
145 UniRef90_A0A0R3QRT7_7_137       --------------------------------------------------
146 UniRef90_A0A0L6X1G9_47_219      ----STLTWISWFCSLPGHEYF------CEVTEDFIEDDFNLTGLNAMV-
147 UniRef90_A0A6S7GGY9_40_229      -------GWVIRFRKRHGNEWL------EEIDRDFLRDYTNLTGLDEEI-
148 UniRef90_B8BZE6_1_179           ------------FCGLKGHEMF------CEVERAYIEDGFNLYGLRACV-
149 UniRef90_J9IF68_28_225          ---MESISWIEWHCSLEGHQFL------AQVDTQFIRDNFNQSGLKKIF-
150 UniRef90_B4IGD2_3_165           -----------------GNEFV------CRVPIDFIENKFNLTGLE----

001 Input_pdb_SEQRES_A             P-HYRQAL-DMILDLE----PD-------------EELEDNP---NQSDL
002 UniRef90_D3PJV2_1_193           P-HYRQAL-DLILDLE----SD-------------EDLDDNP---NQSDL
003 UniRef90_A0A443SMC7_9_201       P-HYRQAL-DMILDLE----PD-------------DELEDNP---NQSDL
004 UniRef90_A0A7R8WGP2_1_193       P-HYRAAL-DMILDLE----PD-------------EDLEDNP---NQSDL
005 UniRef90_A0A7R9KER7_1_193       P-HYRQAL-DMILDLE----PD-------------DELEENP---NQSDL
006 UniRef90_K7FHH3_1_194           P-HYRQAL-DMILDLE----PD-------------EELEDNP---NQSDL
007 UniRef90_A0A1I8ISK0_1_192       P-HYHQAL-DMILDLE----AD-------------DNLSD-A---NQSDI
008 UniRef90_A0A6P9F8P8_1_185       P-HYRQAL-DMILDLE----PD-------------EELEDNP---NQSDL
009 UniRef90_A0A315WGG2_1_182       P-HYRQAL-DLILDLE----PD-------------EETEDNP---NQSDL
010 UniRef90_A0A0B2UX36_1_192       P-HYRQAL-DMILDYE----PD-------------EDM-DSG---LQGEL
011 UniRef90_A0A210QLV3_15_188      P-HYRQAL-DMILDLE----PD-------------DELEDNP---NQSDL
012 UniRef90_A0A6A4WWH4_1_175       P-HYRQAL-DMILDLE----P-----------------------------
013 UniRef90_A0A673UYT7_1_167       P-HYRQAL-DMILDL---------------------------------DL
014 UniRef90_A0A090MVV2_1_191       P-HFRNAM-DMILDLE----PD---------------DEYDE---IHSDL
015 UniRef90_A0A0N5AZ67_43_214      P-HYREAL-DMILDLE----PD-------------DDFDDDP---NQSDL
016 UniRef90_A0A0D2U6T3_2_190       P-LYRQAI-DMILDLE-------------------MEEELLE---DQQSA
017 UniRef90_A0A0N4UWY4_7_187       P-HYRHAL-EMILDSE----PGTTTYY--------LDEEINN---AESEL
018 UniRef90_A0A1W4V542_1_191       N-NYRCAL-EVILDLN-------------------SGSASDM---PAEPE
019 UniRef90_A0A6P4JVR0_1_191       T-NYRCAL-EVILDLN-------------------SGSASDQ---PPEPE
020 UniRef90_A0A5F8HIB5_36_187      P-HYRQVLDDMILDLE----PD-------------EELENNP---NQRDL
021 UniRef90_A0A7S4IQT0_22_213      P-YYDYAL-DMVLDVE----MP-------------MEDSLTE---EQQEI
022 UniRef90_A0A6J0MJ32_91_282      P-YYDYAL-DLILDVD----FS-------------QGERFTD---EQNEL
023 UniRef90_A0A388JRV3_116_307     P-YYDYAL-DLILDVE----SP-------------NDDMLTD---EQHEM
024 UniRef90_A0A0D6QXG8_112_303     P-YYDYAL-DLILDVE----SS-------------NDDILTE---EQNEL
025 UniRef90_A0A151ZRX5_21_208      P-HYDSAL-GIILDSD-------------------PEDPLSE---DQQET
026 UniRef90_UPI0019298C3E_89_278   P-YYDYAL-DLILDVE----SS-------------HGDMFTE---EQNEL
027 UniRef90_A0A7S3PWQ0_98_288      P-YYDYAL-DMVLDVE----MP-------------MEEKLTE---AQQEI
028 UniRef90_A0A6I8V063_1_191       R-NYSSAL-DVILDLN-------------------SGSRSDN---GTHSE
029 UniRef90_A0A250WT55_67_256      P-YYDYAL-DMILDND----PP-------------HDVMLTD---QQHEL
030 UniRef90_A0A6P3Z3J9_56_245      P-YYDYAL-DLILDVD----SS-------------HGDMFTE---EQNEL
031 UniRef90_M4CKX0_70_259          P-YYDYAL-DLILDID----SS-------------NGDMFTE---EQNEL
032 UniRef90_A0A1U8N6I0_93_282      P-YYDYAL-DLILDVE-----S-------------HGDMFTE---EQNEL
033 UniRef90_A0A1U8N824_86_276      P-YYDYAL-DLILDVE----SS-------------HGDMFTE---EQNEL
034 UniRef90_A0A0D2QNY2_22_212      P-YYGYAL-DLILDVE----SS-------------HGDMFTE---DQNEL
```

```
035 UniRef90_UPI0007EFC624_83_271   P-YYDHAL-DLILDLE----SS--------------PGDVFTE---EQHDL
036 UniRef90_A0A6J0MMZ8_88_277      P-YYDYAL-DLILDVE----SS--------------NSDMFTE---EQNEL
037 UniRef90_A0A2I0X454_94_284      P-YYDYAL-DLILDVE----SS--------------HGDTFTE---EQNEL
038 UniRef90_UPI00092FBD2C_80_270   P-YYDYAL-DLILDVV----SS--------------HGDTFRE---ERNEL
039 UniRef90_F0ZXH6_18_205          Q-FYDGAL-GIILDSD--------------------PEDPLSE---DQQEA
040 UniRef90_A0A1U8IV83_84_274      P-YYDYAL-DLILDVE----SS--------------HGDMFTE---EQNEL
041 UniRef90_A0A261XW74_26_208      R-EYQQAL-DLITDSL---------------------EDDLDD---EMREQ
042 UniRef90_A0A162PQW6_12_196      QHYYLEAL-DMITDNL--------------------DEESLDE---KVREQ
043 UniRef90_A0A7S3CG89_21_212      P-YYDYAL-DLILDIE----SP--------------HEEMLTE---EQHEL
044 UniRef90_A0A162UUP8_10_199      KQYYLEAL-DMITDNL--------------------DEESFDE---KAREQ
045 UniRef90_A0A7R9Y293_54_246      P-YYDYAL-DLILDVE----SP--------------NDDMLTE---EQHEM
046 UniRef90_UPI00142E405F_77_267   P-YYEYAL-DLILDVE----SS--------------HGEMFTE---EQNEL
047 UniRef90_A0A0K9Q617_79_270      P-YYDYAL-DLILDVE----SC--------------NGDMFTE---EQNEL
048 UniRef90_A0A6P5GZT7_89_278      N-YYDYAL-DMILDND----SL--------------NDE-VDG---EEHSE
049 UniRef90_A0A1R1PTJ3_11_196      A-HYQQAF-DLITDQL--------------------DEDSFDD---EQSKI
050 UniRef90_A0A7J7KUM0_133_320     P-FYDYAL-DMILDND----SL--------------SGE-LDG---EEHSE
051 UniRef90_F0W6R1_98_288          P-YYEYAL-DLILDIE----NP--------------NEQHLTQ---MQQEM
052 UniRef90_A0A4P9XR61_13_199      Q-HYSQAL-DLITDSL----G---------------AYTDLDD---EVREA
053 UniRef90_A0A7S0ES88_51_226      P-YYDYAL-DIILDIE----TP--------------QDEMLTQ---DQQEL
054 UniRef90_A0A420Y4K1_15_197      S-YYQYAL-DLVTDVF---------------------DLDCDD---EMRET
055 UniRef90_A4RSN9_26_212          P-YYDYAL-DTILDVE----RG--------------HST-LSE---QQQEL
056 UniRef90_A0A642UK79_17_202      D-HIQQSI-DVITDNF---------------------DADVSK---DEREH
057 UniRef90_A0A1R3S306_15_197      A-YYQYAL-DLVTDVF---------------------DLDADD---DLREQ
058 UniRef90_A0A183REZ2_109_233     ---------------------------------------SDNP---ENTDL
059 UniRef90_A0A067N327_22_192      Q-SYSQAL-DLITDQL---------------------DEDIED---ELRGS
060 UniRef90_A0A507CIU8_18_202      S-HYNLAF-DMITDSI---------------------EEEIDE---DLRSE
061 UniRef90_A0A7D8Z2K1_14_184      Q-EYQRAL-DLITDNL--------------------DDEDLDD---ETREA
062 UniRef90_A0A0F4ZAU1_16_198      Q-YYQYAL-DLITDVF---------------------DLECDD---DMRDA
063 UniRef90_A0A7S1VF37_12_200      P-YYEDAL-DRILGDD----V---------------APDDFTE---ERLEL
064 UniRef90_A0A0L0H4P4_15_200      Q-HYALAF-DMITDAL---------------------EEDLDD---DMRAE
065 UniRef90_A0A6H0Y5P8_15_197      V-DYQYAL-DLVTDVF---------------------DMEVDD---ESRES
066 UniRef90_A0A7R9BIZ0_390_519     --------------------EE--------------EILDLAP---SQQDQ
067 UniRef90_A0A3E2HRT2_15_197      Q-YYQYAL-DLVTDVF---------------------DLDCDD---EMRET
068 UniRef90_A0A6A6V4N0_15_197      H-HYQLAL-DLVTDVF---------------------DNDCSD---ELRDQ
069 UniRef90_A0A6A6YKN2_15_197      Q-YYQYAL-DLVTDVF---------------------DLDCDD---DMRES
070 UniRef90_A0A2J5HQ92_16_198      P-YYQYAL-DLVTDVF---------------------DLDADD---ELREQ
071 UniRef90_A0A0J9XL38_11_196      P-RLSQAI-EIITDSF---------------------EDVLSN---EDREK
072 UniRef90_A0A2D3V5L0_22_206      Q-YYQYAL-DLVTDVF---------------------DMEVDD---DMREQ
073 UniRef90_M5BRL5_5_172           S-NYQLAL-DFVTDNL---------------------DDDIDE---ELRQS
074 UniRef90_A0A3N4L0N9_15_197      Q-YYQYAL-DLVTDVF---------------------DFECED---EVREQ
075 UniRef90_A0A397J4Z1_55_250      P-FYKEAL-EMILDVE----SGKHGRI-------TSEEETLKV---PDVSI
076 UniRef90_A0A6J3MH45_20_208      T-YYQYAL-DLVTDVF---------------------DMDVDD---DMREA
077 UniRef90_A0A2C5YX06_1078_1260   Q-YYQYAL-DLVTDVF---------------------DLDCDD---EMRET
078 UniRef90_UPI0018755BD5_76_247   A-NYAQAL-DLITDQL---------------------DDEVQE---ELRGS
079 UniRef90_A0A4R0R889_49_217      A-NYAQAL-DLITDNL---------------------DDEIQD---ELRGS
080 UniRef90_A0A0P1BJJ4_107_281     P-FYKEAL-EMILDVE----P---------------QEDSLKI---PDVSI
081 UniRef90_A0A137PBH0_17_198      E-NFSLAL-DMILDNL---------------------GVALDN---ATSEV
082 UniRef90_A0A4U0WMK8_5_182       Q-YYQYAL-DLVTDVF---------------------DLECDD---DMREQ
083 UniRef90_A0A1Y2F9D6_30_220      P-MYKEAL-EFVLDLE----PDD-------------DEFELDA---AERRI
084 UniRef90_A0A0P8Y6J6_18_200      P-NYTQAL-DLIMDPE------------------------FDNNC-WDNSM
085 UniRef90_A0A2T9Z2A7_16_201      P-FYQQAI-ELMTDEL--------------------DENNYSE---EQFNE
086 UniRef90_A0A1Y1YBP8_15_197      Q-YYQYAL-DLVTDVF---------------------DFDCDD---DMREQ
087 UniRef90_A0A0G2ESH3_15_197      Q-YYQYAL-DLVTDVF---------------------DLDCDD---DMREA
088 UniRef90_A0A4V1J5H5_5_195       P-YYNYAL-EMILDIE----EDM-------------DADLSPV---PDIQV
089 UniRef90_A0A4S8LIP9_20_193      A-NYPQAL-DLITDQL----GGCS------------TDDDIQD---EFRGS
090 UniRef90_A0A1D8PNI6_16_200      S-KLPILI-DIITDVI--------------------DIESQPE---EHKDS
091 UniRef90_A0A448YSS2_19_203      D-RIGLLV-DIITDRT----QI---------------SETQPE---EARAR
092 UniRef90_A0A2N1JDB9_48_222      P-FYKEAL-EMILDIE----P---------------QEDSLKI---PDVSI
093 UniRef90_A0A0W4ZMB2_44_237      P-FYKEAL-EMILDVD----SEN-------------TDEHVRA---PDQNI
094 UniRef90_A0A1D2VL05_15_200      D-RISYVI-DIITDNS----PK--------------RNPREFQ---RIREQ
095 UniRef90_K0KFC4_18_201          E-KIPQVI-DVITDGL--------------------KIEELTD---EEREI
096 UniRef90_A0A4P9ZSB4_16_199      E-HYSLAL-QLISDNL--------------------EEAKLNQ---EMRDA
097 UniRef90_A0A7S2RTJ5_91_283      S-YYNEAL-DVIMDLE----SELD------------EEYEPNS---DARML
098 UniRef90_UPI00053A2101_52_240   P-YYDYAL-DLILDVE-ESSSS--------------RGEIITE---EHNEL
```

```
099 UniRef90_A0A1E3PJQ7_14_200     E-HFQLAL-DLITDEL-------------------DLEEFSE---DHRYV
100 UniRef90_A0A0W0FK04_40_210     P-FWKEAM-EMVLDVE----P--------------DEEASKI---PDVSI
101 UniRef90_K2LYQ1_23_212         Y-FYNEAL-DLILDLK----A--------------SSIMLTA---EQKRI
102 UniRef90_A0A120K193_34_221     S-KFTQVI-QYMVDEL----DEA------------LVEAMPR---TKLNQ
103 UniRef90_A0A1G4JQF6_19_211     N-KFTQVI-QYIVDEL----DDS------------VLEQMPT---SRLEQ
104 UniRef90_A0A7G2C1K6_19_220     S-FYHYAL-ELILDVD----AHCGSENGSGGS---STPPLTE---EQQRL
105 UniRef90_A0A4R5XEN6_51_221     P-FWKEAM-EMVLDVE----P---------------EDSLKI---PDVSI
106 UniRef90_R4X9E9_30_221         S-LYKEAL-ELILDLE----PD-------------DDSYTGA---AEQDA
107 UniRef90_A0A1Y2FJU8_15_197     P-NFQIAY-DLICDTT--------------------DIEVNF---SEEGG
108 UniRef90_A0A1W4WCC7_11_192     E-NFPMAH-ELVLEPE-----------------------FRH---WDSGV
109 UniRef90_A0A6P4GHA8_18_195     --FFSQTL-DLVLEPE-----------------------FEIPG-WDNTV
110 UniRef90_A0A0C7N2A5_19_211     N-KFTQVI-QYIVDEL----DDT------------ILEQLSS---GRMQQ
111 UniRef90_A7TQ13_22_208         S-KFSLVI-QYIVDEL----DES------------VLEAMSP---SKLEQ
112 UniRef90_A0A0M4E7Q4_7_195      S-QLSDAL-DLVLESE-----------------------FDTECFQQVET
113 UniRef90_A0A1X2GUL9_10_193     PHFYEEAL-EMMIDQF-------------------DYRDYSE---ATRVA
114 UniRef90_UPI000510F195_87_251  P-FYDYAL-DLMLDIE----SS-------------NDGLTDE---EQNNL
115 UniRef90_A0A1B9HCU7_34_203     P-FFKEAL-EMVLDVE----P--------------DAESSNL---PDISI
116 UniRef90_A0A1A6A1S9_31_200     P-FFKEAL-EMVLDVE----P--------------EEDSSKI---PDVSI
117 UniRef90_A0A4Q1BBE6_39_208     P-FWKEAL-EMVIDIE----P--------------EEDSSKI---PDVSI
118 UniRef90_A0A642VBA1_50_236     P-HYKQAL-ELISDLE----P---------------SEPVKA---PTIPL
119 UniRef90_A0A1E5RB29_103_291    STRFSTVI-NFIVDEL----DDE------------QLSKLRG---SDYEQ
120 UniRef90_A0A060TJ45_17_202     P-HYREAL-ELISDLE----P---------------SEQVKV---PNIPQ
121 UniRef90_A0A376B670_70_257     SCRFSTVI-QYIIDEL----GES------------TLERMST---KELEL
122 UniRef90_A0A507DW98_129_303    P-YYNEAL-DLILDID-------------------QEEPANP---ACMSE
123 UniRef90_A0A0K3AQN1_24_214     P-YYSRAL-SIILDCG----DEDDY----------MSDDNSK---ENQQI
124 UniRef90_G0QMJ3_13_196         N-HYNEAL-EMILSTE----SP-------------DDDDLED---ERFLE
125 UniRef90_A0A2E5QG28_55_190     P-YYDYAL-DTILDID----RP-------------HSD-ITD---QQQEL
126 UniRef90_W1Q7C8_11_202         P-FYREAL-DVILDLE----P---------------EVPIKV---SNVPL
127 UniRef90_A0A1G4JSI2_22_213     P-HYRKAL-DLVLDLE----AMSDDE---------GQESEEP---VSRSI
128 UniRef90_R1E1U7_15_210         P-LYGEAM-TMILGYA----P--------------TEKQLADPT-QRVHK
129 UniRef90_C5M199_253_444        Q-FYDHAL-EMILSDE----RP-------------DEEDLAD---NDFLE
130 UniRef90_A0A0A1U1B9_13_198     T-YYQEAL-DVIMSVA-------------------EPESFVD---KDILL
131 UniRef90_L1LFG9_30_230         T-CYTRAL-QLILDHC----DNDYYDNEDEYD---DESNSDK---GKQQV
132 UniRef90_A0A1X1BNS6_15_204     K-HYDSAM-DMILGFA-----P-------------TEELFVD---ASFLE
133 UniRef90_A7ART0_26_226         S-YYSHAI-RMILDNY----DDFDYDCYEENDGDTYGPCSER---AKQRI
134 UniRef90_J9IFP4_26_217         KDKFKTCV-KMILSPM----AP-------------NEEDLAD---EHFLE
135 UniRef90_A0A433P780_55_189     ------------------------------------EEELDD---DMREQ
136 UniRef90_A0A7S1T6P4_10_137     -----------------------------------------E---EQHEM
137 UniRef90_F4PAV0_23_209         P-LYNEAL-DLILDLE----LECSDTDDSNSAKVPQQRQQYN---AHLRM
138 UniRef90_Q3SEC1_7_197          N-FYNEAL-DLILSSE----TP-------------DDEDLED---ERFLE
139 UniRef90_A0A2A9M617_91_280     G-NYDAAL-DMILGTA-----P-------------DDEDIDE---QHFLE
140 UniRef90_A0A3M7MUJ5_976_1133   S-MYKEAL-EMILD------------------------------------
141 UniRef90_A0A0K3C7J8_5_146      ----------VLAPTA--------------------DEDLND---EQREE
142 UniRef90_A0A507C5M5_148_333    P-LYSAAL-DMILDME----PTSPCQNTASSV-DTAAQATQA---ARPAD
143 UniRef90_A0A1R1PPR8_25_196     -----------------------------------DEDSVGS---AEQEM
144 UniRef90_A0A084G399_1151_1315  Q-YYQYAL-ELVTDSF--------------------DLDCED---SMRDA
145 UniRef90_A0A0R3QRT7_7_137      -----------------------------------DEARARL---SQSMI
146 UniRef90_A0A0L6X1G9_47_219     P-FWKEAM-EMVLDVE----T--------------DEDASKI---PDVSI
147 UniRef90_A0A6S7GGY9_40_229     C-YLSHGL-HIILSDGLYLDSN-------------GNSKIRT---KNEGK
148 UniRef90_B8BZE6_1_179          S-NFSDCL-DLILDRI----G------------------PDD---SDDSH
149 UniRef90_J9IF68_28_225         E-NYEDAL-QMILGES-----P-------------EQADLDK---ESFSK
150 UniRef90_B4IGD2_3_165          --YLKDTM-NVVLEPL-----------------------FD----RSVAW
```

```
001 Input_pdb_SEQRES_A             IEQ-AAEMLYGLIHARYILTNRGIAQM--LEKYQQGDFGYCPRVYC---E
002 UniRef90_D3PJV2_1_193          IEQ-AAEMLYGLIHARYILTNRGIAQM--IEKYQSGDFGHCPRVYC---E
003 UniRef90_A0A443SMC7_9_201      IEQ-AAEMLYGLIHARYILTNRGIALM--IEKYQNGDFRYCPRVYC---E
004 UniRef90_A0A7R8WGP2_1_193      IEQ-SAEMLYGLIHCRYILTNRGIAQM--IEKYQNGDFGHCPRVYC---E
005 UniRef90_A0A7R9KER7_1_193      IEQ-AAEMLYGLIHARFILTNRGIAQM--IDKYQNGDFGYCPRVYC---E
006 UniRef90_K7FHH3_1_194          IEQ-AAEMLYGLIHARYILTNRGIAQM--LEKYQQGDFGYCPRVYC---E
007 UniRef90_A0A1I8ISK0_1_192      IEQ-AAEMLYGLIHARYIMTNRGIAQM--IEKWQNGEFSYCPRVYC---E
008 UniRef90_A0A6P9F8P8_1_185      IEQ-AAEMLYGLIHARYILTNRGIAQM--LEKYQQG--GYSPRVYC---E
```

```
009 UniRef90_A0A315WGG2_1_182         IEQ-AAEMLYGLIHARYILTNRGIAQM--LEKYQQGDFGYCPRVYC---E
010 UniRef90_A0A0B2UX36_1_192         IEQ-AAEMLYGLIHARYILTTRGIAQM--VEKWQNGDFGYCPRVYC---E
011 UniRef90_A0A210QLV3_15_188        IEQ-AAEMLYGLIHARYIMTNRGIAQM--IEKWQQGDFGYCPRVYC---E
012 UniRef90_A0A6A4WWH4_1_175         ------EMLYGLIHARYILTNRGIAQM--IEKYQSGEFGYCPRVYC---E
013 UniRef90_A0A673UYT7_1_167         IEQ-AAEMLYGLIHARYILTNRGVAQM--LEKYQQGDFGYCPRVYC---E
014 UniRef90_A0A090MVV2_1_191         VEQ-AAEMLYGLIHARYILTNRGIQLM--LEKYRNEEFGNCPRVYC---E
015 UniRef90_A0A0N5AZ67_43_214        VEQ-AAEMLYGLIHARYILTNRGIQQM--LEKWREGDFGVCPRFYC---E
016 UniRef90_A0A0D2U6T3_2_190         IDS-SAEMLYGLIHARYILTNRGIIQM--LEKYRNGDFGVCPRIYC---E
017 UniRef90_A0A0N4UWY4_7_187         VEQ-AAEMLYGLIHARYILTDRGITQM--MDKWNNGDFGFCPRVYC---E
018 UniRef90_A0A1W4V542_1_191         LEA-SAEKLYGLIHARFILTNRGIELM--LNKFYKGEFGTCPRAFC---Q
019 UniRef90_A0A6P4JVR0_1_191         LEA-SAEKLYGLIHARFILTNRGIELM--LEKYYKEAFGTCPRLFC---Q
020 UniRef90_A0A5F8HIB5_36_187        IEQ-AAGMLYGLIHACYILTNCGIAQM--LEKYQQGDFGYCPLVYG---E
021 UniRef90_A0A7S4IQT0_22_213        VES-AAEMLYGLIHARYILTNRGMHAM--YEKYRSASFGRCPHVFC---Q
022 UniRef90_A0A6J0MJ32_91_282        VES-AAEMLYGMIHARYILTSKGLASM--LDKYKNYDFGRCPRVYC---C
023 UniRef90_A0A388JRV3_116_307       VES-AAEMLYGLIHVRYILTSRGMSAM--LDKFKNVDFGRCPRVFC---S
024 UniRef90_A0A0D6QXG8_112_303       VES-AAEMLYGLIHVRYILTSKGMNAM--LEKFKNTDFGRCPRVYC---S
025 UniRef90_A0A151ZRX5_21_208        LER-SADILYGLIHARYILTSKGLAHM--HDKFKKAEFGRCPRVFC---Q
026 UniRef90_UPI0019298C3E_89_278     VES-AAEMLYGLIHARYILTSKGMAAM--LEKYKNYDFGRCPRVYC---C
027 UniRef90_A0A7S3PWQ0_98_288        VES-AAEMLYGLIHARYILTNRGMHAM--YEKYRSASFGRCPHVFC---Q
028 UniRef90_A0A6I8V063_1_191         VDE-SAEKLYGMIHARFILTDRGIDLM--IRKFHKGVFGTCPRVFC---H
029 UniRef90_A0A250WT55_67_256        LES-AAEMLYGLIHARYIVTTKGLAAM--LEKFKNCDFGRCPRCLC---E
030 UniRef90_A0A6P3Z3J9_56_245        VES-AAEMLYGLIHARYILTSKGMAAM--LEKYKNYDFGRCPRVYC---A
031 UniRef90_M4CKX0_70_259            VES-AAEMLYGLIHVRYILTTKGMAAM--LEKYKSCDFGRCPRVFC---S
032 UniRef90_A0A1U8N6I0_93_282        VES-AAEMLYGLIHARYILTSKGMAAM--LDKYKNYDFGRCPRVYC---C
033 UniRef90_A0A1U8N824_86_276        VES-AAEMLYGLIHVRYILTSKGMSAM--LEKYKSYDFGRCPRVYC---C
034 UniRef90_A0A0D2QNY2_22_212        VES-AAEMLYGLIHVRYILTSKGMSAM--LEKYKNYDFGRCPRAFC---C
035 UniRef90_UPI0007EFC624_83_271     VET-AAEMLYGLIHVRYVLTTKGQAAM--LEKYKNAEFGRCPRVCC---S
036 UniRef90_A0A6J0MMZ8_88_277        VES-AAEMLYGLIHVRYILTTKGMAAM--LEKYKNCDFGRCPRVFC---S
037 UniRef90_A0A2I0X454_94_284        VES-AAEMLYGLIHARYILTSKGMAAM--LEKYKNYDFGRCPRVYC---C
038 UniRef90_UPI00092FBD2C_80_270     IES-AAKMLYGLMHARFILTSKGMAAM--LDKYRNYDFGRCPRLYC---N
039 UniRef90_F0ZXH6_18_205            LER-SADILYGLIHARYILTPKGLAHM--HEKFKKAEFGRCPRVFC---Q
040 UniRef90_A0A1U8IV83_84_274        VES-AAEMLYGLIHVRYILTSKGMSAM--LEKYKSYGFGRCPRVYC---C
041 UniRef90_A0A261XW74_26_208        VEK-SARHLYGLIHARFIITSRGLAKM--LEKYKKADFGRCPRVLC---Q
042 UniRef90_A0A162PQW6_12_196        IEK-AARHLYGLIHARFVITTRGLAKM--LDKYKKAEFGRCPRVLC---N
043 UniRef90_A0A7S3CG89_21_212        VES-AAEMLYGLIHQRFILTSRGLSAM--YDKFKNVDFGRCPRVLC---G
044 UniRef90_A0A162UUP8_10_199        IEK-AARHLYGLIHARFVITSRGLIRM--LEKHKKGDFGRCPRVLC---N
045 UniRef90_A0A7R9Y293_54_246        VES-AAEMLYGLIHVRFILTTRGMSKM--LEKFKNVDFGRCPRVFC---S
046 UniRef90_UPI00142E405F_77_267     IESTAAEMLYGLIHARYILTTKGLAAM--LDKYKNYDFGRCPRVYC---C
047 UniRef90_A0A0K9Q617_79_270        VES-AAEMLYGLIHIRYILTNKGMAGM--LEKFKNCDFGRCPSFNC---C
048 UniRef90_A0A6P5GZT7_89_278        VES-AAELLYGLIHARYILTGKGLNAM--HEKYKRADFGRCPRVHC---G
049 UniRef90_A0A1R1PTJ3_11_196        LDR-SAKHLYGLIHARFIITTRGLIKM--VEKYKRGDFGRCPRVYC---N
050 UniRef90_A0A7J7KUM0_133_320       IES-AAELLYGLIHARYIITTKGLNAM--HEKYKKIDFGRCPRVCC---G
051 UniRef90_F0W6R1_98_288            VES-AAEMLYGLIHARYILTTKGMAAM--LEKYRNVEFGRCHRVFC---Q
052 UniRef90_A0A4P9XR61_13_199        VEK-SARHLYGLIHARFVVTGRGLSKM--LEKYKKAEFGRCPRVLC---N
053 UniRef90_A0A7S0ES88_51_226        VES-AAEVLYGLIHARYIATQRGMQQM--LEKYRRCDFGRCPRVLC---D
054 UniRef90_A0A420Y4K1_15_197        IEK-SARHLYGLVHARYIVTTRGLAKM--LEKYKKSDFGKCPRVLC---H
055 UniRef90_A4RSN9_26_212            VES-AAEMLYGLIHARYILTQRGMTLM--LEKYKQNHFGRCPRFMC---A
056 UniRef90_A0A642UK79_17_202        IEK-IATHLYGLVHARYILTARGLQKM--LDKYKNCDFGRCPRVHC---S
057 UniRef90_A0A1R3S306_15_197        IEK-SARHLYGLVHARYIVTTRGLSKM--VEKYKRGDFGKCPRVMC---D
058 UniRef90_A0A183REZ2_109_233       IEQ-AAEMLYGLIHSRYILTNRGICFM--VAKWQQGDFGYCPRVYC---E
059 UniRef90_A0A067N327_22_192        VEI-SARFLYGLIHARWIITSRGLAKM--LEKYKKADFGRCPRVLC---Y
060 UniRef90_A0A507CIU8_18_202        VEK-SARHLYGLIHARYVITARGLAKM--AEKFRTGEFGRCPRVLC---H
061 UniRef90_A0A7D8Z2K1_14_184        VET-SARFLYGLIHARYIVTSRGLQKM--LDKYRKADFGRCPRVYC---A
062 UniRef90_A0A0F4ZAU1_16_198        IEK-SARQLYGLVHARFIVTTRGLSKM--LEKYKKGDFGKCPRVLC---E
063 UniRef90_A0A7S1VF37_12_200        IEQ-SAQLLYGLIHARFIVTGGGVQMM--MGKFEVGEFGRCPRVCC---E
064 UniRef90_A0A0L0H4P4_15_200        VEK-SARHLYGLIHARFILTARGLTKM--QEKFRHAEFGRCPRVLC---D
065 UniRef90_A0A6H0Y5P8_15_197        IER-SARHLYGLVHARYIVTTRGLAKM--MEKYKQGVFGKCPRVIC---E
066 UniRef90_A0A7R9BIZ0_390_519       VHQ-ASILLYGLIHARFILTGHGLGQM--IEKYQNGDFGHCPRVYC---E
067 UniRef90_A0A3E2HRT2_15_197        IEK-SARHLYGLVHARYIVTTRGLAKM--LEKYKKADFGKCPRVMC---K
068 UniRef90_A0A6A6V4N0_15_197        IEK-SARHLYGLVHARYIVTTRGLAKM--LEKFKKADFGKCPRVMC---D
069 UniRef90_A0A6A6YKN2_15_197        IEK-SARHLYGLVHARYIVTTRGLAKM--LEKYKKGDFGKCPRVMC---D
070 UniRef90_A0A2J5HQ92_16_198        IEK-AARHLYGLVHARYIVTTRGLAKM--LDKYKKCDFGKCPRVLC---E
071 UniRef90_A0A0J9XL38_11_196        LEK-SARHLYGLVHARYILTSRGLQKM--LEKYRNCDFGRCPRVHC---Q
```

```
072 UniRef90_A0A2D3V5L0_22_206      IEK-SARHLYGLVHARYVVTTRGLAKM--MEKFKQGVFGKCPRVIC---E
073 UniRef90_M5BRL5_5_172           VEV-SARLLYGLIHARWIVTSRGLSKM--LEKYKKADFGRCPRVMC---Q
074 UniRef90_A0A3N4L0N9_15_197      IEK-SARHLYGLVHARYIITTRGLAKM--LEKYKKCDFGRCPRVLC---R
075 UniRef90_A0A397J4Z1_55_250      VET-SAELLYGLIHQRYIITRLGLQQM--VDKYESRHFGCCPRVYC---Q
076 UniRef90_A0A6J3MH45_20_208      IEK-SARHLYGLVHARYVVTTRGLTKM--MEKYKQGVFGKCPRVIC---E
077 UniRef90_A0A2C5YX06_1078_1260   IEK-SARHLYGLVHARYIVTTRGLTKM--LDKYKKAEFGKCPRVNC---H
078 UniRef90_UPI0018755BD5_76_247   LDT-QARLLYGLIHARWIVTARGLAKM--VEKYKRGDFGRCPRVLC---H
079 UniRef90_A0A4R0R889_49_217      LDV-QARLLYGLIHARWIVTARGLAKM--LEKYKRADFGRCPRVLC---Q
080 UniRef90_A0A0P1BJJ4_107_281     VES-SAELLYGLIHQRFILTRQGLSQM--AEKYEAGHFGYCPRVFC---H
081 UniRef90_A0A137PBH0_17_198      VEK-DAKHLYGLIHARYILTMRGLAKM--LDKMKNNHFGKCPRVLC---Q
082 UniRef90_A0A4U0WMK8_5_182       IEK-SARHLYGLVHARYIVTTRGLAKM--IEKFKQGAFGKCPRVIC---E
083 UniRef90_A0A1Y2F9D6_30_220      VEA-SAELLYGLVHQRFITSRAGLALM--AEKYEAGHFGVCPRLLC---H
084 UniRef90_A0A0P8Y6J6_18_200      DSQ-SAEQLYGMIHARYILTPRGVDDM--LLKYERGEFGSCPRVYC---K
085 UniRef90_A0A2T9Z2A7_16_201      FFS-SAKHLYGLIHARFIITARGIMKM--VEKYKRCDFGRCPRVYC---N
086 UniRef90_A0A1Y1YBP8_15_197      IEK-SARHLYGLVHARYIVTTRGLLKM--LEKFKKSDFGKCPRVMC---D
087 UniRef90_A0A0G2ESH3_15_197      IEK-SARHLYGLVHARYIVTTRGLAKM--LDKYKKADFGKCPRVLC---G
088 UniRef90_A0A4V1J5H5_5_195       IEN-SAERLYGLMHARYIITRNGLQNM--SDKYLVGQFGNCPRTLC---N
089 UniRef90_A0A4S8LIP9_20_193      LDV-QARLLYGLIHARWIVTARGLAKM--LEKYKRAEFGRCPRVLC---Q
090 UniRef90_A0A1D8PNI6_16_200      LEH-NARILYGLIHARYILTTRGLNKM--FEKFRSGDFGYCPRVHC---Q
091 UniRef90_A0A448YSS2_19_203      LED-NAKFLYALIHARYIITSRGLSKM--LEKYRNGDFGYCPRVNC---K
092 UniRef90_A0A2N1JDB9_48_222      VES-SAELLYGLIHQRYVITRQGLQQM--VEKHEEGHFGVCPRVYC---N
093 UniRef90_A0A0W4ZMB2_44_237      IES-SAELLYGLIHQRYIVSRSGLHAM--AEKFEMGHFGHCPRVYC---N
094 UniRef90_A0A1D2VL05_15_200      MEA-QARYLYGLIHARYIITSRGLQKM--LDKYRNGDFGYCQRVYC---D
095 UniRef90_K0KFC4_18_201          LEF-NARLLYGLIHSRYIITARGLTKM--LEKYKNADFGYCSRVFC---Q
096 UniRef90_A0A4P9ZSB4_16_199      VER-SARHLYGLIHARYVLTAPGMQKV--LEKYKSAGFGRCPRVLC---H
097 UniRef90_A0A7S2RTJ5_91_283      IDN-TAETLYGLIHARFILTTQGLLAM--NEKYVAGDFGECLRLQC---E
098 UniRef90_UPI00053A2101_52_240   IES-AAEILYGMIHARYILTG--------LKKYNNYDFGRCPKLYC---R
099 UniRef90_A0A1E3PJQ7_14_200      LDE-SARKLYGLIHARFILSPRGLQKM--LEKYKNSDFGRCPRLHC---N
100 UniRef90_A0A0W0FK04_40_210      VES-SAEMLYGLVHQRYILTRVGLQAM--VDKYEAGIFGSCPRVYC---V
101 UniRef90_K2LYQ1_23_212          VNS-SAETLYGLIHARFITTNRGIAMM--EEKFYAGDFGRCPRVFC---D
102 UniRef90_A0A120K193_34_221      LES-DARKLYGLIHARYIITAKGLQKM--LQKYRDADFGRCPRVHC---N
103 UniRef90_A0A1G4JQF6_19_211      LES-DARKLYGLIHARFIITVKGLQKM--LQKYRNADFGRCPRVYC---K
104 UniRef90_A0A7G2C1K6_19_220      VES-SAETLYGLIHARFITTNRGLKMM--EDKFANCDFGTCPRVFC---G
105 UniRef90_A0A4R5XEN6_51_221      VEA-SAELMYGLIHQRYILTRFGLNAM--AEKYEAGQFGTCPRVYC---L
106 UniRef90_R4X9E9_30_221          VEA-AAEYLFTYIHQRYICSRNGLITM--AEKYEAGHFGVCPRVMC---K
107 UniRef90_A0A1Y2FJU8_15_197      YAA-YGRHLYGMIHARYILTPRGLQKM--LEKYKQGDFGVCPRFLC---H
108 UniRef90_A0A1W4WCC7_11_192      DTT-DAERLYGLIHARYILTQRGVEDM--CLKYERGDFGSCPRFYC---R
109 UniRef90_A0A6P4GHA8_18_195      DTT-DAEQLYGMIHARYILTARGVEDM--CLKYERGDFGSCPRVFC---K
110 UniRef90_A0A0C7N2A5_19_211      LEH-DACKLYGLIHARYVITIKGLQKM--LQKYKEADFGRCPRVYC---N
111 UniRef90_A7TQ13_22_208          LEA-DARKLYSLIHARYIITVKGLQKM--LAKYKDAEFGRCPRIYC---N
112 UniRef90_A0A0M4E7Q4_7_195       DLD-AAAKLYGLIHARYILTARGIDDM--CMKYQRGDFGKCPRLYC---H
113 UniRef90_A0A1X2GUL9_10_193      IEN-AAQHLYGLIHARYIITNSGMSQM--IAKYKKGVFGVCPRHYC---D
114 UniRef90_UPI000510F195_87_251   IES-TTEMLYGLIHARYIVTNKGLSAM--LEKYRNYDFGRCPRVNC---S
115 UniRef90_A0A1B9HCU7_34_203      VES-SAELLYGLVHQRFILTKIGLSCM--VEKYEAGHFGACPRVFC---H
116 UniRef90_A0A1A6A1S9_31_200      VES-SAELLYGLVHQRFILTKVGLSCM--VEKYEAGHFGACPRVFC---H
117 UniRef90_A0A4Q1BBE6_39_208      VES-SAELLYGLVHQRFICTKAGLAAM--ADKYEQGHFGACPRVFC---M
118 UniRef90_A0A642VBA1_50_236      IEQ-SAELLYGLIHARFILSRPGLQIM--AQKYECHHFGVCPRYYC---D
119 UniRef90_A0A1E5RB29_103_291     LCK-DAAKLYGLIHARYIITLKGLDKM--MHKYKEGDFGKCPRVFC---E
120 UniRef90_A0A060TJ45_17_202      IEQ-SAELLYGLIHARFILSRPGLQIM--AQKYECLHFGVCPRYNC---D
121 UniRef90_A0A376B670_70_257      LEA-DANKLYGLIHARYVITIKGLEKM--YTKYKDADFGRCPRVFC---N
122 UniRef90_A0A507DW98_129_303     IES-SAEILYGSIHARYIITKPGLHGM--LQKYLEGVFGTCPRVYCGALG
123 UniRef90_A0A0K3AQN1_24_214      LQS-STQLLYGLIHCRYILTNKGMQAM--LEKYENHTFGNCPNYSC---E
124 UniRef90_G0QMJ3_13_196          IYQ-EATDLYGLIHSRFTTSPKGLAMM--REKFLLGRFGVCPRVLC---E
125 UniRef90_A0A2E5QG28_55_190      IES-AAEMLYGLIHARYILTTRGMTAM--LEKYKQNHFGRCPRFLC---A
126 UniRef90_W1Q7C8_11_202          VEH-AAQLLYGLIHARFILTKQGYHLM--AEKYEQKVFGTCPRYYC---E
127 UniRef90_A0A1G4JSI2_22_213      VEH-AAEQLYGLVHARYVLTKPGLQAM--AEKFDHKEFGTCPRYYC---A
128 UniRef90_R1E1U7_15_210          VLD-TARILYGLIHARYILTAQGLSDM--KAKYKAHEFGACPRTLC---D
129 UniRef90_C5M199_253_444         IYR-EAVDLYGLIHARYCLTPRGLSVV--KEKYLRGDYGTCPRVYC---N
130 UniRef90_A0A0A1U1B9_13_198      IKK-EAALLYGLIHARYILTSSGLKKM--YAKYRKGEFGGCPRVLC---D
131 UniRef90_L1LFG9_30_230          IDS-SAQLLYGLIHSRFIITNKGMQLM--MLKYKEKVFGTCPNFSC---E
132 UniRef90_A0A1X1BNS6_15_204      IYR-SATDLYGLIHARFITSPMGLQMM--KEKYQQGVFGHCPRVRC---Q
133 UniRef90_A7ART0_26_226          INT-SSQLLYGLIHSRFIITSKGMALM--LQKYKEKVFGTCPNFSC---E
134 UniRef90_J9IFP4_26_217          LNQ-EASDLYGLIHARYIQTPRGIAKI--YQKYLSGVFGYCPRALC---D
135 UniRef90_A0A433P780_55_189      VEK-SARHLYGLIHARFVITSRGLAKMVRLEKYKKADFGRCPRVLC---H
```

136 UniRef90_A0A7S1T6P4_10_137 IES-AAELLYGLIHVRYILTTRGLSNM--FEKYKNLDFGRCPRAFC---N
137 UniRef90_F4PAV0_23_209 VES-SAQILYGLIHARFLLTKPALVNM--AERFRMKDFGRCPRVGC---G
138 UniRef90_Q3SEC1_7_197 VYQ-EATDLYGLIHARFIITAKGLSMM--KEKYLGGKFGACPRVLC---E
139 UniRef90_A0A2A9M617_91_280 VYR-DAMDLYGLIHARYIITPRGLAQM--REKYIAGQFGECPRVLC---D
140 UniRef90_A0A3M7MUJ5_976_1133 --S-SAELLYGLIHARYITSRPGIQQM--MEKYELAHFGYCPRVYC---A
141 UniRef90_A0A0K3C7J8_5_146 LES-SARHLYGLIHARYVITSRGLSKM--IEKYKKGDFGRCPRVLC---Y
142 UniRef90_A0A507C5M5_148_333 VEA-SAEVLYGLLHARYIVTKPGLQTM--ADRLSHGEFGKCPRSGC---V
143 UniRef90_A0A1R1PPR8_25_196 IDT-SADTLYGLIHARYIITTSGLRQM--AIKYENGEFGTCARYLC---K
144 UniRef90_A0A084G399_1151_1315 IEK-SARHLYGL-----------------LDKYKKAEFGKCPRVMC---N
145 UniRef90_A0A0R3QRT7_7_137 GEQ-VTQTLYGLIHARYILTGGGIDQM--MIKWRNGDFGFCPRFYC---E
146 UniRef90_A0A0L6X1G9_47_219 IES-SAEMLYGLVHQRYILTRSGLQAM--VEKYENGVFG----------
147 UniRef90_A0A6S7GGY9_40_229 HLE-AAEMLYSLVHARYMLTSKGLEKV--FKKYKEGFYGLCPRYYC---E
148 UniRef90_B8BZE6_1_179 LTQ-SACTLYGLIHARYIVTAHGLDSM--YNKYASKEFGTCPLVQC---N
149 UniRef90_J9IF68_28_225 IYQ-QAFDLYGLIHSRFIQTPKGFQII--RQKYQDGVFGKCPRVQC---G
150 UniRef90_B4IGD2_3_165 VSG-YEEKLYGMIHARYIMSARGVEDM--RLKYLMGDFGSCPKFYC---K

001 Input_pdb_SEQRES_A NQPMLPIG-LSDIPGEAM---VKLYCPKCMDVYT-PK--SSRHH------
002 UniRef90_D3PJV2_1_193 NQPMLPIG-LSDIPGEAM---VKLYCPKCMDVYN-PK--SSRHH------
003 UniRef90_A0A443SMC7_9_201 NQPMLPIG-LSDVPGEAM---VKLYCPKCNDVYN-PK--SSRHH------
004 UniRef90_A0A7R8WGP2_1_193 NQPMLPVG-LSDVPGEAM---VKLYCPKCMEVYT-PK--SSRHH------
005 UniRef90_A0A7R9KER7_1_193 NQTMLPIG-LSDLPGEAM---VKLYCPKCMDIYN-PK--SSRHH------
006 UniRef90_K7FHH3_1_194 NQPMLPIG-LSDIPGEAM---VKLYCPKCMDVYT-PK--SSRHH------
007 UniRef90_A0A1I8ISK0_1_192 NQPVMPIG-LSDVPGEAM---VKLYCPKCQDVYT-PK--SSRHH------
008 UniRef90_A0A6P9F8P8_1_185 NQPMLPVG-LLDIPGEAM---VKLYCPKCMDVYT-TK--SSRHH------
009 UniRef90_A0A315WGG2_1_182 NQPMLPI----DIPGEAM---VKLYCPKCMDVYT-PK--SSRHH------
010 UniRef90_A0A0B2UX36_1_192 TQKLLPIG-LSDVPGESM---VKLYCPKCMDVYT-PR--SSRNQ------
011 UniRef90_A0A210QLV3_15_188 NQAMLPIG-LSDVPGEAM---VKLYCPKCQDVYT-PK--SSRHH------
012 UniRef90_A0A6A4WWH4_1_175 NHPMLPIG-LSDVPGEAM---VKLYCPKCMDVYT-PK--SSRHH------
013 UniRef90_A0A673UYT7_1_167 NQPMLPVG-LSDIPGEAM---VKLYCPKCMDVYT-PK--SSRHH------
014 UniRef90_A0A090MVV2_1_191 GQATLPIG-LSDICGESM---VKLYCPKCCDIFN-PR--SSKYS------
015 UniRef90_A0A0N5AZ67_43_214 NQPMLPIG-LSDIPGESM---VKLYCPRCCDVYV-PK--SSKHH------
016 UniRef90_A0A0D2U6T3_2_190 DQAVLPVG-LTDVPSEST---VKLFCPRCEEVYQ-PR--SARHQ------
017 UniRef90_A0A0N4UWY4_7_187 SQKFLPIG-LSDVPGEAM---VKLYCPKCMDVYT-PR--NVRNQ------
018 UniRef90_A0A1W4V542_1_191 RQPVLPIG-LSDNPGEEM---VRIYCPKCNDVYF-PK--AARHS------
019 UniRef90_A0A6P4JVR0_1_191 RQPVLPIG-LSDNPGEEM---VRLYCPKCNDVYL-PK--AARHS------
020 UniRef90_A0A5F8HIB5_36_187 NQPML------------------LYCPKCMDVYT-PK--PSRHH------
021 UniRef90_A0A7S4IQT0_22_213 GQPVLPVG-LSDLPRNYT---VNVFCPRCHGLFF-PK--STRQA------
022 UniRef90_A0A6J0MJ32_91_282 GQPCLPVG-QSDIPRSST---VKIYCPKCEDVYY-PR--SKYQG------
023 UniRef90_A0A388JRV3_116_307 GQPCLPMG-QSDIPRQST---VKIYCPKCEDIFY-PR--SKYQG------
024 UniRef90_A0A0D6QXG8_112_303 GQPCLPVG-QSDIPRTST---VKIYCPKCEDVYY-PR--SKYQG------
025 UniRef90_A0A151ZRX5_21_208 NQAVLPVG-LSDLQGEET---VKVYCPKCNDIYN-PK--YRRHS------
026 UniRef90_UPI0019298C3E_89_278 GQPCLPVG-QSDVPRSST---VKIYCPKCDDIYY-PR--SKYQG------
027 UniRef90_A0A7S3PWQ0_98_288 GQPVLPVG-LSDLPRNYT---VNVFCPRCHGLFF-PK--STRQA------
028 UniRef90_A0A6I8V063_1_191 RQHVLPIG-LSDTPGEEM---VRLYCPKCNDIYT-PK--SGRHR------
029 UniRef90_A0A250WT55_67_256 GQACLPVG-TSDIPGQST---VKVFCPKCDDIYY-PR--SEYQC------
030 UniRef90_A0A6P3Z3J9_56_245 GQPCLPVG-QSDIPRSST---VKIYCPRCEDIYY-PR--SKYQ-------
031 UniRef90_M4CKX0_70_259 GQSCLPVG-QSDIPRSST---VKIYCPKCEDLYY-PR--SKYQG------
032 UniRef90_A0A1U8N6I0_93_282 GQPCLPVG-QSDIPRAST---VKIYCPRCEDIYY-PR--SKYQG------
033 UniRef90_A0A1U8N824_86_276 GQPCLPVG-QSDIPRSST---VKIYCPKCEDIYY-PR--SKYQG------
034 UniRef90_A0A0D2QNY2_22_212 GQHCLPVG-QSDIPRSST---VKVYCPKCEDIYY-PR--SKYQG------
035 UniRef90_UPI0007EFC624_83_271 GQPCLAVG-QSDIPRQST---VKIYCPKCEDIYA-PR--SRHQD------
036 UniRef90_A0A6J0MMZ8_88_277 GQSCLPVG-QSDTPRSRT---VKIYCPKCEDVYY-PR--SKYQG------
037 UniRef90_A0A2I0X454_94_284 GQPCLPVG-QSDISRSST---VKIYCPKCEDIYY-PR--SRFQG------
038 UniRef90_UPI00092FBD2C_80_270 GQPCLPVG-QSDIVRSSS---VKIYCPRCEDIYS-PS--SKYQD------
039 UniRef90_F0ZXH6_18_205 NQPVLPVG-LADMQGVDT---VKVYCPKCNDIFN-PK--YRRHS------
040 UniRef90_A0A1U8IV83_84_274 GQSCLPVG-QSDIPRAST---VKIYCPKCEDVYY-PR--SKYQG------
041 UniRef90_A0A261XW74_26_208 NQPLFPVG-LSDVSYTKT---VKLYCPRCEDIYN-PK--SSRHA------
042 UniRef90_A0A162PQW6_12_196 LQSLLPVG-MSDVPMTKT---VKLYCPRCEDIYN-PK--SSRHA------
043 UniRef90_A0A7S3CG89_21_212 GQPCLPVG-QADLPRTST---VKIFCPKCQDIYY-PR--SKLQG------
044 UniRef90_A0A162UUP8_10_199 LQSLLPVG-LTDVPMTKT---VKLYCPRCEDIYN-PK--SVRHA------
045 UniRef90_A0A7R9Y293_54_246 GQNCLPVG-QSDVPRTST---VKIFCPKCEDIYY-PR--SKYQG------

```
046 UniRef90_UPI00142E405F_77_267   GQPCLPVG-QSDLPRSST---VKIYCPKCEDIYY-PR--SKYQG------
047 UniRef90_A0A0K9Q617_79_270     GQPCLPLG-QSYIPRVGT---VKLYCPKCEDLYY-PR--SKYQS------
048 UniRef90_A0A6P5GZT7_89_278     GQPCLPVG-TSDIPRNGS---VKIYCPKCEDIYL-PR--CKYQS------
049 UniRef90_A0A1R1PTJ3_11_196     QQNLLPVG-ITDVPNEVS---VKLYCCRCEDIYH-PK--SSRHC------
050 UniRef90_A0A7J7KUM0_133_320    GQPGLPVG-MSDIPRNGS---VKIYCPKCEDLYF-PR--CKYQN------
051 UniRef90_F0W6R1_98_288         GQPVLPVG-QSDGPRHTT---VNVFCPKCREIYF-PK--SQRAG------
052 UniRef90_A0A4P9XR61_13_199     GQAVVPVG-LSDLPYSKA---VKLYCTRCQDIYT-PK--STRHA------
053 UniRef90_A0A7S0ES88_51_226     GQAVLPVG-QSDIPHQLT---VKVFCPKCQDMYY-PK--SSRKA------
054 UniRef90_A0A420Y4K1_15_197     SHPLLPVG-LSDCPNVRP---VKLYCARCEDTYN-PK--SSRHA------
055 UniRef90_A4RSN9_26_212         NTPCLPVG-TSDIFRTAT---VKIFCPKCKDIYF-PR--SKYQG------
056 UniRef90_A0A642UK79_17_202     SNPLLPIG-LHDLPRHST---VKLYCARCEDIYN-PK--SSRHQ------
057 UniRef90_A0A1R3S306_15_197     GHPLLPMG-QHDVPNQST---VRLYCPKCEDIYN-PK--SSRHA------
058 UniRef90_A0A183REZ2_109_233    SQPCLPVG-LSDVPGEAM---VKIYCPRCQDTYT-PK--STRHH------
059 UniRef90_A0A067N327_22_192     SQPLLPIG-LTDTPYEKA---VKLYCPRCEDLYS-PK--SSRHG------
060 UniRef90_A0A507CIU8_18_202     NQTSLPVG-MSDLPGTKS---VKLYCPRCEDVYN-PP--YRRHA------
061 UniRef90_A0A7D8Z2K1_14_184     GQPLLPVG-LTDIPYQKA---VKLYCPRCEDIYS-PK--SNRHG------
062 UniRef90_A0A0F4ZAU1_16_198     GHHLLPMG-LADIPGQKA---VKLYCARCEDIYN-PK--SSRHS------
063 UniRef90_A0A7S1VF37_12_200     DQPVIPVG-ESDVAGQRR---ARVFCPRCQDIYV-PR--SRHHA------
064 UniRef90_A0A0L0H4P4_15_200     NQPVLPVG-LWDVAGVKC---VKLYCPRCEDVYN-PI--SRRHL------
065 UniRef90_A0A6H0Y5P8_15_197     SQHLLPMG-QHDIPNISH---VKLYCAKCEDLYN-PK--SSRHN------
066 UniRef90_A0A7R9BIZ0_390_519    NQPMLPIG-LSDVPGDAM---VKLYCPKCMDVYN-PK--SSRHH------
067 UniRef90_A0A3E2HRT2_15_197     SHPLLPMG-QSDNPNVKA---VKLYCAKCEDIYN-PK--SSRHA------
068 UniRef90_A0A6A6V4N0_15_197     SQPLLPMG-LSDVANQQP---VKLYCARCEDLYN-PK--SSRHA------
069 UniRef90_A0A6A6YKN2_15_197     QQPLLPMG-QSDVSNTSP---VKLFCAKCEDLYN-PK--SSRHA------
070 UniRef90_A0A2J5HQ92_16_198     GHPLLPMG-QHDIPNTST---VRLYCAKCEDIYN-PK--SSRHG------
071 UniRef90_A0A0J9XL38_11_196     LHPLLPVG-LSDIPRHNA---VKLYCAKCEDIYS-PK--LSRHA------
072 UniRef90_A0A2D3V5L0_22_206     SQHLLPMG-QHDIPNTSH---VKLYCAKCEDIYN-PK--SSRHN------
073 UniRef90_M5BRL5_5_172          SQPLLPCG-LTDVPYEKA---VKLYCPRCEDLYS-PK--SSRHG------
074 UniRef90_A0A3N4L0N9_15_197     SHPLLPLG-LSDIAQQKS---VKLYCAKCEDVYS-PK--STRHA------
075 UniRef90_A0A397J4Z1_55_250     SCPVIPCG-RSDLPGLET---VKLYCPNCLDLYT-PP--SSRFH------
076 UniRef90_A0A6J3MH45_20_208     SQHLLPMG-QHDQPGHSH---VKLYCAKCEDIYN-PK--SSRHN------
077 UniRef90_A0A2C5YX06_1078_1260  SHPLLPMG-LSDVPNVKA---VKLYCARCEDIYN-PK--SSRHA------
078 UniRef90_UPI0018755BD5_76_247  AQPLLPVG-IVDIPYEKS---VKLFCGRCEDLYS-PK--SSRHG------
079 UniRef90_A0A4R0R889_49_217     SQPLLPVG-LTDIPYQKA---VKLYCGRCEDIYS-PK--SSRHG------
080 UniRef90_A0A0P1BJJ4_107_281    SHPVLPCG-RSDLPGLDT---VKLFCPNCIDNYS-PP--SSRFH------
081 UniRef90_A0A137PBH0_17_198     GQPLLPVG-LSDIPRSKG---VKLYCSRCEDIYQ-PK--ANRHI------
082 UniRef90_A0A4U0WMK8_5_182      QQPLLPMG-QHDTPNVSA---VKLYCAKCEDIYN-PK--SSRHA------
083 UniRef90_A0A1Y2F9D6_30_220     EAAVLPVG-LSDVVEEDP---VKLFCPNCLDAYN-PP--NSRYN------
084 UniRef90_A0A0P8Y6J6_18_200     GQRVLPVG-LTDLIGQSH---VKVYCPRCHDIFQ-PR---SRCA------
085 UniRef90_A0A2T9Z2A7_16_201     QHPLLPIG-VTDEVGEMP---VRLYCCKCEDIYI-PK--SSKHN------
086 UniRef90_A0A1Y1YBP8_15_197     SQPLLPMG-QSDIANTSP---VKLYCARCEDLYN-PK--SSRHA------
087 UniRef90_A0A0G2ESH3_15_197     GHPLLPMG-QSDIAHLKT---VKLYCPKCEDIYN-PK--SSRHA------
088 UniRef90_A0A4V1J5H5_5_195      NTYVVPCG-RSDTPGVDS---VKLYCPKCKDIYA-PP--STRFN------
089 UniRef90_A0A4S8LIP9_20_193     QQPLLPVG-LTDIPYEKS---VKLYCGRCEDLYS-PK--SSRHG------
090 UniRef90_A0A1D8PNI6_16_200     LNPLLPIG-LNDQPRMAS---VKLYCSKCEDLYN-PK--SGRHS------
091 UniRef90_A0A448YSS2_19_203     LTPLLPVG-LSDKPRVAS---VKLYCPNCEDLYN-PK--SSRHS------
092 UniRef90_A0A2N1JDB9_48_222     TQLVLPCG-RSDLPGLDT---VKLYCPNCQDTYT-PP--SSRFH------
093 UniRef90_A0A0W4ZMB2_44_237     STSVVPCG-RSDLPGVET---VKLFCPNCLDIYV-PP--NSRFQ------
094 UniRef90_A0A1D2VL05_15_200     LQPLLPIG-LNDQPKLQP---VKLYCPTCEDIYN-PK--SSRHG------
095 UniRef90_K0KFC4_18_201         LQHLLPVG-LSDTTGVSA---VKLYCPKCEDLYN-PK--SSRHS------
096 UniRef90_A0A4P9ZSB4_16_199     NQALLPVG-LTDVAGLQS---VKLYCPHCEDIYN-PK--SPRHS------
097 UniRef90_A0A7S2RTJ5_91_283     RQALLPIG-LSDIQGEST---VKTFCPRCREIYL-PV--KVRQR------
098 UniRef90_UPI00053A2101_52_240  GQPCLPVG-LSDIPRAST---VKIYCPKCEDVYY-PP--SKYEA------
099 UniRef90_A0A1E3PJQ7_14_200     LHPLLPIG-IYEKQGQGT---VKLYCSKCEELYN-PK--SIRHS------
100 UniRef90_A0A0W0FK04_40_210     GCNVVPCG-RSDTPGLDT---VKLFCPNCNDIYT-PP--SSRFQ------
101 UniRef90_K2LYQ1_23_212         RQALLPVG-QSDVVRESF---VKLYCPKCNDIYY-PR--SSRHR------
102 UniRef90_A0A120K193_34_221     FQPLLPVG-LHDVPGIDC---VKLYCPSCEDLYI-PK--SSRHS------
103 UniRef90_A0A1G4JQF6_19_211     FQPLLPVG-LHDSPGIDC---VKLYCPCCEDLYI-PK--SSRHN------
104 UniRef90_A0A7G2C1K6_19_220     GQAVLPVG-QSDVVRESS---VKLFCPKCEDIYY-PR--SSRHK------
105 UniRef90_A0A4R5XEN6_51_221     ACHVVPCG-RSDMPGLDT---VKLYCPNCNDIFT-PP--SSKFQ------
106 UniRef90_R4X9E9_30_221         KTAVLPVG-LYDVIDDCN---VKLFCPSCLDIYN-PP--NSRFQ------
107 UniRef90_A0A1Y2FJU8_15_197     SQHLLPIG-PSDLPSIAS---VQTYCPHCEDLYT-PK--SKRHA------
108 UniRef90_A0A1W4WCC7_11_192     EQRVLPIG-LSDQCGQAH---VKVYCPQCTDVFQ-PR---SRCA------
109 UniRef90_A0A6P4GHA8_18_195     GQRTLPVG-LSDQWNQSH---VKIYCPRCQDIFQ-PR---SRCG------
```

```
110 UniRef90_A0A0C7N2A5_19_211       FQPLLPVG-LHDAPGVDC---VKLYCPCCEDLYV-PK--STRHS------
111 UniRef90_A7TQ13_22_208           FQPLLPVG-LHDIPGIDS---VKLYCPSCEDLYN-PK--SPRHA------
112 UniRef90_A0A0M4E7Q4_7_195        SQLMLPVG-VSDRFGESH---VKLYCARCRDIYQ-PQ---AHCA------
113 UniRef90_A0A1X2GUL9_10_193       DQPLLPVG-LSDLPQKKT---VKMYCPRCTDVYQ-CS--SPNHI------
114 UniRef90_UPI000510F195_87_251    KQRCLPVG-ESDIPRLST---VKIYCPKCEDIYS-PQ---SRHP------
115 UniRef90_A0A1B9HCU7_34_203       ATPVLPCG-RADMPGIDT---VKLYCPNCGDIYA-PP--SSKYA------
116 UniRef90_A0A1A6A1S9_31_200       ATPVLPCG-RADMPGIDT---VKLYCPNCGDIYT-PP--SSKYA------
117 UniRef90_A0A4Q1BBE6_39_208       ATHVLPCG-RSDMPGVDT---VKLFCPNCGDIYT-PP--SSKYA------
118 UniRef90_A0A642VBA1_50_236       GTGLLPIG-RYDLPGYET---VRLYCPCCMDIYM-PP--NSRYL------
119 UniRef90_A0A1E5RB29_103_291      GQHLLPVG-LHDSYSKEF---VKLYCPCCEDVYV-PK--STRHS------
120 UniRef90_A0A060TJ45_17_202       QTGLLPMG-RHDLPGYET---VRLYCPCCTDIYH-PP--NSRYL------
121 UniRef90_A0A376B670_70_257       YQPLLPIG-LHDQPDMDY---VKLYCPSCEDLYT-PK--SSRHS------
122 UniRef90_A0A507DW98_129_303      ALSLLPMG-PSDVPGEDT---IKMVCGRCGDLYF-PK--EAKYQ------
123 UniRef90_A0A0K3AQN1_24_214       NMPGLPIG-VVDAPSYHT---AKIFCPRCNEAYHPPK--QNKLC------
124 UniRef90_G0QMJ3_13_196           RQNVLPLG-MSEELRTSR---VKVFCPRCEDVYI-PK---KNP------
125 UniRef90_A0A2E5QG28_55_190       NAPCLPVG-TSDIFRTAT---VKIFCPKCK--------------------
126 UniRef90_W1Q7C8_11_202           GMRLIPIG-RYDQTGIET---VRLYCPCCNDIYL-PS--SSRYL------
127 UniRef90_A0A1G4JSI2_22_213       GMQMLPCG-LTDTLGKLT---VRLYCPSCQDLYL-PQ--SSRHL------
128 UniRef90_R1E1U7_15_210           KQVVLPVG-LSDTHSTSNENPLKLFCPRCQELYD-HN--VPGGN------
129 UniRef90_C5M199_253_444          GQHVLPTGRVGEELRVEP---VKLYCPKCEQLYV-PR---QKHA------
130 UniRef90_A0A0A1U1B9_13_198       NHAVLPVG-TSDIPYIGE---TKLFCPKCGEEYSIPQ--GFVGS------
131 UniRef90_L1LFG9_30_230           NTAVLPVG-LVDAPSQHT---AKVFCPNCNEVYHPPK--NSRLG------
132 UniRef90_A0A1X1BNS6_15_204       RQNVLPVG-FSDSLHSHR---IKKYCPRCQEAYL-FKP-GEANA------
133 UniRef90_A7ART0_26_226           NTAVLPIG-ITDAPAYHN---TKIFCPRCNEVYHPPK--NSRLS------
134 UniRef90_J9IFP4_26_217           KQKVLPVG-LSDALKTSR---FKVFCPRCEEVYL-PK---HRNV------
135 UniRef90_A0A433P780_55_189       NQPLLPVG-LSDIPYTKT---VKLYCPRCEDIYN-PK--SSRHA------
136 UniRef90_A0A7S1T6P4_10_137       GQPCLPVG-FSDVPRNSS---VKIYCPKCEDVYH-PR--SKYHS------
137 UniRef90_F4PAV0_23_209           KCGVVPCG-LSDIPGVAT---LKMYCPRCGDLYS-PK--KPRFQ------
138 UniRef90_Q3SEC1_7_197            RQNVLPVG-MSEELRTSR---VKVFCPRCEEVYI-PK---KKCP------
139 UniRef90_A0A2A9M617_91_280       RHPVLPVG-LSPDLRSHR---LRLYCPLCQEAYE-VREGSEEAK------
140 UniRef90_A0A3M7MUJ5_976_1133     GARVLPVG-LTDTPGQQT---VKLYCPSCLDVYT-PP--NSRFQ------
141 UniRef90_A0A0K3C7J8_5_146        GQSLLPLG-LSDIAYQKA---VKLYCPRCEDLYS-PK--SSRHG------
142 UniRef90_A0A507C5M5_148_333      GAYVLPCG-RSDGLGADT---VKMFCPRCCDLYH-PR--ETRYH------
143 UniRef90_A0A1R1PPR8_25_196       GTHLLPCG-LSDNLDVDS---VKLFCPSCLDIYN-PL--NSRFY------
144 UniRef90_A0A084G399_1151_1315    SHPLLPMG-LSDVPNLKP---VKLYCAKCEDIYN-PK--SSRHA------
145 UniRef90_A0A0R3QRT7_7_137        KQNLLPVG-ISDIPKVST---VKLFCPSCVDIYT-PI--NEYHQ------
146 UniRef90_A0A0L6X1G9_47_219       ----------SYLPGLDT---MKLFCPNCNDVYV-PP--SSKFQ------
147 UniRef90_A0A6S7GGY9_40_229       KTTVLPIG-MSDKLGQGN---VKTYCPSCEDIYH-TE--LPGNE------
148 UniRef90_B8BZE6_1_179            GQPVLPVG-VKDEIGVDT---VKIFCPKCKSVYQ-PP--PIRTRS-----
149 UniRef90_J9IF68_28_225           MQAVLPIG-MSDELSISK---VKIYCPKCEDVFV-PK--GQRSSHSTNSK
150 UniRef90_B4IGD2_3_165            GQKALPVG-LSDKWGQSK---VKIYCPSCKDVFR-PK----YRP------

001_Input_pdb_SEQRES_A           ----HTDGAYFGTGFPHMLFMVH-PE---YRPK---RPANQFVPRLYGFK
002 UniRef90_D3PJV2_1_193        ----HTDGSYFGTGFPHMLFMVH-PE---YRPK---RPASQFVPRLYGFK
003 UniRef90_A0A443SMC7_9_201    ----HTDGAYFGTGFPHMLFMVH-PE---YRPK---KPINQFVPRLYGFK
004 UniRef90_A0A7R8WGP2_1_193    ----HTDGAYFGTGFPHMLFMVH-PE---YRPK---RSVNQYVARLYGFK
005 UniRef90_A0A7R9KER7_1_193    ----HTDGAYFGSGFPHMLFMVH-PE---YRPK---RPINQFFPRLYGFK
006 UniRef90_K7FHH3_1_194        ----HTDGAYFGTGFPHMLFM----E---YRPK---RPANQFVPRLYGFK
007 UniRef90_A0A1I8ISK0_1_192    ----HTDGAYFGTGFPHMLFLVH-PE---YRPK---RPANQFVPRLYGFK
008 UniRef90_A0A6P9F8P8_1_185    ----HTDGAYFGTGFPHMLFMVH-PA---YRPK---QPANQFVPGL----
009 UniRef90_A0A315WGG2_1_182    ----HTDGAYFGTGFPHMLFMVN-PE---YRPK---RPANQFVPRF----
010 UniRef90_A0A0B2UX36_1_192    ----HTDGAYFGTGFPHMLFFVH-PE---LRPK---RPTNQFVSRLYGFK
011 UniRef90_A0A210QLV3_15_188   ----HTDGSYFGTGFPHMLFMVH-PE---YRPK---RPANQFVPRLYGFK
012 UniRef90_A0A6A4WWH4_1_175    ----HIDGAYFGTGFPHMLFMVH-PE---YRPK---KPTNQFVPRLYGFK
013 UniRef90_A0A673UYT7_1_167    ----HTDGAYFGTGFPHMLFMVH-PE---YRPK---WRA-----------
014 UniRef90_A0A090MVV2_1_191    ----HTDGAYFGTGFPHMLFFVH-PE---LRPK---SPTKYYIPRLFGFK
015 UniRef90_A0A0N5AZ67_43_214   ----NTDGSYFGTGFPHMLFFVH-PE---ERPI---RQSAKFVPRLYGFK
016 UniRef90_A0A0D2U6T3_2_190    ----QLDGSYFGRTFPHMLFTVH-PE---LRPQ---RNPPKYIPTIYGFK
017 UniRef90_A0A0N4UWY4_7_187    ----HTDGAYFGTGFPHMFFFAH-PE---VRPG---LPSAHFVPRLYGFK
018 UniRef90_A0A1W4V542_1_191    ----NLDGAFFGTGFPHMLFMVN-PE---ARPK---RTKQKFVPRLYGFK
019 UniRef90_A0A6P4JVR0_1_191    ----NLDGAYFGTGFPHMLFMVN-PE---ARPK---RGKQKFVPRLYGFK
```

```
020 UniRef90_A0A5F8HIB5_36_187        ----HTDGAYFGTGFPHMPFMVH-PE---YRPK---QPANQFVPRLY---
021 UniRef90_A0A7S4IQT0_22_213        ----NIDGAYFGTTFPHLYLMTH-PD---MIPV---KPAQRYIPRVYGFR
022 UniRef90_A0A6J0MJ32_91_282        ----NIDGAYFGTTFPHLFLMTY-GH---LKPQ---KAAQSYVPRVFGFK
023 UniRef90_A0A388JRV3_116_307       ----NIDGAYFGTTFPHLFMMTY-PY---IKPP---KPAQSYVPRIFGFK
024 UniRef90_A0A0D6QXG8_112_303       ----NTDGAYFGTTFPHLFLMTY-SH---IKPS---KPVQSYVPKIFGFK
025 UniRef90_A0A151ZRX5_21_208        ----HIDGAYFGTTFPHLLLITY-PE---LIPT---KTPQQYVPKIYGFK
026 UniRef90_UPI0019298C3E_89_278     ----NIDGAYFGTTFPHLFLMTY-GH---LKPQ---KATQRFVPRVFGFK
027 UniRef90_A0A7S3PWQ0_98_288        ----NIDGAYFGTTFPHLYLMTH-PD---MIPV---KPTQEYTPRVYGFK
028 UniRef90_A0A6I8V063_1_191         ----NLDGAFFGTNFPQMLFMVK-PE---ARPK---RSKTKFVPRLYGFK
029 UniRef90_A0A250WT55_67_256        ----SVDGAYFSTTFPHLMMMTY-PT---HRPP---KSVEVYVPRVFGFK
030 UniRef90_A0A6P3Z3J9_56_245        ----DIDGAYFGTTFPHLFLMTY-GH---LKPH---KASQSYVPRVFGFK
031 UniRef90_M4CKX0_70_259            ----NIDGAYFGTTFPHLFLMAH-GN---VKPQ---KPSQSYVPKIFGFK
032 UniRef90_A0A1U8N6I0_93_282        ----NIDGAYFGTTFPNLFLMTH-GH---LKPQ---KATQSYVPRVFGYK
033 UniRef90_A0A1U8N824_86_276        ----NMDGAYFGTTFPHLFLMTY-GH---LKPQ---KPTQNYTPRVFGFK
034 UniRef90_A0A0D2QNY2_22_212        ----NIDGAYFGTTFPHLFLMTY-GH---LMPQ---KPAQNYIPRVFGFK
035 UniRef90_UPI0007EFC624_83_271     ----NLDGAYFGTTFPHLFLMTY-GH---LKPQ---KKLTSYIPRVFGFK
036 UniRef90_A0A6J0MMZ8_88_277        ----NIDGAYFGTTFPHLFLMAY-GN---LKPQ---KPTQSYVPKIFGFK
037 UniRef90_A0A2I0X454_94_284        ----NIDGAYFGTTFPHLFLMSY-PH---IKPQ---KASQRYTPRVFGFK
038 UniRef90_UPI00092FBD2C_80_270     ----NTNGAYFGTTFPHLFLMTY-EQ---LKPQ---KPSQKYVPRVFGFK
039 UniRef90_F0ZXH6_18_205            ----HIDGAYFGTTFPHLLLITY-PE---LIPT---KPPQQYVPKIYGFK
040 UniRef90_A0A1U8IV83_84_274        ----NIDGAYFGATFPHLFLMTY-GH---LKPQ---KATQNYIPRVFGFK
041 UniRef90_A0A261XW74_26_208        ----AIDGAYFGSSFPHMLFQVY-PN---LVPA---KGTDRYVPRIFGFK
042 UniRef90_A0A162PQW6_12_196        ----SIDGAYFGTSMPHMLFQVH-PT---YLPA---KSHERYIPRIFGFK
043 UniRef90_A0A7S3CG89_21_212        ----NNDGAYFGTTFPHLFLMTY-SH---LRYA---KQASKYCPKVFGFK
044 UniRef90_A0A162UUP8_10_199        ----AIDGAYFGTSLPHMLFQIH-PA---YLPS---KTNERYVPRIFGFK
045 UniRef90_A0A7R9Y293_54_246        ----NIDGACFGTTFPHLFLMTY-GAT--IKPK---KPAQCYVPRIFGFK
046 UniRef90_UPI00142E405F_77_267     ----NIDGAYFGTTFPHLFLMTY-GH---LKPL---KATQNYVPRVFGFK
047 UniRef90_A0A0K9Q617_79_270        ----NIDAAYFGATFPHLFLMTY-GN---VVPQ---KPSQNYVPRIYGFK
048 UniRef90_A0A6P5GZT7_89_278        ----NMDGAYIGTTFPHLYLMTY-PS---TKPA---KAVQKYVPRVFGFK
049 UniRef90_A0A1R1PTJ3_11_196        ----SIDGAYFGTSFPHMLLQAY-PS---LKPPP-LTEAQRYVPKIFGFK
050 UniRef90_A0A7J7KUM0_133_320       ----NMDGAYFGTTFPHLYLMSY-PT---AKPA---KPVQSYVPRVFGFK
051 UniRef90_F0W6R1_98_288            ----QIDGAYFGSTFPHMFLMTH-SY---LVPA---PPTQTYTPRVFGYK
052 UniRef90_A0A4P9XR61_13_199        ----AMDGAYFGTTFAHMLFMVY-PH---LVPR---PTTERYVPKIFGFK
053 UniRef90_A0A7S0ES88_51_226        ----ATDGAYFGTSFPHLLLQTF-PE---LFPP---SP------------
054 UniRef90_A0A420Y4K1_15_197        ----SMDGAYFGTSFAHILFQVY-PA---LVPT---KSADRYVPRVYGFK
055 UniRef90_A4RSN9_26_212            ----NTDGAYFGTTFPHLFLMSF-KH---LQTP---KQTDLYEPRIFGFK
056 UniRef90_A0A642UK79_17_202        ----NIDGAYFGTSFPGMLFQVY-PH---LIPK---RTNARFVPKIFGFK
057 UniRef90_A0A1R3S306_15_197        ----SIDGAYFGTSFHSMLFQVY-PA---LLPE---KSVRRYEPRIFGFK
058 UniRef90_A0A183REZ2_109_233       ----HTDGAYFGTGFPHMLFLVH-PE---YRPK---RASKQFTARY---
059 UniRef90_A0A067N327_22_192        ----SIDGAYFGSTFPHMLFMVY-PN---MIPS---KS------------
060 UniRef90_A0A507CIU8_18_202        ----MIDGAYFGTSLPHLLLQVY-PN---LIPQ---KSTERYVPRIFGFK
061 UniRef90_A0A7D8Z2K1_14_184        ----SIDGAYFGTTFPHMLFMAY-PQ---MIPA---K-------------
062 UniRef90_A0A0F4ZAU1_16_198        ----VLDGAFFGASFHNIFFQVY-PP---LVPP---KGTQRYTPRVYGFK
063 UniRef90_A0A7S1VF37_12_200        ----SLDGAFFGTTLPHLLLSTY-PQ---LVPS---LPTQSYTPRIFGFR
064 UniRef90_A0A0L0H4P4_15_200        ----AIDGAHFGTTFPHLLLQAY-PN---LMPA---KTPKRYVPKIFGFK
065 UniRef90_A0A6H0Y5P8_15_197        ----SIDGAYFGSSFHNILFQVY-PA---LQPQ---KTQRRYEPRLYGFR
066 UniRef90_A0A7R9BIZ0_390_519       ----HTDGSFFGTGFPHVLFMVH-PE---YRPK---RPTNQYIARR---
067 UniRef90_A0A3E2HRT2_15_197        ----SIDGAYFGTSFHNIIFQVY-PA---LIPT---KSYDRYVPRIYGFK
068 UniRef90_A0A6A6V4N0_15_197        ----VIDGAYFGTSFHNILFQVY-PA---MLPP---KTRRRYEPRVFGFK
069 UniRef90_A0A6A6YKN2_15_197        ----SIDGAYFGTSFHNILFQVY-PA---FIPP---KTQRRYEPRVFGFR
070 UniRef90_A0A2J5HQ92_16_198        ----SIDGAYFGSSFHSMMFQVY-PA---LLPE---KSIRRYEPRIFGFR
071 UniRef90_A0A0J9XL38_11_196        ----TIDGAYFGTSFPAMFFQVF-PT---IIPT---KPKERYVPKIFGFK
072 UniRef90_A0A2D3V5L0_22_206        ----SIDGAYFGTSFHNILFQVY-PT---LLPQ---KTQRRYEPRIYGFR
073 UniRef90_M5BRL5_5_172             ----SIDGAYFGTTFPHMLLMGY-PH---MIPS---K-------------
074 UniRef90_A0A3N4L0N9_15_197        ----AIDGAYFGASFPHILFQVY-PG---LIPP---KCNERYTPRCFGFK
075 UniRef90_A0A397J4Z1_55_250        ----NVDGAFFGTTFPHLLFQQY-PD---LLPN---IQTKIYEPKIFGFK
076 UniRef90_A0A6J3MH45_20_208        ----SIDGAYFGTSFHNILFQVY-PT---LQPR---KTQRRYEPRIYGFR
077 UniRef90_A0A2C5YX06_1078_1260     ----AIDGAYFGTSFHNIIFQVY-PT---LVPS---KVVDRYVPRVYGFK
078 UniRef90_UPI0018755BD5_76_247     ----SIDGAYFGTTFPHLLFLVY-PA---IIPP---K-------------
079 UniRef90_A0A4R0R889_49_217        ----SIDGAYFGTSFPHLLFLVY-PT---LIPP---K-------------
080 UniRef90_A0A0P1BJJ4_107_281       ----GVDGAFFGTTFPHLLFQSF-RE---MAPS-----------------
081 UniRef90_A0A137PBH0_17_198        ----NLDGAYFGTTFPHMLLQIY-SH---IVPA---KSNERYVPRIYGFK
082 UniRef90_A0A4U0WMK8_5_182         ----AIDGAYFGTSFHNILFQVY-PA---MMPQ---RTQRRYEPRIYGFR
```

```
083 UniRef90_A0A1Y2F9D6_30_220      ----AIDGAYFGTTFPHLFFMTF-PE---LLPF---ATGSIYDPCIFGFK
084 UniRef90_A0A0P8Y6J6_18_200      ----LLDGAMFGSSFPHMFLMQL-PA---LRPQ---PPKEKYVARLYGFQ
085 UniRef90_A0A2T9Z2A7_16_201      ----TIDGAYFGASFPHMLLQMY-PA---IKTQI-SEPMQRYVPKIFGFK
086 UniRef90_A0A1Y1YBP8_15_197      ----VIDGAYFGTSFHNILFQVY-PA---MLPP---KSQRRYEPRVFGFK
087 UniRef90_A0A0G2ESH3_15_197      ----SIDGAYFGTSFHNILFQVY-PA---LIPE---KSKRRYEPKVFGFR
088 UniRef90_A0A4V1J5H5_5_195       ----SVDGAYFGTSFPHIFFMSF-PG---FGDF--DRTSEYYIPRIFGFK
089 UniRef90_A0A4S8LIP9_20_193      ----SIDGAYFGTTFPHLLFLVY-PT---LIPP---KS------------
090 UniRef90_A0A1D8PNI6_16_200      ----AIDGAYFGTSFPAMFFQNF-PN---TVPI---HAKETYVPRVFGFK
091 UniRef90_A0A448YSS2_19_203      ----SIDGAFFGTSFPAMFLQNY-PE---LVPT---HNVDVYIPKIFGFQ
092 UniRef90_A0A2N1JDB9_48_222      ----GIDGAFFGTTFPHLLMQCY-RD---LAPS-----------------
093 UniRef90_A0A0W4ZMB2_44_237      ----NIDGAFFGTTFPHLFFQIY-PE---YKPTILQKCFRVYQPKIYGFK
094 UniRef90_A0A1D2VL05_15_200      ----QIDGAFFGTSFPGMFFQTF-PN---LVPP---HPTKRYIPRVFGFQ
095 UniRef90_K0KFC4_18_201          ----LIDGAFFGTSFPGMFLQAF-PH---LVPQ---HSTERYVPKIFGFN
096 UniRef90_A0A4P9ZSB4_16_199      ----NVDGAYFGTGLPHMIMQAY-ST---LVPQ---KSAARYEPKIFGFK
097 UniRef90_A0A7S2RTJ5_91_283      ----GIDGAFFGRTFAHLFLMVM-KT---NIPP--TPSEAPFVPTIFGYR
098 UniRef90_UPI00053A2101_52_240   ----NIDGAYFGTTFSHQFMMTY-EF---IRPE---TVSQSYVPRVFGFN
099 UniRef90_A0A1E3PJQ7_14_200      ----NIDGAYFGANFPAMLMQTF-AN---IIPA---KPKEIYVPKIFGFK
100 UniRef90_A0A0W0FK04_40_210      ----GVDGAFFGTTFAHLFFQTY-RE---LAP------------------
101 UniRef90_K2LYQ1_23_212          ----TLDGAFWGTTFPHLFIMLM-RKN-GLVVG---KPKKHYVPRIYGFR
102 UniRef90_A0A120K193_34_221      ----GIDGAYFGTSFPGMFLQAF-PE---VVPK---HPTKRYVPKVFGFE
103 UniRef90_A0A1G4JQF6_19_211      ----SIDGAFFGTSFPGMFLQAF-PE---MVPT---HPVKRYVPKIFGFE
104 UniRef90_A0A7G2C1K6_19_220      ----SLDGAFWGTTFPHLLLLQM-RDNPSKTIP---KPVQRYVPRIYGFR
105 UniRef90_A0A4R5XEN6_51_221      ----GVDGAFFGTTFAHLFFQTF-PQ---FAPA-----------------
106 UniRef90_R4X9E9_30_221          ----HVEGANFGTTFPHLFFMTF-PE---LLPP--ARDTDIYRAKIYGFK
107 UniRef90_A0A1Y2FJU8_15_197      ----NIDGAYFGTTFPHMLLQLN-SA---MMPA---KSEERYVPKIFGFR
108 UniRef90_A0A1W4WCC7_11_192      ----LLDGAMFGTGFPHMFFMQL-PD---LRPD---PPKEKYVARLYGFQ
109 UniRef90_A0A6P4GHA8_18_195      ----LLDGAMFGTGFPHMFFMQL-PS---MRPH---PPKEKYVARLYGFK
110 UniRef90_A0A0C7N2A5_19_211      ----AIDGAYFGTSFAGMFLQAF-PE---MVPK---HPVQRYVPKIFGFD
111 UniRef90_A7TQ13_22_208          ----SIDGAYFGKSFPGMLLQTF-PE---FVPK---HPIRRYIPSIFGFD
112 UniRef90_A0A0M4E7Q4_7_195       ----MLDGANFGRSFPHMFFMQL-TE---LLPS---PPKEQYTPRIYGFQ
113 UniRef90_A0A1X2GUL9_10_193      ----NLDGAYFGTTFAHLLLQSD-PT---LIND---TSFQRYTPRIYGFK
114 UniRef90_UPI000510F195_87_251   ----HTYASFFSPSPSRPFLV-----------------------------
115 UniRef90_A0A1B9HCU7_34_203      ----SVDGAFFGTTFAPLFFQQY-PE---LH-------------------
116 UniRef90_A0A1A6A1S9_31_200      ----SVDGAFFGTTFSPLFFQQY-PE---L--------------------
117 UniRef90_A0A4Q1BBE6_39_208      ----GVDGAFFGTSFAALFFQTY-PE---FL-------------------
118 UniRef90_A0A642VBA1_50_236      ----NIDGAYFGTTFAGLFLKTF-PE---IEQECSQRRQKQFELKIYGFR
119 UniRef90_A0A1E5RB29_103_291     ----NLDGSFFGTTFPGIFLQQY-PK---LVPV---KPIDNYVPKINGFE
120 UniRef90_A0A060TJ45_17_202      ----NIDGAFFGTSFPGLFLQTF-PD---IEQRAARKRAKQFELKIYGFK
121 UniRef90_A0A376B670_70_257      ----TIDGAFFGTSFPGMFLQQY-PQ---LVPK---HPIDNYVPKIFGFE
122 UniRef90_A0A507DW98_129_303     ----SIDGAFFGTTFPHFLWLTY-PD---SLPP-----------------
123 UniRef90_A0A0K3AQN1_24_214      ----LIDGAFFGTTFAHLFLMQH-QS---LISR---GPTYYYVPKIHGFK
124 UniRef90_G0QMJ3_13_196          ----DIDGCYFGVSFPQTLLLTY-PD---LNPQ---KNNQSYVPRIYGFR
125 UniRef90_A0A2E5QG28_55_190      --------------------------------------------------
126 UniRef90_W1Q7C8_11_202          ----NIDGAFFGTSFAGLFIKMF-PE---IERQCQQRLNKQYELKLFGFR
127 UniRef90_A0A1G4JSI2_22_213      ----CLEGAFWGTSFPGVFLKHF-KE---LEDYVDRKTKETYQLKVFGFR
128 UniRef90_R1E1U7_15_210          ----YIDGAFFGSTYPHLFLQTF-RE---VRPP---PPTQRYVPRVFGFK
129 UniRef90_C5M199_253_444         ----HLDGAYFGASLPSLFFQTL-PK---LIPA---EVPAYFEPRVFGFK
130 UniRef90_A0A0A1U1B9_13_198      ----SLDGSYFGTTFPHLLMLNV-QG---I------QEAQKYKPTVFGFL
131 UniRef90_L1LFG9_30_230          ----YIDGAFYGTTFAHLFLMVN-ES---IIPR---GPAYYYVPKVYGYR
132 UniRef90_A0A1X1BNS6_15_204      ----DVDGAFFGRSFPHMFLLSF-PH---LIPE---EPPIPYVPRIFGFK
133 UniRef90_A7ART0_26_226          ----LIDGAYFGTTFAHLFLMVH-DT---LVTR---GPTYYYVPQIYGFK
134 UniRef90_J9IFP4_26_217          ----NIDGAYFGTSFPHMFLKHY-PE---AVIL--PPKIYLYEPKISGFK
135 UniRef90_A0A433P780_55_189      ----AIDGAYYGTSFPHMLFQVH-PT---LLPQ---KTGERYTPRIFGFK
136 UniRef90_A0A7S1T6P4_10_137      ----NIDGAYFGTTFPHLLLMTY-PV---LRPP---QPGVQYVPRVFGFK
137 UniRef90_F4PAV0_23_209          ----SLDGSSFGTSFPSFLFLTM-PH---LVP------------------
138 UniRef90_Q3SEC1_7_197           ----DVDGAYFGCSFPSIFLMNFIQE---IQIP---ANNQTYVPRVFGFR
139 UniRef90_A0A2A9M617_91_280      ----DIDGAFFGPSFPHIFLQTF-PN---LVPL---DVPVPYQSKIFGFR
140 UniRef90_A0A3M7MUJ5_976_1133    ----AVDGAFFGTTFGCLFFMTF-PD---LDISP----------------
141 UniRef90_A0A0K3C7J8_5_146       ----SIDGAYFGSTFAHMLFMVY-PG---MIPS---KSVERIRPRIFGFQ
142 UniRef90_A0A507C5M5_148_333     ----RIDGAFFGTTFPHVLFLSF-PE---LIP------------------
143 UniRef90_A0A1R1PPR8_25_196      ----SVDGAFFGTTFPHLFFETF-QE---YWPT---DSTALYIPKIFGFS
144 UniRef90_A0A084G399_1151_1315   ----SIDGAYFGTSFHNILFQVY-ST---LVPT---KSADRYVPRVYGFK
145 UniRef90_A0A0R3QRT7_7_137       ----EIDGAFFGTGFPHMFFFEH-PE---LKPR---RSVINFVPRYVTF-
146 UniRef90_A0A0L6X1G9_47_219      ----GVDGAFFGTTFAHLFFQSY-RE---LAPAPFWKPQSSGSSLIYGFK
```

```
147 UniRef90_A0A6S7GGY9_40_229      ----PTDGASFGTSLPQMFFMAY-PA---LHPS---PTLGHYVPKLFGFK
148 UniRef90_B8BZE6_1_179           ----TVDGAAFGTTFPHLFLMTF-NN---LVPDP-LPSDSAYIPRVFGFR
149 UniRef90_J9IF68_28_225          GCQIFLDGAYFGTSFPSVYLMNY-QR---DVPD---YGPQTFIPTIYGFK
150 UniRef90_B4IGD2_3_165           ----KLDGAMFGTSFPHLFFMQF-PM---LRPQ---PPVEKYVPRIHGFR


001 Input_pdb_SEQRES_A              IH
002 UniRef90_D3PJV2_1_193           IH
003 UniRef90_A0A443SMC7_9_201       IH
004 UniRef90_A0A7R8WGP2_1_193       IH
005 UniRef90_A0A7R9KER7_1_193       IH
006 UniRef90_K7FHH3_1_194           IH
007 UniRef90_A0A1I8ISK0_1_192       VH
008 UniRef90_A0A6P9F8P8_1_185       --
009 UniRef90_A0A315WGG2_1_182       --
010 UniRef90_A0A0B2UX36_1_192       IH
011 UniRef90_A0A210QLV3_15_188      IH
012 UniRef90_A0A6A4WWH4_1_175       IH
013 UniRef90_A0A673UYT7_1_167       --
014 UniRef90_A0A090MVV2_1_191       VH
015 UniRef90_A0A0N5AZ67_43_214      IH
016 UniRef90_A0A0D2U6T3_2_190       IH
017 UniRef90_A0A0N4UWY4_7_187       IH
018 UniRef90_A0A1W4V542_1_191       IH
019 UniRef90_A0A6P4JVR0_1_191       IH
020 UniRef90_A0A5F8HIB5_36_187      --
021 UniRef90_A0A7S4IQT0_22_213      VH
022 UniRef90_A0A6J0MJ32_91_282      LH
023 UniRef90_A0A388JRV3_116_307     LH
024 UniRef90_A0A0D6QXG8_112_303     IH
025 UniRef90_A0A151ZRX5_21_208      IH
026 UniRef90_UPI0019298C3E_89_278   IH
027 UniRef90_A0A7S3PWQ0_98_288      I-
028 UniRef90_A0A6I8V063_1_191       IH
029 UniRef90_A0A250WT55_67_256      LH
030 UniRef90_A0A6P3Z3J9_56_245      LH
031 UniRef90_M4CKX0_70_259          VH
032 UniRef90_A0A1U8N6I0_93_282      IH
033 UniRef90_A0A1U8N824_86_276      IH
034 UniRef90_A0A0D2QNY2_22_212      IH
035 UniRef90_UPI0007EFC624_83_271   VH
036 UniRef90_A0A6J0MMZ8_88_277      VH
037 UniRef90_A0A2I0X454_94_284      IH
038 UniRef90_UPI00092FBD2C_80_270   LH
039 UniRef90_F0ZXH6_18_205          IH
040 UniRef90_A0A1U8IV83_84_274      IH
041 UniRef90_A0A261XW74_26_208      IH
042 UniRef90_A0A162PQW6_12_196      VH
043 UniRef90_A0A7S3CG89_21_212      IH
044 UniRef90_A0A162UUP8_10_199      VH
045 UniRef90_A0A7R9Y293_54_246      IH
046 UniRef90_UPI00142E405F_77_267   LH
047 UniRef90_A0A0K9Q617_79_270      IH
048 UniRef90_A0A6P5GZT7_89_278      LH
049 UniRef90_A0A1R1PTJ3_11_196      IH
050 UniRef90_A0A7J7KUM0_133_320     IH
051 UniRef90_F0W6R1_98_288          VH
052 UniRef90_A0A4P9XR61_13_199      IH
053 UniRef90_A0A7S0ES88_51_226      --
054 UniRef90_A0A420Y4K1_15_197      MH
055 UniRef90_A4RSN9_26_212          I-
056 UniRef90_A0A642UK79_17_202      IH
```

057	UniRef90_A0A1R3S306_15_197	V	H
058	UniRef90_A0A183REZ2_109_233	-	-
059	UniRef90_A0A067N327_22_192	-	-
060	UniRef90_A0A507CIU8_18_202	I	H
061	UniRef90_A0A7D8Z2K1_14_184	-	-
062	UniRef90_A0A0F4ZAU1_16_198	V	H
063	UniRef90_A0A7S1VF37_12_200	I	H
064	UniRef90_A0A0L0H4P4_15_200	V	H
065	UniRef90_A0A6H0Y5P8_15_197	V	H
066	UniRef90_A0A7R9BIZ0_390_519	-	-
067	UniRef90_A0A3E2HRT2_15_197	V	H
068	UniRef90_A0A6A6V4N0_15_197	V	H
069	UniRef90_A0A6A6YKN2_15_197	V	H
070	UniRef90_A0A2J5HQ92_16_198	V	H
071	UniRef90_A0A0J9XL38_11_196	L	H
072	UniRef90_A0A2D3V5L0_22_206	V	H
073	UniRef90_M5BRL5_5_172	-	-
074	UniRef90_A0A3N4L0N9_15_197	T	H
075	UniRef90_A0A397J4Z1_55_250	V	-
076	UniRef90_A0A6J3MH45_20_208	V	H
077	UniRef90_A0A2C5YX06_1078_1260	V	H
078	UniRef90_UPI0018755BD5_76_247	-	-
079	UniRef90_A0A4R0R889_49_217	-	-
080	UniRef90_A0A0P1BJJ4_107_281	-	-
081	UniRef90_A0A137PBH0_17_198	I	-
082	UniRef90_A0A4U0WMK8_5_182	V	H
083	UniRef90_A0A1Y2F9D6_30_220	V	-
084	UniRef90_A0A0P8Y6J6_18_200	L	H
085	UniRef90_A0A2T9Z2A7_16_201	V	H
086	UniRef90_A0A1Y1YBP8_15_197	M	H
087	UniRef90_A0A0G2ESH3_15_197	V	H
088	UniRef90_A0A4V1J5H5_5_195	V	-
089	UniRef90_A0A4S8LIP9_20_193	-	-
090	UniRef90_A0A1D8PNI6_16_200	L	H
091	UniRef90_A0A448YSS2_19_203	L	H
092	UniRef90_A0A2N1JDB9_48_222	-	-
093	UniRef90_A0A0W4ZMB2_44_237	I	-
094	UniRef90_A0A1D2VL05_15_200	L	H
095	UniRef90_K0KFC4_18_201	L	H
096	UniRef90_A0A4P9ZSB4_16_199	I	H
097	UniRef90_A0A7S2RTJ5_91_283	I	H
098	UniRef90_UPI00053A2101_52_240	L	H
099	UniRef90_A0A1E3PJQ7_14_200	I	H
100	UniRef90_A0A0W0FK04_40_210	-	-
101	UniRef90_K2LYQ1_23_212	L	-
102	UniRef90_A0A120K193_34_221	L	H
103	UniRef90_A0A1G4JQF6_19_211	L	H
104	UniRef90_A0A7G2C1K6_19_220	V	-
105	UniRef90_A0A4R5XEN6_51_221	-	-
106	UniRef90_R4X9E9_30_221	V	-
107	UniRef90_A0A1Y2FJU8_15_197	V	H
108	UniRef90_A0A1W4WCC7_11_192	I	H
109	UniRef90_A0A6P4GHA8_18_195	I	H
110	UniRef90_A0A0C7N2A5_19_211	L	H
111	UniRef90_A7TQ13_22_208	L	H
112	UniRef90_A0A0M4E7Q4_7_195	V	H
113	UniRef90_A0A1X2GUL9_10_193	I	-
114	UniRef90_UPI000510F195_87_251	-	-
115	UniRef90_A0A1B9HCU7_34_203	-	-
116	UniRef90_A0A1A6A1S9_31_200	-	-
117	UniRef90_A0A4Q1BBE6_39_208	-	-
118	UniRef90_A0A642VBA1_50_236	I	-
119	UniRef90_A0A1E5RB29_103_291	L	H
120	UniRef90_A0A060TJ45_17_202	I	-

121	UniRef90_A0A376B670_70_257	L	H
122	UniRef90_A0A507DW98_129_303	-	-
123	UniRef90_A0A0K3AQN1_24_214	V	-
124	UniRef90_G0QMJ3_13_196	I	Y
125	UniRef90_A0A2E5QG28_55_190	-	-
126	UniRef90_W1Q7C8_11_202	I	-
127	UniRef90_A0A1G4JSI2_22_213	I	-
128	UniRef90_R1E1U7_15_210	I	-
129	UniRef90_C5M199_253_444	V	H
130	UniRef90_A0A0A1U1B9_13_198	V	-
131	UniRef90_L1LFG9_30_230	I	-
132	UniRef90_A0A1X1BNS6_15_204	V	H
133	UniRef90_A7ART0_26_226	V	-
134	UniRef90_J9IFP4_26_217	L	H
135	UniRef90_A0A433P780_55_189	I	H
136	UniRef90_A0A7S1T6P4_10_137	L	H
137	UniRef90_F4PAV0_23_209	-	-
138	UniRef90_Q3SEC1_7_197	I	Y
139	UniRef90_A0A2A9M617_91_280	V	H
140	UniRef90_A0A3M7MUJ5_976_1133	-	-
141	UniRef90_A0A0K3C7J8_5_146	V	H
142	UniRef90_A0A507C5M5_148_333	-	-
143	UniRef90_A0A1R1PPR8_25_196	I	-
144	UniRef90_A0A084G399_1151_1315	V	H
145	UniRef90_A0A0R3QRT7_7_137	-	-
146	UniRef90_A0A0L6X1G9_47_219	V	-
147	UniRef90_A0A6S7GGY9_40_229	I	H
148	UniRef90_B8BZE6_1_179	V	H
149	UniRef90_J9IF68_28_225	V	-
150	UniRef90_B4IGD2_3_165	L	H

1	2	3	4	5	6	7	8	9
Variable				Average				Conserved

X - Insufficient data - the calculation for this site was performed on less than 10% of the sequences.
