# Supplementary figures and images for: Predictive functional, statistical and structural analysis of CSNK2A1 and CSNK2B variants linked to neurodevelopmental diseases
Source: Front Mol Biosci. 2022 Oct 13;9:851547. doi: 10.3389/fmolb.2022.851547 (PMC9608649; doi:10.3389/fmolb.2022.851547)

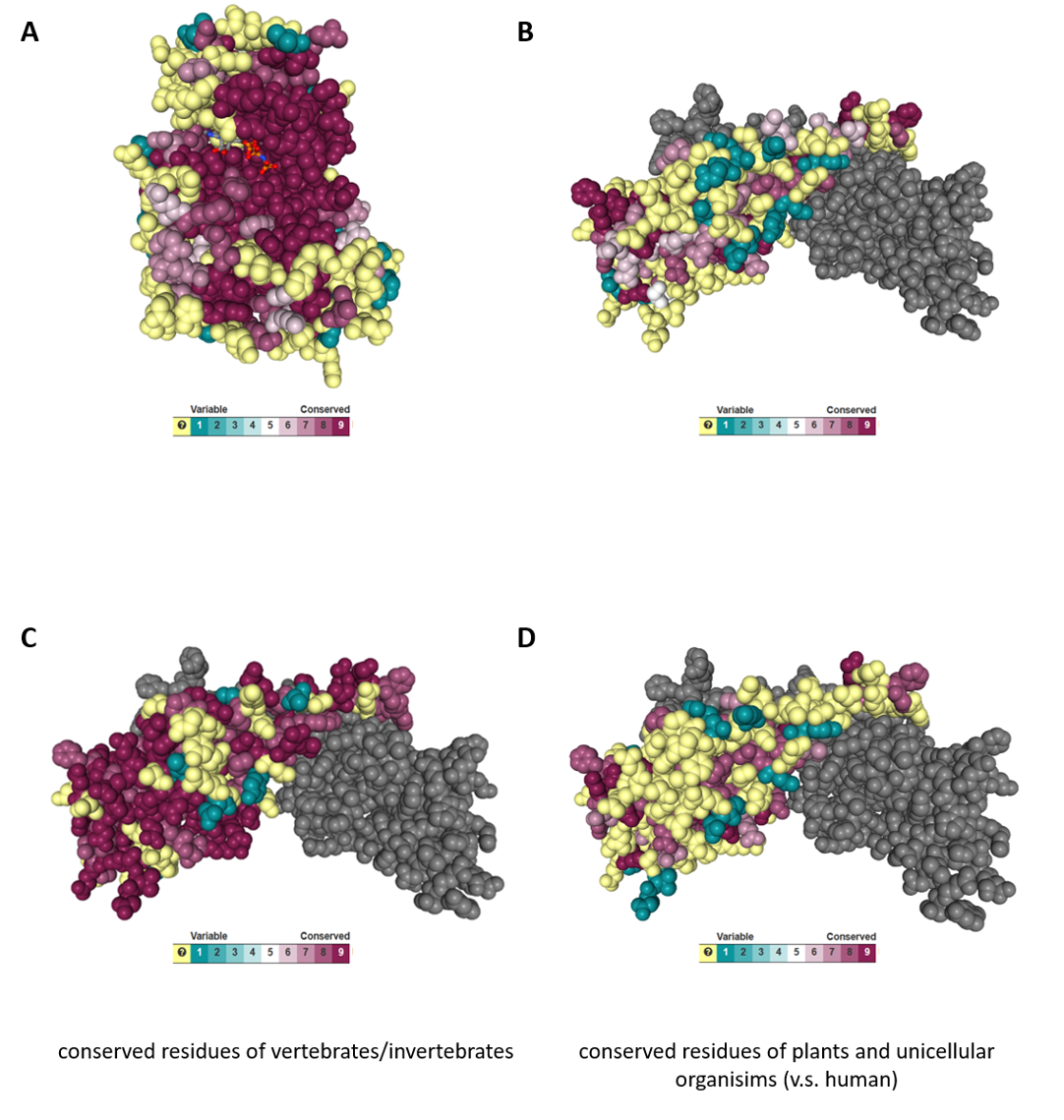

Supplement: Supplementary file 5 [file Image5.tiff]
