# Supplementary material for: Predictive functional, statistical and structural analysis of CSNK2A1 and CSNK2B variants linked to neurodevelopmental diseases
Source: Front Mol Biosci. 2022 Oct 13;9:851547. doi: 10.3389/fmolb.2022.851547 (PMC9608649; doi:10.3389/fmolb.2022.851547)
Supplement: Supplementary file 6 [file DataSheet3.PDF]

ConSurf Color-Coded MSA

001 Input_pdb_SEQRES_A	SGPV-PSRARVYTDVNTHRP--REYWDYES-HVVEW-----G-NQDDYQL
002 UniRef90_A0A060X538_2_334	SGPV-PSRARVYTDVNTQRP--REYWDYES-HVVEW-----G-NQDDFQL
003 UniRef90_UPI000719C52A_2_332	--PL-PSRARMYVDANTHRP--REYWDYES-HVVEW-----G-KQEDYQL
004 UniRef90_A0A7R8XAU3_2_332	--PL-PSRARVYAEVNSHRP--REYWDYES-HVIEW-----G-QQDDYQL
005 UniRef90_V3ZFM5_3_333	--PL-PSRARVYPDINTHKP--KDYWDYES-HVVEW-----G-NQDDYQI
006 UniRef90_UPI00193A3309_3_330	---T-PSRARVYPEVNTHRP--RDYWDYES-HVVDW-----GRSQDDYQL
007 UniRef90_A0A1Q3F4A5_3_329	---L-PSSARVYADVNSHKP--REYWDYEN-YVVDW-----A-NQDDYQL
008 UniRef90_A0A4W3H6I6_2_321	AGPV-PSRARVYTDVNTHKP--REYWDYES-HVVEW-----G-NQDDYQL
009 UniRef90_UPI000C6E8BEA_2_341	--PL-PSRARVYADVNSHRP--REYWDYES-HVVEW-----G-NQDDYQL
010 UniRef90_A0A0L0BLN9_116_444	---L-PSAARVYTDVNAHKP--DEYWDYEN-YVVDW-----A-NQDDYQL
011 UniRef90_A0A183IEN2_2_329	--PV-PSRARVYAEINSKKP--REYWDYES-HVIEW-----G-NIDDYQL
012 UniRef90_A0A5N4B3X1_3_332	---V-ASLARVYADVNSHKP--REYWDYEN-YIVDW-----G-LQDNYQL
013 UniRef90_A0A556VUA8_53_369	TGPV-TSRSRVYPDINTQRP--REYWDYES-HVVEW-----G-NQDDYQL
014 UniRef90_A0A1I8HE15_2_329	--PL-PSKARVYADANAVRE--RDYWDYEA-LVVEW-----G-NQDDYQI
015 UniRef90_F6RN85_7_328	-----GSKARVYADVNSLRS--REYWDYEA-HVPNW-----G-NQEDYQL
016 UniRef90_UPI0011E4E1F1_2_326	--PL-ASKARVYYDVNMSKP--REYWDYEN-SIIEW-----G-NHDDYQL
017 UniRef90_A0A0P7V994_7_333	-----GNKARVYADVNTAKN--REYWDYEA-HVPTW-----S-NQEDYQL
018 UniRef90_A0A3Q4N194_2_309	SGPV-PSRARVYTEVNTHRP--REYWDYES-HVVEW-----G-NQDDFQL
019 UniRef90_A0A0N5ANV2_2_328	--PI-PSRSLIYAEVNSEKP--REYYEYEC-HMIEW-----G-NIDDYQL
020 UniRef90_A0A0M3HMV3_2_324	--PI-PSRARVYAEVNQQKP--REYWDYEC-HMIEW-----G-NIDDYQL
021 UniRef90_A0A2R7W6M3_5_330	-----PNRARVYADVNTNKV--SDYWDYES-YTISW-----G-QQDDIHL
022 UniRef90_A0A368G0G8_241_571	--------------VNPSKP--REYWDYES-HVIEW-----G-NIDDYQL
023 UniRef90_A0A5F4C0P0_224_555	------PTVRFYTDVCPART--RRAAAWRS-VCPPWDGRRPS-NQDDYQL
024 UniRef90_A0A671N1V7_3_326	-GPVAGSKSRVYADVNTLKS--REYWDYEA-HVPTW-----S-NQEDFQL
025 UniRef90_A0A667Y761_7_331	-----SSKARVYTDVNTQKS--REYWDYDA-HVPSW-----S-NQDNYQL
026 UniRef90_I0Z462_2_325	------SRARVYADVNVTRP--KEYWDYEA-LSIQW-----G-NQDDYEV
027 UniRef90_A0A4W5MUS6_7_326	-----GSKSRVYADVNTLKS--REYWDYEA-HVPSW-----S-NQEDYQL
028 UniRef90_UPI000C1CFB1C_2_322	------SQARVYTDINVQRP--SDYWDYES-LTVQW-----G-EQDDYEV
029 UniRef90_A0A200QFN8_2_323	------SKARVYTDINVLRP--KEYWDYES-LAVQW-----G-DQDDYEV
030 UniRef90_A0A6I9QC06_84_406	-----ASKARAYADINVHRP--NEYWDYES-LTVQW-----G-EQDDYEV
031 UniRef90_A9NVF1_102_427	---V-TSKARVYADVNVVRP--KEYWDYEA-LTVQW-----G-DQEDYEV
032 UniRef90_A0A6S7M9R1_74_394	------SKSRVYADVNVVRP--REYWDYEA-LTVQW-----G-DQDDYEV
033 UniRef90_A0A5J4ZXR3_110_433	---A-PSKARVYTDVNVIRP--KEYWDYES-LTLQW-----G-EQDDYEV
034 UniRef90_A0A0D2WV07_2_326	-----TSRARVYKDVNVHRP--REYWDYES-LVIQW-----G-SQEKYQI
035 UniRef90_A0A2P6Q0W9_107_428	-----MSRARVYADVNVQRP--RDYWDYES-FVLQW-----G-VQDEYEV
036 UniRef90_A0A2J6LRB0_100_422	-----SSKARVYTDVNVVRP--KEYWDYES-LAVQW-----G-EQDDYEV
037 UniRef90_A0A4S8ISY9_72_395	---A-PSRARVYADINVHRP--KDYWDYES-LTVQW-----G-EQDDYEV
038 UniRef90_A0A1U8LZW6_92_416	---F-PSKSRVYADVNVIRP--KEYWDYES-LIVQW-----GREQDDYRV
039 UniRef90_K3XBI1_15_338	-----MSRARVYCDVNETRP--REYWDYDN-LNVTW-----G-NQDNYEV
040 UniRef90_E1ZRP6_14_337	---M-TSLARVYADTNVNNP--KEYWDYEN-LAIDW-----G-SQDDYEV
041 UniRef90_A0A2I0AFK3_70_391	-----ASKARVYPDVNVVRP--KDYWDYES-LTVQW-----G-DQDDYEV
042 UniRef90_UPI000F7CDB20_80_401	-----TSKARVYADINVVRP--KEYWDYES-LSVQW-----G-EQDDYEV
043 UniRef90_G7K3U9_82_403	-----SSKARIYADVNVVRP--KEYWDYES-LAVQW-----G-EQDDYQV
044 UniRef90_A0A663MTN9_26_324	-------------------------------HGDDK-----S-NQDDYQL
045 UniRef90_A4S5X1_14_334	---V-ASRARVYAEANDEKA--REYWDYEA-LSVQW-----G-EQDEYEV
046 UniRef90_A0A183LBY6_9_327	--PL-SSKARVYADVNLNRP--REYWDYES-YEVKW-----G-DQDDYQI
047 UniRef90_A0A1J9RPL1_99_422	--PA-PSMARVYADVNANMP--RSYWDYDS-VNISW-----G-VLENYEV
048 UniRef90_D5GJ91_2_320	--------ARVYADVNQHMP--RTYWDYDS-VNIPW-----G-VLENYEV
049 UniRef90_A0A4W4DPG2_3_315	-GPV-ASKARVYVDVNTLKS--KDYWDYEA-HVPSW-----S-NQDDYQL
050 UniRef90_A0A7S2RD37_2_324	------SHARVYADVNVHRP--REYWDYEN-LDVKW-----G-GQEDYEV
051 UniRef90_A0A367JQL9_12_336	------SIARCYADVNAKMP--ASYWDYDN-LQVEW-----G-NQEKYEI
052 UniRef90_UPI001924B863_29_350	---I-MSKSRVYTDINVLRP--KEYWDYES-LRVQW-----G-DQDDYDV
053 UniRef90_A0A367KWU7_10_331	------SIARCYADVNTNMP--SSYWDYDN-LQVEW-----G-NQENYEI
054 UniRef90_A0A0D2AMV0_2_320	--------ARVYADVNANMP--RSYWDYDS-VNISW-----G-VLENYEV
055 UniRef90_A0A1Y1WVY1_2_324	------SIAKVYANANELQP--REYWDYDN-LLITW-----G-DQDKYEI
056 UniRef90_Q8TG13_2_324	--------ARVYADVNQNMP--RAYWDYDS-VNISW-----G-VLENYEV
057 UniRef90_A0A0F8B0Y9_1_322	---M-PSLARVYPDVNQNMP--RAYWDYDA-VNISW-----G-ILENYEV
058 UniRef90_A0A7S1NI55_6_324	----------YKHDVNASRP--KDYWDYES-LQVTW-----N-AQDNYEV
059 UniRef90_A0A383VX45_13_335	---C-PSISRVYADINDKRP--PEYWDYES-LNIQW-----G-EQDNYEV
060 UniRef90_I1C1X5_16_338	--------TRCYADVNSKLP--PSYWDYDN-LQVEW-----G-SQENYEI

```
061 UniRef90_A0A364LA26_2_320      --------ARVYADVNTHMP--RSYWDYDS-VNISW-----G-ALENYEV
062 UniRef90_A0A1X7RU70_2_320      --------ARVYADVNQQMP--RAYWDYDS-VNISW-----G-VLENYEV
063 UniRef90_A0A067BWS1_2_324      ------SLARVYADACESRP--REYWDYDN-LSVAW-----G-DQDAYEV
064 UniRef90_A0A0P9FCQ6_6_328      ------SIARIYPDANERFG--ESWHNYDA-LMVAW-----G-IQDNYEV
065 UniRef90_A0A0K3CS88_10_335     --PA-RSIARVYADANSSRP--KSWWNYDE-LM--------G-IQDNYEV
066 UniRef90_A0A4S4KUV1_8_328      ------SVSRVYADVNGKLG--PSWYEYDN-LQVQW-----G-AQDHYEI
067 UniRef90_UPI000511B7B1_2_322   ------SKAHVYVDVNVQHP--RDYWVYES-VTLQW-----G-EQDDYEV
068 UniRef90_A0A5C3QYQ7_5_326      -----KSVSRIYPDVNSKLG--PSWHEYDN-LQVQW-----G-SQDHYEI
069 UniRef90_J4GNM1_5_326          -----KSVARVYPDVNAKLG--SSWHEYDN-LQVQW-----G-PQDHYEI
070 UniRef90_A0A1B6QHX1_78_406     -GAV-ASVARVYADANAQRP--KEYWDYES-LDIQW-----G-EQDGYEV
071 UniRef90_E3Q428_2_328          --------ARVYADVNQNMP--RSYWDYDS-VNISW-----G-VLENYEV
072 UniRef90_A0A161HHJ2_5_327      ---K-PSVSRVYADYNQRMP--NSYWDYDN-VSILW-----G-TLQNYEI
073 UniRef90_A0A0C2TUQ3_4_328      -SPV-KSVARVYQDVNTRLG--PSWYEYDN-LQVQW-----G-SQDHYEI
074 UniRef90_UPI000644E1AF_26_344  ------NKARIYCDVNYNKP--KDYWNYEA-LNVEW-----G-SQDDFEV
075 UniRef90_F2U7H7_13_336         ------SVAKVYADVNAHKP--REYWDYEA-LALQW-----G-NPEPYQI
076 UniRef90_A0A1E3Q523_2_312      -------------------P--AAYSDYDS-YQIQW-----G-EIENYEI
077 UniRef90_A0A446UN67_26_313     -------------------------------------------EQDDYEV
078 UniRef90_A0A1E4RU21_6_330      ---V-TSVSKVYADVLATKP--QAYWDYEN-INIKW-----N-GQENYEI
079 UniRef90_A5DCV5_5_327          -NPV-SSVSKVYTDVLASKP--QSYWDYEN-INIKW-----N-SQDNYEI
080 UniRef90_L8GRW8_10_332         ---V-KSVARVYPDINQKRP--PEYSDYEN-HVVQW-----G-GQDDYEI
081 UniRef90_A3GID5_8_329          ---V-TSISRVYSDVLSTKP--QAYWDYDD-LNIKW-----N-SQDNYEI
082 UniRef90_A0A2V1AT92_3_322      ---V-SSVSRVYTDALATKP--QAFWDYES-TNVKW-----N-SQEKYEV
083 UniRef90_A0A642UHU8_5_327      ---V-TSVARVYADVLSHKP--QSYWDYDD-LQIQW-----N-SQENYEI
084 UniRef90_UPI001036488A_60_387  -----MSKARVYSDVNVLRP--RDYWDYES-HTVQW-----G-DQDDYEV
085 UniRef90_A0A448YKC4_4_323      ------SISRVYADVNEHKP--QSYWDYEN-VSIFW-----G-PQYNYEI
086 UniRef90_UPI001900E851_4_329   ------SISRVYADINEHKP--QSYWDYEN-VSIFW-----G-PQYTYEI
087 UniRef90_A0A4P9ZDD8_3_324      ---V-SSVSKVYTDVLAYKP--QAYWDYEN-VNIRW-----N-SQDKYEV
088 UniRef90_UPI00064AE4FC_6_334   -PPL-PSRARVYADVNTRQP--PEFWDHES-YVVKW-----G-NIRDYKI
089 UniRef90_A0A7S3CI51_5_325      ---------KFYANVCQEKD--AEYSDYEN-FEVKF-----G-CMENYEI
090 UniRef90_UPI00098E15C8_59_372  ---A-PSKSRVYSDVNVVRP--KEYWDYES-FGLQW-----G-DQDEYEV
091 UniRef90_A5E6J5_9_319          --------------VLSTKP--TSYYDYES-MSIEW-----N-DQKNYEI
092 UniRef90_A0A0S4KFI2_14_334     ----------RYARVNVDKP--RDYWDYES-IPIQW-----N-SPERYEI
093 UniRef90_A0A4Y7PMM1_7_330      -----PSVARAYADVNRQLG--PSWYEYDN-LHVHW-----G-QQDNYEV
094 UniRef90_A0A674DLQ6_2_238      SGPV-TSRSRVYPDVNTQRP--REYWDYES-HVVEW-----G-NQDDYQL
095 UniRef90_A0A367YK45_12_333     ---V-PSVSRVYSDVLASKP--QEYWDYDD-INIKW-----N-PQENYEI
096 UniRef90_Q6BUZ4_8_327          -----YSVARVYADANSNRP--TEYWDYET-HKIEW-----G-QIKNYEI
097 UniRef90_A0A7S3F0G7_17_281     --PQ-AMGARWHSDVNVRRT--KEYWDYES-LQVVW-----G-DQDHYEV
098 UniRef90_A0A0C3HJB5_2_320      --------ARIYADVNQQMP--QAYWDYDS-VSIPW-----G-VLENYEV
099 UniRef90_A0A1R2D4Q4_3_325      ------RRARHYADVLSTKD--PDYYKYES-LSIKW-----N-SPENYEI
100 UniRef90_UPI0004F4A9E2_17_332  -----------YARVNVDKP--REYWDYES-VRIQW-----S-SPDRYEI
101 UniRef90_A0A4P6XGL7_6_324      -----YSVARVYADVNQQKP--QEYWDYEA-HTIEW-----G-SIRNYEI
102 UniRef90_A0A6P6RSI1_108_381    --------------------------------------------------
103 UniRef90_A0A4U0VRL3_43_361     ----------LYAHVNAELG--PSWWHYDD-LQLRW-----G-TQQQYEI
104 UniRef90_K0KNM3_9_329          ---V-YSVARVYADANEKRP--KEYWDYEN-YDIEW-----G-QISNYEI
105 UniRef90_A0A2X0MBX0_6_345      ---A-PSISRVYADANTERG--ATWYDYDQ-LAISW-----G-NQEQYEI
106 UniRef90_A0A7S1VNL6_68_384     --------SKVHADVNEKLG--RSYYDYDV-VHVEW-----G-NVDDYEI
107 UniRef90_A0A2H9TP39_34_357     ------SIARVYADANNRLP--SSYWDYET-LNIRW-----G-SQDIYEV
108 UniRef90_A0A315WAV6_110_366    SSPA-SSKARVYTDVNTQKN--REYWDYDA-HVPNW-----S-NQDNYQL
109 UniRef90_UPI001386DBE1_151_468 -----GSRARVQAERNSLRS--REYWDXEA-RVPSW-----G-IQEGYQL
110 UniRef90_A0A5D2A803_51_293     ---V-MSKSRVYSDVNVLRP--KEYWDYES-LTVQW-----G-DQDGYEV
111 UniRef90_A0CQX8_4_329          -------KPRLYGEANLKKR--PSYFEFEN-IDINY-----G-QLDNYEI
112 UniRef90_T1EXU0_70_294         --------------------------------------------------
113 UniRef90_A0A7H9B7U0_15_336     ---V-YSVAKVYRDACEKRP--QEYWDYEQGVTIEW-----G-KISYYEI
114 UniRef90_A0A482VYB7_21_342     -----VSTSKVYSDVFTSKP--SSYFEYES-YNSPT-----D-NIENYSL
115 UniRef90_A0A1D2VEG0_6_324      ------SVSKVYANACEDRP--REYWDYED-HKIFW-----S-NISNYEI
116 UniRef90_A0A0D0B3X6_2_320      --------ARIYAGANAQLG--RSWYDYDN-FRIEW-----S-SPERYEI
117 UniRef90_A0A1G4MEF4_15_334     ---V-YSVARVYKDACEKRP--QEYWDYEQGVTIDW-----G-RISNYEI
118 UniRef90_A0A2V0NTF2_16_339     --AL-RTVARCYADVAAGQP--KEYWN-DA-CTVEW-----G-AMEQYEV
119 UniRef90_A0A4Y9YFI0_2_320      --------ARVYTDVNAVLG--PAWYEFES-TKIEW-----N-VPDRYEI
120 UniRef90_W6KIF7_13_329         ----------RYSRVNVSKP--REYWDYES-LKVEW-----N-SPDRYEV
121 UniRef90_A0A0C3NKK6_2_320      --------ARVYADVNAELG--PGWYEYEH-LHIEW-----N-VPDNYEI
122 UniRef90_A0A0D7AGR9_2_320      --------SRIYADVNRGLG--KDWYDYEN-FTIEW-----S-EPDRYEV
123 UniRef90_G0VHQ2_23_343         ---I-YSIAKVYASECERRP--QQYWDYEQCCEIDW-----G-TLTDYEI
124 UniRef90_UPI000EA961A9_2_209   SGPV-PSRARVYTDVNTQRP--CEYWDYES-HVVEW-----G-NQDDYQL
```

```
125 UniRef90_A0A165QAJ6_2_320      --------ARVYADVNALLG--PAWYDYES-MKIEW-----N-VPDRYEI
126 UniRef90_A0A6J1ACU4_2_223      ------SRARVYANVNVHRP--RDYWDYES-LTVQW-----G-DQDHYEV
127 UniRef90_A0A146IBX2_2_322      --------ARVYADANTIRD--KSWSEYHK-FRIEW-----S-APDRYEI
128 UniRef90_A0A1E5RA31_17_339     ---V-YSVARVYTHACTDKP--REYWDYEQCTQLKF-----G-DITRYEI
129 UniRef90_J9HLC0_29_343         ------------KNVCVERP--HDFSYYQT-LEIPW-----G-NQNDYEV
130 UniRef90_A0A183CIH8_805_1008   --PI-PSRARVYADINVNRP--REYWDYEC-HQIRW-----G-NIDDYQL
131 UniRef90_A0A1M2V5N7_3_298      ---------------------------------VDW-----N-VPDRYEI
132 UniRef90_A0A1B7TD73_22_342     -----YSVARVHADYCTEQP--REYWDYEQSVSFDF-----G-EISQYEV
133 UniRef90_A0A4P9VZ92_1_208      --------------------------------------------------
134 UniRef90_W0T7F4_129_366        --------------------------------------------------
135 UniRef90_UPI000711954B_2_325   ------NRSRVYADVISTKP--SDYCDYEH-MNIQW-----N-SPDRYEL
136 UniRef90_A0A1C7MA44_2_304      --------ARVYSDVNARLG--PSWYDYES-MRIDG-----N-KPDRYEI
137 UniRef90_C5DWN1_128_366        --------------------------------------------------
138 UniRef90_A0A7H9B7A0_128_371    --------------------------------------------------
139 UniRef90_W5KQ02_73_295         --------------------------------------------------
140 UniRef90_A0A6P6YBM1_25_347     ---------KYYTTYCEDQG--AEYWDYEV-YKVDW-----Y-TPDGIEL
141 UniRef90_A0A196S5L7_3_325      -------VSRVYADVVSNMP--PDYADYDS-VNITW-----E-SPDRYQI
142 UniRef90_A0A7S3FVA9_17_301     -----------YANVCSNHPLGSQYTDYENDYEVVF-----G-PQDDYEF
143 UniRef90_A2FBX9_18_336         ------TTAKVYADACEKRG--PEWSEIDN-WTLPT-----A-SPEPYEI
144 UniRef90_UPI0010FB2831_6_323   -------EARGYENYLLKQP--ESYYDYEN-YEISM-----G-SIDEYQI
145 UniRef90_Q8SRU0_6_317          -------VARSNADVNEKKD--ERYWNYEC-YTITT-----G-SIEKYQI
146 UniRef90_E9ACB1_46_365         ----------------RQRG--VSYWDYKN-ARVDF-----NANLAPYEL
147 UniRef90_W6KPE3_52_367         -----------------ELP--KAYWNYED-FEIEF-----NNDLEAYDA
148 UniRef90_A0A1J4JEQ9_9_332      ---L-ISVSKVYANVNEEKG--PEWYDHRQ-FHPQW-----N-PPDPYYL
149 UniRef90_G9N8S2_27_316         -------------------------------------------SSDAYEI
150 UniRef90_A0A4Z1TD09_8_331      -----PVRPKVNMHALAQFP--PSYAFHDT-LPCVY-----G-RQDDYEI
```

```
001 Input_pdb_SEQRES_A             VRKLGRGKYSEVFEA----INITN-NEKVVVKILKPVKKKKIKR--EIKI
002 UniRef90_A0A060X538_2_334      VRKLGRGKYSEVFEA----INITN-NEKVVVKILKPVKKKKIKR--EIKI
003 UniRef90_UPI000719C52A_2_332   VRKLGRGKYSEVFEA----INITN-NDKCVVKILKPVKKKKIKR--EIKI
004 UniRef90_A0A7R8XAU3_2_332      VRKLGRGKYSEVFEA----VNITN-NEKCVVKILKPVKKKKIKR--EIKI
005 UniRef90_V3ZFM5_3_333          VRKLGRGKYSEVFES----VNITN-NEKCVIKILKPVKKKKIKR--EIKI
006 UniRef90_UPI00193A3309_3_330   IRKLGRGKYSEVFEA----INITN-NDKVVVKILKPVKKKKIKR--EIKI
007 UniRef90_A0A1Q3F4A5_3_329      VRKLGRGKYSEVFEA----IKITN-NEKCVVKILKPVKKKKIKR--EIKI
008 UniRef90_A0A4W3H6I6_2_321      VRKLGRGKYSEVFEA----INITN-NEKVVVKILKPVKKKKIKR--EIKI
009 UniRef90_UPI000C6E8BEA_2_341   VRKLGRGKYSEVFEA----VNITN-NEKCVVKILKPVKKKKIKR--EIKI
010 UniRef90_A0A0L0BLN9_116_444    VRKLGRGKYSEVFEA----INITN-NEKCVVKILKPVKKKKIKR--EIKI
011 UniRef90_A0A183IEN2_2_329      VRKLGRGKYSEVFEG----VNVNN-DDKVVIKILKPVKKKKIKR--EIKI
012 UniRef90_A0A5N4B3X1_3_332      IRKLGRGKYSEVFEE----INVTN-NEKGVVKILKPVKKKKIKR--EIKI
013 UniRef90_A0A556VUA8_53_369     VRKLGRGKYSEVFEA----VNITN-NEKVVVKILKPVKKKKIKR--EIKI
014 UniRef90_A0A1I8HE15_2_329      VRKLGRGKYSEVFEG----INISN-NEKVVIKVLKPVKKRKIKR--EIKI
015 UniRef90_F6RN85_7_328          VRKLGRGKYSEVFEA----INITN-NERVVVKILKPVKKKKIKR--EVKI
016 UniRef90_UPI0011E4E1F1_2_326   VRKLGRGKYSEVFEG----VNITT-NEKCVVKILKPVKKKKIKR--EIKI
017 UniRef90_A0A0P7V994_7_333      VRKLGRGKYSEVFEA----INITN-NEKVVIKILKPVKKKKIKR--EIKI
018 UniRef90_A0A3Q4N194_2_309      VRKLGRGKYSEVFEA----INITN-NEKVVVKILKPVKKKKIKR--EIKI
019 UniRef90_A0A0N5ANV2_2_328      LRKLGRGKYSEVFEG----VKAPL-EEKCVIKILKPVKKKKIKR--EIKI
020 UniRef90_A0A0M3HMV3_2_324      IRKLGRGKYSEVFEG----VKAPM-DQKCVIKILKPVKKKKIKR--EIKI
021 UniRef90_A0A2R7W6M3_5_330      VRKLGRGKYSEVFEA----INIKT-NDKCVVKILKPVKKKKIKR--EIKI
022 UniRef90_A0A368G0G8_241_571    VRKLGRGKYSEVFEG----VKITT-EEKVVVKILKPVKKKKIKR--EIKI
023 UniRef90_A0A5F4C0P0_224_555    VRKLGRGKYSEVFEA----INITN-NERVVVKILKPVKKKKIKR--EVKI
024 UniRef90_A0A671N1V7_3_326      VRKLGRGKYSEVFEA----ININN-NEKVVVKILKPVKKKKIKR--EIKI
025 UniRef90_A0A667Y761_7_331      VRKLGRGKYSEVFEA----INVSN-NEKVVVKILKPVKKKKIKR--EIKI
026 UniRef90_I0Z462_2_325          VRKVGRGKYSEVFEG----INTIN-NQKCIIKILKPVKKKKIKR--EIKI
027 UniRef90_A0A4W5MUS6_7_326      VRKLGRGKYSEVFEA----ININN-NEKVVVKILKPVKKKKIKR--EIKI
028 UniRef90_UPI000C1CFB1C_2_322   VRKVGRGKYSEVFEG----INIAN-NEKCIIKILKPVKKKKIKR--EIKI
029 UniRef90_A0A200QFN8_2_323      VRKVGKGKYSEVFEG----INATS-SEKCIIKILKPVKKKKIKR--EIKI
030 UniRef90_A0A6I9QC06_84_406     VRKVGRGKYSEVFEG----VHCAN-NEKCIIKILKPVKKKKIKR--EIKI
031 UniRef90_A9NVF1_102_427        VRKVGRGKYSEVFEG----VNSIN-NERCIIKILKPVKKKKIKR--EIKI
032 UniRef90_A0A6S7M9R1_74_394     VRKVGRGKYSEVFEG----INVNS-NEKCVIKILKPVKKKKIKR--EIKI
033 UniRef90_A0A5J4ZXR3_110_433    VRKVGRGKYSEVFEG----IHCTN-NEKCIIKILKPVKKKKIKR--EIKI
034 UniRef90_A0A0D2WV07_2_326      VRKIGRGKYSEVFEG----INITN-DAKCVIKILKPVKKKKIKR--EIKI
```

```
035 UniRef90_A0A2P6Q0W9_107_428    VRKVGRGKYSEVFEG----INVTN-NEKCVIKILKPVKKKIKR--EIKI
036 UniRef90_A0A2J6LRB0_100_422    VRKVGRGKYSEVFEG----LHSVN-DEKCIIKILKPVKKKIKR--EIKI
037 UniRef90_A0A4S8ISY9_72_395     VRKVGRGKYSEVFEG----VRATD-KEKCIIKILKPVKKKIKR--EIKI
038 UniRef90_A0A1U8LZW6_92_416     VRKVGRGKYSEVFEG----VHCTD-NEKCIIKILKPVKKKIKR--EIKI
039 UniRef90_K3XBI1_15_338         VRKIGRGKYSEVFEG----YNVTN-NSRCVIKILKPVKKKIKR--EIKI
040 UniRef90_E1ZRP6_14_337         VRKVGRGKYSEVFEG----VNTRN-NEKCIIKILKPVKKKIKR--EIKI
041 UniRef90_A0A2I0AFK3_70_391     VRKVGRGKYSEVFEG----VHSPD-GQPCIIKILKPVKKKIKR--EIKI
042 UniRef90_UPI000F7CDB20_80_401  VRKVGRGKYSEVFEG----VHCTD-NEKCVIKILKPVKKKIKR--EIKI
043 UniRef90_G7K3U9_82_403         LKKVGRGKYSEVFEG----VHCGN-DEKCIIKILKPVRKKIKR--EIKI
044 UniRef90_A0A663MTN9_26_324     VRKLGRGKYSEVFEA----INITN-NERVVVKILKPVKKKIKR--EVKI
045 UniRef90_A4S5X1_14_334         VRKVGRGKYSEVFEG----RRTES-GEKCVIKILKPVKKKIKR--EIKI
046 UniRef90_A0A183LBY6_9_327      VRKLGRGKYSEVFEG----LNITN-NEKCVIKVLKVNLGKSTWR--D---
047 UniRef90_A0A1J9RPL1_99_422     VRKIGRGKYSEVFEG----INIVN-YQKCVIKVLKPVKKKIKR--EIKI
048 UniRef90_D5GJ91_2_320          VRKIGRGKYSEVFEG----INITN-YQKTVIKVLKPVKKKIKR--EIKI
049 UniRef90_A0A4W4DPG2_3_315      VRKLGRGKYSEVFEA----ININS-NDRVVVKILKPVKKKIKR--EIKI
050 UniRef90_A0A7S2RD37_2_324      VKKIGRGKYSEVFDG----YNIRN-KQRCVVKILKPVKKKIKR--EIKI
051 UniRef90_A0A367JQL9_12_336     IRKVGRGKYSEVFQG----INITN-NEKCVIKALKPVKKKIKR--EIKI
052 UniRef90_UPI001924B863_29_350  ARKVGRGKYSEVFEG----INVNN-NERCIIKILKPVRKKIKR--EIKI
053 UniRef90_A0A367KWU7_10_331     IRKVGRGKYSEVFEG----IDIVS-NEKCVIKALKPVKKKIKR--EIKI
054 UniRef90_A0A0D2AMV0_2_320      VRKIGRGKYSEVFEG----INIVN-YQKCVIKVLKPVKKKIKR--EIKI
055 UniRef90_A0A1Y1WVY1_2_324      IRKIGRGKYSEVFEG----INITN-QQRCVIKVLKPVKKKIRR--EIKI
056 UniRef90_Q8TG13_2_324          VRKIGRGKYSEVFEG----INVVN-YQKCVIKVLKPVKKKIKR--EIKI
057 UniRef90_A0A0F8B0Y9_1_322      VRKIGRGKYSEVFEG----INVAN-YQKCVIKVLKPVKKKIKR--EIKI
058 UniRef90_A0A7S1NI55_6_324      VRKIGRGKYSEVFEG----VNTTS-NDKCVIKVLKPVKKKIKR--EIKI
059 UniRef90_A0A383VX45_13_335     VRKVGRGKYSEVFEG----VCVAN-SQKCIIKILKPVKKKIRR--EIKI
060 UniRef90_I1C1X5_16_338         IRKVGRGKYSEVFEG----INIAN-NEKCVIKALKPVKKKIKR--EIKI
061 UniRef90_A0A364LA26_2_320      VRKIGRGKYSEVFEG----INVVN-YQKCVIKVLKPVKKKIKR--EIKI
062 UniRef90_A0A1X7RU70_2_320      VRKIGRGKYSEVFEG----VNVVN-YQKCVIKVLKPVKKKIKR--EIKI
063 UniRef90_A0A067BWS1_2_324      SRKIGRGKYSEVFEG----VALAT-GAKCVIKILKPVKKKIKR--EIKI
064 UniRef90_A0A0P9FCQ6_6_328      VRKVGRGKYSEVFEG----VNVVN-EDKCVIKVLKPVKKKIKR--EIKI
065 UniRef90_A0A0K3CS88_10_335     VRKVGRGKYSEVFEG----VNIVN-DDKCVIKVLKPVKKKIKR--EIKI
066 UniRef90_A0A4S4KUV1_8_328      VRKVGRGKYSEVFEG----VNISN-EEKCIIKVLKPVKKKIKR--EIKI
067 UniRef90_UPI000511B7B1_2_322   VRKIGRGKYNEVFEG----VNVTK-NEKCVMKILKPIKKMKIKR--KIKI
068 UniRef90_A0A5C3QYQ7_5_326      VRKVGRGKYSEVFEG----VNVLT-DEKCIIKVLKPVKKKIKR--EIKI
069 UniRef90_J4GNM1_5_326          VRKVGRGKYSEVFEG----INIVN-EEKCIIKVLKPVKKKIKR--EIKI
070 UniRef90_A0A1B6QHX1_78_406     LRKVGKGKYSEVFEG----YRAGS-DERCVIKILKPVKKKIKR--EIKI
071 UniRef90_E3Q428_2_328          VRKIGRGKYSEVFEG----INVVN-YQKCVIKVLKPVKKKIKR--EIKI
072 UniRef90_A0A161HHJ2_5_327      VRKIGRGKYSEAFEG----INLAN-NGKCVIKVLKPVKKKIKR--EIKI
073 UniRef90_A0A0C2TUQ3_4_328      VRKVGRGKYSEVFEG----VNVVT-EDKCIIKVLKPVKKKIKR--EIKI
074 UniRef90_UPI000644E1AF_26_344  VRKIGRGKYSEVFEG----AHAKT-NEKCVIKVLKPVKKKIKR--EIKI
075 UniRef90_F2U7H7_13_336         TCKLGRGKYSEVFEG----IDTRT-NKTIVIKVLKPVKKKIKR--EIKV
076 UniRef90_A0A1E3Q523_2_312      VKKVGRGKYSEVFEG----VNIAN-QQKCIIKVLKPVKKKIKR--EVKI
077 UniRef90_A0A446UN67_26_313     VRKVGRGKYSEVFEG----FSVNN-SEKCVIKILKPVKKKIKR--EIKI
078 UniRef90_A0A1E4RU21_6_330      IKKLGRGKYSEVFLG----IELTR-GEKVVIKVLKPVKRKIKR--EISI
079 UniRef90_A5DCV5_5_327          IKKLGRGKYSEVFLG----VDLKK-GEKCVIKVLKPVKRKIKR--EISI
080 UniRef90_L8GRW8_10_332         ICKVGRGKYSEVFES----MNVRD-NEKCIIKVLKPVRDKIRR--EIKI
081 UniRef90_A3GID5_8_329          IKKLGRGKYSEVFLG----IDLSK-QEKVVIKVLKPVKRKIKR--EISI
082 UniRef90_A0A2V1AT92_3_322      VKRLGRGKYSEVFLG----VDITS-GDKLVIKVLKPVKRKIKR--EISI
083 UniRef90_A0A642UHU8_5_327      IKKLGRGKYSEVFLG----IDLTS-GAKCVIKVLKPVKRKIKR--EISI
084 UniRef90_UPI001036488A_60_387  VRKVGRGKYSEVFEG----INVNN-NERCVIKILKPVKKKVTLWWGITY
085 UniRef90_A0A448YKC4_4_323      TRKVGRGKYSEVFEG----YQMST-GTKIIIKALKPIKKKIKR--EIKI
086 UniRef90_UPI001900E851_4_329   TRKVGRGKYSEVFEG----YQITT-GTKIIIKALKPIKKKIKR--EIKI
087 UniRef90_A0A4P9ZDD8_3_324      IKKLGRGKYSEVFLG----VDLTS-GSKCVIKVLKPVKRKIKR--EISI
088 UniRef90_UPI00064AE4FC_6_334   GKVIGRGGYSEVFEA----VKILT-SEKVAIKVLRPTKEEKIKR--EIKI
089 UniRef90_A0A7S3CI51_5_325      IKKIGRGKYSEVYEG----INTLN-NERIVIKILKPVKKTKIRR--EIKI
090 UniRef90_UPI00098E15C8_59_372  VRKVGRGKYSEVFEG----VHSTN-EEKCVIKILKPVKKKVKR--EVKI
091 UniRef90_A5E6J5_9_319          LRKIGRGKYSEVFLG----LDLVK-GQKVVIKVLKPVKRKIKR--EISI
092 UniRef90_A0A0S4KFI2_14_334     VKKIGRGKYSDVFLG----WDCET-ERDVVIKVLKPVKKKIMR--ELKI
093 UniRef90_A0A4Y7PMM1_7_330      VRKIGRGKYSEVFEA----INVVT-EEKCVVKVLKPVKKQKVKR--EIKI
094 UniRef90_A0A674DLQ6_2_238      VRKLGRGKYSEVFEA----INITN-NEKVVVKILKPVKKKIKR--EIKI
095 UniRef90_A0A367YK45_12_333     IKKLGRGKYSEVFLG----LDLAK-GEKVVIKVLKPVKRKIKR--EISI
096 UniRef90_Q6BUZ4_8_327          ISKIGRGKYSEVFQG----INIYN-DEPCVIKVLKPVKMKKIYR--EVKI
097 UniRef90_A0A7S3F0G7_17_281     VRKVGRGKYSEVFEG----WNVTN-NTKVIIKILKPVKKKIKR--EIKI
098 UniRef90_A0A0C3HJB5_2_320      LRKIGRGKYSDVFEG----VSNIN-YQKCAIKVLKPVKLEKIKR--QIKV
```

```
099 UniRef90_A0A1R2D4Q4_3_325        IKKIGHGKYSEVFEG----IDISN-NQKIVIKVLKPVKKEKYKR--EVKI
100 UniRef90_UPI0004F4A9E2_17_332    VEKIGRGKYSDVFLG----WDTKT-RHQVVIKVLKPVKKKILR--ELKV
101 UniRef90_A0A4P6XGL7_6_324        VSKIGRGKYSEVFEG----VNVRN-DEPCVIKVLKPVKMKKIFR--EVKI
102 UniRef90_A0A6P6RSI1_108_381      -------------------------VV-GQRCVIKILKPVKKKIRR--EVKI
103 UniRef90_A0A4U0VRL3_43_361       TRKLGRGKYSEVFEG----VDVYNSNELIVIKVLKPIKKKKVKG--ELKV
104 UniRef90_K0KNM3_9_329            ISKIGRGKYSEVFQG----LNIVN-NQPCVIKVLKPVKIKKIYR--EVKI
105 UniRef90_A0A2X0MBX0_6_345        VNRLGRGKYSEVFGG----VDVCHGYRKICIKVLKPIKKKKVKR--ELKI
106 UniRef90_A0A7S1VNL6_68_384       VRKIGRGKYSEVFEG----VAVAS-GDRVVIKVLKPVKKKKIRR--EIKI
107 UniRef90_A0A2H9TP39_34_357       VEKVGRGKYSEVFSG----VNVAK-NERCIVKVLKPVRSKKIKR--EVKI
108 UniRef90_A0A315WAV6_110_366      VRKLGRGKYSEVFEA----INVTN-NEKVVVKILKPVKKKKIKR--EIKI
109 UniRef90_UPI001386DBE1_151_468   VRKLGRGKNSEEFEA----MHIPD-NE-XVVKILKPVKKKKIKR--DVKI
110 UniRef90_A0A5D2A803_51_293       IRKVGRGKYSEVFEG----KNVNN-NERCVIKILKPVKKKKIKR--EIKI
111 UniRef90_A0CQX8_4_329            VRKIGRGKYAEVFEG----INVNM-LQKVVIKALKPIRQKKIKR--EIKI
112 UniRef90_T1EXU0_70_294           --------------------------------------------------
113 UniRef90_A0A7H9B7U0_15_336       ISKIGRGKYSEVFSG----KSVLN-NQPCVIKVLKPVKMKKIYR--ELKI
114 UniRef90_A0A482VYB7_21_342       IRKIGQGKYSLVFEG----LHDVK-KDSVVVKLLKPIKKPKIKR--EIKI
115 UniRef90_A0A1D2VEG0_6_324        ISKIGRGKYSEVFDG----INILN-QQPCAIKVLKPVKLKKIFR--EVKI
116 UniRef90_A0A0D0B3X6_2_320        VDRVGGGKYSEVFEG----LDTAN-SETCIIKVLKPVALKKINR--EIKI
117 UniRef90_A0A1G4MEF4_15_334       VNKVGRGKYSEVFRG----KCVLT-DQYCVIKVLKPVKLKKIYR--ELKI
118 UniRef90_A0A2V0NTF2_16_339       LQQLGKGKYGEVFEG----IDLDA-GARCVVKIMRPVKEQRLKR--EIKI
119 UniRef90_A0A4Y9YFI0_2_320        TRRIGGGKYSDVFEG----VDSSN-EEIVVIKVLKPVAKRKIKR--EIKI
120 UniRef90_W6KIF7_13_329           ERRIGRGKYSNVFLG----HDTEL-SRRVSIKVLKPVKKKKILR--ELKI
121 UniRef90_A0A0C3NKK6_2_320        IKRVGGGKFSEVFQG----IDTSN-EETCVIKVLKPIAKRKIRR--EIKV
122 UniRef90_A0A0D7AGR9_2_320        IQRVGGGRYSEVFTG----MDMAN-HERCIIKVLKPVAPKKIKR--EIKV
123 UniRef90_G0VHQ2_23_343           INKIGRGKYSEVFRG----KHILN-DVSCVIKVLKPVKLRKIHR--ELKI
124 UniRef90_UPI000EA961A9_2_209     VRKLGRGKYSEVFEA----INITN-NEKVVVKILKPVKKKKIKR--EIKI
125 UniRef90_A0A165QAJ6_2_320        IQRIGGGKYSDVFEG----VDSSN-EDAVVIKVLKPVAKRKIKR--EIKV
126 UniRef90_A0A6J1ACU4_2_223        VRKVGRGKYSEVFEG----ISVIN-NEKCIIKILKPVKKKKIKR--EIKI
127 UniRef90_A0A146IBX2_2_322        IRRLGGGKYSEVFEG----VDTVN-NDRCVIKVLKPVASHKIKR--EIKV
128 UniRef90_A0A1E5RA31_17_339       LCKIGRGKYSEVFRG----CFADEFKKLCVIKVLKPVKMKKIYR--ELKI
129 UniRef90_J9HLC0_29_343           VHKLGRGKYSEVFEG----VNLKN-NQKCVLKILKPVRTEKIYR--EIKI
130 UniRef90_A0A183CIH8_805_1008     VRKLGRGKYSEVFEG----VMLPT-EQKVVIKILKPVKKKKIKR--EIKI
131 UniRef90_A0A1M2V5N7_3_298        VRRIGGGRYSEVFEG----IDSSN-EESCVIKVLKPVAKKKIKR--EIKI
132 UniRef90_A0A1B7TD73_22_342       KTKIGRGKYSEVFRG----CFAQDYNKLCVIKILKPVKMKKIYR--EFHI
133 UniRef90_A0A4P9VZ92_1_208        --------------------------------------------------
134 UniRef90_W0T7F4_129_366          --------------------------------------------------
135 UniRef90_UPI000711954B_2_325     ISSVGRGKFSEVFKA----YDKVT-GDYVSIKHLKPIRRKKIRR--EIKV
136 UniRef90_A0A1C7MA44_2_304        VRRIGGGKYSEVFEG----IDSFN-EETCVIKVLKPVAKKKIKR--EIKI
137 UniRef90_C5DWN1_128_366          --------------------------------------------------
138 UniRef90_A0A7H9B7A0_128_371      --------------------------------------------------
139 UniRef90_W5KQ02_73_295           --------------------------------------------------
140 UniRef90_A0A6P6YBM1_25_347       INKIGRGKYSDVFDGVMKRVDSEE-EDPVIVKILKPVRKKKIKR--EISI
141 UniRef90_A0A196S5L7_3_325        VKKVGRGKYSEVFKG----IDMQT-GEPISIKYLKPVRFKKIKR--EIKI
142 UniRef90_A0A7S3FVA9_17_301       MMKLGRGRYSEVFSA----IDLLS-NKKVVVKILKPVKRMKVRR--EIHI
143 UniRef90_A2FBX9_18_336           ADWVGTGKYSDVFTA----YK--G-DTKVAIKILKPVRPQKYNR--EAKI
144 UniRef90_UPI0010FB2831_6_323     YKKIGRGKYSEVFEG----RVLLK-ESKCIIKVLKPVRESKIHR--EILI
145 UniRef90_Q8SRU0_6_317            YQRMGRGKYSEVFEG------RKD-REKIVIKALKPVRKAKICR--EVLI
146 UniRef90_E9ACB1_46_365           LQKIGRGKYSEVFRG----RNRNN-GCLCVLKLLKPVRYQKILR--EISI
147 UniRef90_W6KPE3_52_367           HQKIGQGKYSEVFRG----WNRTT-GRPCVLKILKPVRYRKIQR--EISI
148 UniRef90_A0A1J4JEQ9_9_332        LKKVGRGKYSTVFKA----LQNK--KRDVAIKILVPLDPKRYLR--EIKI
149 UniRef90_G9N8S2_27_316           VRKIGFGKHADIFEG----VRMID-LKRCIVKPAKEVGRLSIEQ--EIKI
150 UniRef90_A0A4Z1TD09_8_331        GRKLGYGKYSDVFEG----YCPET-DSSVVIKILKPVRKKKICR--EIKI


001 Input_pdb_SEQRES_A               LENLRGGPNIITLADIVKDPVSRTPALVFEHV---N--N-TDFK-QLY--
002 UniRef90_A0A060X538_2_334        LENLRGGPNVISLVDIVKDPVSRTPALVFEHV---N--N-TDFK-QLY--
003 UniRef90_UPI000719C52A_2_332     LENLNGGTNVITLLAVVKDPVSRTPALIFEHV---N--N-TDFK-QLY--
004 UniRef90_A0A7R8XAU3_2_332        LDNLRGGTNIINLQSVVKDPVSRTPALVFEHV---N--N-TDFK-QLY--
005 UniRef90_V3ZFM5_3_333            LENLRGGTNIITLLAIVKDPVSRTPALIFEHV---N--N-TDFK-QLY--
006 UniRef90_UPI00193A3309_3_330     LQNLRGGPNVITLLDIVKDPVSRTPALVFEHV---N--N-IDFK-QLY--
007 UniRef90_A0A1Q3F4A5_3_329        LENLRGGTNIITLLAVVKDPVSRTPALIFEHV---N--N-TDFK-QLY--
008 UniRef90_A0A4W3H6I6_2_321        LENLRGGPNIITLIDIVKDPVVS--------------------R-QLY--
```

```
009 UniRef90_UPI000C6E8BEA_2_341     LENLRGGPNIISLQAVVKDPVSRTPALIFEHV---N--N-TDFK-QLY--
010 UniRef90_A0A0L0BLN9_116_444      LENLRGGTNIITLLAVVKDPVSRTPALIFEHV---N--N-TDFK-QLY--
011 UniRef90_A0A183IEN2_2_329        LENLRNGTNVVSLIDVVKDPVSRTPALIFEYV---N--N-TDFK-QLY--
012 UniRef90_A0A5N4B3X1_3_332        LENLRGGTNIITLQAVVKDPVSRTPALVFEHV---N--N-TDFK-ELY--
013 UniRef90_A0A556VUA8_53_369       LENLRGGPNIITLLDIVKDPV----------------------------QLY--
014 UniRef90_A0A1I8HE15_2_329        LENLKGGTNIISLHGIVKDPVSRTPALIFEHV---N--N-TDFK-QLY--
015 UniRef90_F6RN85_7_328            LENLRGGTNIIRLLDTIKDPVSKTPALVFEYI---N--N-TDFK-QLY--
016 UniRef90_UPI0011E4E1F1_2_326     LEDLKGGTNIIALQAVVKDPVSRTPALIFEYV---N--N-VDFK-ILY--
017 UniRef90_A0A0P7V994_7_333        LENLRGGTNIIRLVDTVKDPVSRTPALVFECI---N--N-TDFK-ELY--
018 UniRef90_A0A3Q4N194_2_309        LENLRGGPNIISLIDIVKDPVSRTPALVFEHV---N--N-TDFK-QLY--
019 UniRef90_A0A0N5ANV2_2_328        LENLRGGTNIIDLLDVVKDPISRTPALVFEYV---N--N-IDFK-VLY--
020 UniRef90_A0A0M3HMV3_2_324        LENLRGGTNIISLLDVVKDPISRTPALIFEY--------------QLY--
021 UniRef90_A0A2R7W6M3_5_330        LENLRGGKNIINLQGVVKDPLSRTPALIFEYV---N--N-ADFK-ELY--
022 UniRef90_A0A368G0G8_241_571      LENLRGGTNVITLLDVVKDPISRTPALIFEYV---N--N-SDFK-QLY--
023 UniRef90_A0A5F4C0P0_224_555      LENLRGGTNIIKLIDTVKDPVSKTPALVFEYI---N--N-TDFK-QLY--
024 UniRef90_A0A671N1V7_3_326        LENLRGGTNIIQLMDTVKDPVVRCPSL-------------SSS-ELY--
025 UniRef90_A0A667Y761_7_331        LENLRGGTNIIRLVDTVKDPVSRTPALVFECI---N--N-TDFK-VLM--
026 UniRef90_I0Z462_2_325            LQNLCGGPNVIQLLDVVRDPQSKTPSLIFECV---N--N-TDFK-TLY--
027 UniRef90_A0A4W5MUS6_7_326        LENLRGGTNIIRLVDTVKDPVSRTPALVFECI---N--N-TDFK------
028 UniRef90_UPI000C1CFB1C_2_322     LQNLCGGPNIVKLLDIVRDSDSKTPSLIFEFV---N--S-TDFK-ILY--
029 UniRef90_A0A200QFN8_2_323        LQNLCGGPNIVKLLDIVRDQHSKTPSLIFECV---N--S-TDFK-VLY--
030 UniRef90_A0A6I9QC06_84_406       LQNLCGGPNIVKLLDIVRDQQSKTPSLIFEYV---N--N-TDFK-VLY--
031 UniRef90_A9NVF1_102_427          LQNLSEGPNIVKLLDIVRDQQSKTPSLIFEYV---N--N-TDFK-VLY--
032 UniRef90_A0A6S7M9R1_74_394       LQNLCGGPNVIKLLDIVRDQHSKTPSLIFEYV---N--S-TDFK-VLY--
033 UniRef90_A0A5J4ZXR3_110_433      LQNLCGGPNVVKLLDIVRDQQSKTPSLIFEYV---N--N-TDFK-VLY--
034 UniRef90_A0A0D2WV07_2_326        LQNLCGGTNIISLYDVVKDQQSKIPSLVFEHV---D--N-VDFK-VLY--
035 UniRef90_A0A2P6Q0W9_107_428      LQNLCGGPNIVKLLDIVRDELSKTPSLIFEHV---N--S-TDFK-VLY--
036 UniRef90_A0A2J6LRB0_100_422      LQNLCGGPNIIKLLDIVRDQQSKTPSLVFEYV---N--N-TDFK-VLY--
037 UniRef90_A0A4S8ISY9_72_395       LQNLCGGSNIVKLLDIVRDQQSKTPSLIFEYV---N--N-TDFK-VLY--
038 UniRef90_A0A1U8LZW6_92_416       LQNLCGGPNIVKLLDIVRDQQSKTPSLIFEYV---N--N-TDFK-VLY--
039 UniRef90_K3XBI1_15_338           LQNLAGGINIVQLLDIVRDPQSKTPSLVFEHV---N--A-TDFK-TLY--
040 UniRef90_E1ZRP6_14_337           LQNLFGGPNVIQLLDVVRDPQSKTPSLIFEYV---N--N-TDFKVVLY--
041 UniRef90_A0A2I0AFK3_70_391       LQNLCGGPNIVKLLDIVRDQQSKTPSLIFEYV---N--N-TDFR-VLY--
042 UniRef90_UPI000F7CDB20_80_401    LQNLCGGPNIVKLIDIVRDQQSKTPSFIFEYV---N--N-TDFK-VLY--
043 UniRef90_G7K3U9_82_403           LQNLCGGPNIVKLLDIVRDQQSKTPSLVFECV---N--N-TDFK-VLY--
044 UniRef90_A0A663MTN9_26_324       LENLRGGTNIINLIDTVKDPVSKTPALVFEYI---N--N-TDFK-VIT--
045 UniRef90_A4S5X1_14_334           LQNLAGGVNVIKLLDVVREPSTKTPSLVFEYV---N--N-TDFK-ALY--
046 UniRef90_A0A183LBY6_9_327        KCNIGKGSYCKTKVPDVKFFQSRTPALIFEHV---E--N-RDFK-ELY--
047 UniRef90_A0A1J9RPL1_99_422       LQNLSGGPNIVALLDVVRDNQSKTPSLIFEYV---N--N-TDFR-SLY--
048 UniRef90_D5GJ91_2_320            LQNLSGGPNIVALLDVVRDPQSKTPSLIFEYI---N--N-TDFR-SLY--
049 UniRef90_A0A4W4DPG2_3_315        LENLRGGTNIIRLVDTVKDPV----------------------------DLY--
050 UniRef90_A0A7S2RD37_2_324        LQNLCGGTNIIKLLDVVRDPQSKTPSLIFEYV---D--N-VDFK-TLY--
051 UniRef90_A0A367JQL9_12_336       LQNLAGGTNIVGLLDIVRDPISKTPAIIFEYV---N--N-TDFK-ILY--
052 UniRef90_UPI001924B863_29_350    LQNLCGGPNIVKLLDIVRDPRSKTLSLIFEYV---N--S-TDFK-VLY--
053 UniRef90_A0A367KWU7_10_331       LQNLAGGTNIVGLLDIVRDPISNTPAILFEYV---N--N-TDFK-TLY--
054 UniRef90_A0A0D2AMV0_2_320        LQNLSGGPNIVALLDVVRDNQSKTPSLIFEYV---N--N-TDFR-TLY--
055 UniRef90_A0A1Y1WVY1_2_324        LQNLSGGPNVVALLDIVRDPQSKTPSLITEYV---N--N-TDFK-VLY--
056 UniRef90_Q8TG13_2_324            LQNLAGGPNIVALLDVVRDSQSKTPSLIFEYV---N--N-TEFR-TLY--
057 UniRef90_A0A0F8B0Y9_1_322        LQNLSGGPNIVALLDVVRDPQSKTPSLVFECV---N--N-TDFR-TLY--
058 UniRef90_A0A7S1NI55_6_324        LQNLCGGPNIIKLLDTVRDPQSKTPSLIFEYV---N--N-TDFR-ILY--
059 UniRef90_A0A383VX45_13_335       LQNLSGGPNIIGLLDVVRDPESKTPSLVFEYV---N--N-TDFK-VLY--
060 UniRef90_I1C1X5_16_338           LQNLAGGVNIVGLLDIVRDPISKTPAIISEYV---N--N-TDFK-ILY--
061 UniRef90_A0A364LA26_2_320        LQNLSGGPNVVALLDVVRDNQSKTPSLVFEYI---N--N-TDFR-TLY--
062 UniRef90_A0A1X7RU70_2_320        LQNLSGGPNVIALLDVVRDSQSKTPSLIFEHV---N--N-TDFR-TLY--
063 UniRef90_A0A067BWS1_2_324        LQNLSGGPNIVKLLDVVRDPQSKTPSLITEYV---N--N-HDFK-TLY--
064 UniRef90_A0A0P9FCQ6_6_328        LQNLAGGTNVITLLDVVRDPQSKTPSLVFEHV---N--N-TDFK-VLY--
065 UniRef90_A0A0K3CS88_10_335       LQNLAGGENIINLYDVVRDPQSKTPSLVFEHV---N--N-TDFK-VLY--
066 UniRef90_A0A4S4KUV1_8_328        LQNLAGGPNVIALLDVVRDPSSKIPSLITEYI---H--N-VDFK-VLY--
067 UniRef90_UPI000511B7B1_2_322     LQNLCGGTNIVKLLDIVRDEHSKTPSLIFEYV---N--S-VDFK-VLY--
068 UniRef90_A0A5C3QYQ7_5_326        LQNLAGGPNVVALLDVVRDPASKIPSLITECV---H--N-VDFK-VLY--
069 UniRef90_J4GNM1_5_326            LQNLAGGPNIVALLDVVRDPSSKIPSLVTEYV---H--N-VDFK-VLY--
070 UniRef90_A0A1B6QHX1_78_406       LQNLYGGPNIVKLLDVVRDDESKTPSLIFEHV---N--N-TDFK-VLY--
071 UniRef90_E3Q428_2_328            LQNLAGGPNVVALLDVVRDSQSKTPSLIFEHV---N--N-TDFR-SLY--
```

072 UniRef90_A0A161HHJ2_5_327 LQNLAGGENIVMLLDVVRDPLSKTPSLIFEYV---N--N-TDFR-ILY--
073 UniRef90_A0A0C2TUQ3_4_328 LQNLAGGPNIVALLDVVRDPASKIPSLITEYV---H--N-VDFK-VLY--
074 UniRef90_UPI000644E1AF_26_344 LQNLCGGPNIITLLDVVRDPQSKTPSLIFEYI---N--N-TDFK-HLA--
075 UniRef90_F2U7H7_13_336 LQNLAGGVNVINLLDVVRDPKSKTPALIFEHV---N--N-TDFK-VLF--
076 UniRef90_A0A1E3Q523_2_312 LQNLAGGPNVIHLYDIVRDAQSRTPSLIFEYV---N--N-IDFR-ILY--
077 UniRef90_A0A446UN67_26_313 LQNLCGGPNIIKLLDIVRDQHSKTPSLIFEYV---N--N-TDFK-VLY--
078 UniRef90_A0A1E4RU21_6_330 LKNLVGGPNVIGLLDIVREPQLKTPSLIFEHI---D--N-IDFR-TLY--
079 UniRef90_A5DCV5_5_327 LKNL-DGPNIIGLFDIVREPQSKTPGLIFEHV---N--N-IDFR-TLY--
080 UniRef90_L8GRW8_10_332 LQNLNGGTNVVRLVDVVRNPHTKTPCLIFEFI---N--N-TPYK-HLY--
081 UniRef90_A3GID5_8_329 LKNLFSGPNIVALLDVVREPQSKTPGLIFEHI---N--N-VDFR-SLY--
082 UniRef90_A0A2V1AT92_3_322 LKNLVGGPNIINFLDMVREPQSKTPALVFEYV---D--N-TDFR-TLY--
083 UniRef90_A0A642UHU8_5_327 LKNLVNGPNIITLYDVVREPQSKTPAFIFELV---E--N-IDFR-TLY--
084 UniRef90_UPI001036488A_60_387 LGLLFGNWELLSSLPFLFFLDSILPSCISFNVWSCN--I-VKIY-VLL--
085 UniRef90_A0A448YKC4_4_323 LEDLAGGPQIVDLLDVVRDPISKIPALIFEYV---D--N-TEFR-TLY--
086 UniRef90_UPI001900E851_4_329 LQDLAGGPQIVDLLDVVRDPISKTPALIFEYV---N--N-TEFR-TLY--
087 UniRef90_A0A4P9ZDD8_3_324 LQNLVGGPNIVGLLDVVRDPQLKSPALIFEHI---N--N-VDFR-QLY--
088 UniRef90_UPI00064AE4FC_6_334 LEMLRGGPNIINLLAVVKIRDSEALALVFEFI---N--N-IQFK-ELH--
089 UniRef90_A0A7S3CI51_5_325 LQTLKGGINIINLIDVVRDPMTKTPALIMEYV---DTGD-VDFR-TLY--
090 UniRef90_UPI00098E15C8_59_372 LQNLCGGPNIVSLLDIVRDQQSKTFSLIFEFV---N--N-TEFK-VLY--
091 UniRef90_A5E6J5_9_319 LKNLVNGPNIITLLDIVREPQLKTPGLIFENV---N--N-IDFR-TLY--
092 UniRef90_A0A0S4KFI2_14_334 LQNLQNGPNVIQLLDAVRDPYSKTPSFVFEYV---K--A-ADFR-VLF--
093 UniRef90_A0A4Y7PMM1_7_330 LHNLAGGPNIITLLDVVRDPTSKISSVVTEYI---D--N-ADFR-VLY--
094 UniRef90_A0A674DLQ6_2_238 LENLRGGPNIISLLDIVKDPVSRTPALVFEHV---N--N-TDFK-VRR--
095 UniRef90_A0A367YK45_12_333 LKNLVNGPNIVSLLDVVREPQLKTPALVFEWV---Q--N-VDFR-SLY--
096 UniRef90_Q6BUZ4_8_327 LKNLTGGPNVIGLLDIVRDESSKIPALIFEKV---N--N-VDFR-VLY--
097 UniRef90_A0A7S3F0G7_17_281 LQNLRGGTNIIRLLDIVRDPQSKTPSLIFEYV---N--N-TDFK-VLY--
098 UniRef90_A0A0C3HJB5_2_320 LQNLAHGPNIIALRDIVMDDESRTPSLIFEYV---N--N-VDFR-SLY--
099 UniRef90_A0A1R2D4Q4_3_325 LINLTGGTNIIKLLNTVKDPISKTPALIFEYV---N--N-THFR-TLY--
100 UniRef90_UPI0004F4A9E2_17_332 LQNLQGGPNIVELFDVVRDPCSKTPSFIFEYV---Q--T-SNFR-TIF--
101 UniRef90_A0A4P6XGL7_6_324 LKNITGGPNVVALLDIVRDANSKIPALIFEQV---K--N-VDFR-VLY--
102 UniRef90_A0A6P6RSI1_108_381 LQNLYGGPNIIKLLDVVKDPLSRTPALIFEYV---N--N-IDFK-TLY--
103 UniRef90_A0A4U0VRL3_43_361 LSNLRGGTNIIELLDVVRDPQSKTPAIITEHV---D--N-LESR-LLY--
104 UniRef90_K0KNM3_9_329 LQNLTGGPNVIALLDLVKDTHSKIPALIFEEV---K--N-VDFR-TLY--
105 UniRef90_A0A2X0MBX0_6_345 LMNLRGGPNCIELLDVVRDARSNTPALITEFV---N--N-MDSK-LLY--
106 UniRef90_A0A7S1VNL6_68_384 LQNISGGTNIVTMIDAVIDRATGTPALIFEHI---A--N-DDFK-SLY--
107 UniRef90_A0A2H9TP39_34_357 LQNLAGGPNIIQLKDLVIEPESRTPAFIFELV---K--N-VEYR-TLY--
108 UniRef90_A0A315WAV6_110_366 LENLRGGTNIIRLVDTVKDPVSRTPALVFEFI---N--N-TDFK-ELY--
109 UniRef90_UPI001386DBE1_151_468 LENLRGGANIMKLIDIVKDSVSATPALVFEYI---R--N-TDFK-QRY--
110 UniRef90_A0A5D2A803_51_293 LQNLCGGPNIIKLLDIVRDQHSKTPSLVFEYV---N--N-TDFK-VLY--
111 UniRef90_A0CQX8_4_329 LQNLNGQNNVLKLLDVVYDPASKTTSLILEFI---N--N-TDYR-VLY--
112 UniRef90_T1EXU0_70_294 ---------------------SRTPALVFEYV---N--N-VDFK-QLY--
113 UniRef90_A0A7H9B7U0_15_336 LTNLTGGPNVIGLLDIVQDPGSKIPALIFEEV---K--N-VEFR-ALY--
114 UniRef90_A0A482VYB7_21_342 LEHLKNGPNIINLYATVTIPNTGLHALIFEST-S-N--N-EDFK-EVY--
115 UniRef90_A0A1D2VEG0_6_324 LQNLTGGKNIVGLLDIVHDPNSKIPALIFEHV---N--N-IDFR-VLY--
116 UniRef90_A0A0D0B3X6_2_320 LRNLTGGPNVVALLDVVQDPSSRYHSLIMEYV---D--N-VDWK-SLY--
117 UniRef90_A0A1G4MEF4_15_334 LTNLTGGPNVIGLLDIVQDPGSKIPALIFEEV---K--N-VDFR-TLY--
118 UniRef90_A0A2V0NTF2_16_339 LRHVRGGPNIVTLVDLVRDPDTKTPCFVFELV---E--A-LPLR-ELQ--
119 UniRef90_A0A4Y9YFI0_2_320 LRNLSGGPNIVGLVDVVRDYASRSHSLVMEYV---R--N-ADWR-DIY--
120 UniRef90_W6KIF7_13_329 LQILKGCPNIVELYDTVRDPSSKVPSFVFEYI---E--A-SDFR-TLF--
121 UniRef90_A0A0C3NKK6_2_320 LRNLAGGPNVISLLDVVRDPPSKSHSLIMEYV---D--N-TDWK-LLY--
122 UniRef90_A0A0D7AGR9_2_320 LRNLTGGPNVVALLDVVRDPSGRYHSLVMEYV---D--N-VEWK-QLY--
123 UniRef90_G0VHQ2_23_343 LWNLTGGPNIVELLDVVHDEKTRVPAFIFEDV---K--N-VDFR-ELY--
124 UniRef90_UPI000EA961A9_2_209 LENLRGGPNIINLLDIVKDPVVRRGGLFLTPI---P--S-SALQ-QLY--
125 UniRef90_A0A165QAJ6_2_320 LRNLSGGPNIVGLVDVVRDHVSRSHSLVMEYV---N--S-SDWR-DIY--
126 UniRef90_A0A6J1ACU4_2_223 LQNLCGGPNIVKLLDVVRDHHSKTPSLIFEYV---N--S-TDFK-VLY--
127 UniRef90_A0A146IBX2_2_322 LRNLAGAPNCISLIDVVRDPSRIYHSLITEFV---A--N-TEWS-QFY--
128 UniRef90_A0A1E5RA31_17_339 LTHLVGGTNVITLYDIVQDQECKVPALIFENV---G--N-VDYK-PLY--
129 UniRef90_J9HLC0_29_343 LQTLYGGPNIVKLYDVVRDPDNKTPSLVYEYI---P--N-IESK-ILF--
130 UniRef90_A0A183CIH8_805_1008 LENLRGGINVIGLLDVVKDPVSRTPALVFEYV---N--N-TDFK-QLY--
131 UniRef90_A0A1M2V5N7_3_298 LRNLAGGPNVIRLMDTVHDSPSRSNSLVMEYV---A--N-TDWK-ELY--
132 UniRef90_A0A1B7TD73_22_342 LTHLTGGDNIVTLYDIVHDPRSKIPAFIFENI---E--H-IDHK-KIF--
133 UniRef90_A0A4P9VZ92_1_208 ---DFK-VLY--
134 UniRef90_W0T7F4_129_366 -----GHQNIVTLLDVVRDPVSKTPALVFEYI---N--N-TDFR-TLY--
135 UniRef90_UPI000711954B_2_325 MQILKNGPYILPILDMVRDEESGSPNIVTKWI---S--T-LPYR-EFY--

```
136 UniRef90_A0A1C7MA44_2_304        LRNLAAAP----------------MSLVMEYV---Q--N-TDWK-ELH--
137 UniRef90_C5DWN1_128_366          ----NGHENIVQLLDVVRDPISRTPALVFEHV---N--N-IDFR-ILY--
138 UniRef90_A0A7H9B7A0_128_371      ----NGHENIVQLLDVIRDPISKTPALVFEHV---N--N-VDFR-VLY--
139 UniRef90_W5KQ02_73_295           ---------------------SRTPALVFECI---N--N-TDFK-ELY--
140 UniRef90_A0A6P6YBM1_25_347       LNHLQGCPNIIEFITAIKDPDSKVMSLVTEKV---N--T-WDFR-LLF--
141 UniRef90_A0A196S5L7_3_325        MQQLTGGPSILPLLSTVMNEETKAPSLITKWV---E--T-KDYR-EFY--
142 UniRef90_A0A7S3FVA9_17_301       LENVRGGPFIIEMLNQVYDPSNKTPSIIFELV---E--N-KDFR-QLW--
143 UniRef90_A2FBX9_18_336           LLNLRDGPNIVKLYDIVQNPKTLQYSMVFEYV---K--E-SDYT-DLF--
144 UniRef90_UPI0010FB2831_6_323     LKNLK-HDNIIRMIDVVKDVDTHTYSLIFEYI---D--F-LEVK-NVF--
145 UniRef90_Q8SRU0_6_317            LRNLS-HKNIIKLMDVVVDPESQIYSLIFEYI---E--H-EDYA-KIF--
146 UniRef90_E9ACB1_46_365           LRNLCGGPNVVRLLDVLRDTESQTVVLVTEYV---H--NPTTLR-NLLYS
147 UniRef90_W6KPE3_52_367           LQNLCGGPNVVRLLDVLQDARTNTIVLVTEYV---E--NPVTLR-WLMNK
148 UniRef90_A0A1J4JEQ9_9_332        LSNLEGGTNIVSLVDLVRDPMTGIYSFVFEWV---E--F-YDWR-SLY--
149 UniRef90_G9N8S2_27_316           LRSLGGGINIPKLYDIVQDSQMEPPSLVFEYI---E--N-IDFR-TLY--
150 UniRef90_A0A4Z1TD09_8_331        LSNLIGGPNCINLLDVVPVHGSDYPALIMEHG------G-LSLA-HSL--


001 Input_pdb_SEQRES_A               QTLTDYDIRFYMYEIL-KALDYCHSMGIMHRDVKPHNVMIDHEHR-----
002 UniRef90_A0A060X538_2_334        QTLTDYDIRFYMFEIL-KALDYSHSMGIMHRDVKPHNVMIDHEHR-----
003 UniRef90_UPI000719C52A_2_332     QTLTDYDIRFYLYELL-KALDFCHSMGIMHRDVKPHNVMIDHENK-----
004 UniRef90_A0A7R8XAU3_2_332        QTLTDFDIRFYVYELL-KALDYCHSMGIMHRDVKPHNVMIDHENR-----
005 UniRef90_V3ZFM5_3_333            QTFTDYDIRFYLYELL-KALDYCHSFGIMHRDVKPHNVMIDHENR-----
006 UniRef90_UPI00193A3309_3_330     QTLTDYDIRYYMFEIL-KALDFCHSMGIMHRDVKPHNVMIDHESR-----
007 UniRef90_A0A1Q3F4A5_3_329        QTLTDYDIRYYLYELL-KALDYCHSMGIMHRDVKPHNVMIDHENR-----
008 UniRef90_A0A4W3H6I6_2_321        QTLTDYDIRFYMYEIL-KALDYCHSMGIMHRDVKPHNVMIDHEHR-----
009 UniRef90_UPI000C6E8BEA_2_341     QTLTDYDIRFYLYELL-KALDYSHSMGIMHRDVKPHNVMIDHENR-----
010 UniRef90_A0A0L0BLN9_116_444      QTLTDYEIRYYLFELL-KALDYCHSMGIMHRDVKPHNVMIDHENL-----
011 UniRef90_A0A183IEN2_2_329        QTLTDYDIRFYIYELL-KALDFCHSMGIMHRDVKPHNVMIDHEAR-----
012 UniRef90_A0A5N4B3X1_3_332        QRLTDFDIRYYLYELL-KALDYCHSMGIMHRDVKPHNVMIDHENR-----
013 UniRef90_A0A556VUA8_53_369       QTLSDYDIRFYMYEIL-KALDYCHSMGIMHRDVKPHNVMIDHEHR-----
014 UniRef90_A0A1I8HE15_2_329        QTLVDYDIRYYLFELL-KALDYCHSMGIMHRDVKPHNVMIDHEHR-----
015 UniRef90_F6RN85_7_328            QILTDYDIRYYMYELL-KALDYCHSQGVMHRDVKPHNVMIDHQQK-----
016 UniRef90_UPI0011E4E1F1_2_326     QTLSDYDIRYYLYELL-KAIDYSHSMGIMHRDVKPHNVMIDHENR-----
017 UniRef90_A0A0P7V994_7_333        QKLTDYDIRYYMFELL-KALDYCHSKGIMHRDVKPHNVMIDHQLR-----
018 UniRef90_A0A3Q4N194_2_309        QTLTDFDIRFYMYEIL-KVSPPAHSMM-----CQTHNVVI----------
019 UniRef90_A0A0N5ANV2_2_328        QTLTDFDIRYYLYELL-KALNYCHSMGIMHRDVKPHNVMIDHDRR-----
020 UniRef90_A0A0M3HMV3_2_324        QTLTDYDIRYYLYELL-KALDFCHSQGIMHRDVKPHNVMIDHEQR-----
021 UniRef90_A0A2R7W6M3_5_330        QTLSDYDIRFYIYELL-KALDYSHSMGIMHRDIKPHNVMIDHENR-----
022 UniRef90_A0A368G0G8_241_571      GTLSDFDIRFYLYELL-KALDYCHSQGIMHRDVKPHNVMIDHEKKQFTLL
023 UniRef90_A0A5F4C0P0_224_555      QILTDFDIRFYMYELL-KALDYCHSKGIMHRDVKPHNVMIDHQQK-----
024 UniRef90_A0A671N1V7_3_326        QKLTDYDIRFYMFELL-KALDYCHSMGIMHRDVKPHNVMIDHQLR-----
025 UniRef90_A0A667Y761_7_331        FV---WLRSYILWSLLSQALDYCHSMGIMHRDVKPHNVMIDHQLR-----
026 UniRef90_I0Z462_2_325            PTLVDYDIRYYIHELL-KALDYCHSQGIMHRDVKPHNVMIDHEQR-----
027 UniRef90_A0A4W5MUS6_7_326        --VESFPLGMGLWFL---ALDYCHSMGIMHRDVKPHNVMIDHQMR-----
028 UniRef90_UPI000C1CFB1C_2_322     PTLTDYDIRYYIYELL-KALDYCHSQGIMHRDVKPHNVMIDHELR-----
029 UniRef90_A0A200QFN8_2_323        PTLTDYDIRYYIYELL-KALDYCHSQGIMHRDVKPHNVMIDHELR-----
030 UniRef90_A0A6I9QC06_84_406       PTLTDYDVRYYIYELL-KALDYCHSQGIMHRDVKPHNVMIDHEQR-----
031 UniRef90_A9NVF1_102_427          PTLSDFDIRYYIYELL-KALDYCHSQGIMHRDVKPHNVMIDHEHR-----
032 UniRef90_A0A6S7M9R1_74_394       PTLTDYDIRYYIYELL-KALDYCHSQGIMHRDVKPHNVMIDHELR-----
033 UniRef90_A0A5J4ZXR3_110_433      PTLSDFDIRYYIYELL-KALDFCHSQGIMHRDVKPHNVMIDHEQH-----
034 UniRef90_A0A0D2WV07_2_326        PTLSDYDIRFYLYELV-KALDFCHSNGIMHRDVKPHNVMIDHTKR-----
035 UniRef90_A0A2P6Q0W9_107_428      PTLTDYDIRYYIYELL-KALDYCHSQGIMHRDVKPHNVMIDHELR-----
036 UniRef90_A0A2J6LRB0_100_422      PTLSDFDIRYYIFELL-KALEYCHSQGIMHRDVKPHNVMIDHEQR-----
037 UniRef90_A0A4S8ISY9_72_395       PTLSDYDIRYYIYELL-KALDYCHSQGIMHRDVKPHNVMIDHQQR-----
038 UniRef90_A0A1U8LZW6_92_416       PTLSDYDIRYYIYELL-KALDYCHSQGIMHRDVKPHNVMIDHELR-----
039 UniRef90_K3XBI1_15_338           PTLTDYDIRYYIFELL-KALDFCHANGIMHRDVKPHNVMIDHEKR-----
040 UniRef90_E1ZRP6_14_337           PTLSDYDIRYYVMEIL-KALDFCHSKGIMHRDVKPHNIMIDHSQK-----
041 UniRef90_A0A2I0AFK3_70_391       PTLTDYDTRYYIYELL-KALEYCHSQGIMHRDVKPHNVMIDHEQK-----
042 UniRef90_UPI000F7CDB20_80_401    PTLSDYDIRYYIYELL-KALDYCHSQGIMHRDVKPHNVMIDHEQR-----
043 UniRef90_G7K3U9_82_403           PTLSDYDIRYYIYELL-KALDYCHSQGIMHRDVKPHNIMIDHAQR-----
044 UniRef90_A0A663MTN9_26_324       ------DVSNVCACVC-QALDYCHSMGIMHRDVKPHNVMIDHQQK-----
045 UniRef90_A4S5X1_14_334           PTLTDYDIRYYINELL-KALDFCHSQGIMHRDVKPHNVMIDHSKR-----
```

```
046 UniRef90_A0A183LBY6_9_327        HTLTDYDIRYYMFELL-RALDACHSMGIMHRDVKPHNVMINHSQR-----
047 UniRef90_A0A1J9RPL1_99_422       PKFQDYDVRFYIFELL-KALDFCHSKGIMHRDVKPHNVMIDHEKR-----
048 UniRef90_D5GJ91_2_320            PKFQDYDVRYYIYELL-KALDFCHSKGIMHRDVKPHNVMIDHEKR-----
049 UniRef90_A0A4W4DPG2_3_315        QRLTDFDIRFYLYELL-RALDYSHSMGIMHRDVKPHNVMIDHQMR-----
050 UniRef90_A0A7S2RD37_2_324        PTLTDFDIRYYINELL-KALDFSHSNGIMHRDVKPHNVMIDHKNR-----
051 UniRef90_A0A367JQL9_12_336       PRFTVYDIQYYMYELL-KALDYCHSKGIMHRDVKPHNVMIDHEKK-----
052 UniRef90_UPI001924B863_29_350    PTLTDYDIRYYIYELL-KALDYCHSQGIMHRDVKPHNVMIDHELR-----
053 UniRef90_A0A367KWU7_10_331       PRFTSYDIRYYMYELL-KALDFCHSKGIMHRDVKPHNVMIDHEKK-----
054 UniRef90_A0A0D2AMV0_2_320        PRFTDYDVRYYIYELL-KALDFCHSKGIMHRDVKPHNVMIDHEKR-----
055 UniRef90_A0A1Y1WVY1_2_324        PRFVDYDIRYYLFELL-KALDFCHSKGIMHRDVKPHNVMIDHEKR-----
056 UniRef90_Q8TG13_2_324            PRFNDFDVRYYIFELL-KALDFCHSKGIMHRDVKPHNVMIDHENR-----
057 UniRef90_A0A0F8B0Y9_1_322        PRFTDLDVRFYIFELL-KALDFCHSKGIMHRDVKPHNIMIDHENR-----
058 UniRef90_A0A7S1NI55_6_324        PTLFDHDIRYYMFELL-KALAFCHSMGIMHRDVKPHNVMIDHEKK-----
059 UniRef90_A0A383VX45_13_335       PLLGDYDIRYYVFLLL-KALDYSHSQGIMHRDVKPHNVMIDHERR-----
060 UniRef90_I1C1X5_16_338           PKLTPYDIRYYMYELL-KALDYCHSKGIMHRDVKPHNVMIDHEKK-----
061 UniRef90_A0A364LA26_2_320        PRFVDYDVRYYILELL-KALDFCHSKGIMHRDVKPHNVMIDHENR-----
062 UniRef90_A0A1X7RU70_2_320        PKFQDLDVRYYIFELL-KALDFCHSKGIMHRDVKPHNVMIDHENR-----
063 UniRef90_A0A067BWS1_2_324        PTLTDFDIRYYMFELL-KALDFCHANGIMHRDVKPHNVMIDHEKR-----
064 UniRef90_A0A0P9FCQ6_6_328        PRLTDYDVRYYIYELL-KALDFCHSRGIMHRDVKPHNVMIDHEQR-----
065 UniRef90_A0A0K3CS88_10_335       PRLTDYDVRYYIYELL-KALDFCHSRGIMHRDVKPHNVMIDHEKR-----
066 UniRef90_A0A4S4KUV1_8_328        PRFTDFDVRFYMLELL-KALDYCHSKGIMHRDVKPHNVMIDHERR-----
067 UniRef90_UPI000511B7B1_2_322     PTLTDYDVRYYIYELL-KALDYCHSXGIMHRDVKPHNVMIDHDLR-----
068 UniRef90_A0A5C3QYQ7_5_326        PRFTDYDVRFYMHELL-KALDFCHSKGIMHRDVKPHNVMIDHEHK-----
069 UniRef90_J4GNM1_5_326            PRFADYDVRYYMLELL-KALDFCHSKGIMHRDVKPHNVMIDHERR-----
070 UniRef90_A0A1B6QHX1_78_406       PVLSDYDIRYYIFELL-KALDYCHSRGIMHRDVKPHNVMIDHEKR-----
071 UniRef90_E3Q428_2_328            PKFNDLDVRYYIHELL-KALDFCHSKGIMHRDVKPHNVMIDHENR-----
072 UniRef90_A0A161HHJ2_5_327        PKLTDFDIRYYIFELL-KALDYSHSKGIMHRDVKPHNVMIDHEKR-----
073 UniRef90_A0A0C2TUQ3_4_328        PRFTDMDVRYYMFELL-KALDYCHSKGIMHRDVKPHNVMIDHERR-----
074 UniRef90_UPI000644E1AF_26_344    PTLTDFDVRFYILELL-RALDFCHSNGVMHRDVKPSNVMVDHQKR-----
075 UniRef90_F2U7H7_13_336           PRFTLQDIRYYLYQLL-KALDFCHSKGIMHRDVKPHNFMIDHEKK-----
076 UniRef90_A0A1E3Q523_2_312        PRFTDYDIRYYIYELL-VALDFCHSKGIMHRDVKPHNVMIDHEKR-----
077 UniRef90_A0A446UN67_26_313       PTLTDYDIRYYLYELL-KALDHCHSQGIMHRDVKPHNVMIDHDLR-----
078 UniRef90_A0A1E4RU21_6_330        PTFTDYDIRYYMYELL-KALDFSHSMGIMHRDVKPHNVMIDHEHK-----
079 UniRef90_A5DCV5_5_327            QSFTDYDIRFYMYELL-RALDYSHSMGIMHRDVKPHNVMIDHEKK-----
080 UniRef90_L8GRW8_10_332           ANFSDFDCRFYMYELL-RALDFAHSNGVMHRDVKPQNVMIDHQKR-----
081 UniRef90_A3GID5_8_329            PTFGDYDIRYYMYELL-KALDYSHSMGIMHRDVKPHNVMIDHDKR-----
082 UniRef90_A0A2V1AT92_3_322        PTFTDYDNRYYMFELL-RALDYCHSMGIIHRDVKPQNVMIDHGAK-----
083 UniRef90_A0A642UHU8_5_327        PTFTDYDIRFYMFQLL-RALDYCHSQGIMHRDVKPHNVMIDHKEK-----
084 UniRef90_UPI001036488A_60_387    TTLTDYDIRYYIYELL-KALDYCHSQGIMHRDVKPHNVMIDHELR-----
085 UniRef90_A0A448YKC4_4_323        PQLSDMEVRYYMYELL-EALNYAHSMGIMHRDVKPHNIMIDHAHR-----
086 UniRef90_UPI001900E851_4_329     PKLSDMEVRYYMYQLL-EALDFAHSMGIMHRDVKPHNIMIDHAHK-----
087 UniRef90_A0A4P9ZDD8_3_324        PTFTDKDIRYYMYELL-RALDFSHSMGIMHRDVKPHNVMIDQTTK-----
088 UniRef90_UPI00064AE4FC_6_334     QILTDYEIRFYTYQIL-KALDYCHSMGIMHRDLKPENIMIDHKQR-----
089 UniRef90_A0A7S3CI51_5_325        KSFADYDIRFYIYEVL-KALDFCHSKGIMHRDVKPHNIMIDHNNK-----
090 UniRef90_UPI00098E15C8_59_372    PTLTDYDVRYYIYELL-KALDYCHSQGIMHRDVKPHNVMIDHERR-----
091 UniRef90_A5E6J5_9_319            PTFTDFDIRFYMYELL-KALSYSHSMGIMHRDVKPHNVMIDHDKK-----
092 UniRef90_A0A0S4KFI2_14_334       PTLSDLEVRYYIFEVL-KALEFAHSMGIMHRDVKPHNVMIDHEQK-----
093 UniRef90_A0A4Y7PMM1_7_330        PRFTDLDVRYYVFELL-KALDFCHSHGIMHRDVKPHNIMIDHKHR-----
094 UniRef90_A0A674DLQ6_2_238        ITVNFYPVKME----A-EALDYCHSMGIMHRDVKPHNVMIDHEHR-----
095 UniRef90_A0A367YK45_12_333       PTFSDHDIRFYMYQLL-LALDFAHSQGIMHRDVKPHNVMIDHNKK-----
096 UniRef90_Q6BUZ4_8_327            PKFTISDIQYYFTQLL-IALDYSHSMGIIHRDVKPQNIMIDPLTK-----
097 UniRef90_A0A7S3F0G7_17_281       PTLTDYDLRYYIFELL-KALEYCHSNGIMHRDVKPHNVMIDHQKR-----
098 UniRef90_A0A0C3HJB5_2_320        PRFVDHDIRFYIYELL-KALDFCHSKGIMHRNVRPHNVMIDHEKQ-----
099 UniRef90_A0A1R2D4Q4_3_325        PILTDFQVRYYIYELL-KALDYSHSMGIMHRDVKPQNVMIDHSQR-----
100 UniRef90_UPI0004F4A9E2_17_332    PTLSDLEVRYYTFEVL-KALEYAHSMGIMHRDVKPNNIVIDHQRR-----
101 UniRef90_A0A4P6XGL7_6_324        AKFTIPDIQYYFTQLL-IALDFSHSMGIMHRDVKPQNIMIDPQSK-----
102 UniRef90_A0A6P6RSI1_108_381      PTLKDYEIRYYIYQIL-KALDYCHSQGIMHRDVKPHNVMIDHEKR-----
103 UniRef90_A0A4U0VRL3_43_361       PKFTDNDVRYYMYELL-KALDFCHSKGIIHRDVKPLNILIDHSQR-----
104 UniRef90_K0KNM3_9_329            AKFKRGDIQFYFTQLL-TALDYSHSMGIIHRDVKPQNIMIDPFNR-----
105 UniRef90_A0A2X0MBX0_6_345        PTFSDYDVRYYIYELL-KALDYCHSHGIIHRDVKPLNILIDHQQK-----
106 UniRef90_A0A7S1VNL6_68_384       PTFTDADVRHFFFELL-RALEYCHARGIAHRDVKPHNIMYNAQTR-----
107 UniRef90_A0A2H9TP39_34_357       PTLQDHDVRYYIFQLL-RALDFAHSRGIMHRDVKPHNVMIDSETK-----
108 UniRef90_A0A315WAV6_110_366      QKLTDYDIRYYMYELL-KALDYCHSMGIMHRDVKPHNVMIDHQLR-----
109 UniRef90_UPI001386DBE1_151_468   RIPTDFEVRCYMHELR-KALDSCRSKGVRPRDGKPHDVMVGQQQR-----
```

```
110 UniRef90_A0A5D2A803_51_293     PTLTDYDIRYYIYELL-KALDYCHSQGIMHRDVKPHNIMIDHEQR-----
111 UniRef90_A0CQX8_4_329          PQLQDQDVRFYMYEIL-KSLDHCHSMGIIHRDIKPHNIMIDHEKK-----
112 UniRef90_T1EXU0_70_294         PTLTDYDIRFYILEVL-KALEYCHSHGIMHRDVKPHNVMIDHDLK-----
113 UniRef90_A0A7H9B7U0_15_336     PTFKSNDIQYYFTQLL-IALDYCHSMGIMHRDVKPQNVMIDPSEK-----
114 UniRef90_A0A482VYB7_21_342     LKLNDLDIRFYIYEIL-KALDYCHSKGIMHRDVKPHNIIIDRKNR-----
115 UniRef90_A0A1D2VEG0_6_324      PKFQIIDIKYYFTQLL-IGLDYCHSMGIIHRDVKPQNVMIDPIKR-----
116 UniRef90_A0A0D0B3X6_2_320      PVLGEVDIKFYTFQLL-KALDFVHSRGIMHRDVKPGNVMIDHRHR-----
117 UniRef90_A0A1G4MEF4_15_334     PSFTLSDIQYYFTQLL-IALDYCHSMGIMHRDVKPQNVMIDPTER-----
118 UniRef90_A0A2V0NTF2_16_339     AVVSDLDVRSYMHQLL-QALDFTHSRGIMHRDVKPANVLIDHSKR-----
119 UniRef90_A0A4Y9YFI0_2_320      RSLTELEIKQYLFQLL-RALDFAHSRGIMHRDVKPGNIMYDREQR-----
120 UniRef90_W6KIF7_13_329         PHFSDLDVRNLIYQVL-IALEYAHSRGIMHRDVKPNNVCIDYKQK-----
121 UniRef90_A0A0C3NKK6_2_320      PTFTELEIKHYLFQLL-TALDFVHSRGIMHRDVKPGNIMFDRANH-----
122 UniRef90_A0A0D7AGR9_2_320      MSLTEGDIKHYTFQLL-KALDFVHAHGIMHRDVKPGNVMVDARTR-----
123 UniRef90_G0VHQ2_23_343         PTFKLSDVQYYFKQLL-IALNYAHSMGIMHRDVKPQNVMIDPVQR-----
124 UniRef90_UPI000EA961A9_2_209   QTLSDFDIRFYMYEIL-KALDYCHSMGVMHRDVKPHNVMIDHEHR-----
125 UniRef90_A0A165QAJ6_2_320      RSLTELEIKQYVFQIL-RALEFAHSRGIMHRDVKPGNIVFDREQR-----
126 UniRef90_A0A6J1ACU4_2_223      PTLTDYDIRYYIYELL-KALDYCHSQGIMHRDVKPHNVMIDHELR-----
127 UniRef90_A0A146IBX2_2_322      NKLTDVDIRHYTFQLL-LALDFVHAHGIMHRDIKPANVMIDHQRR-----
128 UniRef90_A0A1E5RA31_17_339     GSFSLMDIQHYFRQLL-YALDYCHSMGIMHRDVKPQNVMIDPIKR-----
129 UniRef90_J9HLC0_29_343         QRLNDLEIRFYIYRLL-ESLEYSHLHGIMHRDLKPLNIVINHEKK-----
130 UniRef90_A0A183CIH8_805_1008   QTLSDYEIRYYLYELL-KALEYCHSMGIMHRDVKPHNVMIDHEQR-----
131 UniRef90_A0A1M2V5N7_3_298      CTLTEMEIKHYLFQLL-RALDFVHSRGIIHRDVKPANIMFDRARR-----
132 UniRef90_A0A1B7TD73_22_342     PTFRIPEMQNYFKQLL-NALKYAHSIGIMHRDVKPQNIMYDPIRK-----
133 UniRef90_A0A4P9VZ92_1_208      PKLTDYDVRYYIMELL-KALDFCHSRGIMHRDVKPHNVMIDHEKR-----
134 UniRef90_W0T7F4_129_366        ATFTDLDIRYYMFELL-KALDYCHSMGIMHRDVKPHNVMIDHKQR-----
135 UniRef90_UPI000711954B_2_325   SSLTDHEFRYYLYKML-IGLDYTHSCGIIHRDIKPQNVLVDYKTR-----
136 UniRef90_A0A1C7MA44_2_304      ATISETEIKHYLFQLL-RALDFVHSRGIIHRDVKPGNIMFDRQRR-----
137 UniRef90_C5DWN1_128_366        PKLTDMDMRFYMFELL-KALDYCHSMGIMHRDVKPHNVMIDHKHK-----
138 UniRef90_A0A7H9B7A0_128_371    PKFSDFDMRYYTFELL-KALDYCHSMGIMHRDVKPHNVMIDHKQR-----
139 UniRef90_W5KQ02_73_295         QKLTDFDIRFYMYELL-KALDYCHSMGIMHRDVKPHNVMIDHQC------
140 UniRef90_A0A6P6YBM1_25_347     STFTATDLKIYLFNLL-IALDMANSRGIMHRDIKPHNIMYDKANK-----
141 UniRef90_A0A196S5L7_3_325      MNLTDYQLRYYFYKIL-VGLDYAHSKGIMHRDIKPQNILIDYVTK-----
142 UniRef90_A0A7S3FVA9_17_301     PLLTLEDIQTYILQIL-IGLDYAHSRGIIHRDIKPGNVLINHKAK-----
143 UniRef90_A2FBX9_18_336         PDIKEDEAKLYLYQLL-QALDYCHSCGIMHRDVKPLNILFDRSTR-----
144 UniRef90_UPI0010FB2831_6_323   MAFDMSEIKFYCRQIL-QALEYCHSHGIVHRDVKPHNMIIDKDTK-----
145 UniRef90_Q8SRU0_6_317          EKLCYKDIVEYSRQIL-SALSYCHSMGIIHRDIKPQNMVINQARR-----
146 UniRef90_E9ACB1_46_365         NKLSNFDMRYYLYEIL-RSLDFAHRRGIFHRDIKPYNVMIDHERK-----
147 UniRef90_W6KPE3_52_367         SLLENFDLRYYMYEIL-RTLDFAHRRGIFHRDIKPHNIMIDHKHR-----
148 UniRef90_A0A1J4JEQ9_9_332      MSFTIDDIRLYMYKLL-LSLEYSHSHGIMHRDIKPQNIAIDKEKR-----
149 UniRef90_G9N8S2_27_316         PTFSDGDVRYYMKELL-KAVEFCHSKSVMHRDIRPHNIMIDHNQR-----
150 UniRef90_A0A4Z1TD09_8_331      PQLDSQSIRIIIFQTL-RALDWLHIHGIIHRDTKPGNITYDLTTH-----
```

```
001 Input_pdb_SEQRES_A             --------K----------------LRLIDWGLAEFYHPGQEYNVRVASR
002 UniRef90_A0A060X538_2_334      --------K----------------LRLIDWGLAEFYHPGQEYNVRVASR
003 UniRef90_UPI000719C52A_2_332   --------K----------------LRLIDWGLAEFYHPGQEYNVRVASR
004 UniRef90_A0A7R8XAU3_2_332      --------K----------------LRLIDWGLAEFYHPNQEYNVRVASR
005 UniRef90_V3ZFM5_3_333          --------K----------------LRLIDWGLAEFYHPGMEYNVRVASR
006 UniRef90_UPI00193A3309_3_330   --------K----------------LRLIDWGLAEFYHPSQEYNVRVASR
007 UniRef90_A0A1Q3F4A5_3_329      --------K----------------LRLIDWGLAEFYHPGQEYNVRVASR
008 UniRef90_A0A4W3H6I6_2_321      --------K----------------LRLIDWGLAEFYHPGQEYNVRVASR
009 UniRef90_UPI000C6E8BEA_2_341   --------K----------------LRLIDWGLAEFYHPGQEYNVRVASR
010 UniRef90_A0A0L0BLN9_116_444    --------K----------------LRLIDWGLAEFYHPGQEYNVRVASR
011 UniRef90_A0A183IEN2_2_329      --------K----------------LRLIDWGLAEFYHPGQEYNVRVASR
012 UniRef90_A0A5N4B3X1_3_332      --------K----------------LRLIDWGLAEFYHPGQEYNVRVASR
013 UniRef90_A0A556VUA8_53_369     --------K----------------LRLIDWGLAEFYHPSQEYNVRVASR
014 UniRef90_A0A1I8HE15_2_329      --------K----------------LRLIDWGLAEFYHAGQEYNVRVASR
015 UniRef90_F6RN85_7_328          --------K----------------LRLIDWGLAEFYHPAQEYNVRVASR
016 UniRef90_UPI0011E4E1F1_2_326   --------K----------------LRLIDWGLAEFYHPGQEYNVRVASR
017 UniRef90_A0A0P7V994_7_333      --------K----------------LRLIDWGLAEFYHPAQEYNVRVASR
018 UniRef90_A0A3Q4N194_2_309      --------H----------------LRLIDWGLAEFYHPGQEYNVRVASR
019 UniRef90_A0A0N5ANV2_2_328      --------T----------------LRLIDWGLAEFYHPEQEYNVRVASR
```

```
020 UniRef90_A0A0M3HMV3_2_324        --------T----------------LRLIDWGLAEFYHTRQEYNVRVASR
021 UniRef90_A0A2R7W6M3_5_330        --------K----------------LRLIDWGLAEFYHPNKEYNVRVASR
022 UniRef90_A0A368G0G8_241_571      IYSNIMLLQ----------------LRLIDWGLAEFYHPKQDYNVRVASR
023 UniRef90_A0A5F4C0P0_224_555      --------K----------------LRLIDWGLAEFYHPAQEYNVRVASR
024 UniRef90_A0A671N1V7_3_326        --------K----------------LRLIDWGLAEFYHPAQEYNVRVASR
025 UniRef90_A0A667Y761_7_331        --------K----------------LRLIDWGLAEFYHPAQEYNVRVASR
026 UniRef90_I0Z462_2_325            --------K----------------LRLIDWGLAEFYHPGKEYNVRVASR
027 UniRef90_A0A4W5MUS6_7_326        --------K----------------LRLIDWGLAEFYHPAQEYNVRVASR
028 UniRef90_UPI000C1CFB1C_2_322     --------K----------------LRLIDWGLAEFYHPGKEYNVRVASR
029 UniRef90_A0A200QFN8_2_323        --------K----------------LRLIDWGLAEFYHPGKEYNVRVASR
030 UniRef90_A0A6I9QC06_84_406       --------K----------------LRLIDWGLAEFYHPGKEYNVRVASR
031 UniRef90_A9NVF1_102_427          --------K----------------LRLIDWGLAEFYHPGKEYNVRVASR
032 UniRef90_A0A6S7M9R1_74_394       --------K----------------LRLIDWGLAEFYHPGKEYNVRVASR
033 UniRef90_A0A5J4ZXR3_110_433      --------K----------------LRLIDWGLAEFYHPGKEYNVRVASR
034 UniRef90_A0A0D2WV07_2_326        --------Q----------------LRLIDWGLAEFYHPGREYNVRVASR
035 UniRef90_A0A2P6Q0W9_107_428      --------K----------------LRLIDWGLAEFYHPGKEYNVRVASR
036 UniRef90_A0A2J6LRB0_100_422      --------K----------------LRLIDWGLAEFYHPGTEYNVRVASR
037 UniRef90_A0A4S8ISY9_72_395       --------K----------------LRLIDWGLAEFYHPEKEYNVRVASR
038 UniRef90_A0A1U8LZW6_92_416       --------K----------------LRLIDWGLAEFYHPGKEYNVRVASR
039 UniRef90_K3XBI1_15_338           --------Q----------------LRLIDWGLAEFYHPGREYNVRVASR
040 UniRef90_E1ZRP6_14_337           --------K----------------LRLIDWGLAEFYHPGREYNVRVASR
041 UniRef90_A0A2I0AFK3_70_391       --------T----------------LRLIDWGLAEFYHPGKEYNVRVASR
042 UniRef90_UPI000F7CDB20_80_401    --------R----------------LRLIDWGLAEFYHPGKEYNVRVASR
043 UniRef90_G7K3U9_82_403           --------K----------------LCLIDWGLAEFYHPGQEYNVRVASR
044 UniRef90_A0A663MTN9_26_324       --------K----------------LRLIDWGLAEFYHPAQEYNVRVASR
045 UniRef90_A4S5X1_14_334           --------E----------------LRLIDWGLAEFYHPGKEYNVRVASR
046 UniRef90_A0A183LBY6_9_327        --------K----------------LRLIDWGLAEFYHPGQEYNVRVASR
047 UniRef90_A0A1J9RPL1_99_422       --------K----------------LRLIDWGLAEFYHAGTEYNVRVASR
048 UniRef90_D5GJ91_2_320            --------K----------------LRLIDWGLAEFYHAGTEYNVRVASR
049 UniRef90_A0A4W4DPG2_3_315        --------K----------------LRLIDWGLAEFYHPAQEYNVRVASR
050 UniRef90_A0A7S2RD37_2_324        --------K----------------LRLIDWGLAEFYHPGREYNVRVASR
051 UniRef90_A0A367JQL9_12_336       --------E----------------LRLIDWGLAEFYHAGTAYNVRVASR
052 UniRef90_UPI001924B863_29_350    --------K----------------LRLIDWGLAEFYHPGKEYSVRVGSR
053 UniRef90_A0A367KWU7_10_331       --------Q----------------LRLIDWGLAEFYHSGTAYNVRVASR
054 UniRef90_A0A0D2AMV0_2_320        --------K----------------LRLIDWGLAEFYHKGTEYNVRVASR
055 UniRef90_A0A1Y1WVY1_2_324        --------Q----------------LRLIDWGLAEFYHPGTEYNVRVASR
056 UniRef90_Q8TG13_2_324            --------K----------------LRLIDWGLAEFYHPGTEYNVRVASR
057 UniRef90_A0A0F8B0Y9_1_322        --------K----------------LRLIDWGLAEFYHPGTEYNVRVASR
058 UniRef90_A0A7S1NI55_6_324        --------K----------------LRLIDWGLAEFYHAGQEYNVRVASR
059 UniRef90_A0A383VX45_13_335       --------Q----------------LRLIDWGLAEFYHQGREYNVRVASR
060 UniRef90_I1C1X5_16_338           --------Q----------------LRLIDWGLAEFYHAGTAYNVRVASR
061 UniRef90_A0A364LA26_2_320        --------K----------------LRLIDWGLAEFYHKGTEYNVRVASR
062 UniRef90_A0A1X7RU70_2_320        --------K----------------LRLIDWGLAEFYHAGTEYNVRVASR
063 UniRef90_A0A067BWS1_2_324        --------Q----------------LRLIDFGLAEFYHPHREYNVRVASR
064 UniRef90_A0A0P9FCQ6_6_328        --------K----------------LRLIDWGLAEFYHPGTEYNVRVASR
065 UniRef90_A0A0K3CS88_10_335       --------Q----------------LRLIDWGLAEFYHPNTEYNVRVASR
066 UniRef90_A0A4S4KUV1_8_328        --------K----------------LRLIDWGLAEFYHPKTEYNVRVASR
067 UniRef90_UPI000511B7B1_2_322     --------K----------------LRLIDWGLTEFYHPGKEYNVRVASR
068 UniRef90_A0A5C3QYQ7_5_326        --------K----------------LRLIDWGLAEFYHPKTEYNVRVASR
069 UniRef90_J4GNM1_5_326            --------K----------------LRLIDWGLAEFYHPKTEYNVRVASR
070 UniRef90_A0A1B6QHX1_78_406       --------Q----------------LRLIDWGLAEFYHPKMEYNARVASR
071 UniRef90_E3Q428_2_328            --------KACL--------RAWRSLRLIDWGLAEFYHQGTEYNVRVASR
072 UniRef90_A0A161HHJ2_5_327        --------Q----------------LRLIDWGLAEFYHPGTEYNVRVASR
073 UniRef90_A0A0C2TUQ3_4_328        --------K----------------LRLIDWGLAEFYHSRTEYNVRVASR
074 UniRef90_UPI000644E1AF_26_344    --------K----------------LYLIDWGLAEFYHPNQDYNVRVASR
075 UniRef90_F2U7H7_13_336           --------E----------------LRLIDWGLAEFYHAGREYNVRVASR
076 UniRef90_A0A1E3Q523_2_312        --------S----------------LRLIDWGLAEFYHAGQEYNVRVASR
077 UniRef90_A0A446UN67_26_313       --------K----------------LRLIDWGLAEFYHPGKEYNVRVASR
078 UniRef90_A0A1E4RU21_6_330        --------K----------------IRLIDWGLAEYYHPGTEYNVRVASR
079 UniRef90_A5DCV5_5_327            --------L----------------LRLIDWGLAEYYHPGTEYNVRVASR
080 UniRef90_L8GRW8_10_332           --------Q----------------LRLIDWGLAEFYHPYMEYNVRVASR
081 UniRef90_A3GID5_8_329            --------S----------------LRLIDWGLAEYYHPGTEYNVRVASR
082 UniRef90_A0A2V1AT92_3_322        --------E----------------LRLIDWGLAEYYHPGTEYNVRVASR
```

```
083 UniRef90_A0A642UHU8_5_327          ---------K----------------LRLIDWGLAEYYHPGTEYNVRVASR
084 UniRef90_UPI001036488A_60_387      ---------K----------------LRLIDWGLAEFYHPGKEYNVRVASR
085 UniRef90_A0A448YKC4_4_323          ---------K----------------LRLIDWGLAEFYHPGTDYNVRVASR
086 UniRef90_UPI001900E851_4_329       ---------K----------------LRLIDWGLAEFYHPGTDYNVRVASR
087 UniRef90_A0A4P9ZDD8_3_324          ---------T----------------LRLIDWGLAEYYHPGTEYNVRVASR
088 UniRef90_UPI00064AE4FC_6_334       ---------K----------------LWLIDWGMSQFYNPGEEYSAEVVTR
089 UniRef90_A0A7S3CI51_5_325          ---------K----------------LRLIDWGLAEFYHPQQEYNVRVASR
090 UniRef90_UPI00098E15C8_59_372      ---------K----------------LRAIPKNFVLF--PKSHF------R
091 UniRef90_A5E6J5_9_319              ---------I----------------LRLIDWGLAEYYHPGTEYNVRVASR
092 UniRef90_A0A0S4KFI2_14_334         ---------K----------------LKLIDWGLAEFYHPATTYNARVASR
093 UniRef90_A0A4Y7PMM1_7_330          ---------K----------------LRLIDWGLADFYHPEKDLSVRVASP
094 UniRef90_A0A674DLQ6_2_238          ---------K----------------LRLIDWGLAEFYHPAQEYNVRVASR
095 UniRef90_A0A367YK45_12_333         ---------L----------------LRLIDWGLAEYYHAGADYNVRVASR
096 UniRef90_Q6BUZ4_8_327              ---------K----------------LRLIDWGLAEFYHSGMDYNVRVASR
097 UniRef90_A0A7S3F0G7_17_281         ---------Q----------------LRLIDWGLAEFYHPDREYNVRVASR
098 UniRef90_A0A0C3HJB5_2_320          ---------K----------------LQLIGWDLAEFYHPGTKYCVRVCPG
099 UniRef90_A0A1R2D4Q4_3_325          ---------L----------------LRLIDWGLAEFYHPLNEYNVRVASR
100 UniRef90_UPI0004F4A9E2_17_332      ---------K----------------LVLIDWGLAEFYHPATTYNARVASR
101 UniRef90_A0A4P6XGL7_6_324          ---------K----------------LRLIDWGLAEFYHAGMDYNVRVASR
102 UniRef90_A0A6P6RSI1_108_381        ---------E----------------LRLIDWGLAEFYHPGHEYNVRVASR
103 UniRef90_A0A4U0VRL3_43_361         ---------K----------------LRLIDWGLAEFYHPGVELNVRVASR
104 UniRef90_K0KNM3_9_329              ---------R----------------LRLIDWGLAEFYHPGVDYNVRVASR
105 UniRef90_A0A2X0MBX0_6_345          ---------K----------------LRLVDFGLAEFYIKGVELNVRVASR
106 UniRef90_A0A7S1VNL6_68_384         ---------E----------------LRLIDWGLAEFYHPDQDYNVRVASR
107 UniRef90_A0A2H9TP39_34_357         ---------T----------------LRLIDWGLAEFYHPGVAYNVRVASR
108 UniRef90_A0A315WAV6_110_366        ---------KVLLLFLLFLLFRPKMILRLIDWGLAEFYHPAQEYNVRVASR
109 UniRef90_UPI001386DBE1_151_468     ---------K----------------LRLIAWGLAEFHRPAPEYHVRAASR
110 UniRef90_A0A5D2A803_51_293         ---------K----------------LRLIDWGLAEFYHPGKEYNVRVASR
111 UniRef90_A0CQX8_4_329              ---------L----------------LKLIDFGLAEFYFPNKNYNCRVASR
112 UniRef90_T1EXU0_70_294             ---------K----------------LRLIDWGLAEFYHPGQEYNVRVASR
113 UniRef90_A0A7H9B7U0_15_336         ---------K----------------LRLIDWGLAEFYHPGVDYNVRVASR
114 UniRef90_A0A482VYB7_21_342         ---------K----------------IRLIDFGLAEFYRPGERYNIRVASR
115 UniRef90_A0A1D2VEG0_6_324          ---------K----------------LRLIDWGLAEFYHPGTNYNVRVASR
116 UniRef90_A0A0D0B3X6_2_320          ---------K----------------LRVIDWGLAEFYHPGTPYHIRVGSR
117 UniRef90_A0A1G4MEF4_15_334         ---------K----------------LRLIDWGLAEFYHPGVDYNVRVASR
118 UniRef90_A0A2V0NTF2_16_339         ---------Q----------------LKLIDWGLADFYFPGKEYPVRVATR
119 UniRef90_A0A4Y9YFI0_2_320          ---------K----------------LRLIDWGLAEFYHPGTEYPPRVGSR
120 UniRef90_W6KIF7_13_329             ---------K----------------LRLIDWGLAEFYHPETSYNARVASR
121 UniRef90_A0A0C3NKK6_2_320          ---------K----------------LRLIDWGLAEFYHPNTEYHIRVGSR
122 UniRef90_A0A0D7AGR9_2_320          ---------K----------------LRLIDWGLAEFYHPSTDYHIRVGSR
123 UniRef90_G0VHQ2_23_343             ---------K----------------LRLIDWGLAEFYHPGVDYNVRVASR
124 UniRef90_UPI000EA961A9_2_209       ---------K----------------LRLIDWGLAEFYHPGQEYNVRVASR
125 UniRef90_A0A165QAJ6_2_320          ---------K----------------LRLIDWGLAEFYHPDTEYPSHVGSR
126 UniRef90_A0A6J1ACU4_2_223          ---------K----------------LRLIDWGLAEFYHPSKEYNVRVASR
127 UniRef90_A0A146IBX2_2_322          ---------E----------------LRVIDWGLAEFYHPNEEYHHRVGSR
128 UniRef90_A0A1E5RA31_17_339         ---------E----------------LRLIDWGLAEFYHPGVDYNIRVASR
129 UniRef90_J9HLC0_29_343             ---------D----------------LRLIDWGLADFYKPGQEYNVRVASR
130 UniRef90_A0A183CIH8_805_1008       ---------Q----------------LRLIDWGLAEFYHPRQEYNVRVASR
131 UniRef90_A0A1M2V5N7_3_298          ---------K----------------LRLIDWGLAEFYHPGTELHIRVASR
132 UniRef90_A0A1B7TD73_22_342         ---------R----------------LRLIDWGLAEFYHPGVEYNVRVASR
133 UniRef90_A0A4P9VZ92_1_208          ---------Q----------------LRLIDWGLAEFYHPGTEYNVRVASR
134 UniRef90_W0T7F4_129_366            ---------K----------------LRLIDWGLAEFYHPSMEYNVRVASR
135 UniRef90_UPI000711954B_2_325       ---------D----------------VFIIDWGLADFYEPNKPMNVRVSTR
136 UniRef90_A0A1C7MA44_2_304          ---------K----------------LRLIDWGLAEFYHPGTELHIRVASR
137 UniRef90_C5DWN1_128_366            ---------K----------------LRLIDWGLAEYYHANMEYNVRVASR
138 UniRef90_A0A7H9B7A0_128_371        ---------K----------------LRLIDWGLAEFYHKNMEYNVRVASR
139 UniRef90_W5KQ02_73_295             --------------------------VQTCDWNCVLFLSVTSLCGISVLKC
140 UniRef90_A0A6P6YBM1_25_347         ---------K----------------LRLIDWGLAEYYLEGKFYNVRVASR
141 UniRef90_A0A196S5L7_3_325          ---------E----------------VYIIDWGLADYYKPHEKYNVRVSTR
142 UniRef90_A0A7S3FVA9_17_301         ---------Q----------------VRISDWGLADYYLPGKKFNCRVASR
143 UniRef90_A2FBX9_18_336             ---------K----------------LRLIDWGLAEFYHPETRYNIHVASR
144 UniRef90_UPI0010FB2831_6_323       ---------I----------------LKLIDWGLAEFYHPKTEYSVKVASR
145 UniRef90_Q8SRU0_6_317              ---------E----------------LKIIDWGLAEFYHPKKEYSVRVASR
146 UniRef90_E9ACB1_46_365             ---------I----------------LRVIDWGLGEYYIHGQALNCGVATR
```

```
147 UniRef90_W6KPE3_52_367       --------I----------------LRVIDWGLAEYYLHNEALNCGVATR
148 UniRef90_A0A1J4JEQ9_9_332    --------R----------------LRLLDWGLADFYHPRQKYNSHVAIR
149 UniRef90_G9N8S2_27_316       --------K----------------LRLFGWDYAEVHVPNCRYSVRVGRG
150 UniRef90_A0A4Z1TD09_8_331    --------K----------------LHLLDYGLAEFYSYGTTLHHRVASR


001 Input_pdb_SEQRES_A           YFKGPELLVDYQMYDYSLDMWSLGCMLASMIFRKEPFFHGHDNYDQLVRI
002 UniRef90_A0A060X538_2_334    YFKGPELLVDYQMYDYSLDMWSLGCMLASMVFRKEPFFHGHDNYDQLVRI
003 UniRef90_UPI000719C52A_2_332 YFKGPELLVDYQQYDYSLDMWSLGCMLASMIFRKEPFFHGHDNYDQLVRI
004 UniRef90_A0A7R8XAU3_2_332    YFKGPELLVDYQMYDYSLDMWSLGCMFASMIFRKEPFFHGHDNYDQLVRI
005 UniRef90_V3ZFM5_3_333        YFKGPELLLDYQMYDYSLDMWSLGCMFASMIFRKEPFFHGHDNYDQLVRI
006 UniRef90_UPI00193A3309_3_330 YFKGPELLVDYQYYDYSLDMWSLGCMLASMIFRKEPFFHGHDNYDQLVRI
007 UniRef90_A0A1Q3F4A5_3_329    YFKGPELLVDYQMYDYSLDMWSLGCMLASMIFRKEPFFHGHDNYDQLVRI
008 UniRef90_A0A4W3H6I6_2_321    YFKGPELLVDYQMYDYSLDMWSLGCMLASMIFRKEPFFHGHDNYDQLVRI
009 UniRef90_UPI000C6E8BEA_2_341 YFKGPELLADYQMYDYSLDMWSLGCMLASMIFRKEPFFHGHDNYDQLVRI
010 UniRef90_A0A0L0BLN9_116_444  YFKGPELLVDYQMYDYSLDMWSLGCMLASMIFRKEPFFHGHDNYDQLVRI
011 UniRef90_A0A183IEN2_2_329    YFKGPELLVDYQMYDYSLDMWSLGCMLASMIFRKEPFFHGHDNYDQLVRI
012 UniRef90_A0A5N4B3X1_3_332    YFKAPELLVDYQMYDYSLDMWSLGCMLASMIFRKEPFFHGHDNYDQLVRI
013 UniRef90_A0A556VUA8_53_369   YFKGPELLVDYQMYDYSLDMWSLGCMLASMVFRKEPFFHGHDNYDQLVRI
014 UniRef90_A0A1I8HE15_2_329    YFKGPELLLDYQMYDYSLDMWSLGCMFASMIFKKEPFFHGQDNYDQLVRI
015 UniRef90_F6RN85_7_328        YFKGPELLVDYQMYDYSLDMWSLGCMLASMIFRKEPFFHGQDNYDQLVRI
016 UniRef90_UPI0011E4E1F1_2_326 YFKGPELLVDYQMYDYSLDMWSLGCMFASMIFRKEPFFHGHDNYDQLVRI
017 UniRef90_A0A0P7V994_7_333    YFKGPELLVDYQMYDYSLDMWSLGCMLASMIFQKEPFFHGQDNYDQLVRI
018 UniRef90_A0A3Q4N194_2_309    YFKGPELLVDYQMYDYSLDMWSLGCMLASMIFRKEPFFHGHDNYDQ----
019 UniRef90_A0A0N5ANV2_2_328    YFKGPELLVDYQFYDYSLDMWSLGCMFASMIFRKEPFFHGHDNYDQLVRI
020 UniRef90_A0A0M3HMV3_2_324    YFKGPELLVDYQCYDYSLDMWSLGCMLASMIFRKEPFFHGHDNYDQLVRI
021 UniRef90_A0A2R7W6M3_5_330    YFKGPELLLDYQKYDYSLDMWSLGCMLASMIFKKEPFFHGCDNYDQLVRI
022 UniRef90_A0A368G0G8_241_571  YFKGPELLVDYQCYDYSLDMWSLGCMLASMIFRKEPFFHGHDNYDQLVRI
023 UniRef90_A0A5F4C0P0_224_555  YFKGPELLVDYQMYDYSLDMWSLGCMLASMIFRKEPFFHGQDNYDQLVRI
024 UniRef90_A0A671N1V7_3_326    YFKGPELLVDYQMYDYSLDMWSLGCMLASMIFQKEPFFHGQDNYDQLVRI
025 UniRef90_A0A667Y761_7_331    YFKGPELLVDYQMYDYSLDMWSLGCMLASMIFLKEPFFHGQDNYDQLVRI
026 UniRef90_I0Z462_2_325        YFKGPELLVDLQDYDYSLDLWSLGCMFAGMIFRKEPFFYGHDNYDQLVKI
027 UniRef90_A0A4W5MUS6_7_326    YFKGPELLVDYQMYDYSLDMWSLGCMLASMIFQKEPFFHGQDNYDQLVRI
028 UniRef90_UPI000C1CFB1C_2_322 YFKGPELLVDLQDYDYSLDMWSLGCMFAGMIFRKEPFFYGHDNHDQLVKI
029 UniRef90_A0A200QFN8_2_323    YFKGPELLVDLQDYDYSLDMWSLGCMFAGMIFRKEPFFYGHDNQDQLVKI
030 UniRef90_A0A6I9QC06_84_406   YFKGPELLVDLQDYDYSLDMWSLGCMFAGMIFRKEPFFYGHDNYDQLVKI
031 UniRef90_A9NVF1_102_427      YFKGPELLVDLQDYDYSLDMWSLGCMFAGMIFRKEPFFYGHDNYDQLVKI
032 UniRef90_A0A6S7M9R1_74_394   YFKGPELLVDLQDYDYSLDMWSLGCMFAGMIFRKEPFFYGHDNQDQLVKI
033 UniRef90_A0A5J4ZXR3_110_433  YFKGPELLVDLQDYDYSLDLWSLGCMFAGMIFRKEPFFYGHDNYDQLVKI
034 UniRef90_A0A0D2WV07_2_326    YFKGPELLVDFQEYDYSLDMWSLGCMFASMIFRKEPFFHGHDNYDQLIKI
035 UniRef90_A0A2P6Q0W9_107_428  HYKGPELLVDLQDYDYSLDMWSLGCMFAGMIFRKEQFFYGHDNQDQLVKI
036 UniRef90_A0A2J6LRB0_100_422  YFKGPELLVDLQDYDYSLDLWSLGCMFAGMIFRKEPFFYGHDNHDQLVKI
037 UniRef90_A0A4S8ISY9_72_395   YFKGPELLVDLQDYDYSLDMWSLGCMFAGMIFRKEPFFYGHDNYDQLVKI
038 UniRef90_A0A1U8LZW6_92_416   YFKGPELLVDLQDYDYSLDLWSLGCMFAGMIFRKEPFFYGRDNYDQLVKI
039 UniRef90_K3XBI1_15_338       YFKGPELLVDMQEYDYSLDMWSLGCMFAGMIFRKEHFFRGNDNFDQLVKI
040 UniRef90_E1ZRP6_14_337       YFKGPELLVDLQDYDYSLDMWSLGCMLAGMIFRKEPFFYGHDNYDQLVKI
041 UniRef90_A0A2I0AFK3_70_391   YFKGPELLVDLQDYDYSLDMWSLGCMFAGMIFHKEPFFYGHDNYDQLVKI
042 UniRef90_UPI000F7CDB20_80_401 YFKGPELLVDLQDYDYSLDLWSLGCMFAGMIFRKEPFFYGQDNYDQLVKI
043 UniRef90_G7K3U9_82_403       YFKGPELLIDLQDYDYSLDLWSLGCMFAGMIFRKEPFFYGQDNYDQLVKI
044 UniRef90_A0A663MTN9_26_324   YFKGPELLVDYQMYDYSLDMWSLGCMLASMIFRKEPFFHGQDNYDQLVRI
045 UniRef90_A4S5X1_14_334       YFKGPELLVDLQDYDYSLDMWSLGCMFAGMIFRKEPFFAGHDNYDQLVRI
046 UniRef90_A0A183LBY6_9_327    YFKGPELLVDYQMYDYSLDLWSLGCVFASMIFRKEPFFHGHDNYDQLVRI
047 UniRef90_A0A1J9RPL1_99_422   YFKGPELLVDFQEYDYSLDMWSLGAMFASMIFRKEPFFHGNSNSDQLVKI
048 UniRef90_D5GJ91_2_320        YFKGPELLVDYQEYDYSLDLWSLGAMFASMIFRKEPFFHGNSNSDQLVKI
049 UniRef90_A0A4W4DPG2_3_315    SYKGPELLVDYQMYDYSLDMWSLGCMLASMIFQKEPFFHGQDNYDQLVRI
050 UniRef90_A0A7S2RD37_2_324    YFKGPELLVDLQDYDYSLDMWSLGAMFAGMIYHREPFFHGHDNYDQLVKI
051 UniRef90_A0A367JQL9_12_336   YFKGPELLVDFQEYDYSLDMWSYGCMFASMIFRKEPFFHGHDNYDQLVKI
052 UniRef90_UPI001924B863_29_350 YYKGPELLVDLQDYDYSLDLWSLGCMFAAMIFQKEPFFYGHDNQD-LVKI
053 UniRef90_A0A367KWU7_10_331   YFKGPELLVDFQEYDYSLDLWSYGCMFASMIFRKEPFFHGHDNYDQLVKI
054 UniRef90_A0A0D2AMV0_2_320    YFKGPELLVDYQEYDYSLDMWSLGAMFASMIFRKEPFFHGNSNPDQLVKI
055 UniRef90_A0A1Y1WVY1_2_324    YFKGPELLVDLQAYDYSLDMWSLGCMFAGMIFRKDPFFQGHDNYDQLVKI
056 UniRef90_Q8TG13_2_324        YFKGPELLVDFQEYDYSLDMWSLGAMFASMIFRKEPFFHGQSNSDQLVKI
```

```
057 UniRef90_A0A0F8B0Y9_1_322      YFKGPELLVDFQEYDYSLDMWSLGAMYASMIFRKEPFFHGNSNSDQLVKI
058 UniRef90_A0A7S1NI55_6_324      YFKGPELLVDLRDYDYSLDMWSLGCMFAGMIFRKEPFFHGRDNCDQLVKI
059 UniRef90_A0A383VX45_13_335     YYKGPELLVDLQDYDYGLDLWSLGCMLAALLFRKDPFFCGHDNYDQLAKI
060 UniRef90_I1C1X5_16_338         YFKGPELLVDFQEYDYSLDMWSYGCMFASMIFRKEPFFHGHDNYDQLVKI
061 UniRef90_A0A364LA26_2_320      YFKGPELLVDFQEYDYSLDMWSLGAMFASMIFRKEPFFHGISNADQLVKI
062 UniRef90_A0A1X7RU70_2_320      YFKGPELLVDFQEYDYSLDMWSLGAMFASMIFRKEPFFHGNSNSDQLVKI
063 UniRef90_A0A067BWS1_2_324      YFKGPELLVDMQEYDYSLDMWSVGCVLAGMIFKKEPFFHGHDNYDQLVKI
064 UniRef90_A0A0P9FCQ6_6_328      YFKGPELLVDFQEYDYSLDMWSLGCMFASMIFRKEPFFHGHSNEDQLVKI
065 UniRef90_A0A0K3CS88_10_335     YFKGPELLVDFQEYDYSLDMWSLGCMFASMIFRKEPFFHGHSNEDQLVKI
066 UniRef90_A0A4S4KUV1_8_328      YFKGPELLVDFQEYDYSLDMWSYGCMFASMIFRKEPFFHGHDNYDQLVKI
067 UniRef90_UPI000511B7B1_2_322   HYKGPELLVDLQDYDHSLDLWSLGCMFAGMIFRKEQFFYGHDNQDQLVKL
068 UniRef90_A0A5C3QYQ7_5_326      YFKGPELLVDFQEYDYSLDMWSFGCMFASMIFRKEPFFHGHDNYDQLVKI
069 UniRef90_J4GNM1_5_326          YFKGPELLVDFQEYDYSLDMWSYGCMFASMIFRKEPFFHGHDNYDQLVKI
070 UniRef90_A0A1B6QHX1_78_406     CYKGPELLVDLLDYDYSLDLWSLGCMFAAMIFRVDPFFSGQDNYDQLVKI
071 UniRef90_E3Q428_2_328          YFKGPELLVDFQEYDYSLDMWSLGAMFASMIFRKEPFFHGNSNSDQLVKI
072 UniRef90_A0A161HHJ2_5_327      CFKGPELLVDLQMYDYSLDMWSLGAMFASMIFKKDPFFHGSSNSDQLVKI
073 UniRef90_A0A0C2TUQ3_4_328      YFKGPELLVDFQEYDYSLDMWSFGCMFASMIFRKEPFFHGHDNYDQLVKI
074 UniRef90_UPI000644E1AF_26_344  PYKGPELLVDMEDYDYSLDMWSLGCMFAGILFGRDPFFHGHDNIDQLVKI
075 UniRef90_F2U7H7_13_336         YFKGPELLVNFEEYDYSLDLWSFGCVMASMIFRKEPFFHGHDNYDQLIKI
076 UniRef90_A0A1E3Q523_2_312      YFKGPELLVDFQEYDYSLDMWSLGAMFASMIFRKEPFFHGNSNTDQLVKI
077 UniRef90_A0A446UN67_26_313     YFKGPELLVDLQDYDYSLDMWSLGCMFAGMIFRKEPFFYGHDNHDQLVKI
078 UniRef90_A0A1E4RU21_6_330      YFKGPELLVDFRLYDYSLDLWSYGCMFASMVFKKEPFFHGKSNTDQLVQI
079 UniRef90_A5DCV5_5_327          YFKGPELLVDFRLYDYSLDLWSFGCMLASMVFKKEPFFHGKSNTDQLVQI
080 UniRef90_L8GRW8_10_332         HYKGPELLVGYQMYDYSLDMWSLGCMFAGILFNKQPFFCGSDNQNQLVKI
081 UniRef90_A3GID5_8_329          YFKGPELLVDFRLYDYSLDMWSYGCMLASMVFMKEPFFHGKSNTDQLVQI
082 UniRef90_A0A2V1AT92_3_322      YFKGPELLVDFRLYDYSLDMWSYGCMLASMVFKKDPFFHGKSNTDQLVQI
083 UniRef90_A0A642UHU8_5_327      YFKGPELLVNFRLYDYSLDLWSFGCMLASMVFMKEPFFHGKSNTDQLVQI
084 UniRef90_UPI001036488A_60_387  YFKGPELLVDLQDYDYSMDMWSLGCMFAGMIFRKEPFFYGHDNHDQLVKI
085 UniRef90_A0A448YKC4_4_323      YFKGPELLVDFRYYDYSLDIWSFGATLASIIFKKEPFFHGKSNTDQLVQI
086 UniRef90_UPI001900E851_4_329   YFKGPELLVDYRYYDYSLDIWSFGATFASIIFKKEPFFHGKSNTDQLVQI
087 UniRef90_A0A4P9ZDD8_3_324      YFKGPELLADFRLYDYSLDLWSFGCMLASIVFKKEPFFHGKSNTDQFVQI
088 UniRef90_UPI00064AE4FC_6_334   FYKGPELLVEYEMYDYSLDMWSLGCVLASMIFRKDPFFAGSNNNDQLLKI
089 UniRef90_A0A7S3CI51_5_325      YFKGPELLIDLQTYDYSLDIWSLGCMFAGMIFQKEPFFQGKDNYDQLVKI
090 UniRef90_UPI00098E15C8_59_372  YYKGPELLVDLQDYDYSLDLWSLGCMFAGMIFRKDPFFYGQDNHDQLVKI
091 UniRef90_A5E6J5_9_319          YFKGPELLVDYRLYDYSLDLWSFGCMLASMVFMKEPFFHGKSNTDQLVQI
092 UniRef90_A0A0S4KFI2_14_334     YFKGPELLVELPMYDYSLDMWSLGCMMAGMIFMKEPFFHGKDNNDQLVRI
093 UniRef90_A0A4Y7PMM1_7_330      FWKGPEVLVDYRKYNYSLDMWSVGCIFASMIFRKEPFFHGHDNYDMLVNI
094 UniRef90_A0A674DLQ6_2_238      YFKGPELLVDYQMYDYSLDMWSLGCMLASMIFRKEPFFHGHDNYDQV---
095 UniRef90_A0A367YK45_12_333     YFKGPELLVDFRWYDYSLDLWSFGCMLASMVFQKEPFFHGKSNTDQLVQI
096 UniRef90_Q6BUZ4_8_327          YHKGPELLVNLQQYDYSLDLWSVGCMLAAIIFKKEPFFRGDSNNDQLVQI
097 UniRef90_A0A7S3F0G7_17_281     YFKGPELLVDLQDYDYALDMWSLGCMFAGMIFRKEPFFHGHDNYDQLVKI
098 UniRef90_A0A0C3HJB5_2_320      -FKPPELLVDFQEYDYSLDMWSLGAMFASMIFRKEPFFHGNSNSDQLIKI
099 UniRef90_A0A1R2D4Q4_3_325      YFKGPELLVDDELYYYSLDIWSLGAMLAGIIFQKDPFFHGRDNQDQLVKI
100 UniRef90_UPI0004F4A9E2_17_332  YFKGPELLVELPMYDYRLDMWSLGCMLAGIIFMKEPFFHGKDNNDQLVHI
101 UniRef90_A0A4P6XGL7_6_324      YHKGPELLINLQQYDYSLDLWSVGCMLAAIIFKKEPFFRGDSNNDQLVQI
102 UniRef90_A0A6P6RSI1_108_381    YYKGPELLVDLQVYDYSLDIWSLGCMLAGIIFKKEPFFYGHDNYDQLVKI
103 UniRef90_A0A4U0VRL3_43_361     YYKAPELLVEYTHYDYSLDLWSVGCTFGTMIFHRDPLFHGSSNEDQLVKI
104 UniRef90_K0KNM3_9_329          YHKGPELLVNLNQYDYSLDLWSVGAMLAAIIFKKEPFFRGDSNFDQLIKI
105 UniRef90_A0A2X0MBX0_6_345      YYKGPELLVEFGYYDYSLDMWSVGCTFGSMIFRRDPLFHGHDNNDQLCKI
106 UniRef90_A0A7S1VNL6_68_384     YFKGPELLTDMQDYDYSLDLWSTGCVLAGIIFRREPFFHGRDNADQLCKI
107 UniRef90_A0A2H9TP39_34_357     YYKGPELLVDLFEYDYSLDMWSLGCMFGGMIFMMDTLFHGRDNNDQLFKI
108 UniRef90_A0A315WAV6_110_366    YFKGPELLVDYQMYDYSLDMWSFGCMLASMIFLKEPFFHGQDNYDQV---
109 UniRef90_UPI001386DBE1_151_468 YFKGRELLVGDQRYDYSLDMCSLGRMLASTIFREQPFFHGRDTYDQLVRI
110 UniRef90_A0A5D2A803_51_293     YFKGPELLIDLQDYDYSLDMWSLDCMFAGMIFRKEPFFYGHDNNDQLVKI
111 UniRef90_A0CQX8_4_329          YFKSPELLLDYQYYDYSLDIWSLGSLFAGIIFQQEPFFHGQDNNDQLDKI
112 UniRef90_T1EXU0_70_294         YFKGPELLLDYQLYDYSLDLWSLGCMFASMIFKKEPFFHGHDNYDQLVRI
113 UniRef90_A0A7H9B7U0_15_336     YHKGPELLVNLNQYDYSLDLWSVGCMLAAIIFKREPFFKGSTNADQLIKI
114 UniRef90_A0A482VYB7_21_342     HYKSPELLVEYGYYDYSLDLWSLGCVFAAMIFRKEPFFQGFDNRDQLYCI
115 UniRef90_A0A1D2VEG0_6_324      YHKGPELLVNLQQYDYSLDLWSVGAMLAAIIFKIEPFFRGNSNPDQLVKI
116 UniRef90_A0A0D0B3X6_2_320      YYKAPELLVGYKLYDYSLDLWSIGCMLASMLFRKEHFFRGSDNNDQLLKI
117 UniRef90_A0A1G4MEF4_15_334     YHKGPELLVNLNQYDYSLDLWSVGCMLAAIVFKKEPFFKGSTNPDQLVKI
118 UniRef90_A0A2V0NTF2_16_339     FYKGPELLLDIRDYDYSLDIWGVGCMLAAFLFKRQVFFRGEDEFDQLVRI
119 UniRef90_A0A4Y9YFI0_2_320      YWKGPELLVSHRTYDYSLDLWSVGCILAALVFRKEAFFRGRDNEDQLLKI
120 UniRef90_W6KIF7_13_329         YFKGPELLIELPTYDYRLDMWSLGCMMAGIIFMKEPFFRGKDNTDQLVRI
```

```
121 UniRef90_A0A0C3NKK6_2_320      PYKSPELLVGYSMYDYSLDLWCVGCMLAAMIFRKEHFFRGRDNEEQLARI
122 UniRef90_A0A0D7AGR9_2_320      YYKAPELLVGYRQYDYSLDLWSLGCMFASMIFRKEHFFRGSDNDDQLLKI
123 UniRef90_G0VHQ2_23_343         YHKGPELLTDLNQYDYSLDMWSVGCMIAAIIFKKEPFFKGGSNPDQLVKI
124 UniRef90_UPI000EA961A9_2_209   YFKGPELLVDYQVS------------------------------------
125 UniRef90_A0A165QAJ6_2_320      HYKSPELLVAYRTYDYSLDLWSVGCVLAALVFRKEAFFRGRDNEDQLIKI
126 UniRef90_A0A6J1ACU4_2_223      YFKGPELLVDLQDYDYSLDMWSLGCMFAGMV-------------------
127 UniRef90_A0A146IBX2_2_322      HYKSPELLVGYKRYDYSLDLWSTGCMFASMIFRKQHFFRGSDNEDQLLKI
128 UniRef90_A0A1E5RA31_17_339     YHKGPELLVNLKQYDYSLDLWSVGCMIGGIIFNKDPLFKGDSNPDQLIKI
129 UniRef90_J9HLC0_29_343         YYKGPELLVDDKLYHYSLDMWSLGCTMAGMTLRIDTFFKGNDNEDQLLKI
130 UniRef90_A0A183CIH8_805_1008   YFKGPELLVDYQ--------------------------------------
131 UniRef90_A0A1M2V5N7_3_298      YFKGPELLVGYKHYDYSLDIWSVGCILASMIFRREHFFRGRDNDDQLLKI
132 UniRef90_A0A1B7TD73_22_342     HYKGPELLINLRQYDYSLDLWSVGCVLASIMFNVEPLFKGDSNTNQLIKI
133 UniRef90_A0A4P9VZ92_1_208      YFKGPELLVDFQEYDYSLDMWSLGCMFASMIFRKEPFFHGHDNYDQLVKI
134 UniRef90_W0T7F4_129_366        FFKGPELLVDYRMYDYSLDLWSFGAMLASMVFLKEPFFYGSSNTDQLVKI
135 UniRef90_UPI000711954B_2_325   NYKGPELLTNYELYDYSVDMWSFGCMVAGIVFNRLPFFRAHDNMEQLRI
136 UniRef90_A0A1C7MA44_2_304      YYKGPELLVGFKHYDYSLDMWSVGCILASMIFRKEHFFRGRDNEDQLLKI
137 UniRef90_C5DWN1_128_366        FFKGPELLVDYRMYDYSLDLWSFGTMLASLVFQKEPFFHGTSNTDQLVKI
138 UniRef90_A0A7H9B7A0_128_371    FFKGPELLVDYRMYDYSLDIWSFGTMLASMVFQKEPFFHGSSNTDQLVKI
139 UniRef90_W5KQ02_73_295         MY--FFLIVLVQMYDYSLDMWSLGCMLASMIFQKEPFFHGQDNYDQLVRI
140 UniRef90_A0A6P6YBM1_25_347     FYKAPELLTYLYVYDYNIDMWSVGCILAALMFRRDPFFKGADNVDQLVKI
141 UniRef90_A0A196S5L7_3_325      NYKGPELLTNDNDYDYSLDIWSLSCMLLGIVFNRTPFFRGKDNFEQLRKI
142 UniRef90_A0A7S3FVA9_17_301     FFKGPELLLGMSYYDYQLDVWSTGCILAGMMFQREPFFKGQDNFDQMIKI
143 UniRef90_A2FBX9_18_336         HFKPPELLLDYQYYDYSIDMWSFGVTMAGLIFKKTPFFRGNDDLDMVNKI
144 UniRef90_UPI0010FB2831_6_323   FYKGPELLVSYGYYDYSLDIWSFGCILAEIIFKKHPFFCGEDNTDQLMKI
145 UniRef90_Q8SRU0_6_317          YYKGPELLVDYPYYDYSLDIWSFGCVLAELVFKKRPFFHGESNSDQLVKI
146 UniRef90_E9ACB1_46_365         HYKGPELLVGYRHYDYSLDIWCLGCVLAGMLFRSDPFFVGANNEDQLLQI
147 UniRef90_W6KPE3_52_367         HYKGPELLLGYRHYDFSLDIWCLGCVFAGIIFHADPFFDGYSNEDQLLRI
148 UniRef90_A0A1J4JEQ9_9_332      IFKPPELLINYPYYDYSMDLWSTGLTFSIMMFKRIVMECGENDADQLLKV
149 UniRef90_G9N8S2_27_316         FHKAPELLLQYEEYDCSVDIWNVGVVIASMVFRKEPFFHGASNFHILQAI
150 UniRef90_A0A4Z1TD09_8_331      HFKSPELLVGYQLYDYSLDIWSLGAMAAGMIFRRNPFFRGTNNVNQLDKI


001 Input_pdb_SEQRES_A             AKVLGTEDLYDYIDKYNIE--LDPRFNDIL--G-----------RHSRK-
002 UniRef90_A0A060X538_2_334      AKVLGTEDLYDYIDKYNIE--LEPRFNDIL--G-----------RHSRK-
003 UniRef90_UPI000719C52A_2_332   AKVLGTEELYEYIDKYQIE--LDPRFNDIL--G-----------RHSRK-
004 UniRef90_A0A7R8XAU3_2_332      AKVLGTEELFEYLDKYQIE--LDPRFNDIL--G-----------RHSRK-
005 UniRef90_V3ZFM5_3_333          AKVLGTDDLYAYIEKYQID--LDQRFNDIL--G-----------RHSRK-
006 UniRef90_UPI00193A3309_3_330   AKVLGTEDLFDYIEKYNIE--LDPRFNDIL--G-----------RHSRK-
007 UniRef90_A0A1Q3F4A5_3_329      AKVLGTEDLFAYLDKYNIE--LDPRFNDIL--S-----------RHSRK-
008 UniRef90_A0A4W3H6I6_2_321      AKVLGTEDLYDYIDKYNIE--LDPRFNDIL--G-----------RHSRK-
009 UniRef90_UPI000C6E8BEA_2_341   AKVLGTEELYEYLDKYQVE--LDPRFNDIL--GRKNDKSDLLPSRHSRK-
010 UniRef90_A0A0L0BLN9_116_444    AKVLGTEELYAYLDKYNIE--LDPRFHDIL--Q-----------RHSRK-
011 UniRef90_A0A183IEN2_2_329      AKVLGTEELYDYLDKYQIE--LDPRFNDIL--GRNS-------FRHSRK-
012 UniRef90_A0A5N4B3X1_3_332      AKVLGTDELFEYLEKYHIQ--LDSRFNDIL--G-----------RHSRK-
013 UniRef90_A0A556VUA8_53_369     AKVLGTEDLYDYIDKYNIE--LDPRFNDIL--G-----------RHSRK-
014 UniRef90_A0A1I8HE15_2_329      AKVLGTDDLYAYINKYQIV--LDPRFNDIL--A-----------KHSRK-
015 UniRef90_F6RN85_7_328          AKVLGTDELYSYLKKYHIE--LDPHFNDIL--G-----------QHSRK-
016 UniRef90_UPI0011E4E1F1_2_326   AKVLGTKELYEYLERYQIE--LDPRFNDIL--G-----------SHTRK-
017 UniRef90_A0A0P7V994_7_333      AKVLGTDELFGYLRKYRIE--LDPRFKELL--G-----------QQTRK-
018 UniRef90_A0A3Q4N194_2_309      --VLGTEDLYDYIDKYNIE--LEPRFNDIL--G-----------RHSRK-
019 UniRef90_A0A0N5ANV2_2_328      AKVLGTEDLYSYIDKYQIE--LDPRYNELL--G-----------RHSKK-
020 UniRef90_A0A0M3HMV3_2_324      AKVLGTEELFEYIDKYQIE--LDPRFNDIL--G-----------RHSKK-
021 UniRef90_A0A2R7W6M3_5_330      AKVLGTEELYEYLDKYLIK--LDLRFAGIL--S-----------RHSRK-
022 UniRef90_A0A368G0G8_241_571    AKVLGTEELHEYIDKYHIE--LDPRFNDIL--G-----------RHSRK-
023 UniRef90_A0A5F4C0P0_224_555    AKVLGTDELYGYLKKYHID--LDPHFNDIL--G-----------QHSRK-
024 UniRef90_A0A671N1V7_3_326      AKVLGTDELFGYLRKYHIE--LDPRFKDLL--G-----------QQTRK-
025 UniRef90_A0A667Y761_7_331      AKVLGTDELFGYLHKYHIE--LDTRFKDLL--G-----------QQTRK-
026 UniRef90_I0Z462_2_325          AKVLGTEELDAYLQKYGIE--LDAQLEELV--G-----------RHSRK-
027 UniRef90_A0A4W5MUS6_7_326      AKVLGTDELFGYLRKYHIE--LDPRFKDLL--G-----------QQTRK-
028 UniRef90_UPI000C1CFB1C_2_322   AKVLGTDELNAYLNKYHLE--LDPQLDALV--G-----------RHSRK-
029 UniRef90_A0A200QFN8_2_323      ARVMGTDDLNSYLNKYRLE--LDPQLDALV--G-----------RHSRK-
030 UniRef90_A0A6I9QC06_84_406     AKVLGTDELNAYLNKYRLE--LDPHLEALV--G-----------RHSRK-
```

```
031 UniRef90_A9NVF1_102_427          AKVLGTDELNAYLNKYRIE--LDPHLEALV--G-----------RHSRK-
032 UniRef90_A0A6S7M9R1_74_394       ARVLGTDELNAYLNKYQLE--LEPQLEALV--G-----------RHSRK-
033 UniRef90_A0A5J4ZXR3_110_433      AKVLGTDELNAYLSKNRLE--LDPHLAALV--G-----------RHSRK-
034 UniRef90_A0A0D2WV07_2_326        AKVLGTDELFAYLDKYNIN--LGAHYDNIL--G-----------RYPRK-
035 UniRef90_A0A2P6Q0W9_107_428      AKVLGTDELHAYLTKYHLV--LEPQLEALV--G-----------SHSRK-
036 UniRef90_A0A2J6LRB0_100_422      AKVLGTDELNTYLQKYRIE--LDPNLSELV--G-----------RHNRK-
037 UniRef90_A0A4S8ISY9_72_395       AKVLGTDELNAYLKKYQLE--LDPQLEALV--G-----------RHSRK-
038 UniRef90_A0A1U8LZW6_92_416       AKVLGTDELNAYLNKYGIE--LDPHLLFLV--G-----------RHSHK-
039 UniRef90_K3XBI1_15_338           AKVKGTDELFDYLTTYDLE--LDPQYDGIL--G-----------THTKK-
040 UniRef90_E1ZRP6_14_337           CKVLGTDSFYAYLNKYGLE--LDPQLEALV--G-----------RHSRK-
041 UniRef90_A0A2I0AFK3_70_391       AKVLGTDDLTAYLNKYRLE--LDPQLSSLV--G-----------RHSRK-
042 UniRef90_UPI000F7CDB20_80_401    AKVLGTDELKAYLHKYRIE--LDPHLAVLV--G-----------KHGRK-
043 UniRef90_G7K3U9_82_403           AKVLGTDELRAYQDKYHIE--LDPRYAVLI--G-----------RHDRK-
044 UniRef90_A0A663MTN9_26_324       AKVLGTDELYGYLKKYHIE--LDPHFNDIL--G-----------QHSRK-
045 UniRef90_A4S5X1_14_334           AKVLGTDELYAYLNKYRIE--LDPNFETLI--G-----------RHSRK-
046 UniRef90_A0A183LBY6_9_327        AKVLGTEELFQYLTKYEIV--LDPRFNEIL--G-----------RHTKK-
047 UniRef90_A0A1J9RPL1_99_422       AKVLGTEDLFDYLDKYDIE--LDAQYDDIL--G-----------RFPKK-
048 UniRef90_D5GJ91_2_320            AKVLGTEELFDYLDKYDIE--LDTQYDDIL--G-----------RFPKK-
049 UniRef90_A0A4W4DPG2_3_315        AKVLGTEELFSYLNKYHIE--LDTRFKDLL--G-----------QQTRK-
050 UniRef90_A0A7S2RD37_2_324        AKVLGTDELLSYLEKYDLE--IDPHFDGVL--G-----------KHSKK-
051 UniRef90_A0A367JQL9_12_336       ARVLGTDDLFRYTAKYSIK--LDPEYDNIL--G-----------RHLRK-
052 UniRef90_UPI001924B863_29_350    AKVLGTDGLNAYLNKYHLE--LDLELDALV--G-----------RHSCK-
053 UniRef90_A0A367KWU7_10_331       ARVLGTDELFKYTAKYAIK--LDSEYDTIL--G-----------RHIRK-
054 UniRef90_A0A0D2AMV0_2_320        AKVLGTEELFDYLDKYDIE--LDQQYDDIL--G-----------RFPKK-
055 UniRef90_A0A1Y1WVY1_2_324        ARVLGTDELHKYLDKYSIK--LDARYNDIL--G-----------KYPRK-
056 UniRef90_Q8TG13_2_324            AKVLGTDELFDYLDKYEIE--LDAQYDDIL--G-----------RFQRK-
057 UniRef90_A0A0F8B0Y9_1_322        AKVLGTDALFDYLDKYEIE--LDPQYDDVL--G-----------RFPKK-
058 UniRef90_A0A7S1NI55_6_324        AKVLGTDDLFAYLDKYSLE--LSPHYDGIL--S-----------RYPKK-
059 UniRef90_A0A383VX45_13_335       ARVLGSEALFEYLGKYGLE--LDPQLEALV--G-----------HHSRK-
060 UniRef90_I1C1X5_16_338           ARVLGTDELFKYTEKYQIK--LDSEYDNIL--G-----------KYSRK-
061 UniRef90_A0A364LA26_2_320        AKVLGTEELFEYLDKYDIE--LDSQYDDIL--S-----------RYPKK-
062 UniRef90_A0A1X7RU70_2_320        AKVLGTEDLFDYLDKYDIE--LDAQYDDIL--G-----------RFPKK-
063 UniRef90_A0A067BWS1_2_324        AKVRGTEDLFEYLSTYDLE--LDAQYDGIL--G-----------THSKK-
064 UniRef90_A0A0P9FCQ6_6_328        TKVLGTDDLYAYLEKYDID--LAPEFDDIL--G-----------RYAKK-
065 UniRef90_A0A0K3CS88_10_335       TKVLGTDELYEYLEKYDIE--LAPEFDDIL--G-----------RYAKK-
066 UniRef90_A0A4S4KUV1_8_328        AKVLGTDELYRYIEKYDIA--LDSQYDEIL--G-----------NYPKK-
067 UniRef90_UPI000511B7B1_2_322     LKVLGTEELNVYLNKYNLV--LDPQLDVLV--G-----------RYTSQ-
068 UniRef90_A0A5C3QYQ7_5_326        TKVLGTDEFYLYVDKYSIR--LDPQYDEIL--G-----------RYPRK-
069 UniRef90_J4GNM1_5_326            TKVLGTQGLYDYVEKYDIA--LDPQYDEIL--Q-----------SYPKK-
070 UniRef90_A0A1B6QHX1_78_406       AEVLGTGDLYNYLEKYGLQ--LDPQLERLV--G-----------RHNRK-
071 UniRef90_E3Q428_2_328            AKVLGTDDLFDYLDKYDIE--LDAQYDDIL--G-----------RFQKK-
072 UniRef90_A0A161HHJ2_5_327        AKVLGTDELYQYLYKYHLT--LDSQYDKIL--G-----------SYPRR-
073 UniRef90_A0A0C2TUQ3_4_328        TKVLGTDELYVYIEKYSIR--LDPQYDELL--Q-----------RYPRK-
074 UniRef90_UPI000644E1AF_26_344    VKILGTEEFYRYLDKYGIV--IDNSLQNIL--G-----------KHPKK-
075 UniRef90_F2U7H7_13_336           AKVLGTDDLHAYIEKYKIV--LDQHYHQLL--P--N-------VRTAPK-
076 UniRef90_A0A1E3Q523_2_312        ARVLGTDSLNAYLEKYDIE--LDPQYDELI--G-----------RFARK-
077 UniRef90_A0A446UN67_26_313       AKVLGTDSLNAYLKKYHLE--LDPQLEHLV--G-----------RHSRK-
078 UniRef90_A0A1E4RU21_6_330        VRVLGSDDLHKYLQKYNLT--LSEEYED-L--G-----------YYNRR-
079 UniRef90_A5DCV5_5_327            VRVLGSDDLHKYLQKYGLV--LSEEYED-L--G-----------YYNRR-
080 UniRef90_L8GRW8_10_332           AKVLGTEKLYAYLKKYNIQ--LDARFRSAI--G-----------TRQRK-
081 UniRef90_A3GID5_8_329            VRVLGSSSLNKYLAKYNLT--LSEEYED-I--G-----------YYNRR-
082 UniRef90_A0A2V1AT92_3_322        VRVLGSDDLHSYLQKYNLT--LSEEYED-L--G-----------YYNRR-
083 UniRef90_A0A642UHU8_5_327        VRVLGSNALNAYLEKYNLT--LSDEYED-L--A-----------YYNRR-
084 UniRef90_UPI001036488A_60_387    AKVLGTDELNAYLNKYHLE--LDPQLDALV--G-----------RHSRK-
085 UniRef90_A0A448YKC4_4_323        ARVLGTDDLYKYLDKYGLK--LGPEYED-L--G-----------HYVRR-
086 UniRef90_UPI001900E851_4_329     ARVLGTDELYKYLDKYGLK--LGPEYED-L--G-----------HYVRR-
087 UniRef90_A0A4P9ZDD8_3_324        VRVLGSDDLHIYLEKYGLT--LAEEYED-L--G-----------YYSRR-
088 UniRef90_UPI00064AE4FC_6_334     VSILGTNRFYAYIQKYRIK--LDKQLRIIV--G-----------KHPRV-
089 UniRef90_A0A7S3CI51_5_325        AKVLGTDGIFSYVEKYNVT--LDSHYDDIL--G-----------QHPKK-
090 UniRef90_UPI00098E15C8_59_372    AKVLGTEELNLYLSKYRLA--LDPCLEALV--G-----------RHSRK-
091 UniRef90_A5E6J5_9_319            VRVLGSSNLNKYLEKYNLT--LGDEYED-M--G-----------YYTRR-
092 UniRef90_A0A0S4KFI2_14_334       AKVLGTDDLYLYLQTYQLT--LQPNLAQAV--G-----------RHSRK-
093 UniRef90_A0A4Y7PMM1_7_330        AKVLGSDDLFAYLEKYDME--IDVAYDDLLVPG-----------RYPRK-
094 UniRef90_A0A674DLQ6_2_238        --------------------------------------------------
```

```
095 UniRef90_A0A367YK45_12_333     VRVLGSDAFGAYIEKYQIT--LGEEYED-L--G-----------YYNKR-
096 UniRef90_Q6BUZ4_8_327          AKVLGTKDLLNYVNKYGIK--LSDEYDSIL--G-----------NYPRK-
097 UniRef90_A0A7S3F0G7_17_281     VKVLGTDELFDYLDKYNLE--LDPQ-------------------------
098 UniRef90_A0A0C3HJB5_2_320      AKILGTDGLFSYLEKYGIQ--LDQQYDDIL--G-----------RFTKQ-
099 UniRef90_A0A1R2D4Q4_3_325      AKILGTQELFKYLDKYGLE--LSSQLEKMV--G-----------KHVVK-
100 UniRef90_UPI0004F4A9E2_17_332  AKVLGTDELFEYLRKYNLT--LPPLLDAAV--G-----------RHTKK-
101 UniRef90_A0A4P6XGL7_6_324      AKVLGTKDLMAYVNKYRIK--LSDEYDSIL--G-----------KYPRK-
102 UniRef90_A0A6P6RSI1_108_381    AKVLGTDGLYAYLEKYDLE--LEKNFAQLV--G-----------KHTKK-
103 UniRef90_A0A4U0VRL3_43_361     ARVLGTDDLWQYLDRYNLD--LDPEFEAQI--G-----------THARK-
104 UniRef90_K0KNM3_9_329          AKVLGTDELYVYLKKYGLR--LSKEYNEVL--T-----------PCPRK-
105 UniRef90_A0A2X0MBX0_6_345      TRVLGTTDFWAYLDKYQIE--IDANLDALI--G-----------THSRK-
106 UniRef90_A0A7S1VNL6_68_384     ARVLGTEDLYKWVAKYGLT--IDPALQAAI--G-----------RHARK-
107 UniRef90_A0A2H9TP39_34_357     VEKLGSDELFKYLNKYSLR--LGKQYAALQ--S----------THYPVV-
108 UniRef90_A0A315WAV6_110_366    --------------------------------------------------
109 UniRef90_UPI001386DBE1_151_468 AKVLAPDEPYGCLKKDHID--LDPRFNHIL--G-----------QYSRK-
110 UniRef90_A0A5D2A803_51_293     AK------------------------------------------------
111 UniRef90_A0CQX8_4_329          ARVLGTDDLMNYLQKYNIS--LGPNFENVL--G-----------QHPKK-
112 UniRef90_T1EXU0_70_294         AKVLGTEELLEYIDKYQIE--LDQRFHDIL--G-----------RHSRK-
113 UniRef90_A0A7H9B7U0_15_336     AAVLGTKPLLAYLEKYGLK--LPSEYDNLM--R-----------DFTTK-
114 UniRef90_A0A482VYB7_21_342     IKVLGTTKFYSYLSKYNIT--LESSLQETL--G-----------IHSKK-
115 UniRef90_A0A1D2VEG0_6_324      TRVLGTNKLELYLNKYGID--LDQETSRLI--S-----------PCNER-
116 UniRef90_A0A0D0B3X6_2_320      MKVLGSDIFDAYLKTYDIP--FETDMEALL--Y-----------SYPRQ-
117 UniRef90_A0A1G4MEF4_15_334     ARVLGTKELLAYLNHYGLK--LPHEYDNIM--K-----------DFERK-
118 UniRef90_A0A2V0NTF2_16_339     AKVLGSEDLYRYADKYGVE--VDPALVTAV--G-----------YRPRQ-
119 UniRef90_A0A4Y9YFI0_2_320      VKVLGTDSFEQYLFTYNLC--LTNVSDELM--Q-----------QHARL-
120 UniRef90_W6KIF7_13_329         VKVLGTDDLRVYLNKFNLK--LPSSLLNLL--T-----------KQTKK-
121 UniRef90_A0A0C3NKK6_2_320      VKVLGTDTFERYLEKYNMQ--VETDHPDLL--R-----------KHARV-
122 UniRef90_A0A0D7AGR9_2_320      LKCLGTERFDLYLQKYCIH--FETEHESLL--S-----------NYPRQ-
123 UniRef90_G0VHQ2_23_343         AKVLGTKQLINYLARYGLH--LPSEHDNLI--R-----------TWARK-
124 UniRef90_UPI000EA961A9_2_209   --------------------------------------------------
125 UniRef90_A0A165QAJ6_2_320      VKILGSDSFENYLYNYNIC--LVNASDDLL--H-----------PYDKQ-
126 UniRef90_A0A6J1ACU4_2_223      --------------------------------------------------
127 UniRef90_A0A146IBX2_2_322      MRVLGTDKFDAYLKAYDIP--FESDVEELL--G-----------SYPAR-
128 UniRef90_A0A1E5RA31_17_339     TQILGTKELLQYLQHYKLP--LPSEYDKIL--K-----------NYERK-
129 UniRef90_J9HLC0_29_343         AQVMGTDDLVEYMKKYKLS--LPSKLSNKL--K-----------RYPQK-
130 UniRef90_A0A183CIH8_805_1008   --------------------------------------------------
131 UniRef90_A0A1M2V5N7_3_298      VKVLGSDDFERYLTTFRLY--LQTYDEELL--Q-----------SYPKI-
132 UniRef90_A0A1B7TD73_22_342     AQIMGTEDLMAYLRKYGLL--LPDEYNGLM--K-----------NFPKK-
133 UniRef90_A0A4P9VZ92_1_208      AKVLGTDELFAYADKYDIE--LDPHFDEVL--G-----------RYPRK-
134 UniRef90_W0T7F4_129_366        VRVLGSDDFEKYLQKYQIT--LPDEFVD-M--D-----------QYIRR-
135 UniRef90_UPI000711954B_2_325   AEVLGTDKLHEYIKKYQLN--VDPIVLSTI--G-----------TCRRR-
136 UniRef90_A0A1C7MA44_2_304      LKVLGTEEFGRYLTTYSLY--LQAYNQELV--Q-----------SYPRQ-
137 UniRef90_C5DWN1_128_366        VRVLGSEDFEKYLLKYEIT--LPSEFHD-M--D-----------QYIRR-
138 UniRef90_A0A7H9B7A0_128_371    VRVLGSDDFEKYLAKYDIV--LPREFHD-M--D-----------QYIRR-
139 UniRef90_W5KQ02_73_295         AKVLGTDELFGYLRKYHIE--LDPRFKDLL--G-----------QQTRK-
140 UniRef90_A0A6P6YBM1_25_347     VEVLGTDEFLKYIKKYGLNTPFDENSKEIL--------------NKPRI-
141 UniRef90_A0A196S5L7_3_325      EEVLGSEDLNRYITKYNLK--LNKETKCLL--G-----------DFEKR-
142 UniRef90_A0A7S3FVA9_17_301     AKVVGTQEVIDYVEKFDLE--IPPKIEEKL--L-----------NFKKK-
143 UniRef90_A2FBX9_18_336         CSVLGGKVLENYLEKYGIP--LPDYLQTSI--F-----------RLKPK-
144 UniRef90_UPI0010FB2831_6_323   MEVLGTEDLFWYLKKYGIA--VPSRLCKEI--P-----------EYPKR-
145 UniRef90_Q8SRU0_6_317          ARILGYTHLKKYVRKYKMA-PLNPKYE-----G-----------VGERV-
146 UniRef90_E9ACB1_46_365         VAVFGTKALYRYLDKYQCR--ISRVVESSM--S-----------ALPDEH
147 UniRef90_W6KPE3_52_367         VAVFGTDAVRRYVAKYNAQ--IPRAVVSNL--E-----------GMAPGH
148 UniRef90_A0A1J4JEQ9_9_332      ADLVGGQGIIEYAESLHVE--LDEETLGQL--R-----------RRKGT-
149 UniRef90_G9N8S2_27_316         ARVLGTKGLLNYVEKYDME--TITDGIDAI--A-----------HFERR-
150 UniRef90_A0A4Z1TD09_8_331      VEILGSAKLFDLLDKYGIK--LPQARAAEL--R-----------GYDGM-


001 Input_pdb_SEQRES_A             -RWERFVHS----------ENQHLV-SPE--ALDFLDKLLRYDHQSRLTA
002 UniRef90_A0A060X538_2_334      -RWERFVHS----------ENQQLI-SPE--GLDFLDKLLRYDHQARLTA
003 UniRef90_UPI000719C52A_2_332   -RWERFVHS----------ENQHLV-SPE--ALDFLDKLLRYDHQERLTA
004 UniRef90_A0A7R8XAU3_2_332      -RWERFVHS----------ENQHLV-SPE--ALDFLDKLLRYDHQERMTA
```

```
005 UniRef90_V3ZFM5_3_333            -RWERFVHS----------ENQHLV-SPE--ALDFLDKLLRYDHQERLTA
006 UniRef90_UPI00193A3309_3_330     -RWERFIHT----------DNQHLV-SPE--ALDFLDNLLRYDHQTRLTA
007 UniRef90_A0A1Q3F4A5_3_329        -RWERFVHS----------ENQHLV-SPE--ALDFLDKLLRYDHYERLTA
008 UniRef90_A0A4W3H6I6_2_321        -RWERFVHS----------ENQHLV-SSE--ALDFLDKLLRYDHQTRLTA
009 UniRef90_UPI000C6E8BEA_2_341     -RWERFVHS----------ENQHLV-SPE--ALDFLDKLLRYDHQERLTA
010 UniRef90_A0A0L0BLN9_116_444      -RWERFVHS----------DNQHLV-SPE--ALDFLDKLLRYDHVERLTA
011 UniRef90_A0A183IEN2_2_329        -RWERFVHS----------ENQHLV-TPE--SLDFLDKLLRYDHNERLTA
012 UniRef90_A0A5N4B3X1_3_332        -RWERFVHS----------ENQQLV-SGE--ALDFLDKLLRYDHMERLTA
013 UniRef90_A0A556VUA8_53_369       -RWERFVHS----------ENQHLI-STE--ALDFLDKLLRYDHQARLTA
014 UniRef90_A0A1I8HE15_2_329        -RWERFVHS----------GNQYLV-SPE--ALDFLDRLLRYDHNDRLTA
015 UniRef90_F6RN85_7_328            -RWENFLHS----------ENRHLV-SAE--ALDLLDKLLRYDHQQRLTA
016 UniRef90_UPI0011E4E1F1_2_326     -RWERFVHQ----------ENQHLV-TPD--ALDFLDKLLRYDHRERMTA
017 UniRef90_A0A0P7V994_7_333        -RWDQFVQL----------ENQHLV-SPE--ALDLLDKLLRYDHQQRLTA
018 UniRef90_A0A3Q4N194_2_309        -RWERFVHS----------ENQHLV-SPE--ALDFLDKLLRYDHQARLTA
019 UniRef90_A0A0N5ANV2_2_328        -RWERFVHS----------ENEHLV-SQE--ALDFLDRLLRYDHQDRLTA
020 UniRef90_A0A0M3HMV3_2_324        -RWERFVHS----------ENQHLV-TPE--AIDFLDKLLRYDHQERLTA
021 UniRef90_A0A2R7W6M3_5_330        -RWERFVHS----------ENQHLV-SPE--ALDFLDKLLRYDHHDRLTA
022 UniRef90_A0A368G0G8_241_571      -RWERFIHA----------ENQHLV-TPE--AIDFLDKLLRYDHQERLTA
023 UniRef90_A0A5F4C0P0_224_555      -RWENFIHS----------ENRHLV-SPE--ALDLLDKLLRYDHQQRLTA
024 UniRef90_A0A671N1V7_3_326        -RWEQFVQT----------ENQHLV-SPE--ALDLLDKLLRYDHQQRLTA
025 UniRef90_A0A667Y761_7_331        -RWEQFVQT----------ENQHLV-SPE--ALDLLDKLLRYDHQQRLTA
026 UniRef90_I0Z462_2_325            -PWTKFVNS----------ENQHLV-SPE--ALDFLDKLLRYDHQERLTA
027 UniRef90_A0A4W5MUS6_7_326        -RWEQFVQT----------DNQHLV-SPE--ALDLLDKLLRYDHQQRLTA
028 UniRef90_UPI000C1CFB1C_2_322     -PWSRFINS----------NNQHLV-SPE--AIDFLDKLLQYDHQDRLTA
029 UniRef90_A0A200QFN8_2_323        -PWSKFINA----------DNQHLV-SPE--AIDFLDKLLRYDHQDRLTA
030 UniRef90_A0A6I9QC06_84_406       -PWSKFINV----------DNQHLA-VPE--AVDFVDKLLQYDHQERLTA
031 UniRef90_A9NVF1_102_427          -PWSKFINA----------DNQHLV-VPE--AVDFLDKLLRYDHQERPTA
032 UniRef90_A0A6S7M9R1_74_394       -PWSKFMNA----------DNQHLV-SPE--AIDFLDKLLRYDHQDRLTA
033 UniRef90_A0A5J4ZXR3_110_433      -PWTKFINA----------DNQHLA-VPE--AIDFLDKLLRYDHQERATA
034 UniRef90_A0A0D2WV07_2_326        -SWDKFVTA----------DNKRFI-TPE--ALDLLDKLLRYDHAQRITA
035 UniRef90_A0A2P6Q0W9_107_428      -PWQRFITP----------DNRHLV-SSE--AIDFLDKLLRYDHQDRLTA
036 UniRef90_A0A2J6LRB0_100_422      -AWSKFINA----------DNQHLA-VPE--AIDFLDKLLRYDHQERPTA
037 UniRef90_A0A4S8ISY9_72_395       -PWTRFINA----------DNQHVA-VPE--AVDFVDKLLRYDHQERPTA
038 UniRef90_A0A1U8LZW6_92_416       -PWTKFINA----------ENQHLA-LPE--AIDFLDKLLRYDHLERLTA
039 UniRef90_K3XBI1_15_338           -PWEKFITQ----------ENKHLV-SPE--ALDFLDGLLRYDHQERLTA
040 UniRef90_E1ZRP6_14_337           -PFTKFVNS----------DNQHLV-SAE--AIDFIDKLLRYDHQERLTA
041 UniRef90_A0A2I0AFK3_70_391       -PWARFVND----------ENQHVA-VPE--AVDFLDKLLRYDHQERLTA
042 UniRef90_UPI000F7CDB20_80_401    -PWEKFINA----------DNQHLA-VPE--AVDFVDKLLQYDHQERPTA
043 UniRef90_G7K3U9_82_403           -PWTKFINM----------DNRHLA-VPE--AIDFLDKLLQYDHQERLTA
044 UniRef90_A0A663MTN9_26_324       -RWENFIHS----------ENRHLV-SPE--VLDLLDKLLRYDHQQRLTA
045 UniRef90_A4S5X1_14_334           -PWNKYVTA----------ENQHLV-STD--AIDFLDKLLRYDHQERITA
046 UniRef90_A0A183LBY6_9_327        -RWERFVHA----------ENQHLV-SPE--SLDLLDKLLRYDHAERLTA
047 UniRef90_A0A1J9RPL1_99_422       -SWHSFINA----------ENQRFV-SND--AIDFLDKLLRYDHQERLTA
048 UniRef90_D5GJ91_2_320            -SWHQFVNA----------DNQRFV-SND--ALDFLDKLLRYDHQERLTA
049 UniRef90_A0A4W4DPG2_3_315        -RWEHFVQP----------ENQHLV-SPE--ALDLLDKLLRYDHQQRLTA
050 UniRef90_A0A7S2RD37_2_324        -PWTKFVHQ----------DNQHLI-STE--ALDFLSKLLRYDHQERLTA
051 UniRef90_A0A367JQL9_12_336       -PWTKFITA----------DNQEFA-SED--ALDFLDKLLRYDHQERLTA
052 UniRef90_UPI001924B863_29_350    -PWSKFINA----------DNQHLV-SPE--AIDLLDKLLRYDHQDRLTA
053 UniRef90_A0A367KWU7_10_331       -PWTKFVTA----------DNQLFA-TDD--ALDFLDKLLRYDHQERLTA
054 UniRef90_A0A0D2AMV0_2_320        -SWHSFITP----------ENQRFV-SSE--AIDFLDKLLRYDHMERLTA
055 UniRef90_A0A1Y1WVY1_2_324        -PWSQFVNA----------DNQKYI-SNE--AIDFLDHLLRYDHQERLTA
056 UniRef90_Q8TG13_2_324            -PWHSFINA----------ENQRFV-SNE--AIDFLDKLLRYDHNERLTA
057 UniRef90_A0A0F8B0Y9_1_322        -PWHSFVTA----------DNQRYV-SNE--AIDFLDKLLRYDHQERLTA
058 UniRef90_A0A7S1NI55_6_324        -QWSYFITK----------ENQHLC-TSD--ALDFLDKLLRYDHQERITA
059 UniRef90_A0A383VX45_13_335       -AWSKFVTP----------DNAHLV-SPE--ALDFLDGLLRYDHQERFTC
060 UniRef90_I1C1X5_16_338           -PWTKFITS----------DNQPFA-TED--ALDFLDKLLRYDHQERLTA
061 UniRef90_A0A364LA26_2_320        -PWHSFVNA----------ENQRFV-SNE--AIDFLDKLLKYDHAERLTA
062 UniRef90_A0A1X7RU70_2_320        -SWHAFVNP----------DNQRFV-SVD--AIDFLDKLLRYDHQERLTA
063 UniRef90_A0A067BWS1_2_324        -PWEKFITP----------ENKHLV-TPE--ALDFLESLLQYDHQKRPTA
064 UniRef90_A0A0P9FCQ6_6_328        -PWSKFITS----------ENQRYI-SNE--AIDFLDHLLRYDHQERATA
065 UniRef90_A0A0K3CS88_10_335       -PWSKFVNA----------DNQRYI-SNE--AIDFLDHLLRYDHNARITA
066 UniRef90_A0A4S4KUV1_8_328        -AWSRFITS----------ENQRYI-SNE--AIDFLDKLLRYDHQERLTA
067 UniRef90_UPI000511B7B1_2_322     -PWSRFINA----------ENQHLV-SPE--AIDFLDKXLRYDHQERLTA
```

```
068 UniRef90_A0A5C3QYQ7_5_326      -PWTRFITS----------ENQRYI-SNE--AIDLLDKLLRYDHQERLTA
069 UniRef90_J4GNM1_5_326          -SWSRFITS----------ENQRYI-SNE--AIDFLDKLLRYDHQERLTA
070 UniRef90_A0A1B6QHX1_78_406     -PWPKFVNA----------RNRHLA-TPE--AIDLVDKLLRYDHQERPTA
071 UniRef90_E3Q428_2_328          -PWHSFVTA----------ENQRFV-SNE--AIDFLDKLLRYDHQERLTA
072 UniRef90_A0A161HHJ2_5_327      -PWSRFVNS----------ENSHLV-SEE--AIDFVDKLLRYDHQDRLTA
073 UniRef90_A0A0C2TUQ3_4_328      -PWTKFITS----------ENQRYI-SNE--AIDLLDKLLRYDHQERLTA
074 UniRef90_UPI000644E1AF_26_344  -PWARFVTS----------DNQSLA-VPE--AIDFLDKLLRYDPAERLTT
075 UniRef90_F2U7H7_13_336         -NWMRFVNP----------ENKHLV-TPD--GIDLLDNLLRYDHQERLTA
076 UniRef90_A0A1E3Q523_2_312      -PWQRFVNA----------DNQKYI-SPE--ALDFLDKLLRYDHQERLTA
077 UniRef90_A0A446UN67_26_313     -PWSKFINA----------DNQHLV-SPE--AIDFLDKLLRYDHQDRLTA
078 UniRef90_A0A1E4RU21_6_330      -PWVRFVNE----------NNQHLV-SDE--FLDLIDKLLRYDHQERLTA
079 UniRef90_A5DCV5_5_327          -PWKRFVNE----------NNQHLV-SDE--FLDFIDKLLRYDHQERLTA
080 UniRef90_L8GRW8_10_332         -PWKKFIKS----------KNNKLA-VPE--AIDLLDKLLRYDHQERLTA
081 UniRef90_A3GID5_8_329          -PWERFIND----------NNQHLL-SKE--FLDFIDQLLRYDHQERLTA
082 UniRef90_A0A2V1AT92_3_322      -PWKRFINE----------NNQHLV-SEE--FLDFIDKLLRYDHQERLTA
083 UniRef90_A0A642UHU8_5_327      -SWDRFVND----------NNQHLV-SDE--FLDFIDKLLRYDHQERLTA
084 UniRef90_UPI001036488A_60_387  -PWSKFIND----------DNQHLV-SPE--AIDFLDKLLRYDHQDRLTA
085 UniRef90_A0A448YKC4_4_323      -PWTRFINS----------TNRDLV-SDD--SIDFIDKILRYDHQERLTA
086 UniRef90_UPI001900E851_4_329   -PWNRFVNP----------NNKDLI-SDE--ALDFVDHILRYDHQERLTA
087 UniRef90_A0A4P9ZDD8_3_324      -PWKRFVNE----------NNRHLV-SDE--LIDFFDKLLRYDHQERLTA
088 UniRef90_UPI00064AE4FC_6_334   -RWQHFISG----------ENKHLV-SPE--ALDLLDNLLQYDPQLRLTA
089 UniRef90_A0A7S3CI51_5_325      -PWSKFINH----------QNEALV-SEE--ALDLLDKMLRYDHAERITP
090 UniRef90_UPI00098E15C8_59_372  -PWTKFINA----------GNQHLA-VPE--AIDFIDKLLRYDHQERLTA
091 UniRef90_A5E6J5_9_319          -PWERFINE----------NNQHLV-SNE--FLDFIDHLLRYDHQERLTA
092 UniRef90_A0A0S4KFI2_14_334     -PWTVFITK----------ENQHLC-PPE--ALDFLDKLLRYDHAERIQA
093 UniRef90_A0A4Y7PMM1_7_330      -QWMSFVTT----------DNQRYI-PND--AIDFLDKLLRYDHNDRLTA
094 UniRef90_A0A674DLQ6_2_238      --------------------------------------------------
095 UniRef90_A0A367YK45_12_333     -PFSRFVTP----------QNQHLV-SEE--ALDLLDKLLRYDHQERLTA
096 UniRef90_Q6BUZ4_8_327          -PWSQFINS----------DNQHLI-SEE--IVDFIDKLLTYDHQLRPTA
097 UniRef90_A0A7S3F0G7_17_281     --------------------------------------------------
098 UniRef90_A0A0C3HJB5_2_320      -NWQTFINP----------ENQRFT-TSE--AIDLLDKLLRYDHQERLTA
099 UniRef90_A0A1R2D4Q4_3_325      -PWHRFITP----------ENQHLV-SPE--VLDLLSAMLKYDHNDRILP
100 UniRef90_UPI0004F4A9E2_17_332  -SWNMFITP----------ENRHLC-PPE--ALNFLDQLLQYDHLKRVQA
101 UniRef90_A0A4P6XGL7_6_324      -PWSSFVNK----------DNKHLV-LEE--VVDLIDKLLTYDHQLRPTA
102 UniRef90_A0A6P6RSI1_108_381    -HWGRFITP----------ENQHLA-TPE--AVDLIDQMLVYDHQMRILP
103 UniRef90_A0A4U0VRL3_43_361     -PWAKFVTS----------DNQHLV-SNA--AIDLIDRLLRYEHTERLTA
104 UniRef90_K0KNM3_9_329          -PWSAFVND----------NNKYLV-DDE--VVDLIDNLLRYDHQERLTA
105 UniRef90_A0A2X0MBX0_6_345      -PWLKYLTSGPLELIPFQIENQRFI-SNE--AIDLLDKLVRYDHQERLTA
106 UniRef90_A0A7S1VNL6_68_384     -PFNRFIAR----------GAESLA-NAD--ALSLLEKLLVYDPAERLTA
107 UniRef90_A0A2H9TP39_34_357     -PWSQFVTP----------ANKHLA-VPE--AIDFLERLLKYDHQERLTA
108 UniRef90_A0A315WAV6_110_366    --------------------------------------------------
109 UniRef90_UPI001386DBE1_151_468 -RWENYIHS----------ENRPLV-SPE--ALDLLDK-----HQQRLTA
110 UniRef90_A0A5D2A803_51_293     --------------------------------------------------
111 UniRef90_A0CQX8_4_329          -QFNKFVNE----------DNEHLA-KAD--AIELLQSMLIYDHNLRITA
112 UniRef90_T1EXU0_70_294         -RWERFVHG----------ENEHLV-SVE--ALDFLDKLLRYDHQERLTA
113 UniRef90_A0A7H9B7U0_15_336     -PWSTFITD----------ETP-LA-VPE--IVDLVDNLLRYDHQERLTA
114 UniRef90_A0A482VYB7_21_342     -PWQRFVNT----------ENEYLV-NHS--AFDFLDSLLCYDHLERLTA
115 UniRef90_A0A1D2VEG0_6_324      -SWQSFIND----------NNRKFT-TNT--VIDLIDNLLRYDHQERLTA
116 UniRef90_A0A0D0B3X6_2_320      -PWARFVNA----------ENQQFV-TSE--ALDLLDKLLRYDHQKRLTA
117 UniRef90_A0A1G4MEF4_15_334     -PWSYFVTS----------DTP-LA-VPE--IIDLIDHLLRYDHQERLTA
118 UniRef90_A0A2V0NTF2_16_339     -PWRKFVTD----------DNRSLV-TPE--ALDLLGKLLTYDHQARPTA
119 UniRef90_A0A4Y9YFI0_2_320      -PWSRFVTA----------ENRTAA-TPE--ALDLLDKLLRYDHRERLTA
120 UniRef90_W6KIF7_13_329         -PWSLFVTP----------ENRHLC-PDT--ALDFLDKLLRYDHTERIQA
121 UniRef90_A0A0C3NKK6_2_320      -PWAKFVTN----------DNRPNV-TPE--AVDLLDRLLRYDHQERLTA
122 UniRef90_A0A0D7AGR9_2_320      -LWTRYINA----------DNQVYA-SPD--ALDLLDKLLRYDHQERLTA
123 UniRef90_G0VHQ2_23_343         -PWSHFISI----------ETP-LA-TPE--AVDLVDNLLRYDHQERLTA
124 UniRef90_UPI000EA961A9_2_209   --------------------------------------------------
125 UniRef90_A0A165QAJ6_2_320      -SWARFVAS----------DNRTTA-TPE--AIDLVDKLLRFDHRERLTA
126 UniRef90_A0A6J1ACU4_2_223      ----------------------------------------------RC--
127 UniRef90_A0A146IBX2_2_322      -PWTRFITQ----------ENQHLA-GPE--ALAVLDRLLRFDPRERYTA
128 UniRef90_A0A1E5RA31_17_339     -PWEKLITD----------RNRRRC-IPE--LVDLVDKLVRYDHQERLTA
129 UniRef90_J9HLC0_29_343         -DLEEFINR----------NNQGLC-SEE--SFDLLRGMLQYDKNLRITP
130 UniRef90_A0A183CIH8_805_1008   --------------------------------------------------
131 UniRef90_A0A1M2V5N7_3_298      -PWSRFVTA----------DNRPNV-STD--GIDLLDKLLRYNHLERLTA
```

```
132 UniRef90_A0A1B7TD73_22_342       -PWSKYISE----------KNQYKC-PPE--ALDLLDNLLVYDHQKRLTA
133 UniRef90_A0A4P9VZ92_1_208        -PWHKFITN----------DNERYV-STE--ALDFLDKLLRYDHQERLTP
134 UniRef90_W0T7F4_129_366          -PWQRFVND----------ANKHLCGNDE--IIDLIDNLLRYDHQERLTC
135 UniRef90_UPI000711954B_2_325     -DWSEFVAS----------GNQNLI-CTE--LFDLLDNLLMYDHGERYTA
136 UniRef90_A0A1C7MA44_2_304        -PWSRFVTS----------DNRLNV-SPD--SIDLLDKLLRYDHQQRLTA
137 UniRef90_C5DWN1_128_366          -PWRRFIND----------SNRHLSDNDE--VIDLLDNLLRYDHAERLTA
138 UniRef90_A0A7H9B7A0_128_371      -PWHRFIND----------SNRHLCDNDD--IIDLLDNLLRYDHQERLTA
139 UniRef90_W5KQ02_73_295           -RWEQFVQT----------ENQHLV-SPE--ALDLLDKLLRYDHQQRLTA
140 UniRef90_A0A6P6YBM1_25_347       -PWKEFIDS----------DDQRFL-SND--AFDLLDKLLVFDHTERITA
141 UniRef90_A0A196S5L7_3_325        -PWSDFINR----------ATEKFI-NDD--LFDFLDRTLVYDHNDRLTA
142 UniRef90_A0A7S3FVA9_17_301       -SWEKFVNN----------QNLNLL-------------------------
143 UniRef90_A2FBX9_18_336           -KWQSFVNY----------DNQSLA-TPD--AIDLIDKCIRFDHTERITA
144 UniRef90_UPI0010FB2831_6_323     -DLKDYLED----------EDYEFL-SEYDLIIDLLGNILVYDHQMRLTA
145 UniRef90_Q8SRU0_6_317            -LLSSFTPP----------GKTGL--YTN--AIDLLEKILIYDHQDRPTA
146 UniRef90_E9ACB1_46_365           VDWRRYIKRGS--------VQESWC-DAT--ALDLLDKMLQFDHQDRIMA
147 UniRef90_W6KPE3_52_367           CGWKRFVKS----------SGSKHC-DDL--ALNLLDKMLQFDHQDRIMA
148 UniRef90_A0A1J4JEQ9_9_332        -GWDRHIAK----------AGKGIC-TPE--AIDLLNRLMAIDHRERLTA
149 UniRef90_G9N8S2_27_316           -SWQSFFNE----------SNERFA-SME--VMDLLDKLLQWDHKQRLTA
150 UniRef90_A0A4Z1TD09_8_331        -SFDVFLNN----------DNQHLV-CEQ--ALDFLKKVLVYDPEERLTV
```

```
001 Input_pdb_SEQRES_A           R E A M - - - E H P Y F Y T V V K D Q A R M G
002 UniRef90_A0A060X538_2_334    R E A M - - - D H P Y F Y P I V K E Q G R M -
003 UniRef90_UPI000719C52A_2_332  R E S M - - - E H P Y F Y P I V K D Q G R M -
004 UniRef90_A0A7R8XAU3_2_332    R E A M - - - E H P Y F F P I V K E Q A R L -
005 UniRef90_V3ZFM5_3_333        R E A M - - - D H P Y F Y A I V Q E Q G R M -
006 UniRef90_UPI00193A3309_3_330 Q E A M - - - D H P Y F F P V V K V Q - - - -
007 UniRef90_A0A1Q3F4A5_3_329    R E A M - - - E H P Y F G I I V N G Q - - - -
008 UniRef90_A0A4W3H6I6_2_321    R E A M - - - E H P Y F Y P I V K D Q S R M G
009 UniRef90_UPI000C6E8BEA_2_341 Q E A M - - - E H S Y L Y P I A K E Q Q - - -
010 UniRef90_A0A0L0BLN9_116_444  R E A M - - - A H P Y F A P I V N G Q M K - -
011 UniRef90_A0A183IEN2_2_329    K E A M - - - E H P Y L C T S - - - - - - - -
012 UniRef90_A0A5N4B3X1_3_332    R D A M - - - A H P Y F Y P V V K D Q S R L -
013 UniRef90_A0A556VUA8_53_369   R E A M - - - E H P Y F Y P I V K D P A R M G
014 UniRef90_A0A1I8HE15_2_329    K E A M - - - D H P Y F V P V V S A Q - - - -
015 UniRef90_F6RN85_7_328        R E A M - - - D H P Y F F T C - - - - - - - -
016 UniRef90_UPI0011E4E1F1_2_326 R E A M - - - Q H P Y F H Q V A - - - - - - -
017 UniRef90_A0A0P7V994_7_333    T E A M - - - Q H P Y F Y P V V K D Q S - - -
018 UniRef90_A0A3Q4N194_2_309    R E A M - - - D H P Y F C - - - - - - - - - -
019 UniRef90_A0A0N5ANV2_2_328    K E A M - - - K H P Y F Y P V L E A - - - - -
020 UniRef90_A0A0M3HMV3_2_324    K E A M - - - Q H P Y F Y P V V E N E Q R - -
021 UniRef90_A0A2R7W6M3_5_330    R E A M - - - E H P Y F Y P V K N E S - - - -
022 UniRef90_A0A368G0G8_241_571  R E A M - - - A H P Y F Y P V V E A A R - - -
023 UniRef90_A0A5F4C0P0_224_555  K E A M - - - E H P Y F Y P V V K E Q S Q - -
024 UniRef90_A0A671N1V7_3_326    T E A M - - - E H P Y F Y P V M K E Q S H - -
025 UniRef90_A0A667Y761_7_331    A E A M - - - Q H P Y F Y P V V K E Q T - - -
026 UniRef90_I0Z462_2_325        K E A M - - - A H P Y F A A V R A Q - - - - -
027 UniRef90_A0A4W5MUS6_7_326    T E A M - - - E H P Y F Y P V L K E Q S - - -
028 UniRef90_UPI000C1CFB1C_2_322 K E A M - - - A H P Y F I Q V - - - - - - - -
029 UniRef90_A0A200QFN8_2_323    K E A M - - - A H P Y F F Q V R - - - - - - -
030 UniRef90_A0A6I9QC06_84_406   K E A M - - - A H P Y F H P V R - - - - - - -
031 UniRef90_A9NVF1_102_427      K E A M - - - A H P Y F Y H V R S A - - - - -
032 UniRef90_A0A6S7M9R1_74_394   K E A M - - - A H P Y F M Q V - - - - - - - -
033 UniRef90_A0A5J4ZXR3_110_433  K E A M - - - A H P Y F Y P V R - - - - - - -
034 UniRef90_A0A0D2WV07_2_326    P E A L - - - Q H P Y F H P V I R A - - - - -
035 UniRef90_A0A2P6Q0W9_107_428  R E A M - - - A H P Y F S E V - - - - - - - -
036 UniRef90_A0A2J6LRB0_100_422  K E A M - - - A H P Y F Y P V R - - - - - - -
037 UniRef90_A0A4S8ISY9_72_395   K E A M - - - A H P Y F N P V R - - - - - - -
038 UniRef90_A0A1U8LZW6_92_416   K E A M - - - A H P Y F Y P I R - - - - - - -
039 UniRef90_K3XBI1_15_338       K E A M - - - Q H A Y F Q P V R D - - - - - -
040 UniRef90_E1ZRP6_14_337       R E A M - - - A H P F L A P V - - - - - - - -
041 UniRef90_A0A2I0AFK3_70_391   K E A M - - - A H P Y F N P V - - - - - - - -
```

```
042 UniRef90_UPI000F7CDB20_80_401 KEAM---AHPYFNPV--------
043 UniRef90_G7K3U9_82_403 KEAM---AHPYFSPV--------
044 UniRef90_A0A663MTN9_26_324 KEAM---EHPYFYPVVKEQSQ--
045 UniRef90_A4S5X1_14_334 KEAQ---SHPYFD----------
046 UniRef90_A0A183LBY6_9_327 REAM---EHVYFR----------
047 UniRef90_A0A1J9RPL1_99_422 KEAM---AHPYFNPV--------
048 UniRef90_D5GJ91_2_320 KEAM---AHPYFEPV--------
049 UniRef90_A0A4W4DPG2_3_315 QEAM---EHPYFYPVMNGQS---
050 UniRef90_A0A7S2RD37_2_324 KEAM---AHDYFKPVYA------
051 UniRef90_A0A367JQL9_12_336 KEAM---GHHYFDAVRQNQ----
052 UniRef90_UPI001924B863_29_350 KEAV---AHPYFSQV--------
053 UniRef90_A0A367KWU7_10_331 KEAM---EHPYFDVVR-------
054 UniRef90_A0A0D2AMV0_2_320 KEAM---AHPYFAPV--------
055 UniRef90_A0A1Y1WVY1_2_324 QEAM---NHAYFDPVRK------
056 UniRef90_Q8TG13_2_324 KEAM---AHPYFAPVRDEA----
057 UniRef90_A0A0F8B0Y9_1_322 KEAQ---AHVYFDP---------
058 UniRef90_A0A7S1NI55_6_324 KEAM---EHLYFVPITK------
059 UniRef90_A0A383VX45_13_335 KEAM---AHPYFAPV--------
060 UniRef90_I1C1X5_16_338 KEAM---VHHYFDAVRQNQ----
061 UniRef90_A0A364LA26_2_320 QEAM---AHPYFDPV--------
062 UniRef90_A0A1X7RU70_2_320 KEAM---AHQYFAPI--------
063 UniRef90_A0A067BWS1_2_324 KEAM---AHPFFQVIRD------
064 UniRef90_A0A0P9FCQ6_6_328 KELLDSPGHAYFNP---------
065 UniRef90_A0A0K3CS88_10_335 KEGQ---SHALFAPVREQER---
066 UniRef90_A0A4S4KUV1_8_328 REAQ---THPYFVAL--------
067 UniRef90_UPI000511B7B1_2_322 REAM---AHAYFSQV--------
068 UniRef90_A0A5C3QYQ7_5_326 REAQ---GQPYFGRV--------
069 UniRef90_J4GNM1_5_326 REAQ---AHPYFDPV--------
070 UniRef90_A0A1B6QHX1_78_406 KETM---AHPYFNPVRSSE----
071 UniRef90_E3Q428_2_328 KEAQ---AHPYFNPV--------
072 UniRef90_A0A161HHJ2_5_327 KEAM---AHAYFEPV--------
073 UniRef90_A0A0C2TUQ3_4_328 REAQ---AQSYFDPV--------
074 UniRef90_UPI000644E1AF_26_344 KEAM---EHPYFK----------
075 UniRef90_F2U7H7_13_336 TEAM---AHKFFDSVR-------
076 UniRef90_A0A1E3Q523_2_312 KEAM---EHDYFAPVRKE-----
077 UniRef90_A0A446UN67_26_313 REAM---LLS-------------
078 UniRef90_A0A1E4RU21_6_330 QEAM---NHPYFYPITKN-----
079 UniRef90_A5DCV5_5_327 KEAM---AHPYFDPV--------
080 UniRef90_L8GRW8_10_332 IEAM---EHPYFAPI--------
081 UniRef90_A3GID5_8_329 KEAM---DHPYFDPV--------
082 UniRef90_A0A2V1AT92_3_322 KEAM---DHPWFD----------
083 UniRef90_A0A642UHU8_5_327 QEAM---DHAYFDPVR-------
084 UniRef90_UPI001036488A_60_387 KEAM---AHPYFSQVR-------
085 UniRef90_A0A448YKC4_4_323 QECM---MHPYFAPI--------
086 UniRef90_UPI001900E851_4_329 KECM---MHSYFAPIREEQKK--
087 UniRef90_A0A4P9ZDD8_3_324 QEAM---AHPYFDPV--------
088 UniRef90_UPI00064AE4FC_6_334 REAM---AHPYLSPFVKEL----
089 UniRef90_A0A7S3CI51_5_325 KDAM---DHPYFKPVK-------
090 UniRef90_UPI00098E15C8_59_372 KEAM---AHPYFNP---------
091 UniRef90_A5E6J5_9_319 KEAM---EHPYFDP---------
092 UniRef90_A0A0S4KFI2_14_334 QEAM---QHPYFAPIRKMQ----
093 UniRef90_A0A4Y7PMM1_7_330 KEAM---SHKYFVPV--------
094 UniRef90_A0A674DLQ6_2_238 -----------------------
095 UniRef90_A0A367YK45_12_333 KEAM---AHPYFDPV--------
096 UniRef90_Q6BUZ4_8_327 KEAM---DHPFFT----------
097 UniRef90_A0A7S3F0G7_17_281 -----------------------
098 UniRef90_A0A0C3HJB5_2_320 KEAM---AHPFFEPVR-------
099 UniRef90_A0A1R2D4Q4_3_325 HEAM---KHIYFAPVVQ------
100 UniRef90_UPI0004F4A9E2_17_332 LEAM---QHPYFDPV--------
101 UniRef90_A0A4P6XGL7_6_324 KETL---AHPFF-----------
102 UniRef90_A0A6P6RSI1_108_381 RDAM---LHPYFRPVVEEEQR--
103 UniRef90_A0A4U0VRL3_43_361 AEAM---QHEYFAPIR-------
104 UniRef90_K0KNM3_9_329 KEAM---AHPFFK----------
105 UniRef90_A0A2X0MBX0_6_345 KEAM---QHPYFAAVAQADQE--
```

```
106 UniRef90_A0A7S1VNL6_68_384      T E A M - - - A H P Y F D - - - - - - - - - -
107 UniRef90_A0A2H9TP39_34_357      T E A M - - - A H P Y F D S V R K - - - - - -
108 UniRef90_A0A315WAV6_110_366     - - - - - - - - - - - - - - - - - - - - - - -
109 UniRef90_UPI001386DBE1_151_468  K E A T - - - E H P V Y V A M K E - - - - - -
110 UniRef90_A0A5D2A803_51_293      - - - - - - - - - - - - - - - - - - - - - - -
111 UniRef90_A0CQX8_4_329           K E A M - - - L H P Y F Q S I R K N S K R - -
112 UniRef90_T1EXU0_70_294          K E S M - - - D H P Y F D M I L K G - - - - -
113 UniRef90_A0A7H9B7U0_15_336      K E A I - - - E H P Y F K T - - - - - - - - -
114 UniRef90_A0A482VYB7_21_342      K E A L - - - E H K Y F D L - - - - - - - - -
115 UniRef90_A0A1D2VEG0_6_324       K E A M - - - E H P F F E - - - - - - - - - -
116 UniRef90_A0A0D0B3X6_2_320       R E A Q - - - A H P Y F N L V - - - - - - - -
117 UniRef90_A0A1G4MEF4_15_334      K E T M - - - A H E F F - - - - - - - - - - -
118 UniRef90_A0A2V0NTF2_16_339      A E A M - - - A H P Y F E P I R - - - - - - -
119 UniRef90_A0A4Y9YFI0_2_320       A E A M - - - A H T Y F N V V - - - - - - - -
120 UniRef90_W6KIF7_13_329          A E A L - - - Q H P Y F D P V - - - - - - - -
121 UniRef90_A0A0C3NKK6_2_320       A E A L - - - S H T Y F N S V - - - - - - - -
122 UniRef90_A0A0D7AGR9_2_320       R E C Q - - - A H V Y F N S V - - - - - - - -
123 UniRef90_G0VHQ2_23_343          E E A M - - - N H Q F F Q - - - - - - - - - -
124 UniRef90_UPI000EA961A9_2_209    - - - - - - - - - - - - - - - - - - - - - - -
125 UniRef90_A0A165QAJ6_2_320       A E A M - - - A H T Y F N V V - - - - - - - -
126 UniRef90_A0A6J1ACU4_2_223       - - - - - - - - - - - - - - - - - - - - - - -
127 UniRef90_A0A146IBX2_2_322       K E A M - - - A N A Y F S T V R I - - - - - -
128 UniRef90_A0A1E5RA31_17_339      K E A L - - - D H E F F H - - - - - - - - - -
129 UniRef90_J9HLC0_29_343          T D A M - - - K H K Y F D P V - - - - - - - -
130 UniRef90_A0A183CIH8_805_1008    - - - - - - - - - - - - - - - - - - - - - - -
131 UniRef90_A0A1M2V5N7_3_298       A E A L - - - A H S F F M M - - - - - - - - -
132 UniRef90_A0A1B7TD73_22_342      E E A L - - - A H P F F - - - - - - - - - - -
133 UniRef90_A0A4P9VZ92_1_208       Q E A M - - - A H P Y F D P V - - - - - - - -
134 UniRef90_W0T7F4_129_366         K E A M - - - A H P W F A P I - - - - - - - -
135 UniRef90_UPI000711954B_2_325    Q E C M - - - K H P Y F D P V R D E - - - - -
136 UniRef90_A0A1C7MA44_2_304       A E A Q - - - A H S F F N I V - - - - - - - -
137 UniRef90_C5DWN1_128_366         R E A M - - - G H P W F E P I - - - - - - - -
138 UniRef90_A0A7H9B7A0_128_371     R E A M - - - G H P W F E P I R K G E S - - -
139 UniRef90_W5KQ02_73_295          T E A M - - - E H P Y F Y P V L K E Q S - - -
140 UniRef90_A0A6P6YBM1_25_347      K D A L - - - Y H P Y F D E V - - - - - - - -
141 UniRef90_A0A196S5L7_3_325       K E A M - - - A H K W F D P V R D D - - - - -
142 UniRef90_A0A7S3FVA9_17_301      - - - - - - - - - - - - - - - - - - - - - - -
143 UniRef90_A2FBX9_18_336          A E A L - - - A H P Y F D S V - - - - - - - -
144 UniRef90_UPI0010FB2831_6_323    R E C L - - - D H P F F - - - - - - - - - - -
145 UniRef90_Q8SRU0_6_317           D E C L - - - R H P L F E - - - - - - - - - -
146 UniRef90_E9ACB1_46_365          H E A M - - - Q H P F F A P V R - - - - - - -
147 UniRef90_W6KPE3_52_367          H D A M - - - T H P Y F E P V - - - - - - - -
148 UniRef90_A0A1J4JEQ9_9_332       K E A L - - - K H P Y F R P L V K - - - - - -
149 UniRef90_G9N8S2_27_316          G D A L - - - K H A Y F - - - - - - - - - - -
150 UniRef90_A0A4Z1TD09_8_331       R E A I - - - A H P Y F D P I R E Q - - - - -
```

1	2	3	4	5	6	7	8	9
Variable				Average				Conserved

X - Insufficient data - the calculation for this site was performed on less than 10% of the sequences.
