# Supplementary material for: Predictive functional, statistical and structural analysis of CSNK2A1 and CSNK2B variants linked to neurodevelopmental diseases
Source: Front Mol Biosci. 2022 Oct 13;9:851547. doi: 10.3389/fmolb.2022.851547 (PMC9608649; doi:10.3389/fmolb.2022.851547)
Supplement: Supplementary file 8 [file DataSheet1.PDF]

Alignment showing conservation in CK2α mutated residues across eukaryotic species. Alignment was performed using MUSCLE (HTML format). Residues in Blue are mostly conserved across species, somehow conserved in gray and not conserved in white. The human sequence is bolded and the residues mutated are marked in red.

```
MUSCLE alignment. HTML format

gi|50311589|ref|XP_455820.1|       -------------------------------MStl-------------------------
gi|6324635|ref|NP_014704.1|        -------------------------------MplPPS-----------------------
gi|45188077|ref|NP_984300.1|       -------------------------------MSlPPS-----------------------
gi|19114554|ref|NP_593642.1|       ---------------------------MNqTeaaP-------------------------
gi|17505290|ref|NP_492811.1|       --------------------------------mpP-------------------------
gi|326671531|ref|XP_003199454.1|   -------------------------------MSGP-------------------------
gi|24668866|ref|NP_730775.1|       ---------------------------------mt-------------------------
gi|158294401|ref|XP_315576.4|      ---------------------------------mt-------------------------
gi|410054859|ref|XP_003953729.1|   -------------------------------MSGP-------------------------
XP_018092886.1                     -------------------------------MSGP-------------------------
XP_031750688.1                     -------------------------------MSGP-------------------------
gi|50355952|ref|NP_001002242.1|    -------------------------------MSGP-------------------------
gi|29570791|ref|NP_808227.1|       -------------------------------MSGP-------------------------
gi|297260098|ref|XP_002798227.1|   -------------------------------MSGP-------------------------
gi|545540258|ref|XP_005634959.1|   -------------------------------MSGP-------------------------
gi|88319941|ref|NP_777060.2|       -------------------------------MSGP-------------------------
gi|31542427|ref|NP_031814.2|       -------------------------------MSGP-------------------------
gi|16758674|ref|NP_446276.1|       -------------------------------MSGP-------------------------
gi|15227778|ref|NP_179889.1|       malrpctgftisslrnasaannnlfslLSfSsSsPakrnlllsslqdnlrrfassaslyr
gi|389637090|ref|XP_003716185.1|   ------------------------------------------------------------
gi|164427285|ref|XP_964983.2|      ------------------------------------------------------------
                                                                  M1

gi|50311589|ref|XP_455820.1|       --------------------------QpsaLNkKSkRVySvARVYqDacekRPqEYWDYE
gi|6324635|ref|NP_014704.1|        -----------------------------tLNqKSnRVySvARVYknaceeRPqEYWDYE
gi|45188077|ref|NP_984300.1|       -----------------------------tLNqKSaRVyStARVYkDacaqRsqdYWDYE
gi|19114554|ref|NP_593642.1|       -------------------------------------VvSvsRVYahVNeemPREYWDYE
gi|17505290|ref|NP_492811.1|       -------------------------------------IPSRARVYaeVNpsRPREYWDYE
gi|326671531|ref|XP_003199454.1|   -------------------------------------VkSRARVYTeVNTHRPkEYWDYq
gi|24668866|ref|NP_730775.1|       -------------------------------------lPSaARVYTDVNaHkPdEYWDYE
gi|158294401|ref|XP_315576.4|      -------------------------------------lPSsARVYaDVNsHkPREYWDYE
gi|410054859|ref|XP_003953729.1|   -------------------------------------VPSRARVYTDVNTHRPREYWDYE
XP_018092886.1                     -------------------------------------VPSRARVYTDVNTHRPREYWDYE
XP_031750688.1                     -------------------------------------VPSRARVYTDVNTHRPREYWDYE
gi|50355952|ref|NP_001002242.1|    -------------------------------------VPSRARVYTDVNTHRPREYWDYE
gi|29570791|ref|NP_808227.1|       -------------------------------------VPSRARVYTDVNTHRPREYWDYE
gi|297260098|ref|XP_002798227.1|   -------------------------------------VPSRARVYTDVNTHRPREYWDYE
gi|545540258|ref|XP_005634959.1|   -------------------------------------VPSRARVYTDVNTHRPREYWDYE
gi|88319941|ref|NP_777060.2|       -------------------------------------VPSRARVYTDVNTHRPREYWDYE
gi|31542427|ref|NP_031814.2|       -------------------------------------VPSRARVYTDVNTHRPREYWDYE
gi|16758674|ref|NP_446276.1|       -------------------------------------VPSRARVYTDVNTHRPREYWDYE
gi|15227778|ref|NP_179889.1|       qhlrnqqqqhqqqqqsrvkeksetlaQkigksirragaPSKARVYaDVNvvRPkdYWDYE
gi|389637090|ref|XP_003716185.1|   -------------------------------------mhSmARVYaDVNanmPRsYWeYd
gi|164427285|ref|XP_964983.2|      ----------------------------------------mARVYaDVNqnmPRaYWDYd
                                                                                  R21    E27

gi|50311589|ref|XP_455820.1|       qcVmIDWGkisnYevikKiGRGKYSEVFkgksVlNdipcViKvLKPVKmKKIyRElKvLt
gi|6324635|ref|NP_014704.1|        qgVTIDWGkisnYeiinKiGRGKYSEVFsgrcIvNNqKcViKvLKPVKmKKIyRElKILt
gi|45188077|ref|NP_984300.1|       qgITInWGkinnYeiinKiGRGKYSEVFrgksIvNdhpcViKvLKPVKmKKIyRElKILt
gi|19114554|ref|NP_593642.1|       nmq-evfGyQDnYeiiRKvGRGKYSEVFEgLNVlNNsKciiKvLKPVKyKKIKREIKILq
gi|17505290|ref|NP_492811.1|       aHm-IEWGqiDDYQLVRKLGRGKYSEVFEgfkmstdEKVVVKILKPVKKKKIKREIKILE
gi|326671531|ref|XP_003199454.1|   SHk-pQWGNheDfQmVtKLGiGKYSdVFEAINITNNEKVVVKtLKPVKrKKIlREvKILE
gi|24668866|ref|NP_730775.1|       nYV-VDWGNQDDYQLVRKLGRGKYSEVFEAINITttEKcVVKILKPVKKKKIKREIKILE
gi|158294401|ref|XP_315576.4|      nYI-VDWvNQDDYQLVRKLGRGKYSEVFEAIkmTsNEKcVVKILKPVKKKKIKREIKILE
gi|410054859|ref|XP_003953729.1|   SHV-VEWGNQDDYQLVRKLGRGKYSEVFEAINITNNEKVVVKILKPVKKKKIKREIKILE
XP_018092886.1                     SHV-VEWGNQDDYQLVRKLGRGKYSEVFEAINITNNEKVVVKILKPVKKKKIKREIKILE
XP_031750688.1                     SHV-VEWGNQDDYQLVRKLGRGKYSEVFEAINITNNEKVVVKILKPVKKKKIKREIKILE
gi|50355952|ref|NP_001002242.1|    SHV-VEWGNQDDYQLVRKLGRGKYSEVFEAINITNNEKVVVKILKPVKKKKIKREIKILE
gi|29570791|ref|NP_808227.1|       SHV-VEWGNQDDYQLVRKLGRGKYSEVFEAINITNNEKVVVKILKPVKKKKIKREIKILE
gi|297260098|ref|XP_002798227.1|   SHV-VEWGNQDDYQLVRKLGRGKYSEVFEAINITNNEKVVVKILKPVKKKKIKREIKILE
gi|545540258|ref|XP_005634959.1|   SHV-VEWGNQDDYQLVRKLGRGKYSEVFEAINITNNEKVVVKILKPVKKKKIKREIKILE
gi|88319941|ref|NP_777060.2|       SHV-VEWGNQDDYQLVRKLGRGKYSEVFEAINITNNEKVVVKILKPVKKKKIKREIKILE
gi|31542427|ref|NP_031814.2|       SHV-VEWGNQDDYQLVRKLGRGKYSEVFEAINITNNEKVVVKILKPVKKKKIKREIKILE
gi|16758674|ref|NP_446276.1|       SHV-VEWGNQDDYQLVRKLGRGKYSEVFEAINITNNEKVVVKILKPVKKKKIKREIKILE
gi|15227778|ref|NP_179889.1|       Sla-VQWGvQDDYevVRKvGRGKYSEVFEgIhaTdNEKcViKILKPVKKKKIKREIKILq
gi|389637090|ref|XP_003716185.1|   tvn-IsWGvlenYevVRKiGRGKYSEVFEgINVvNyqKcViKvLKPVKKKKIKREIKILq
gi|164427285|ref|XP_964983.2|      Svn-IsWGvlenYevVRKiGRGKYSEVFEgINVvNyqKcViKvLKPVKKKKIKREIKILq
                                      E32 Q36 Y39  G46-R47  Y50-S51-E52-V53        V73    R80

gi|50311589|ref|XP_455820.1|       NLtGGPNIIgLlDIVqDPgSkiPALiFEeVkNvDFrtLYpsfTlsDtqFYftqlLtALDY
gi|6324635|ref|NP_014704.1|        NLtGGPNvvgLyDIVqDadSkiPALiFEeikNvDFrtLYpTfklpDIqyYftqlLiALDY
gi|45188077|ref|NP_984300.1|       NLtGGPNIIgLlDIVqDPgSkiPALiFEeVkNveFrtLYpafTlsDtqhYfkqlLiALDY
```

```
gi|19114554|ref|NP_593642.1|        NLaGGPNIIsLlDIVrDPeSkTPsLiFEfVdNiDFrtLYpTLsDYDIRyYsYElLKALDf
gi|17505290|ref|NP_492811.1|        NLRGGtNIITLlDvVKDPISRTPALiFEHVNNsDFKQLYQTLsDYDIRyYLYElLKALDf
gi|326671531|ref|XP_003199454.1|    NLRGGPNIIsLiDmVKDPViRTPALVFEHVNNTDFKQLYQsLTDYDIRFYMFEILKALDf
gi|24668866|ref|NP_730775.1|        NLRGGtNIITLlavVKDPVSRTPALiFEHVNNTDFKQLYQTLTDYeIRyYLFElLKALDY
gi|158294401|ref|XP_315576.4|       NLRGGtNIITLlavVKDPVSRTPALiFEHVNNTDFKQLYQTLsDYDIRyYLYElLKALDY
gi|410054859|ref|XP_003953729.1|    NLRGGPNIITLADIVKDPVSRTPALVFEHVNNTDFKQLYQTLTDYDIRFYMYEILKALDY
XP_018092886.1                      NLRGGPNIITLADIVKDPVSRTPALVFEHVNNTDFKQLYQTLTDYDIRFYMYEILKALDY
XP_031750688.1                      NLRGGPNIITLADIVKDPVSRTPALVFEHVNNTDFK-LYQTLTDYDIRFYMYEILKALDY
gi|50355952|ref|NP_001002242.1|     NLRGGPNIITLADIVKDPVSRTPALVFEHVNNTDFKQLYQTLTDYDIRFYMYEILKALDY
gi|29570791|ref|NP_808227.1|        NLRGGPNIITLADIVKDPVSRTPALVFEHVNNTDFKQLYQTLTDYDIRFYMYEILKALDY
gi|297260098|ref|XP_002798227.1|    NLRGGPNIITLADIVKDPVSRTPALVFEHVNNTDFKQLYQTLTDYDIRFYMYEILKALDY
gi|545540258|ref|XP_005634959.1|    NLRGGPNIITLADIVKDPVSRTPALVFEHVNNTDFKQLYQTLTDYDIRFYMYEILKALDY
gi|88319941|ref|NP_777060.2|        NLRGGPNIITLADIVKDPVSRTPALVFEHVNNTDFKQLYQTLTDYDIRFYMYEILKALDY
gi|31542427|ref|NP_031814.2|        NLRGGPNIITLADIVKDPVSRTPALVFEHVNNTDFKQLYQTLTDYDIRFYMYEILKALDY
gi|16758674|ref|NP_446276.1|        NLRGGPNIITLADIVKDPVSRTPALVFEHVNNTDFKQLYQTLTDYDIRFYMYEILKALDY
gi|15227778|ref|NP_179889.1|        NLcGGPNIvkLlDIVrDqqSkTPsLiFEHVNNkDFKvLYpTLsDYDVRyYiFElLKALDf
gi|389637090|ref|XP_003716185.1|    NLaGGPNvvaLlDvVrDsqSkTPsLiFEyVNNTDFrtLYpkfnDiDVRyYilElLKALDf
gi|164427285|ref|XP_964983.2|       NLaGGPNIvaLlDvVrDsqSkTPsLiFEyVNNTeFrtLYprfnDfDVRyYiFElLKALDf
                                                      R107            Q126-T127

gi|50311589|ref|XP_455820.1|        CHSMGIMHRDVKPqNVMIDpneRKLRLIDWGLAEFYHPGvdYNiRVASRYhKGPELLVsl
gi|6324635|ref|NP_014704.1|         CHSMGIMHRDVKPqNVMIDpteRKLRLIDWGLAEFYHPGvdYNVRVASRYhKGPELLVnl
gi|45188077|ref|NP_984300.1|        CHSMGIMHRDVKPqNVMIDpteRKLRLIDWGLAEFYHPGvdYNVRVASRYhKGPELLVnl
gi|19114554|ref|NP_593642.1|        CHSrGIMHRDVKPHNVMIDHkkRKLRLIDWGLAEFYHaGmEYNVRVASRYFKGPELLVDf
gi|17505290|ref|NP_492811.1|        CHSqGIMHRDVKPHNVMIDaEkReLRLIDWGLAEFYHPrQdYNVRVASRYFKGPELLVDY
gi|326671531|ref|XP_003199454.1|    sHSMGIMHRDVKPHNiMIDHEHRKLRLIDWGLAEFYHPGQEYNVRVASRYFKGPELLiDY
gi|24668866|ref|NP_730775.1|        CHSMGIMHRDVKPHNVMIDHEnRKLRLIDWGLAEFYHPGQEYNVRVASRYFKGPELLVDY
gi|158294401|ref|XP_315576.4|       CHSlGIMHRDVKPHNVMIDHEnRKLRLIDWGLAEFYHPGQEYNVRVASRYFKGPELLVDY
gi|410054859|ref|XP_003953729.1|    CHSMGIMHRDVKPHNVMIDHEHRKLRLIDWGLAEFYHPGQEYNVRVASRYFKGPELLVDY
XP_018092886.1                      CHSMGIMHRDVKPHNVMIDHEHRKLRLIDWGLAEFYHPGQEYNVRVASRYFKGPELLVDY
XP_031750688.1                      CHSMGIMHRDVKPHNVMIDHEHRKLRLIDWGLAEFYHPGQEYNVRVASRYFKGPELLVDY
gi|50355952|ref|NP_001002242.1|     CHSMGIMHRDVKPHNVMIDHEHRKLRLIDWGLAEFYHPGQEYNVRVASRYFKGPELLVDY
gi|29570791|ref|NP_808227.1|        CHSMGIMHRDVKPHNVMIDHEHRKLRLIDWGLAEFYHPGQEYNVRVASRYFKGPELLVDY
gi|297260098|ref|XP_002798227.1|    CHSMGIMHRDVKPHNVMIDHEHRKLRLIDWGLAEFYHPGQEYNVRVASRYFKGPELLVDY
gi|545540258|ref|XP_005634959.1|    CHSMGIMHRDVKPHNVMIDHEHRKLRLIDWGLAEFYHPGQEYNVRVASRYFKGPELLVDY
gi|88319941|ref|NP_777060.2|        CHSMGIMHRDVKPHNVMIDHEHRKLRLIDWGLAEFYHPGQEYNVRVASRYFKGPELLVDY
gi|31542427|ref|NP_031814.2|        CHSMGIMHRDVKPHNVMIDHEHRKLRLIDWGLAEFYHPGQEYNVRVASRYFKGPELLVDY
gi|16758674|ref|NP_446276.1|        CHSMGIMHRDVKPHNVMIDHEHRKLRLIDWGLAEFYHPGQEYNVRVASRYFKGPELLVDY
gi|15227778|ref|NP_179889.1|        CHSrGIMHRDVKPHNVMIDHEqRKLRLIDWGLAEFYHPGkEYNVRVASRYFKGPELLVDl
gi|389637090|ref|XP_003716185.1|    CHSkGIMHRDVKPHNVMIDHEnRKLRLIDWGLAEFYHPGtEYNVRVASRYFKGPELLVDY
gi|164427285|ref|XP_964983.2|       CHSkGIMHRDVKPHN---------LRLIDWGLAEFYHPGtEYNVRVASRYFKGPELLVDf
                                C147 M153 D156 K158 H160-N161 I174-D175 G177-L178 R191 S194-R195 F197-K198-G199

gi|50311589|ref|XP_455820.1|        nqYDYSLDlWavGCMiAaivFkKEPFFkGstNaDQLVkIAKVLGTqeLfyYlkhYgldLp
gi|6324635|ref|NP_014704.1|         nqYDYSLDlWSvGCMLAaivFkKEPFFkGssNpDQLVkIAtVLGTkeLlgYlgKYglhLp
gi|45188077|ref|NP_984300.1|        nqYDYSLDlWSvGCMLAaivFkKEPFFkGstNpDQLVkIArVLGTkqLlaYlehYgltLp
gi|19114554|ref|NP_593642.1|        reYDYSLDiWSfGvMfAalIFkKdtFFrGrDNYDQLVkIAKVLGTdeLfaYVqKYqIvLD
gi|17505290|ref|NP_492811.1|        QcYDYSLDMWSLGCMLASMIFRKEPFFHGHDNYDQLVRIAKVLGTdeLYEYIarYhIdLD
gi|326671531|ref|XP_003199454.1|    QMYDYSLDMWSLGCMLASMIFRKEPFFHGrDNYDQLVRIAKVLGTdgLYDYIDKYNIELD
gi|24668866|ref|NP_730775.1|        QMYDYSLDMWSLGCMLASMIFRKEPFFHGHDNYDQLVRIAKVLGTEeLYaYlDKYNIdLD
gi|158294401|ref|XP_315576.4|       QMYDYSLDMWSLGCMLASMIFRKEPFFHGHDNYDQLVRIAKVLGTEDLfaYlDKYNIELD
gi|410054859|ref|XP_003953729.1|    QMYDYSLDMWSLGCMLASMIFRKEPFFHGHDNxDQmVgIAKfLGTEDLYDYIDKYNIELD
XP_018092886.1                      QMYDYSLDMWSLGCMLASMIFRKEPFFHGHDNYDQLVRIAKVLGTEDLYDYIDKYNIELD
XP_031750688.1                      QMYDYSLDMWSLGCMLASMIFRKEPFFHGHDNYDQLVRIAKVLGTEDLYDYIDKYNIELD
gi|50355952|ref|NP_001002242.1|     QMYDYSLDMWSLGCMLASMIFRKEPFFHGHDNYDQLVRIAKVLGTEDLYDYIDKYNIELD
gi|29570791|ref|NP_808227.1|        QMYDYSLDMWSLGCMLASMIFRKEPFFHGHDNYDQLVRIAKVLGTEDLYDYIDKYNIELD
gi|297260098|ref|XP_002798227.1|    QMYDYSLDMWSLGCMLASMIFRKEPFFHGHDNYDQLVRIAKVLGTEDLYDYIDKYNIELD
gi|545540258|ref|XP_005634959.1|    QMYDYSLDMWSLGCMLASMIFRKEPFFHGHDNYDQLVRIAKVLGTEDLYDYIDKYNIELD
gi|88319941|ref|NP_777060.2|        QMYDYSLDMWSLGCMLASMIFRKEPFFHGHDNYDQLVRIAKVLGTEDLYDYIDKYNIELD
gi|31542427|ref|NP_031814.2|        QMYDYSLDMWSLGCMLASMIFRKEPFFHGHDNYDQLVRIAKVLGTEDLYDYIDKYNIELD
gi|16758674|ref|NP_446276.1|        QMYDYSLDMWSLGCMLASMIFRKEPFFHGHDNYDQLVRIAKVLGTEDLYDYIDKYNIELD
gi|15227778|ref|NP_179889.1|        QdYDYSLDlWSLGCMfAgMIFRKEPFFyGHDNYDQLVkIAKVLGTdeLnaYlnKYrIELD
gi|389637090|ref|XP_003716185.1|    QeYDYSLDMWSLGaMfASMIFRKEPFFHGnsNsDQLVkIAKVLGTdDLfDYlDKYeIELD
gi|164427285|ref|XP_964983.2|       QeYDYSLDMWSLGaMfASMIFRKEPFFHGqsNsDQLVkIAKVLGTdeLfDYlDKYeIELD
                                       D210                P231                       Y261

gi|50311589|ref|XP_455820.1|        aeyNDImknyeRKpWsyFan-dktpLavdEiiDliDhLLRYDHQERLTAkEAMEHkfFkk
gi|6324635|ref|NP_014704.1|         seydnImrdftkKsWthFitSEtk-LavPEvvDliDnLLRYDHQERLTAkEAMDHkfFkt
gi|45188077|ref|NP_984300.1|        heydnImkdfeRKpWsyFisddNt-LavPEvvDliDhLLRYDHQERLTAkEAMDHdfFkk
gi|19114554|ref|NP_593642.1|        rqydnILGqypkrdWysFVnrdNrsLandEAiDlLnrLLRYDHQERLTcqEAMaHPYFqv
gi|17505290|ref|NP_492811.1|        PRFNDILGRHSRKRWERFiHaENQHLVtPEALDFLDKLLRYDHaERLTAqEAMgHeYFrP
gi|326671531|ref|XP_003199454.1|    PRFieILGRHSRKRWkKFVHnENQHLISPEALDFLDKLLRYDHQtRLTAqEAMEHqYFFP
gi|24668866|ref|NP_730775.1|        PRFhDILqRHSRKRWERFVHSdNQHLVSPEALDFLDKLLRYDHvDRLTAREAMaHPYFlP
gi|158294401|ref|XP_315576.4|       PRFNDILsRHSRKRWERFVHSENQHLVSPEgLDFLDKLLRYDHfERLTAREAMEHPYFai
gi|410054859|ref|XP_003953729.1|    PRFNDxLGRHSRKRWERFVHSENQHLVSPEALDFLDKLLRYDHQsRLTAREAMEHPYFc-
XP_018092886.1                      PRFNDILGRHSRKRWERFVHSENQHLVSPEALDFLDKLLRYDHQtRLTAREAMDHPYFYP
XP_031750688.1                      PRFNDILGRHSRKRWERFVHSENQHLVSPEALDFLDKLLRYDHQtRLTAREAMDHPYFYP
gi|50355952|ref|NP_001002242.1|     PRFNDILGRHSRKRWERFVHSENQHLVSPEALDFLDKLLRYDHQsRLTAREAMEHPYFYP
gi|29570791|ref|NP_808227.1|        PRFNDILGRHSRKRWERFVHSENQHLVSPEALDFLDKLLRYDHQsRLTAREAMEHPYFYt
gi|297260098|ref|XP_002798227.1|    PRFNDILGRHSRKRWERFVHSENQHLVSPEALDFLDKLLRYDHQsRLTAREAMEHPYFYt
gi|545540258|ref|XP_005634959.1|    PRFNDILGRHSRKRWERFVHSENQHLVSPEALDFLDKLLRYDHQsRLTAREAMEHPYFYt
gi|88319941|ref|NP_777060.2|        PRFNDILGRHSRKRWERFVHSENQHLVSPEALDFLDKLLRYDHQsRLTAREAMEHPYFYt
gi|31542427|ref|NP_031814.2|        PRFNDILGRHSRKRWERFVHSENQHLVSPEALDFLDKLLRYDHQsRLTAREAMEHPYFYt
```

```
gi|16758674|ref|NP_446276.1|           PRFNDILGRHSRKRWERFVHSENQHLVSPEALDFLDKLLRYDHQsRLTAREAMEHPYFYt
gi|15227778|ref|NP_179889.1|           PnltslvGRHSRKpWtKFinSENQHLavPEAvDFvDKLLRYDHQERpTAkEAMaHPYFYP
gi|389637090|ref|XP_003716185.1|       aqydDILGRfqkKpWhsFVtaENQrfVSnEAiDFLDKLLRYDHmERLTAkEAMaHPYFeP
gi|164427285|ref|XP_964983.2|          aqydDILGRfqRKpWhsFinaENQrfVSnEAiDFLDKLLRYDHnERLTAkEAMaHPYFaP
                                                                     R306   R312      Y325-T326

gi|50311589|ref|XP_455820.1|           dfs---------------------------------------------------------
gi|6324635|ref|NP_014704.1|            kfe---------------------------------------------------------
gi|45188077|ref|NP_984300.1|           eyp---------------------------------------------------------
gi|19114554|ref|NP_593642.1|           lk----------------------------------------------------------
gi|17505290|ref|NP_492811.1|           VVeahARa-ngteqadGqgasnSAssq---------------------------------
gi|326671531|ref|XP_003199454.1|       VVKgQscfaGStNvPsvnTmVSSAtMi-----------tGItalPastaLlPLtGSPifn
gi|24668866|ref|NP_730775.1|           IVngQmnp----------------------------------------------------
gi|158294401|ref|XP_315576.4|          IVngQ--------------------------------------mPpP-------------
gi|410054859|ref|XP_003953729.1|       ---EsryL-ahrvcaGaesyrtessfl-----------SGISSVPTPSPLGPLAGSPVIA
XP_018092886.1                         IVKDQsRM-GgSNMPsGSTPVSSAsMM-----------SGIStVPTPSaLGsLAGSPVIs
XP_031750688.1                         IVKDQsRM-GgSNMPsGSTPVSSAsMMsgqsrssvrdkSGISaVPTPSaLGsLAGSPVIs
gi|50355952|ref|NP_001002242.1|        IVKDQARM-GSSNMPGGSTPVSSAsMM-----------SGISSVPTPSPLGPLAGSPVIs
gi|29570791|ref|NP_808227.1|           VVKDQARM-GSSSMPGGSTPVSSANMM-----------SGISSVPTPSPLGPLAGSPVIA
gi|297260098|ref|XP_002798227.1|       VVKDQARM-GSSSMPGGSTPVSSANMM-----------SGISSVPTPSPLGPLAGSPVIA
gi|545540258|ref|XP_005634959.1|       VVKDQARM-GSSSMPGGSTPVSSANMM-----------SGISSVPTPSPLGPLAGSPVIA
gi|88319941|ref|NP_777060.2|           VVKDQARM-GSSSMPGGSTPVSSANMM-----------SGISSVPTPSPLGPLAGSPVIA
gi|31542427|ref|NP_031814.2|           VVKDQARM-sStSMaGGSTPVSSANMM-----------SGISSVPTPSPLGPLAGSPVIA
gi|16758674|ref|NP_446276.1|           VVKDQARM-sSagMaGGSTPVSSANMM-----------SGISSVPTPSPLGPLAGSPVIA
gi|15227778|ref|NP_179889.1|           Irnae----------ssrTPrSq-------------------------------------
gi|389637090|ref|XP_003716185.1|       VrgEgv------------------------------------------------------
gi|164427285|ref|XP_964983.2|          VrdEatRa---rylaGetin----------------------------------------
                                             R333                                S356  P363

gi|50311589|ref|XP_455820.1|           -----------------------
gi|6324635|ref|NP_014704.1|            -----------------------
gi|45188077|ref|NP_984300.1|           -----------------------
gi|19114554|ref|NP_593642.1|           -----------------------
gi|17505290|ref|NP_492811.1|           ----------ssdakidGA----
gi|326671531|ref|XP_003199454.1|       slN-------tPVPAAtGAtQ--
gi|24668866|ref|NP_730775.1|           -----------------nnQQ--
gi|158294401|ref|XP_315576.4|          -----------PtssAkGgtn--
gi|410054859|ref|XP_003953729.1|       AANPLGclfqLPlrsAvtAlsvS
XP_018092886.1                         AtNtLG----tPVaAAAGAtQ--
XP_031750688.1                         AtNtLG----tPVaAAAGAtQ--
gi|50355952|ref|NP_001002242.1|        AtttLG----MPVPAAAGAQQ--
gi|29570791|ref|NP_808227.1|           AANPLG----MPVPAAAGAQQ--
gi|297260098|ref|XP_002798227.1|       AANPLG----MPVPAAAGAQQ--
gi|545540258|ref|XP_005634959.1|       AANPLG----MPVPAAAGAQQ--
gi|88319941|ref|NP_777060.2|           AANPLG----MsVPAAAGAQQ--
gi|31542427|ref|NP_031814.2|           AANsLG----IPVPAAAGAQQ--
gi|16758674|ref|NP_446276.1|           AANsLG----IPVPAAAGAQQ--
gi|15227778|ref|NP_179889.1|           -----------------------
gi|389637090|ref|XP_003716185.1|       ----------LerllAAGtntsA
gi|164427285|ref|XP_964983.2|          -----------------------
                                                  P382
```

Accession	Organism	Common name or disease	Classification
gi|50311589|ref|XP_455820.1|	Kluyveromyces lactis	Milk yeast	Yeast and Fungi
gi|6324635|ref|NP_014704.1|	Saccharomyces cerevisiae	Baker's yeast	
gi|45188077|ref|NP_984300.1|	Eremothecium gossypii	Filamentous fungus	
gi|19114554|ref|NP_593642.1|	Schizosaccharomyces pombe	Fission yeast	
gi|17505290|ref|NP_492811.1|	Caenorhabditis elegans	Nematode roundworm	Invertebrates
gi|326671531|ref|XP_003199454.1|	Danio rerio	Zebrafish	Vertebrates
gi|24668866|ref|NP_730775.1|	Drosophila melanogaster	Fruit fly	Invertebrates
gi|158294401|ref|XP_315576.4|	Anopheles gambiae	African malaria mosquito	
gi|410054859|ref|XP_003953729.1|	Pan troglodytes	Chimpanzee	Vertebrates
XP_018092886.1	Xenopus laevis	African claw-toed frog	
XP_031750688.1	Xenopus tropicalis	Western clawed frog	
gi|50355952|ref|NP_001002242.1|	Gallus gallus	Chicken	
**gi|29570791|ref|NP_808227.1|**	**Homo sapiens**	**Human**	
gi|297260098|ref|XP_002798227.1|	Macaca mulatta	Rhesus monkey	
gi|545540258|ref|XP_005634959.1|	Canis lupus familiaris	Domestic dog	
gi|88319941|ref|NP_777060.2|	Bos taurus	Cow	
gi|31542427|ref|NP_031814.2|	Mus musculus	Mouse	
gi|16758674|ref|NP_446276.1|	Rattus norvegicus	Rat	
gi|15227778|ref|NP_179889.1|	Arabidopsis thaliana	Thale cress	Plant
gi|389637090|ref|XP_003716185.1|	Pyricularia oryzae	Rice blast disease fungus	Yeast and Fungi
gi|164427285|ref|XP_964983.2|	Neurospora crassa	Red bread mold	
